# Integrated Proteomics and Metabolomics Link Acne to the Action Mechanisms of Cryptotanshinone Intervention

**DOI:** 10.3389/fphar.2021.700696

**Published:** 2021-09-01

**Authors:** Zhaoming Zhu, Tingting Chen, Zhuxian Wang, Yaqi Xue, Wenfeng Wu, Yuan Wang, Qunqun Du, Yufan Wu, Quanfu Zeng, Cuiping Jiang, Chunyan Shen, Li Liu, Hongxia Zhu, Qiang Liu

**Affiliations:** ^1^ School of Traditional Chinese Medicine, Southern Medical University, Guangzhou, China; ^2^ Integrated Hospital of Traditional Chinese Medicine, Southern Medical University, Guangzhou, China

**Keywords:** Cryptotanshinone1, Acne2, label-free Proteomic3, metabolomics4, action mechanisms5

## Abstract

The label-free methods of proteomic combined with metabolomics were applied to explore the mechanisms of Cryptotanshinone (CPT) intervention in rats with acne. The model group consisted of rats given oleic acid (MC), then treated with CPT, while control groups did not receive treatment. The skin samples were significantly different between control, model and CPT-treated groups in hierarchical clustering dendrogram. Obvious separations of the skin metabolic profiles from the three groups were found through PCA scoring. In total, 231 and 189 differentially expressed proteins (DEPs) were identified in MC and CPT groups, respectively. By the KEGG analysis, five protein and metabolite pathways were found to be significantly altered. These played important roles in response to oleic acid-induced acne and drug treatment. CPT could negatively regulate glycolysis/gluconeogenesis and histidine metabolisms to decrease keratinocyte differentiation and improve excessive keratinization and cellular barrier function. CPT could down-regulate the IL-17 signaling pathway and regulate the acne-driven immune response of sebum cells. The biosynthesis of unsaturated fatty acids metabolism, glycerophospholipid metabolism and linoleic acid pathways could significantly alter sebum production and control sebaceous gland secretion after CPT treatment. The gap junction was up-regulated after CPT treatment and the skin barrier turned back to normal. Krt 14, Krt 16 and Krt 17 were significantly down-regulated, decreasing keratinization, while inflammatory cell infiltration was improved by down-regulation of Msn, up-regulation of linoleic acid and estrogen pathways after CPT treatment. These results propose action mechanisms for the use of CPT in acne, as a safe and potential new drug.

## Introduction

Acne vulgaris has become one of the most common skin diseases (Oules et al., 2020) since more than 85% of teenagers and young adults have been affected worldwide (Kang et al., 2015). Acne vulgaris is considered a chronic skin inflammation caused by pilosebaceous (Saurat, 2015), and sebaceous glands (SG) abnormally increased in the hair follicles (HF) (Li Z. et al., 2021). Although the pathogenesis of acne remains unclear, four major factors are involved, namely: excessive sebum growth, excessive hair follicle keratinization, skin bacteria colonization and skin inflammation (O’Neill and Gallo, 2018; Harper, 2020).

Although oral isotretinoin is an effective therapy, its use is limited by adverse effects (Markovics et al., 2019). Thus, more research is needed to find new, safe and effective therapeutic drugs with few side effects (Williams et al., 2012). Cryptotanshinone (CPT), a major lipophilic compound extracted from *Salvia miltiorrhiza* demonstrated various pharmacological effects, including anti-tumor (Li H. et al., 2021), anti-inflammatory (Tang et al., 2018), anti-bacterial, anti-proliferation (Ashrafizadeh et al., 2021), anti-androgen (Xu et al., 2012), pulmonary fibrosis protection, cardio protection, anti-metabolic disorders, anti-angiogenic (Maione et al., 2018) and liver protection (Nagappan et al., 2020). In addition, it is often used to treat multiple chronic diseases, including angiocardiopathy, hyperlipidemia, acne vulgaris and chronic renal failure, with few side effects (Rahman et al., 2016; Zhang et al., 2019). Previous research has improved CPT dosage forms to strengthen its anti-acne activity (Yu et al., 2016; Zuo et al., 2016; Wang et al., 2020), leading to the development of CPT cerasomes, CPT ethosomes and 3D-Printed CPT niosomal hydrogel. Our group has made contributions to these studies (Ruan et al., 2020). However, the underlying mechanisms of the anti-acne effects of CPT have not been studied yet.

As a high-throughput technology, omics technique has been widely used in drug discovery (Tricarico et al., 2019; Worheide et al., 2021). Indeed, high-throughput omics techniques are being used to screen and identify specific molecular biomarkers for CPT in acne. Proteomics can discover biomarkers to illuminate the underlying mechanisms and reveal novel diagnostic and therapeutic targets by investigating the profile of protein alterations (Nesvizhskii, 2014). We have previously used proteomics to elucidated the potential mechanisms of Licorice flavonoid in acne (Ruan et al., 2020). Quanico J et al. have studied the response pathways and pathophysiological differences for microcysts and papule lesions of acne using proteomic and transcriptomic techniques (Quanico et al., 2017). These results have contributed to identifying targeted therapy for acne vulgaris. However, the pathogenesis of acne is very complex and, in addition to inflammation, there should be other mechanisms such as bacteria, sebum and androgens. Nevertheless these issues have not been elucidated yet, while the development of anti-acne drugs based on proteomics is still a rare practice.

Our study was designed to find underlying molecular mechanisms of CPT treatment in a rat acne model through the use of label-free quantitative proteomics and metabolomics. Indeed, metabolomic can complement the proteomic analysis and represent disease phenotype (Gertsman and Barshop, 2018). Metabolomics can therefore be used effectively in diagnosing and identifying therapeutic targets of diseases and investigating the mechanisms of biological processes (Patti et al., 2012). Metabolites can be regulated by proteins, while, protein expression may also be influenced by metabolites (Ma et al., 2020). The functional interpretation of proteomics by metabolomics facilitates the comprehension of the investigated biological phenomenon (Gui et al., 2018). Proteomics mainly determines biological functions and regulatory mechanisms, while metabolites are the main source of substances and the basis of phenotypes. Proteomics studies can only explain the function and mechanism, but lack a direct description of phenotype (Monti et al., 2019). In this study, we compared the differential protein expression between rats with acne and rats with acne treated with CPT, and used the KEGG pathway enrichment analysis to assess the differential metabolic pathways between the two groups. Finally, we tried to explain the differential pathways with metabolomics analysis to illustrate the mechanism for CPT intervention in acne.

## Materials and Methods

### Materials

Cryptotanshinone (Lot number 17071601, purity ≥98%, high-performance liquid chromatography; HPLC; Baoji Herbest Bio-Tech Co., Ltd., Baoji, China), carbomer 940 (Macklin Biochemical Co., Ltd., Shanghai, China), glycerin (Aladdin Chemical Reagent Co., Ltd., Shanghai, China), pentobarbital (Sigma, United States), oleic acid (Macklin Biochemical Co., Ltd., Shanghai, China), other chemical substances were analytically pure (AR).

### Preparation of CPT Gel

The CPT was dissolved with anhydrous ethanol (sonication for 1 min) and was filtered with 0.22 μm filters. Then, we took 3 g of carbomer 940, dissolved in 100 ml pure water for 24 h. Next, 3 g glycerol and CPT were dissolved in ethanol. Followed by addition of triethanolamine. The CPT gel was an orange transparent semi-solid preparation with a final CPT concentration of 2.2 mg/g.

### Acne Model and CPT Treatment

Adult male SD (Sprague-Dawley) rats (weight: 200 ± 20 g) were acquired from the Experimental Animal Center of the Southern Medical University (SMU). After a 1-week adaptation, rats were randomly separated into three groups: Blank control group (BC), Model group (MC) and CPT treatment group (CPT) with eight rats per group. Three percent sodium pentobarbital was used to anesthetize the rats. The back hair of the rats was removed for further study. Then, 0.5 ml of 80% oleic acid was evenly smeared on the back skin of each rat, for 14 days once per day, except for the BC group (Zuo et al., 2016). CPT gels were then applied to the back skin of CPT rats once per day for 1 week. The procedures of this research were in accordance to the Guide for the Care and Use of Laboratory Animals (Worlein et al., 2011) (eighth versions, revised in 2011), which was approved by the Laboratory Animal Ethics Committee of SMU.

### Histopathological Examination

Rat dorsal skin tissues were fixed and stored in a 10% formaldehyde solution. Then the skin tissues were dehydrated in a gradient of 80–100% alcohol. Then the tissues were embedded with paraffin. The paraffin blocks were cut into 3–5 μm thickness sections. Next Hematoxylin-eosin (HE, Sigma) was used for the section dyeing. Histopathological sections were analyzed by an optical microscope (magnifications ×200 and ×100: type BX53, Olympus; magnifications ×40: type Eclipse E100, Nikon). The pathophysiology of acne was judged based on a previous reference (Gollnick and Dreno, 2015).

### Lesions Analysis

The stratum corneum (SC) thickness and the long diameter and short diameter of sebaceous glands (SG) of the rat skin were measured with Olympus cellSens software in histopathological sections magnified 200 times. (Li et al., 2018).

### Cytokine Levels

The serum samples from rats were tested by the ELISA kits of rat of IL-6, IL-8, TNF-α and IL-1β. According to the instructions of these kits, the OD values of IL-6, IL-8, TNF-α and IL-1β were detected at specific wavelengths, and their contents were calculated by graphpad 8.02 software (Han et al., 2018).

### Proteomics Analysis

#### Protein Extraction

The skin tissues were lysed and proteins were extracted with SDT buffer (4% SDS, 1 mM DTT, 100 mM Tris-HCl, pH = 7.6). The protein content was quantitatively analyzed by a protein-detection kit (Bio-Rad, United States). Protein digestion with trypsin was performed through the filter-aided sample preparation (FASP) procedure from Matthias Mann (Wisniewski et al., 2009). The digested peptides of every skin were desalination processing with a C18 solid phase extraction column (Empore™ SPE, 7 mm × 3 ml, Sigma). Then, the concentration under vacuum centrifugation and reconstitution was performed with 40 µL of 0.1% formic acid.

#### FASP Digestion Procedure of Skin Tissues

A total of 200 μg of proteins for each skin tissue were mixed with 30 μL of 4% SDS, 100 mM DTT, 150 mM Tris-HCl, pH = 8.0. The eluent was removed. Next, DTT and small molecule components were filtered out with a UA of 8 M Urea in 150 mM Tris-HCl, pH 8.0. Then, 100 μL of iodoacetamide in UA with 100 mM IAA was added, and the skin tissues were incubated in the dark for 30 min. Filters were washed three times with 100 μL of UA, and 100 μL of 25 mM NH4HCO3. The buffer was used to digest 4 μg of trypsin in 40 μL of 25 mM NH4HCO3 for 18 h, at 37°C, then the dissolved peptides were collected. The digested peptides were subjected to desalination processing with a C18 solid phase extraction column (Empore™ SPE, 7 mm × 3 ml, Sigma). Then, the concentration under vacuum centrifugation and reconstitution was performed with 40 µL of 0.1% formic acid. The peptides were detected with a UV light spectral (280 nm).

#### Quantitative Proteomic Analysis

Skin samples were analyzed by LC-MS/MS on a Q Exactive mass spectrometer (Thermo Scientific). The peptides were loaded onto a nano Viper C18-reversed phase trap column (Thermo Scientific Acclaim PepMap100, 100 μm × 2 cm), then they were connected to a C18-reversed phase analytical column (Thermo Scientific Easy Column, 75 μm × 10 cm, 3 μm resin). Next, 0.1%-Formic acid (A phase) and B phase (84%-acetonitrile: 0.1%-Formic acid) were used for gradient elution. The flow rate was set at 300 nL/min. Samples were analyzed with the positive ion mode. MS data were acquired from the 300 m/z to 1800 m/z dynamic survey scan mode to choose the most abundant precursor ions for higher energy collisional dissociation fragmentation. Automatic gain control target was set to 3×10^6^, and maximum injection time was 10 ms. Dynamic exclusion duration was 40.0 s. Survey scans were acquired at a resolution of 70,000 atm/z 200. The resolution for higher energy collisional spectra was set to 17,500 atm/z 200. The isolation width was set at 2 m/z. Normalized collision energy was 30 eV. The under fill ratio was 0.1%. The samples were analyzed in peptide recognition mode.

#### Identification and Quantitation of Proteins

The raw data for each sample were combined and searched using the Max Quant 1.5.3.17 software. The parameters were set as follows: enzyme choosing trypsin; max missed cleavages setting two; fixed and variable modifications respectively being carbamidomethyl (C) and oxidation (M); main and first search respectively being 6 and 20 ppm; MS/MS Tolerance: 20 ppm; database pattern: Reverse; Include contaminants: True; protein and peptide FDR both being ≤0.01; peptides used for protein quantification using a razor and unique peptides; time window being set at 2 min; minimum ratio count of 1. The *p*-value was obtained with the *t*-test. Proteins were significantly changed if fold changes >2.0, or <0.5, and *p* < 0 0.05, which were considered as DEPs. In the same way, the SDEPs were set as fold changes >2.0, or <0.5, and *p* < 0.01 (O'Connell et al., 2018). The mass spectrometry proteomics data have been deposited to the ProteomeXchange Consortium (http://proteomecentral.proteomexchange.org) *via* the iProX partner repository with the dataset identifier: PXD027219.

#### Proteomic Bioinformatics Analysis

Cluster analysis of phosphorylated peptides was performed by Cluster 3.0 and Java Treeview softwares. Z-score (label-free or metabolism) mode was set as the standard method. The distance type was set as euclidean, and the clustering algorithm was set as average. Then, GO annotation of the differentially expressed proteins (DEPs) was performed with the software program Blast 2 GO, and the top 20 terms of GO enrichment results were drawn in a bar graph. The top 20 Kyoto Encyclopedia of Genes and Genomes (KEGG) enrichment pathway (*p*-value < 0.05) terms were searched in the KEGG database. Parameters were set as follows: graphic style: bar graph; data type: proteome; screening data: standardized processing; *p*-value/FDR: *p*; *p*-value threshold: 0.05; legend style: standardized processing. Proteins that met the fold change (FC) > 2.0, or FC < 0.5, and *p* < 0.05 were considered DEPs. Both FC > 2.0 and *p* < 0.01 values were considered to be SDEPs (Chen et al., 2020).

### Metabolomics Analysis

#### Chemicals

Ammonium acetate (NHAC), ammonium hydroxide (NH_4_OH), ammonium fluoride (NHaF), and formic acid (FA) were obtained from Sigma Aldrich. Acetonitrile was obtained from Merck.

#### Skin Samples Preparation for Metabolomic

The skin tissues were immediately frozen in liquid nitrogen. Then the samples were cut on 80 mg of dry ice into a 2 ml tube. The skin tissues with five ceramic beads were homogenized. 1 ml methanol-acetonitrile aqueous solution (2:2:1, V/V) was added to the homogenized solution for metabolite extraction. This mixture was centrifuged for 30 min (14,000 × g, 4°C), twice, and placed at −20°C for 1 h to precipitate the proteins. The samples were filtered with a filter tube and centrifuged at 4°C for 20 min, the supernatant was freeze-dried and kept at −80°C. The prepared samples were dissolved in 100 uL acetonitrile/water (1:1,v/v) solvent and analyzed by LC-MS(Gao et al., 2021).

#### Metabolomic Analysis

Metabolomic analysis was performed by a UHPLC (1290 Infinity LC, Agilent Technologies) coupled to a quadrupole time-of-flight (AB Sciex Triple TOF 6600) in HILIC separation. The samples were analyzed with a water column of Ireland (ACOUIY UPLC BEH, 2.1 mm × 100 mm, 1.7 um). The positive and negative modes were set. The mobile phase A was 25 mM ammonium acetate and hydroxide (1:1,v/v) in water, and the mobile phase B was acetonitrile. The elution gradient was set as follows: 0–0.5 min: 95% B; 0.5–7 min: 95% B- 65% B; 7–7.1 min: from 65% B to 40% B; 7.1–8.1 min: 40% B; 8.1–8.2 min: 40% B-95% B; 8.2–12.2 min: 95% B re-balanced time for employing.

An Ireland water column was used (ACQUIY UPLC HSS T3, 2.1 mm × 100 mm, 1.8 um) for sample RPLC separation. In the positive ion mode, mobile phase A was 0.1%-formic acid aqueous solution, and phase B was acetonitrile: 0.1% formic acid. In the negative ion mode, mobile phase A was 0.5 mM ammonium fluoride aqueous solution, and phase B was acetonitrile. The elution gradient was set as follows: 0–1.5 min: 1% B; 1.5–11.5 min: 1% B-99% B; 11.5–15 min: 99% B; 15–15.1 min: 1% B; 15.1–18.5 min: 1% B re-balanced time for employing. The flow rate was set at 0.3 ml/min, then the temperature of the column was set at 25°C. The injection volume of every sample was 2 μL. The sample was placed at a 4°C automatic sampler throughout the analysis process (Yi et al., 2019; Wang et al., 2021).

A series of parameters were set as follows: Gas1: 60, Gas2: 60, CUR: 30, source temperature: 600°C, ISVF: ±5500 V (positive and negative). For single MS acquisition, the m/z range of the instrument was set as 60–1000 Da, and the scanning cumulative time of TOF MS was set as 0.20 s/spectra. For automatic MS/MS acquisition, the m/z range of the instrument was set as 25–1000 Da, and the scanning cumulative time of the product ions was set as 0.05 s/spectra. The information-dependent acquisition tech was applied in the product ion scan, and the mode was set as high sensitivity. The parameters were set as follows: collision energy: 35 V with ±15 eV; declustering potential: ± 60 V; exclude isotopes: ≤ 4 Da, and the candidate ion to be monitored for each period was set to 6.

### Data Processing

The raw MS data were converted to MzXML files with Proteo Wizard MS Convert before importing into freely available XCMS software. Compound identification of metabolites was performed by comparing the accuracy m/z value (<10 ppm), and MS/MS spectra with an in-house database established with available authentic standards. Principle component analysis (PCA) was performed with SIMCA (Version 14.1) using quantitative data.

### KEGG Pathway Analysis

The DEMs were searched and the KEGG enrichment pathways were obtained with the online KEGG database (http://geneontology.org/). The annotation and enrichment results of KEGG were used in R 3.5.1 version. Next, the Venn diagram and bar plot were drawn.

### Statistical Analysis

The *p*-values of proteins and metabolites were obtained with the *t*-test. Significantly changed proteins (fold changes >2.0, or <0.5, and *p* < 0 0.05) were considered DEPs. In the same way, SDEPs had fold changes >2.0, or <0.5, and *p* < 0.01 (O'Connell et al., 2018). Significantly altered metabolites (VIP value >1.0, and *p* < 0.05) were considered DEMs. In the same way, SDEMs had VIP value >1.0, and *p* < 0.01 (O'Connell et al., 2018). All data are presented as mean ± SD. One-way analysis of variance (ANOVA) was used to analyze the data between BC, MC and CPT groups. GraphPad Prism software (version 8.02) was used to perform this statistical analysis. Differences were considered to be statistically significant when *p* < 0.05.

## Results

### Histopathological Examination

**FIGURE 1:**
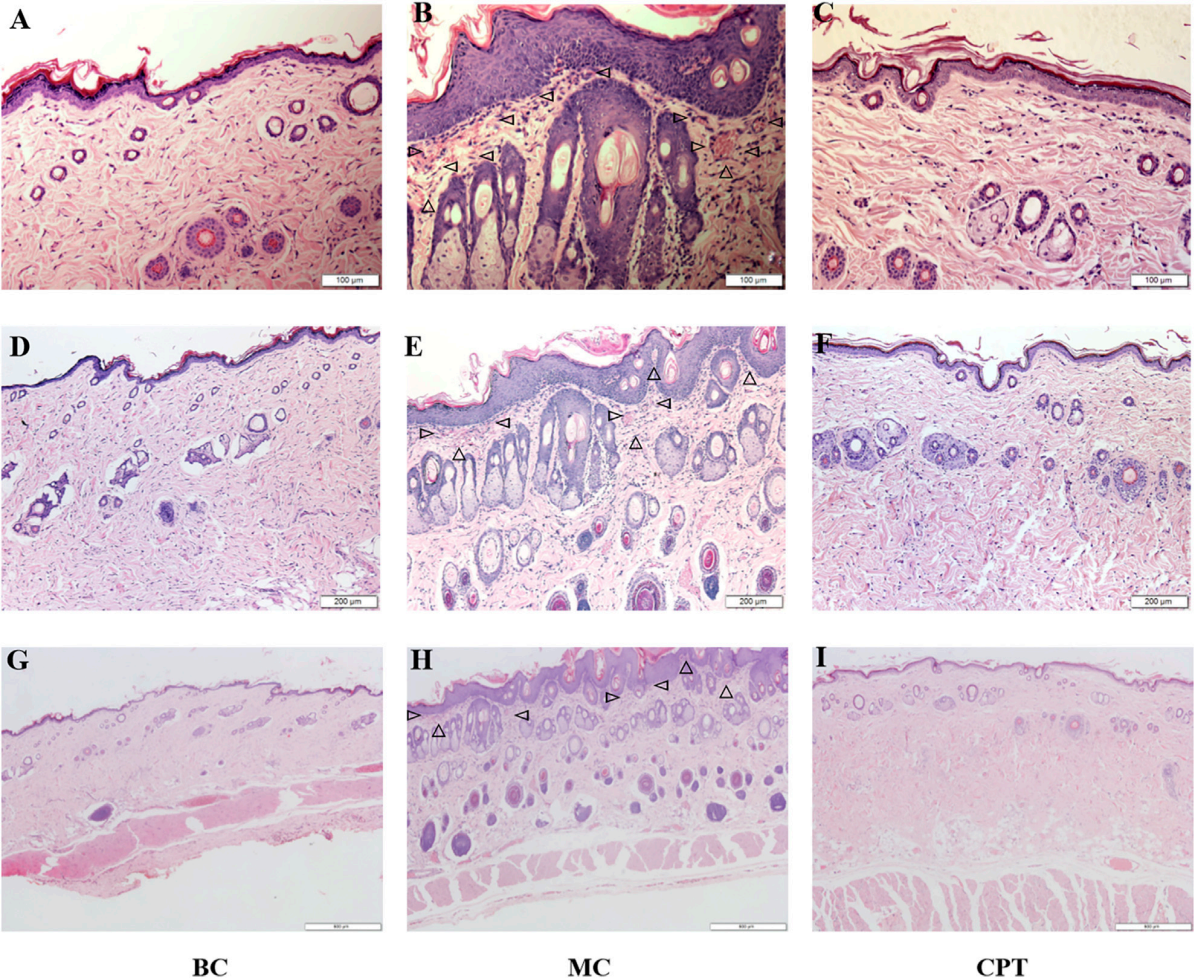
The skin tissues of CPT treatment in acne (HE, magnification ×200, ×100 and ×40) **(A)**, **(B)** and **(C)**: magnification ×200 (100 μm) **(D) (E)** and **(F)**: magnification ×100 (200 μm) **(G)**, **(H)** and **(I)**: magnification ×40 (500 μm) **(A)**, **(D)** and **(G)**: BC (blank control) group; **(B)**, **(E)** and **(H)**: MC (model) group; **(C)**, **(F)** and **(I)**: CPT (treatment) group; the triangles in MC group represent neutrophilic inflammatory cell infiltration.

Histopathological examination of the skin tissues is shown in Figure 1. The epidermal squamous epithelium layer was significantly thickened, and the epithelial cells in the funnel-shaped part of the hair follicle were increased. Follicle pores were blocked due to excessive keratinization of the hair follicle and we observed dermis hyperemia, neutrophilic cell infiltration, and enlarged sebaceous glands in the model control (MC) group (Figure 1 MC) compared with the blank control (BC) group (Figure1 BC). MC rats were treated with CPT (Figure1 CPT) and showed skin tissue similar to the BC group (Figure1 BC) with reduced keratinization. The inflammatory cells were decreased, and the sebaceous glands were normal (Figure1 CPT).

### CPT Improved Acne

**FIGURE 2:**
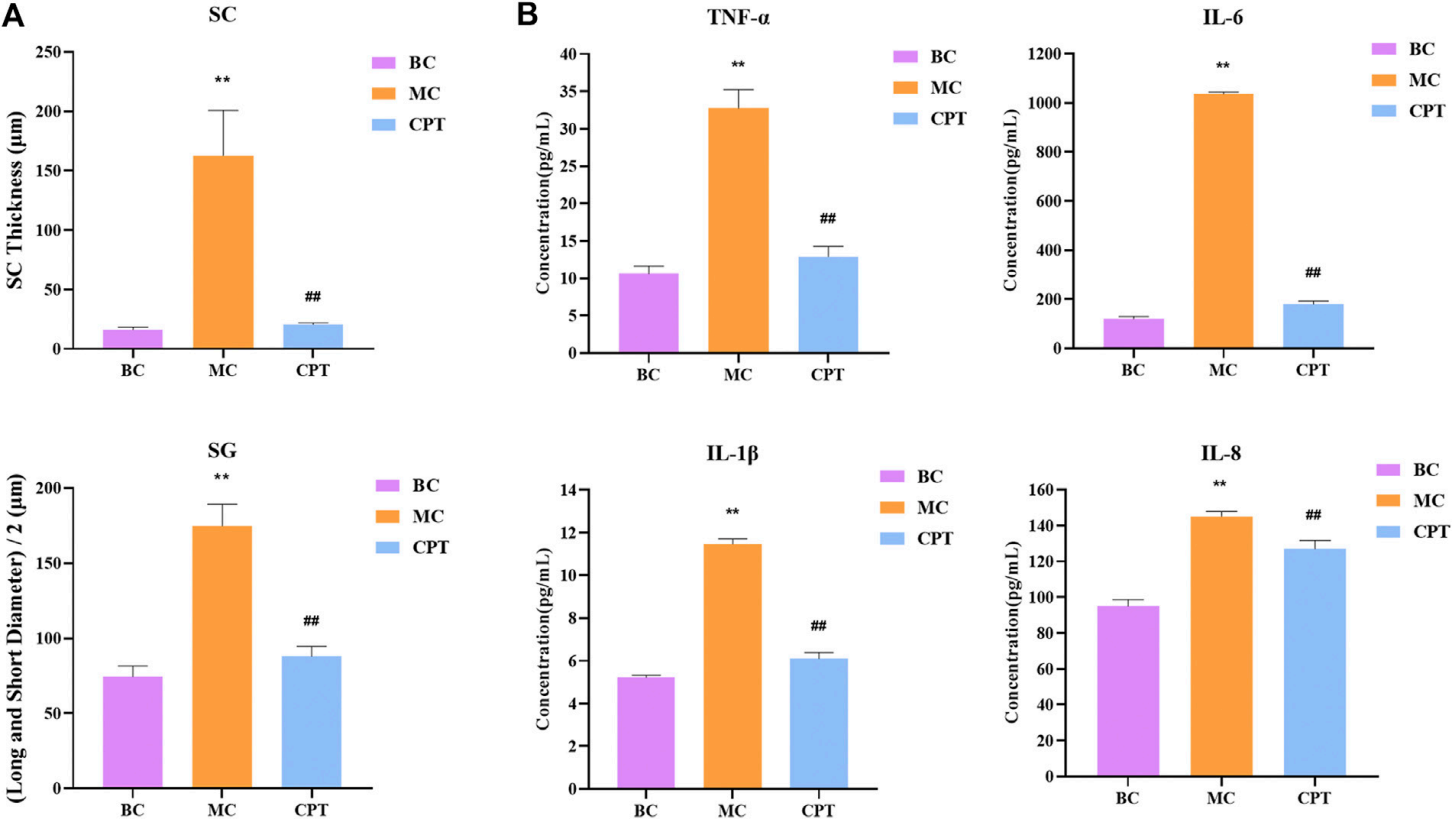
**(A)** Bar graph of stratum corneum (SC) thickness and half of long diameter and short diameter of sebaceous glands (SG); original magnification, ×200 **(B)** Cytokine levels in serum of IL-6, IL-8, TNF-α and IL-1β in the BC, MC and CPT groups. ^**^, *p* < 0.01, compared with the BC group; ^##^, *p* < 0.01, compared with the CPT group.

Compared with the BC group, in the model group, the SC thickness of rat skin was significantly increased. Compared with the MC group, after CPT treatment, the SC thickness of rat skin was significantly reduced. The diameter of SG in the model group was significantly increased, while that in the CPT group was significantly decreased (Figure 2A).

The serum contents of IL-6, IL-8, TNF-α and IL-1β were significantly higher in the MC group (p < 0.05), whereas the contents were significantly lower in the CPT group (*p* < 0.05). The results showed that the highly expressed cytokine levels in model group rats could be reduced by CPT, thus inhibiting the inflammation of the acne rats (Figure 2B).

### Analysis of Differentially Expressed Proteins (DEPs) and Significantly Differential Proteins (SDEPs)

**FIGURE 3:**
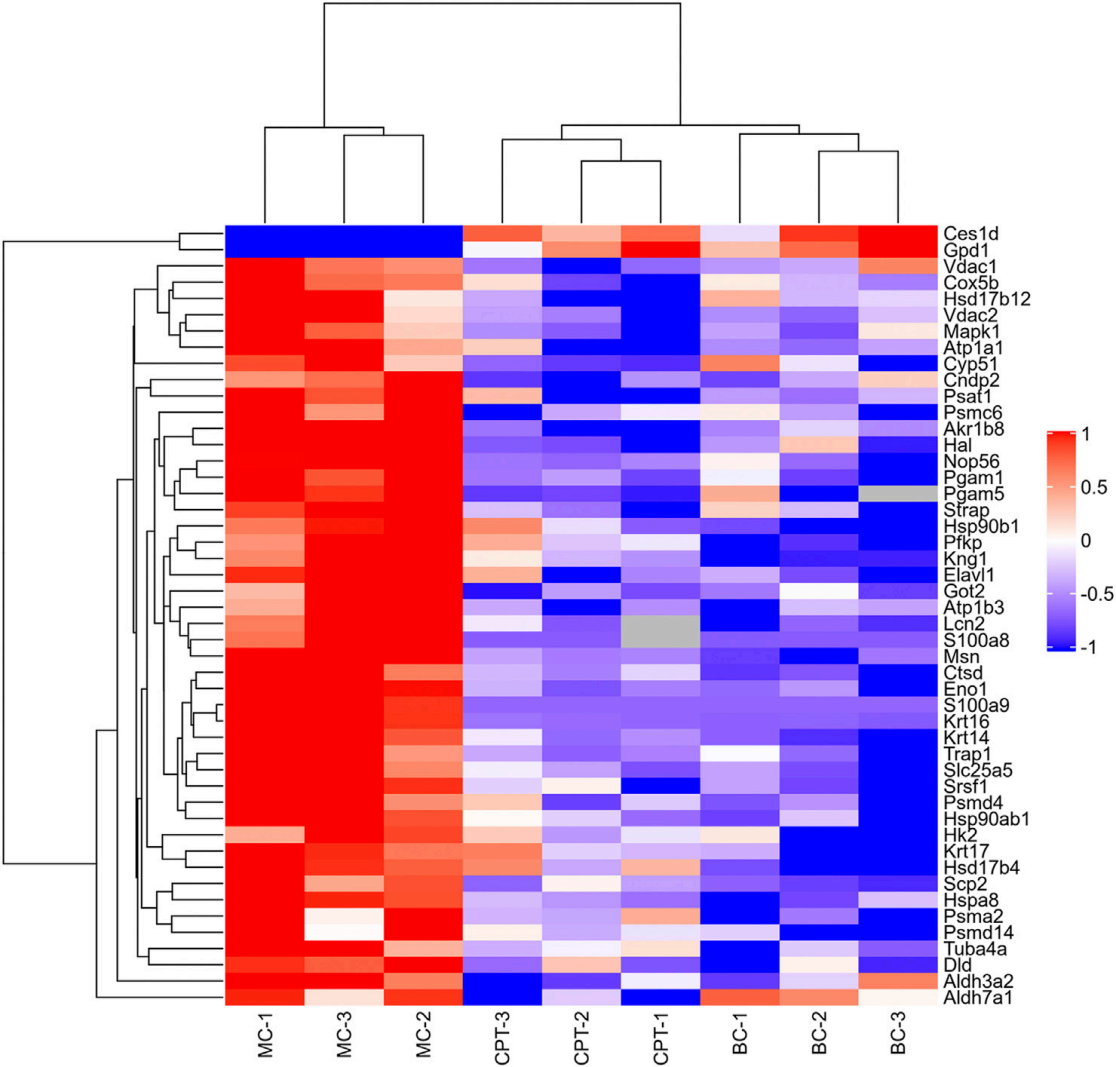
Hierarchical clustering heat map of DEPs of the skin. Changed proteins (right side) and the samples in different groups (bottom). The color from red to blue shows the relative intensity of the DEPs.

Altogether, 3,127 proteins and 25,216 peptides were identified, and 2,869 proteins were quantitatively analyzed (Supplementary Tables 1,2). A total of 231 DEPs (fold change >2, or <0.5, and *p* < 0.05) and 89 SDEPs (fold change >2, or <0.5, and *p* < 0.01) (Yan et al., 2020), were identified of which 214 DEPs (82 SDEPs) in MC were up-regulated and 17 DEPs (7 SDEPs) were down-regulated compared to BC (Supplementary Table 3). A total of 189 DEPs and 103 SDEPs were changed in MC and CPT groups, respectively. A total of 189 DEPs, 72 DEPs (36 SDEPs) were up-regulated and 117 DEPs (67 SDEPs) were down-regulated in MC and CPT group, respectively (Supplementary Table 4). The DEPs were presented by a cluster heat map. Interestingly, the hierarchical clustering figure showed that the skin proteins in the MC group were significantly separated from those in the BC group and the CPT treatment group, and that the CPT group was significantly closer to the BC group (Figure 3).

### GO Enrichment Analysis

**FIGURE 4:**
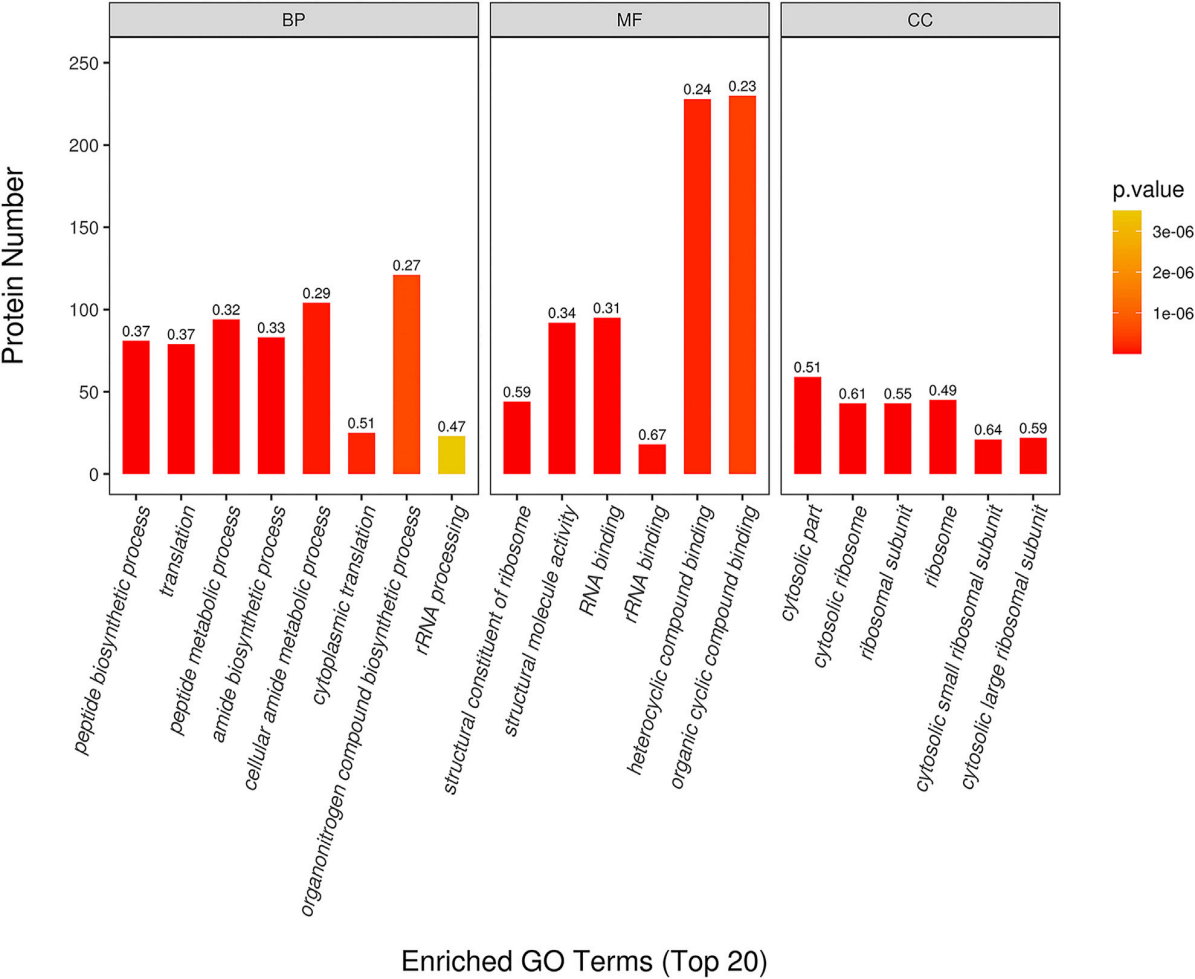
GO enrichment of DEPs in CPT and MC. The *x*-axis represents the Biological Process (BP), Cellular Component (CC), and Molecular Function (MF). The *y*-axis represents gene number, the numbers represent the enrichment factor of DEPs ratio.

The top 20 of GO enrichment analysis are shown in Figure 4. The result showed that DEPs were significantly enriched in several biological processes, molecular functions and cellular component categories (*p* < 0.05) in CPT compared to those of the MC group. DEPs from CPT participated in the biological processes of peptide biosynthesis and metabolic process regulation, translation regulation, amide biosynthesis process, cellular amide metabolic process regulation, cytoplasmic translation, organonitrogen compound biosynthetic process and rRNA processing (Figure 4). These DEPs were related to the regulation of molecular functions, such as structural constituent of ribosome, structural molecule activity, rRNA and RNA binding, heterocyclic compound binding and organic cyclic compound binding. The main enriched cellular components in CPT *versus* model group were the cytosol, cytosolic ribosome, ribosomal subunit, ribosome, cytosolic small and large ribosomal subunit. There was a significant difference in GO enrichment between MC and CPT groups, which was related to acne.

### KEGG Pathway Enrichment Analysis

**FIGURE 5:**
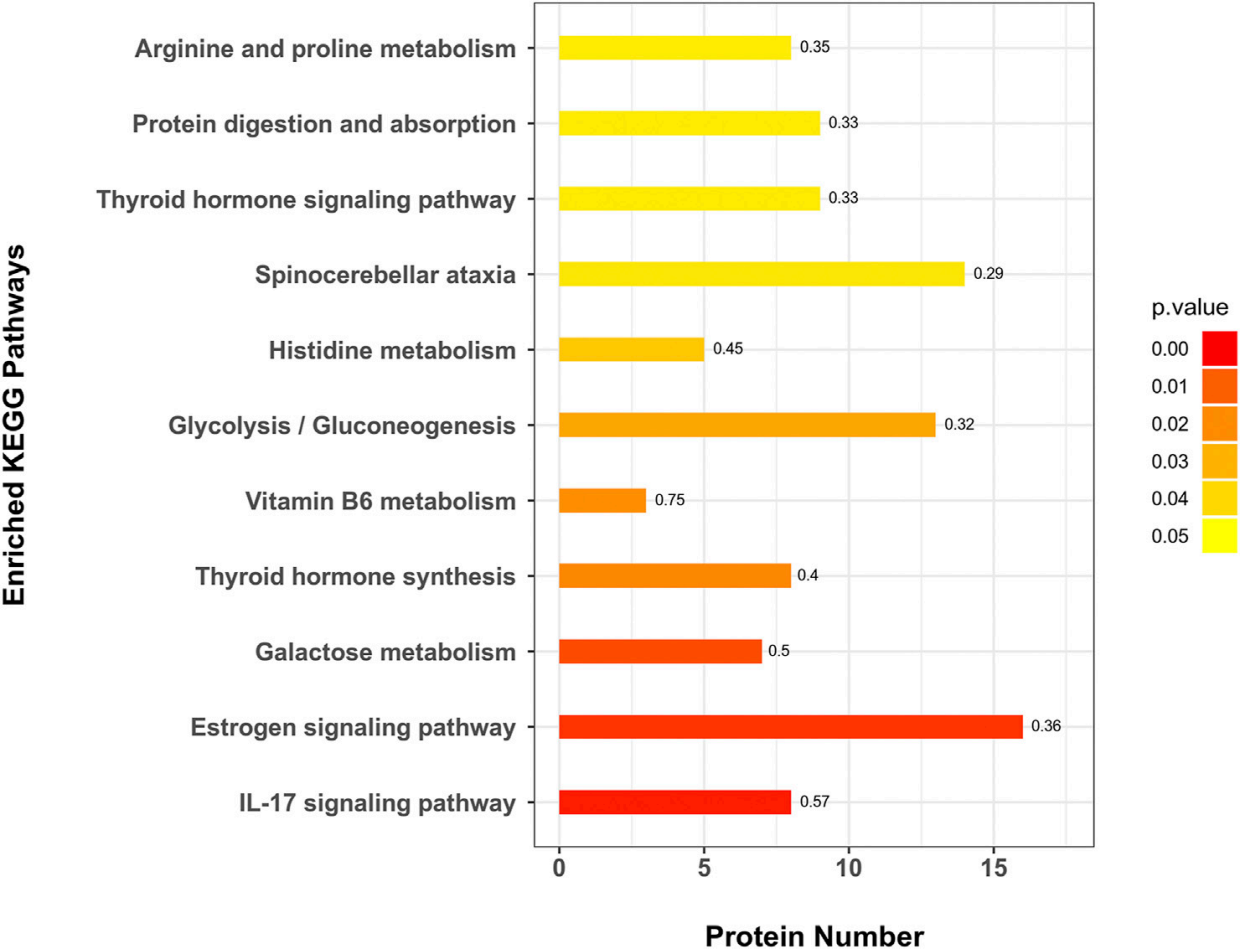
KEGG pathway enrichment of DEPs in CPT and MC groups. The *x*-axis represents the amount of DEPs, the *y*-axis represents the pathway and the numbers represent the enrichment factor ratio of DEPs.

A total of 271 protein pathways were enriched through the KEGG analysis, of which 28 pathways were changed (*p* < 0.05) in the MC and CPT treatment groups (Supplementary Table 5). As shown in Figure 5, the most enriched pathways in CPT *versus* MC group were glycolysis/gluconeogenesis, spinocerebellar ataxia, galactose metabolism, histidine metabolism, IL-17 signaling pathway, protein digestion and absorption, estrogen signaling pathway, thyroid hormone signaling pathway, thyroid hormone synthesis, arginine and proline metabolism (consisting of 13, 14, 7, 5, 8, 9, 16, 9, 8 and 8 proteins, respectively). Interestingly, the IL-17 signaling pathway, which is closely related to the pathogenesis of acne (Bernardini et al., 2020), included seven DEPs enriched in CPT *versus* the MC group.

### Metabolomics Analysis

#### Principle Component Analysis (PCA) Score of Skin Samples

**FIGURE 6:**
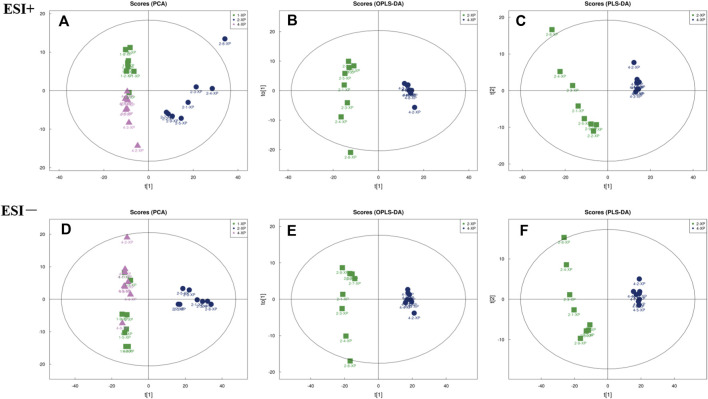
The multivariate analysis of skin metabolites in positive (ESI+) and negative (ESI−) modes (n = 8); 1-XP (the BC group), 2-XP (the MC group), 4-XP (the CPT group) **(A)** PCA score plots in positive modes of the BC, MC and CPT groups. Samples in BC and CPT groups were separated from the MC group; ESI+: R^2^X = 0.548 **(B)** OPLS-DA score plots in positive modes of the MC and CPT groups; ESI+: R^2^X = 0.559, R^2^Y = 0.989, Q^2^ = 0.944 **(C)** PLS-DA score plots in positive modes of the MC and CPT groups; ESI+: R^2^X = 0.559, R^2^Y = 0.989, Q^2^ = 0.962 **(D)** PCA score plots in negative modes of the BC, MC and CPT groups; ESI−: R^2^X = 0.638 **(E)**OPLS-DA score plots in negative modes of the MC and CPT groups; ESI−: R^2^X = 0.689, R^2^Y = 0.986, Q^2^ = 0.970 **(F)** PLS-DA score plots in negative modes of the MC and CPT groups; ESI−: R^2^X = 0.729, R^2^Y = 0.997, Q^2^ = 0.980.

The PCA score plots were plotted and the results (Figure 6A and Figure 6D) showed that the BC, MC and CPT groups were significantly separated in the positive and negative modes. The skin samples of each group were closely clustered in the PCA score plots. The MC group was significantly separated from both the BC and the CPT groups, and the skin samples in the BC group were closer to the CPT group. According to the OPLS-DA score plots in Figure 6B and Figure 6E, the skin metabolic profiles in the MC and CPT groups were significantly separated. Also, the PLS-DA plots illustrated clear differentiation in metabolomics profiles between the MC and CPT groups as showed in Figure 6C and Figure 6F.

#### Analysis of Differentially Expressed Metabolites (DEMs)

The MS/MS total ion chromatography (TIC) and mass spectrometry (MS/MS) of the metabolites were in the Supplementary Materials 2, 3. Altogether, 484 metabolites were identified (Supplementary Table 6). A total of 77 significantly changed metabolites (VIP >1, and *p* < 0.05, DEMs) were identified. A total of 55 DEMs were up-regulated (fold change >0.67) and 22 DEMs were down-regulated (fold change <0.67) in MC compared to BC (Supplementary Table 7). A total of 76 DEMs were identified for MC and CPT. Among these DEMs, 43 metabolites in the CPT group were up-regulated, whereas 33 metabolites were down-regulated *versus* the MC group (Supplementary Table 8).

**FIGURE 7:**
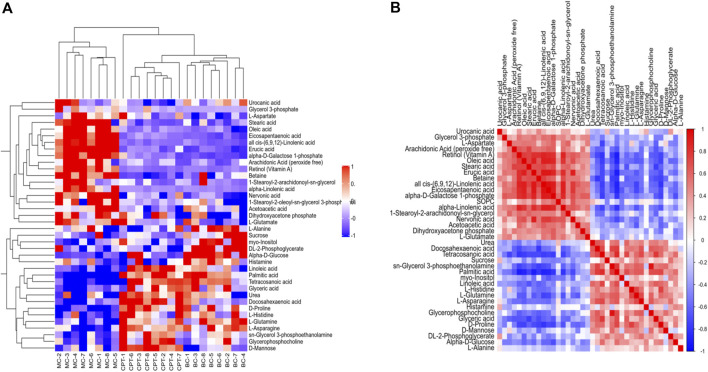
**(A)** Hierarchical clustering heat map of skin DEMs. Cluster of the DEMs (right side) and the samples of each group (bottom) are shown. The color from red to blue showed the relative intensity of the DEMs **(B)** Pearson’s correlation of DEMs in CPT and MC groups. Red and blue represent the positive and negative correlation of DEMs, respectively.

A total of 58 SDEMs (VIP >1, and *p* < 0.01) were identified in MC and CPT groups. Thirty-three metabolites were significantly increased, while 25 were significantly decreased in the MC and CPT groups (Supplementary Table 8). The hierarchical clustering dendrogram indicated that the skin metabolites of the MC group were significantly separated from both the BC and CPT groups, whereas the skin metabolites of BC and CPT groups were clustered together (Figure 7A). Metabolites with similar abundance were clustered together, representing the degree of metabolic proximities and the inner relation among the DEMs (Figure 7B). Besides, a multiple comparisons analysis was conducted in the three groups. These results indicated that, compared to the MC group, metabolites of the CPT group were similar to those of the BC group.

#### Metabolic Pathway (KEGG) Enrichment

**FIGURE 8:**
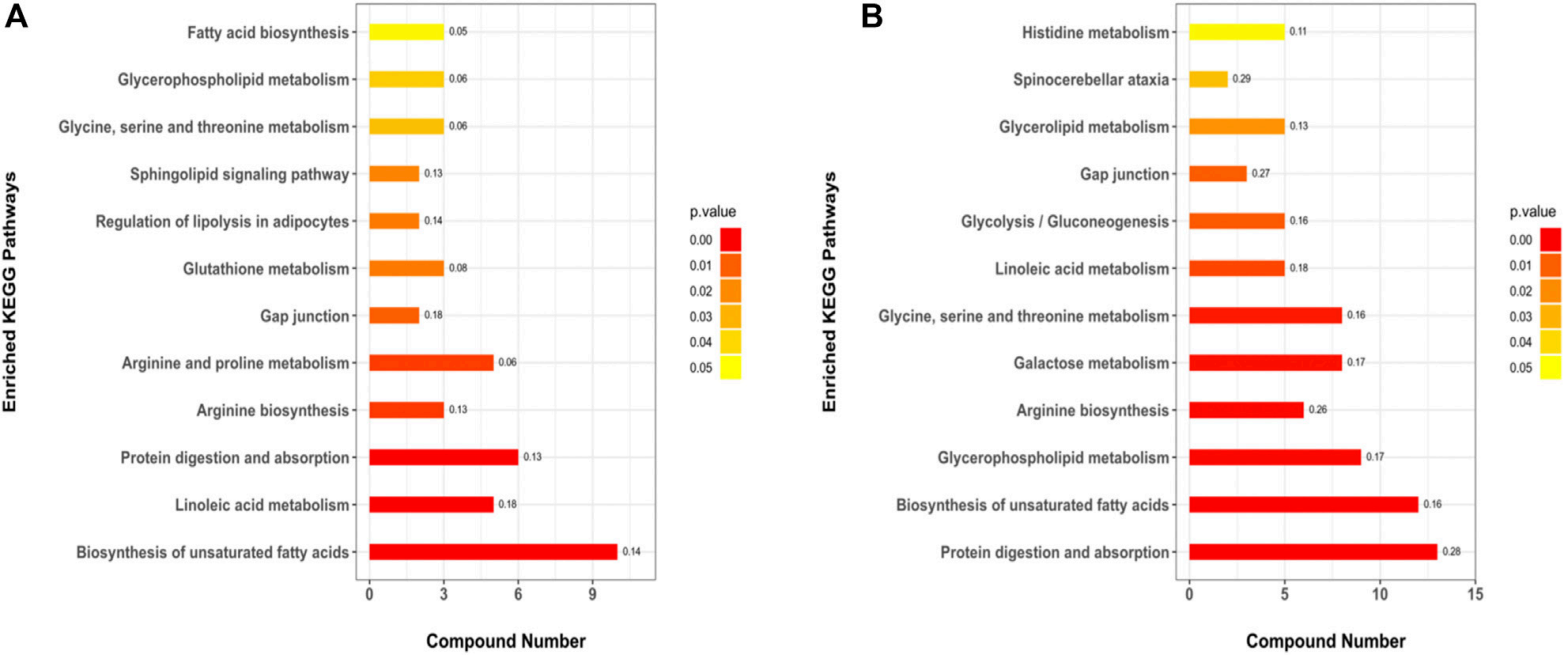
KEGG pathways of the DEMs. **(A)**: BC and MC groups, **(B)**: MC and CPT groups. The *x*-axis represents protein number, the *y*-axis represents the KEGG pathway and the numbers represent the enrichment factor ratio of DEMs.

A total of 179 metabolic pathways were enriched by the KEGG analysis, and 51 of these pathways were significantly changed (*p* < 0.05) in MC and CPT groups (Supplementary Table 9). The major metabolites participated in protein digestion and absorption, biosynthesis of unsaturated fatty acids metabolism, arginine biosynthesis, glycerophospholipid metabolism, galactose metabolism, glycine, serine and threonine metabolism, linoleic acid metabolism, glycolysis/gluconeogenesis, histidine metabolism and spinocerebellar ataxia. The metabolic pathways in CPT and MC group were closely related to the pathogenesis of acne (Figure 8).

### Combination of Proteomics and Metabolomics

**FIGURE 9:**
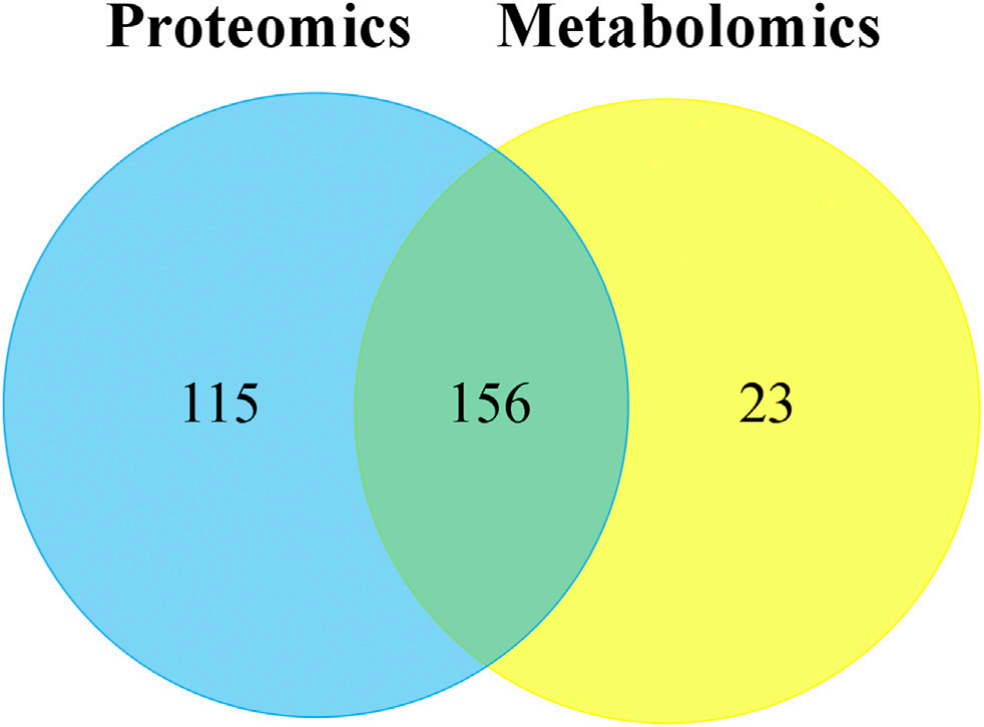
The Venn plot of common pathways of DEPs and DEMs in MC and CPT groups. The blue and yellow circles represent proteomics and metabolomics, respectively. The overlap was the number of pathways shared by the two omics analyzes. The sum of all numbers in the circle represents the sum of the number of pathways involved in DEPs and DEMs.

**FIGURE 10:**
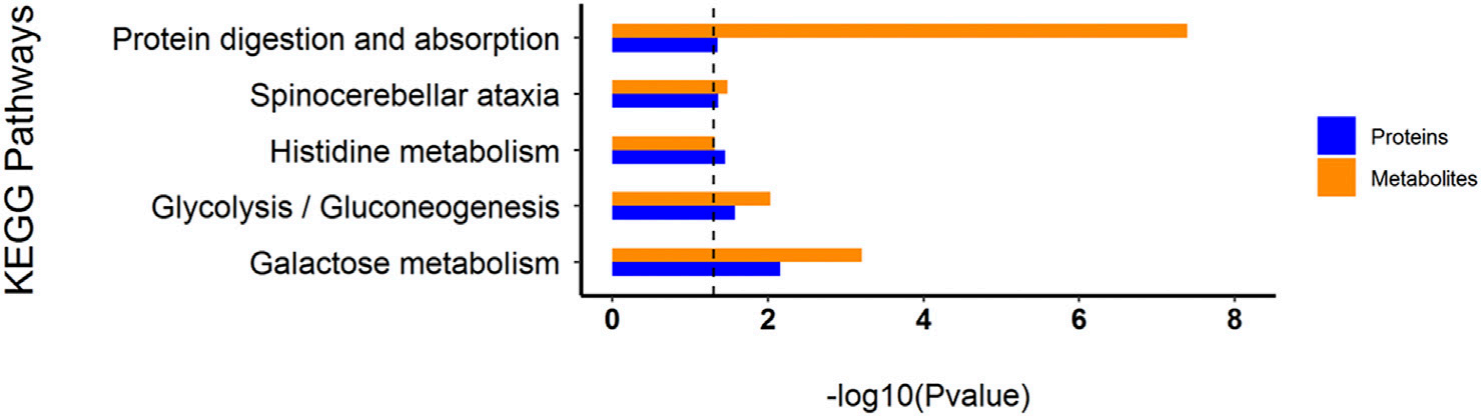
Histogram of common KEGG pathways of the DEPs and DEMs through multiple comparisons of MC and CPT groups. The blue and orange columns represent proteomics and metabolomics, respectively. The *y*-axis represents the pathway, and the *x*-axis represents the *p*-value.

**FIGURE 11:**
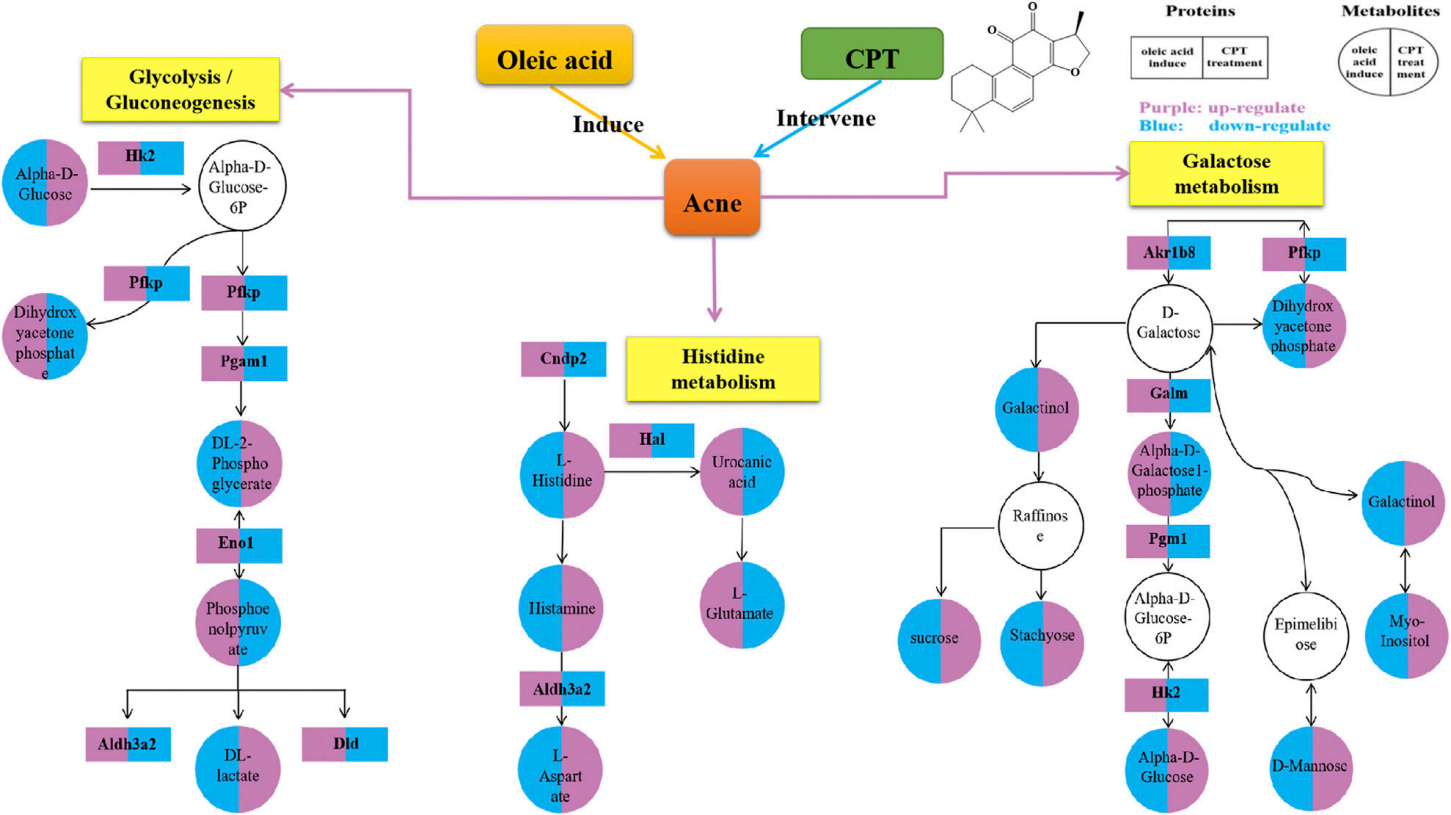
Network for a mechanistic explanation of the proteomics and metabolomics analyzes. *versus* the MC group, the purple and blue represent up-regulation and down-regulation, respectively. The yellow color represents the metabolism pathway. The DEPs and DEMs are represented by rectangles and circles, respectively.

Through the KEGG database, a total of 156 shared pathways of proteins and metabolites are shown in a Venn plot (Figure 9 and Supplementary Table 10). Altogether five pathways were significantly altered in both their proteins and metabolites contents and played important roles in acne rats and CPT-treated rats (Figure 10). These pathways included galactose metabolism, histidine metabolism, glycolysis/gluconeogenesis, spinocerebellar ataxia and protein digestion and absorption. There are regulatory relationships between these DEPs and DEMs. The regulation networks of significantly altered metabolic pathways in response to CPT treatment were assessed (Figure 11). As shown for the glycolysis/gluconeogenesis pathway and galactose metabolism, CPT regulated DEPs such as hexokinase 2(Hk2), enolase 1 (Eno1), dihydrolipoamide dehydrogenase (Dld), phosphofructokinase, platelet (Pfkp), phosphoglycerate mutase 1 (Pgam1), aldehyde dehydrogenase 3 family, member A2 (Aldh3a2) and aldo-keto reductase family 1, member B8 (Akr1b8), and DEMs such as Phosphoenolpyruvate (PEP), Dihydroxyacetone phosphate, dl-lactate, α-d-Glucose, Glycerate-2P, sucrose, Myo-Inositol, d-Mannose, α-d-Galactose1-phosphate, Galactinol and Stachyose. CPT also regulated DEPs relevant for histidine metabolism such as histidine ammonia lyase (Hal), carnosine dipeptidase 2 (Cndp2) and Aldh3a2, and regulated DEMs such as l-Glutamate, l-Aspartate, l-Histidine, Histamine and Urocanic acid.

## Discussion

In the present study, through the integration of proteomics and metabolomics, the mechanisms of CPT treatment in a rat acne model induced by oleic acid were examined. Differentially expressed proteins and metabolites were identified, and the mechanisms of CPT treatment in acne were illuminated. The hierarchical clustering figure showed that the skin proteins and metabolites in the MC group were significantly separated from those in the BC and CPT treatment groups, and that the CPT group was significantly closer to the BC group. The skin samples of each group were closely clustered in the PCA score plots. The MC group was significantly separated from both the BC and the CPT groups, and the skin samples in the BC group were closer to the CPT group. This indicated that the skin metabolic profiles were significantly changed in acne rats and that treatment with CPT led to a metabolic profile similar to that of BC rats. By the KEGG enrichment analysis, CPT rats had five pathways that played an important role in acne and were significantly altered for both their protein and metabolite contents. These pathways included galactose metabolism, glycolysis/gluconeogenesis, histidine metabolism, spinocerebellar ataxia and protein digestion and absorption. CPT regulated differential proteins (DEPs) such as Hk2, Eno1, Dld, Pfkp, Pgam1, Aldh3a2, Akr1b8, Hal and Cndp2, and the DEPs regulated differentially expressed metabolites (DEMs) such as Phosphoenolpyruvate, dl-lactate, Dihydroxyacetone phosphate, dl-2-Phosphoglycerate, sucrose, α-d-Glucose, Myo-Inositol, d-Mannose, Galactinol, Stachyose, α-d-Galactose1-phosphate, l-Glutamate, l-Aspartate, l-Histidine, Histamine and Urocanic acid. In this study, changes in upstream proteins could lead to changes in downstream metabolites, such as Eno1 regulating PEP, Pgam1 regulating dl-2-Phosphoglycerate (Glycerate-2P), Glycerate-2P regulating Eno1, Hal regulating Urocanic acid and Cndp2 regulating l-Histidine. In parallel, changes in upstream metabolites might lead to changes in downstream proteins, such as α-d-Glucose regulating Hk2 and l-Histidine regulating Hal. These proteins were significantly up-regulated in acne rat induced by oleic acid, however they were down-regulated after CPT treatment. Our data suggested that CPT could inhibit the disordered metabolism of acne rats, especially within the glycolysis/gluconeogenesis, galactose and histidine metabolisms, restoring these pathways back to normal.

A previous study reported that regulation of glycolysis might control keratinocyte differentiation by lowering the expression of Eno1. Indeed, Eno1 has a high expression in keratinocytes with accelerated dysfunction of tight junction, which reduces the integrity of the cellular barrier (Tohgasaki et al., 2018; Sutter et al., 2019). Glycolysis is a key metabolic pathway that provides energy for cellular activities and consumes equivalents to sustain cell division and cell proliferation (Sutter et al., 2019). Decreased glycolysis is related to cell differentiation, particularly in keratinocytes (Li et al., 2020). This is in agreement with our finding that the DEPs of glycolysis including Eno1, Hk2, Dld, Pfkp, Pgam1 and Aldh3a2 were significantly increased in MC rats, while these were significantly decreased after CPT treatment. Indeed, CPT rats had a negatively regulated glycolysis/gluconeogenesis pathway, decreased keratinocyte differentiation and improved keratinization and cellular barrier function (Figure 1, Figure 2A and Figure 11).

Through transcriptomics, Eckhart, et al. showed that Hal was significantly up-regulated during keratinocyte differentiation (Eckhart et al., 2008). This is consistent with our study that Hal was up-regulated in MC rats, leading to excessive keratinocyte differentiation. This phenomenon was improved after CPT treatment. Aldehydal dehydrogenase is produced by Aldh3a2, which improves fatty decomposition. A previous study reported that if the function of Aldh3a2 is disrupted, it may cause excessive accumulation of intracellular fat. This process affects both the physiological function of the cell protective membrane and the nutrients necessary to maintain the physiological function of the body (Kumar et al., 2020). The DEPs of histidine metabolism such as Hal, Aldh3a2 and Cndp2 were over-expressed in the MC group, while they were reduced in the CPT group. In our study, histidine metabolism was negative regulated by CPT in acne rats.

Rats with acne induced by oleic acid had significantly up-regulated pathways, such as IL-17 signaling pathway, glycolysis/gluconeogenesis, galactose metabolism, gap junction, histidine metabolism, spinocerebellar ataxia, protein digestion and absorption, estrogen signaling pathway, biosynthesis of unsaturated fatty acids metabolism, glycerophospholipid metabolism and linoleic acid metabolism. The results of the KEGG enrichment showed that DEMs were mainly related to glycolysis metabolism, histidine metabolism, biosynthesis of unsaturated fatty acids, linoleic acid metabolism and glycerophospholipid metabolism (Figure 5 and Figure 8). The metabolites S100 calcium binding protein A8 (S100a8), S100 calcium binding protein A9 (S100a9), heat shock protein 90 alpha family class B member 1 (Hsp90ab1), heat shock protein 90 beta family member 1 (Hsp90b1) and serine and arginine rich splicing factor 1(Srsf1) of the IL-17 signaling pathway were significantly up-regulated in the MC group, while these were significantly down-regulated after CPT treatment (Figure 3). CPT was shown to down-regulate IL-17 signaling pathway and down-regulate acne-driven immune activation of sebum cells (Oules et al., 2020). The metabolite Gpd1 of the glycerophospholipid metabolism was significantly decreased in the model group, and significantly increased after CPT treatment (Figure 3). Gpd1 regulated DEMs such as glycerol 3-phosphate and dihydroxyacetone phosphate (Figure 7A), which were significantly down-regulated in the MC group and up-regulated in the CPT group. The glycerophospholipid metabolism pathway was affected by CPT treatment. The DEPs hydroxysteroid (17-beta) dehydrogenase 12 (Hsd17b12), sterol carrier protein 2 (Scp2) and hydroxysteroid (17-beta) dehydrogenase 4(Hsd17b4) of the biosynthesis of unsaturated fatty acids metabolism pathway were significantly up-regulated by oleic acid; however these were down-regulated by CPT treatment. The DEMs arachidonic acid (AA), oleic acid, stearic acid, all *cis*-(6,9,12)-linolenic acid, alpha-linolenic acid (ALA), eicosapentaenoic acid (EPA), erucic acid and nervonic acid were part of the unsaturated fatty acids metabolism pathway and were significantly up-regulated in the MC group. CPT treatment down-regulated these metabolites. The DEMs of the unsaturated fatty acids metabolism, namely palmitic acid, linoleic acid (LA), docosahexaenoic acid (DHA) and tetracosanoic acid were significantly down-regulated in the MC group, whereas they were significantly up-regulated after CPT treatment. Hsd17b12 was regulated by the DEMs ALA, EPA, all *cis*-(6,9,12)-Linolenic acid, LA, AA, palmitic acid, stearic acid, tetracosanoic acid, oleic acid, erucic acid and nervonic acid. In parallel, DHA was regulated by Scp2 and Hsd17b4. The DEMs of the linoleic acid metabolism pathway, namely AA, 1-Stearoyl-2-oleoyl-sn-glycerol 3-phosphocholine (SOPC) and all *cis*-(6,9,12)-Linolenic acid were significantly up-regulated in the MC group, whereas CPT treatment led to a down-regulation of these metabolites.

Linoleic acid (LA) is an essential unsaturated fatty acid that plays a crucial role in inflammation (Burns et al., 2018). Research has shown that down-regulation of LA led to disordered LA metabolism on inflammatory rats (Liu et al., 2020). Indeed, LA was down-regulated in acne rats, suggesting oleic acid might disrupt LA metabolism, causing inflammation. LA was up-regulated in the CPT treatment group. This is in accord with our histopathological analysis showing that acne rats turned to back normal after CPT treatment. The biosynthesis of unsaturated fatty acids metabolism, glycerophospholipid metabolism and linoleic acid metabolism pathway could significantly alter sebum production and control sebaceous gland secretion after CPT treatment (Clayton et al., 2019). The effective epidermal physical barrier primarily in the stratum corneum (SC) requires a structural and functional combination of adherent junctions, tight junctions, gap junctions (GJ) and desmosomes (Cohen-Barak et al., 2020). The gap junction network of the *epidermis* contributes to keeping the integrity and homeostasis of this layer (Martin et al., 2014). In this study, the gap junctions were disturbed in the MC group, while CPT treatment turned the skin barrier back to normal.

Ces1d (Carboxylesterase 1D) is a glycoprotein involved in lipid metabolism and catalyzes the hydrolysis of triglycerides and monoglycerides (Zheng et al., 2020). cytochrome c oxidase subunit 5B(Cox5b) affects mitochondrial activity and also is involved in lipid synthesis (Soro-Arnaiz et al., 2016). Compared to the BC group, Ces1d in the MC group was significantly down-regulated, while Cox5b was up-regulated. After CPT treatment, Ces1d significantly increased, while Cox5b significantly decreased, when compared to the MC group. Our analysis suggested that CPT could improve the metabolism and biosynthesis of lipids, and mitochondrial function in acne rats. These results are in agreement with our team’s previous study (Ruan et al., 2020).

Keratin 16 (Krt16) and Keratin 17 (Krt17) are the type I intermediate filament proteins, which are primarily presented in the base cells of hair follicles and sebaceous glands of the epithelium. Krt17 can regulate various biological processes of skin cells, such as cell growth, cell proliferation, inflammation and hair follicle circulation, as well as immune responses (Jin and Wang, 2014; Yang et al., 2019). Krt16 has a close relationship with genes that participate in the maintenance of the skin barrier and natural immunity (Lessard et al., 2013). Keratin 14 (Krt14) is a major structural component of keratinocytes in the epidermal base, affecting biological processes such as cell mechanics, cell homeostasis, and epidermal barrier function (Guo et al., 2020). Krt 14, Krt 16 and Krt 17 were significantly up-regulated in the MC group and down-regulated after CPT treatment (Figure 3). These results and the histopathological and lesions analysis agree with previous research that reported that the expression of Krt 14, Krt 16 and Krt 17 were up-regulated in acne rats and could promote keratinocyte proliferation leading to excessive keratinization (Yang et al., 2017).

Tubulin alpha-4A chain (Tuba4a) and Moesin (Msn) were found to be DEPs in MC and CPT groups. Msn is regulated by tight junction assembly in the tight junction barrier, while Tuba4a was found to be significantly up-regulated in acne rat. CPT treatment decreased Tuba4a levels, indicating that the tight junction barrier was restored after CPT treatment. Previous studies have shown that Msn can become activated and induce the infiltration of inflammatory cells in tissues (Liu et al., 2015). We found that Msn in our model group was notably up-regulated. After CPT treatment, Msn was decreased when compared to the levels found for the MC group. The results of the histopathological analysis showed that there was inflammatory cell infiltration in the skin tissue of acne rats, however this phenomenon was improved after treatment with CPT. These results are consistent with a previous study of our group (Ruan et al., 2020).

The biological process of leukocyte transendothelial migration is related to the estrogen signaling pathway. Studies have shown that estrogen treatment could attenuate leukocyte infiltration in rat (Schneider et al., 2012). Krt 14, Krt 16, Krt 17, Hsp90b1 and Hsp90ab1 are proteins of the estrogen signaling pathway that were significantly up-regulated in the MC group and decreased in the CPT group (Figure 5). As shown in the cytokine levels in serum of IL-6, IL-8, TNF-α and IL-1β, the CPT could inhibit the release of these cytokines, thus improving inflammation in acne rats (Figure 2B). This indicated that the skin inflammation was controlled after CPT treatment. Further exploration is needed in the future.

## Conclusion

Through proteomics and metabolomics studies, we showed that CPT may regulate multiple biological processes to improve acne in rats. CPT could inhibit the disordered metabolism of several pathways in rats with acne, especially glycolysis/gluconeogenesis, galactose metabolism and histidine metabolism, which play an important role in acne development. These results can aid to explain the mechanism of action of CPT to treat acne. In conclusion, CPT might be a safe and potential drug to treat acne.

## Data Availability

The datasets presented in this study can be found in online repositories. The names of the repository/repositories and accession number(s) can be found below: PXD027219 *via* ProteomeXchange.

## References

[B1] Ashrafizadeh M. Zarrabi A. Orouei S. Saberifar S. Salami S. Hushmandi K. (2021). Recent Advances and Future Directions in Anti-tumor Activity of Cryptotanshinone: A Mechanistic Review. Phytother Res. 35, 155–179. 10.1002/ptr.6815 33507609

[B2] Bernardini N. Skroza N. Tolino E. Mambrin A. Anzalone A. Balduzzi V. (2020). IL-17 and its Role in Inflammatory, Autoimmune, and Oncological Skin Diseases: State of Art. Int. J. Dermatol. 59, 406–411. 10.1111/ijd.14695 31663126 PMC7216999

[B3] Burns J. L. Nakamura M. T. Ma D. W. L. (2018). Differentiating the Biological Effects of Linoleic Acid from Arachidonic Acid in Health and Disease. Prostaglandins Leukot. Essent. Fatty Acids 135, 1–4. 10.1016/j.plefa.2018.05.004 30103919

[B4] Chen C. Hou J. Tanner J. J. Cheng J. (2020). Bioinformatics Methods for Mass Spectrometry-Based Proteomics Data Analysis. Int. J. Mol. Sci. 21, 25. 10.3390/ijms21082873 PMC7216093 32326049

[B5] Clayton R. W. Göbel K. Niessen C. M. Paus R. Van Steensel M. A. M. Lim X. (2019). Homeostasis of the Sebaceous Gland and Mechanisms of Acne Pathogenesis. Br. J. Dermatol. 181, 677–690. 10.1111/bjd.17981 31056753

[B6] Cohen-Barak E. Godsel L. M. Koetsier J. L. Hegazy M. Kushnir-Grinbaum D. Hammad H. (2020). The Role of Desmoglein 1 in Gap Junction Turnover Revealed through the Study of SAM Syndrome. J. Invest. Dermatol. 140 **,** 556, e9-+. 10.1016/j.jid.2019.08.433 31465738 PMC7039747

[B7] Eckhart L. Schmidt M. Mildner M. Mlitz V. Abtin A. Ballaun C. (2008). Histidase Expression in Human Epidermal Keratinocytes: Regulation by Differentiation Status and All-Trans Retinoic Acid. J. Dermatol. Sci. 50, 209–215. 10.1016/j.jdermsci.2007.12.009 18280705

[B8] Gao Y. Li J. T. Li X. Li X. Yang S. W. Chen N. H. (2021). Tetrahydroxy Stilbene Glycoside Attenuates Acetaminophen-Induced Hepatotoxicity by UHPLC-Q-TOF/MS-based Metabolomics and Multivariate Data Analysis. J. Cel Physiol 236, 3832–3862. 10.1002/jcp.30127 33111343

[B9] Gertsman I. Barshop B. A. (2018). Promises and Pitfalls of Untargeted Metabolomics. J. Inherit. Metab. Dis. 41, 355–366. 10.1007/s10545-017-0130-7 29536203 PMC5960440

[B10] Gollnick H. P. Dreno B. (2015). Pathophysiology and Management of Acne. J. Eur. Acad. Dermatol. Venereol. 29 Suppl 4, 1–2. 10.1111/jdv.13182 26059727

[B11] Gui S. W. Liu Y. Y. Zhong X. G. Liu X. Zheng P. Pu J. C. (2018). Plasma Disturbance of Phospholipid Metabolism in Major Depressive Disorder by Integration of Proteomics and Metabolomics. Neuropsychiatr. Dis. Treat. 14, 1451–1461. 10.2147/ndt.s164134 29922061 PMC5995410

[B12] Guo Y. Redmond C. J. Leacock K. A. Brovkina M. V. Ji S. Jaskula-Ranga V. (2020). Keratin 14-dependent Disulfides Regulate Epidermal Homeostasis and Barrier Function *via* 14-3-3σ and YAP1. Elife 9, e53165. 10.7554/eLife.53165 32369015 PMC7250575

[B13] Han R. Blencke H. M. Cheng H. Li C. (2018). The Antimicrobial Effect of CEN1HC-Br against Propionibacterium Acnes and its Therapeutic and Anti-inflammatory Effects on Acne Vulgaris. Peptides 99, 36–43. 10.1016/j.peptides.2017.11.001 29108811

[B14] Harper J. C. (2020). Acne Vulgaris: What's New in Our 40th Year. J. Am. Acad. Dermatol. 82, 526–527. 10.1016/j.jaad.2019.01.092 31859048

[B15] Jin L. Wang G. (2014). Keratin 17: A Critical Player in the Pathogenesis of Psoriasis. Med. Res. Rev. 34, 438–454. 10.1002/med.21291 23722817

[B16] Kang D. Shi B. Erfe M. C. Craft N. Li H. (2015). Vitamin B12 Modulates the Transcriptome of the Skin Microbiota in Acne Pathogenesis. Sci. Transl Med. 7, 293ra103. 10.1126/scitranslmed.aab2009 PMC6049814 26109103

[B17] Lessard J. C. Piña-Paz S. Rotty J. D. Hickerson R. P. Kaspar R. L. Balmain A. (2013). Keratin 16 Regulates Innate Immunity in Response to Epidermal Barrier Breach. Proc. Natl. Acad. Sci. U S A. 110, 19537–19542. 10.1073/pnas.1309576110 24218583 PMC3845144

[B18] Li H. Gao C. Liu C. Liu L. Zhuang J. Yang J. (2021a). A Review of the Biological Activity and Pharmacology of Cryptotanshinone, an Important Active Constituent in Danshen. Biomed. Pharmacother. 137, 111332. 10.1016/j.biopha.2021.111332 33548911

[B19] Li J. Xing J. Lu F. Chang W. Liang N. Li J. (2020). Psoriatic Dermal-Derived Mesenchymal Stem Cells Reduce Keratinocyte Junctions, and Increase Glycolysis. Acta Derm Venereol. 100, adv00122. 10.2340/00015555-3480 32266413 PMC9129003

[B20] Li W. H. Fassih A. Binner C. Parsa R. Southall M. D. (2018). Low-level Red LED Light Inhibits Hyperkeratinization and Inflammation Induced by Unsaturated Fatty Acid in an *In Vitro* Model Mimicking Acne. Lasers Surg. Med. 50, 158–165. 10.1002/lsm.22747 29095531

[B21] Li Z. Xia J. Jiang L. Tan Y. An Y. Zhu X. (2021b). Characterization of the Human Skin Resistome and Identification of Two Microbiota Cutotypes. Microbiome 9, 47. 10.1186/s40168-020-00995-7 33597039 PMC7890624

[B22] Liu X. Yang T. Suzuki K. Tsukita S. Ishii M. Zhou S. (2015). Moesin and Myosin Phosphatase Confine Neutrophil Orientation in a Chemotactic Gradient. J. Exp. Med. 212, 267–280. 10.1084/jem.20140508 25601651 PMC4322047

[B23] Liu Y. J. Li H. Tian Y. Han J. Wang X. Y. Li X. Y. (2020). PCTR1 Ameliorates Lipopolysaccharide-Induced Acute Inflammation and Multiple Organ Damage *via* Regulation of Linoleic Acid Metabolism by Promoting FADS1/FASDS2/ELOV2 Expression and Reducing PLA2 Expression. Lab. Invest. 100, 904–915. 10.1038/s41374-020-0412-9 32123295

[B24] Ma N. Yang Y. Liu X. Li S. Qin Z. Li J. (2020). Plasma Metabonomics and Proteomics Studies on the Anti-thrombosis Mechanism of Aspirin Eugenol Ester in Rat Tail Thrombosis Model. J. Proteomics 215, 103631. 10.1016/j.jprot.2019.103631 31891783

[B25] Maione F. Piccolo M. De Vita S. Chini M. G. Cristiano C. De Caro C. (2018). Down Regulation of Pro-inflammatory Pathways by Tanshinone IIA and Cryptotanshinone in a Non-genetic Mouse Model of Alzheimer's Disease. Pharmacol. Res. 129, 482–490. 10.1016/j.phrs.2017.11.018 29158049

[B26] Markovics A. Tóth K. F. Sós K. E. Magi J. Gyöngyösi A. Benyó Z. (2019). Nicotinic Acid Suppresses Sebaceous Lipogenesis of Human Sebocytes *via* Activating Hydroxycarboxylic Acid Receptor 2 (HCA2 ). J. Cel Mol Med 23, 6203–6214. 10.1111/jcmm.14505 PMC6714165 31273921

[B27] Martin P. E. Easton J. A. Hodgins M. B. Wright C. S. (2014). Connexins: Sensors of Epidermal Integrity that Are Therapeutic Targets. FEBS Lett. 588, 1304–1314. 10.1016/j.febslet.2014.02.048 24607543

[B28] Monti C. Zilocchi M. Colugnat I. Alberio T. (2019). Proteomics Turns Functional. J. Proteomics 198, 36–44. 10.1016/j.jprot.2018.12.012 30553948

[B29] Nagappan A. Kim J. H. Jung D. Y. Jung M. H. (2020). Cryptotanshinone from the Salvia Miltiorrhiza Bunge Attenuates Ethanol-Induced Liver Injury by Activation of AMPK/SIRT1 and Nrf2 Signaling Pathways. Int. J. Mol. Sci. 21, 19. 10.3390/ijms21010265 PMC6981483 31906014

[B30] Nesvizhskii A. I. (2014). Proteogenomics: Concepts, Applications and Computational Strategies. Nat. Methods 11, 1114–1125. 10.1038/nmeth.3144 25357241 PMC4392723

[B31] O'connell J. D. Paulo J. A. O'brien J. J. Gygi S. P. (2018). Proteome-Wide Evaluation of Two Common Protein Quantification Methods. J. Proteome Res. 17, 1934–1942. 10.1021/acs.jproteome.8b00001610.1021/acs.jproteome.8b00016 29635916 PMC5984592

[B32] O'neill A. M. Gallo R. L. (2018). Host-microbiome Interactions and Recent Progress into Understanding the Biology of Acne Vulgaris. Microbiome 6, 177. 10.1186/s40168-018-0558-5 30285861 PMC6169095

[B33] Oulès B. Philippeos C. Segal J. Tihy M. Vietri Rudan M. Cujba A. M. (2020). Contribution of GATA6 to Homeostasis of the Human Upper Pilosebaceous Unit and Acne Pathogenesis. Nat. Commun. 11, 5067. 10.1038/s41467-020-18784-z 33082341 PMC7575575

[B34] Patti G. J. Yanes O. Siuzdak G. (2012). Innovation: Metabolomics: the Apogee of the Omics Trilogy. Nat. Rev. Mol. Cel Biol 13, 263–269. 10.1038/nrm3314 PMC3682684 22436749

[B35] Quanico J. Gimeno J. P. Nadal-Wollbold F. Casas C. Alvarez-Georges S. Redoulès D. (2017). Proteomic and Transcriptomic Investigation of Acne Vulgaris Microcystic and Papular Lesions: Insights in the Understanding of its Pathophysiology. Biochim. Biophys. Acta Gen. Subj 1861, 652–663. 10.1016/j.bbagen.2016.10.021 27789243

[B36] Rahman N. Jeon M. Song H. Y. Kim Y. S. (2016). Cryptotanshinone, a Compound of Salvia Miltiorrhiza Inhibits Pre-adipocytes Differentiation by Regulation of Adipogenesis-Related Genes Expression *via* STAT3 Signaling. Phytomedicine 23, 58–67. 10.1016/j.phymed.2015.12.004 26902408

[B37] Ruan S. Xiang S. Wu W. Cao S. Du Q. Wang Z. (2020). Potential Role of mTORC1 and the PI3K-Akt Pathway in Anti-acne Properties of Licorice Flavonoids. J. Funct. Foods 70, 103968. 10.1016/j.jff.2020.103968

[B38] Saurat J. H. (2015). Strategic Targets in Acne: The Comedone Switch in Question. Dermatology 231, 105–111. 10.1159/000382031 26113292

[B39] Schneider B. S. Vigil S. A. Moonie S. (2012). Body Weight and Leukocyte Infiltration after an Acute Exercise-Related Muscle Injury in Ovariectomized Mice Treated with Estrogen and Progesterone. Gen. Comp. Endocrinol. 176, 144–150. 10.1016/j.ygcen.2011.12.019 22233774 PMC3319700

[B40] Soro-Arnaiz I. Li Q. O. Y. Torres-Capelli M. Meléndez-Rodríguez F. Veiga S. Veys K. (2016). Role of Mitochondrial Complex IV in Age-dependent Obesity. Cell Rep 16, 2991–3002. 10.1016/j.celrep.2016.08.041 27626667

[B41] Sutter C. H. Olesen K. M. Bhuju J. Guo Z. Sutter T. R. (2019). AHR Regulates Metabolic Reprogramming to Promote SIRT1-dependent Keratinocyte Differentiation. J. Invest. Dermatol. 139, 818–826. 10.1016/j.jid.2018.10.019 30393078 PMC6431567

[B42] Tang L. He S. Wang X. Liu H. Zhu Y. Feng B. (2018). Cryptotanshinone Reduces Psoriatic Epidermal Hyperplasia *via* Inhibiting the Activation of STAT3. Exp. Dermatol. 27, 268–275. 10.1111/exd.13511 29427477

[B43] Tohgasaki T. Ozawa N. Yoshino T. Ishiwatari S. Matsukuma S. Yanagi S. (2018). Enolase-1 Expression in the Stratum Corneum Is Elevated with Parakeratosis of Atopic Dermatitis and Disrupts the Cellular Tight junction Barrier in Keratinocytes. Int. J. Cosmet. Sci. 40, 178–186. 10.1111/ics.12449 29430682

[B44] Tricarico P. M. Boniotto M. Genovese G. Zouboulis C. C. Marzano A. V. Crovella S. (2019). An Integrated Approach to Unravel Hidradenitis Suppurativa Etiopathogenesis. Front. Immunol. 10, 892. 10.3389/fimmu.2019.00892 31105704 PMC6494959

[B45] Udhaya Kumar S. Thirumal Kumar D. Mandal P. D. Sankar S. Haldar R. Kamaraj B. (2020). “Comprehensive In Silico Screening and Molecular Dynamics Studies of Missense Mutations in Sjogren-Larsson Syndrome Associated with the ALDH3A2 Gene,” in Inflammatory Disorders - Pt B. Editor Donev R. (San Diego: Elsevier Academic Press Inc)), 349–377. 10.1016/bs.apcsb.2019.11.004 32085885

[B46] Wang B. Wang Y. Zuo S. Peng S. Wang Z. Zhang Y. (2021). Untargeted and Targeted Metabolomics Profiling of Muscle Reveals Enhanced Meat Quality in Artificial Pasture Grazing Tan Lambs *via* Rescheduling the Rumen Bacterial Community. J. Agric. Food Chem. 69, 846–858. 10.1021/acs.jafc.0c06427 33405917

[B47] Wang Z. Liu L. Xiang S. Jiang C. Wu W. Ruan S. (2020). Formulation and Characterization of a 3D-Printed Cryptotanshinone-Loaded Niosomal Hydrogel for Topical Therapy of Acne. Aaps Pharmscitech 21, 159. 10.1208/s12249-020-01677-1 32476076

[B48] Williams H. C. Dellavalle R. P. Garner S. (2012). Acne Vulgaris. Lancet 379, 361–372. 10.1016/s0140-6736(11)60321-8 21880356

[B49] Wiśniewski J. R. Zougman A. Nagaraj N. Mann M. (2009). Universal Sample Preparation Method for Proteome Analysis. Nat. Methods 6, 359–362. 10.1038/nmeth.1322 19377485

[B50] Wörheide M. A. Krumsiek J. Kastenmüller G. Arnold M. (2021). Multi-omics Integration in Biomedical Research - A Metabolomics-Centric Review. Anal. Chim. Acta 1141, 144–162. 10.1016/j.aca.2020.10.038 33248648 PMC7701361

[B51] Worlein J. M. Baker K. Bloomsmith M. Coleman K. Koban T. L. (2011). Author Index. Altern. Lab. Anim. 39, 98–99. 10.1177/026119291103900111

[B52] Xu D. Lin T. H. Li S. Da J. Wen X. Q. Ding J. (2012). Cryptotanshinone Suppresses Androgen Receptor-Mediated Growth in Androgen Dependent and Castration Resistant Prostate Cancer Cells. Cancer Lett. 316, 11–22. 10.1016/j.canlet.2011.10.006 22154085 PMC3283034

[B53] Yan X. Jin J. Su X. Yin X. Gao J. Wang X. (2020). Intestinal Flora Modulates Blood Pressure by Regulating the Synthesis of Intestinal-Derived Corticosterone in High Salt-Induced Hypertension. Circ. Res. 126, 839–853. 10.1161/circresaha.119.316394 32078445

[B54] Yang L. Fan X. Cui T. Dang E. Wang G. (2017). Nrf2 Promotes Keratinocyte Proliferation in Psoriasis through Up-Regulation of Keratin 6, Keratin 16, and Keratin 17. J. Invest. Dermatol. 137, 2168–2176. 10.1016/j.jid.2017.05.015 28576737

[B55] Yang L. Zhang S. Wang G. (2019). Keratin 17 in Disease Pathogenesis: from Cancer to Dermatoses. J. Pathol. 247, 158–165. 10.1002/path.5178 30306595

[B56] Yi H. Yang G. Xiong Y. Wu Q. Xiao H. Wen X. (2019). Integrated Metabolomic and Proteomics Profiling Reveals the Promotion of Lactobacillus Reuteri LR1 on Amino Acid Metabolism in the Gut-Liver axis of Weaned Pigs. Food Funct. 10, 7387–7396. 10.1039/c9fo01781j 31651917

[B57] Yu Z. Lv H. Han G. Ma K. (2016). Ethosomes Loaded with Cryptotanshinone for Acne Treatment through Topical Gel Formulation. Plos One 11, e0159967. 10.1371/journal.pone.0159967 27441661 PMC4956045

[B58] Zhang Y. Lu W. Zhang X. Lu J. Xu S. Chen S. (2019). Cryptotanshinone Protects against Pulmonary Fibrosis through Inhibiting Smad and STAT3 Signaling Pathways. Pharmacol. Res. 147, 104307. 10.1016/j.phrs.2019.104307 31181334

[B59] Zheng X. Ren B. Li X. Yan H. Xie Q. Liu H. (2020). Selenoprotein F Knockout Leads to Glucose and Lipid Metabolism Disorders in Mice. J. Biol. Inorg. Chem. 25, 1009–1022. 10.1007/s00775-020-01821-z 32995962

[B60] Zuo T. Chen H. Xiang S. Hong J. Cao S. Weng L. (2016). Cryptotanshinone-Loaded Cerasomes Formulation: *In Vitro* Drug Release, *In Vivo* Pharmacokinetics, and *In Vivo* Efficacy for Topical Therapy of Acne. Acs Omega 1, 1326–1335. 10.1021/acsomega.6b00232 30023507 PMC6044685

