# Supplementary figures and images for: Integrated Proteomics and Metabolomics Link Acne to the Action Mechanisms of Cryptotanshinone Intervention (part 2 of 2)
Source: Front Pharmacol. 2021 Sep 1;12:700696. doi: 10.3389/fphar.2021.700696 (PMC8440807; doi:10.3389/fphar.2021.700696)

2-Oxoadipic acid

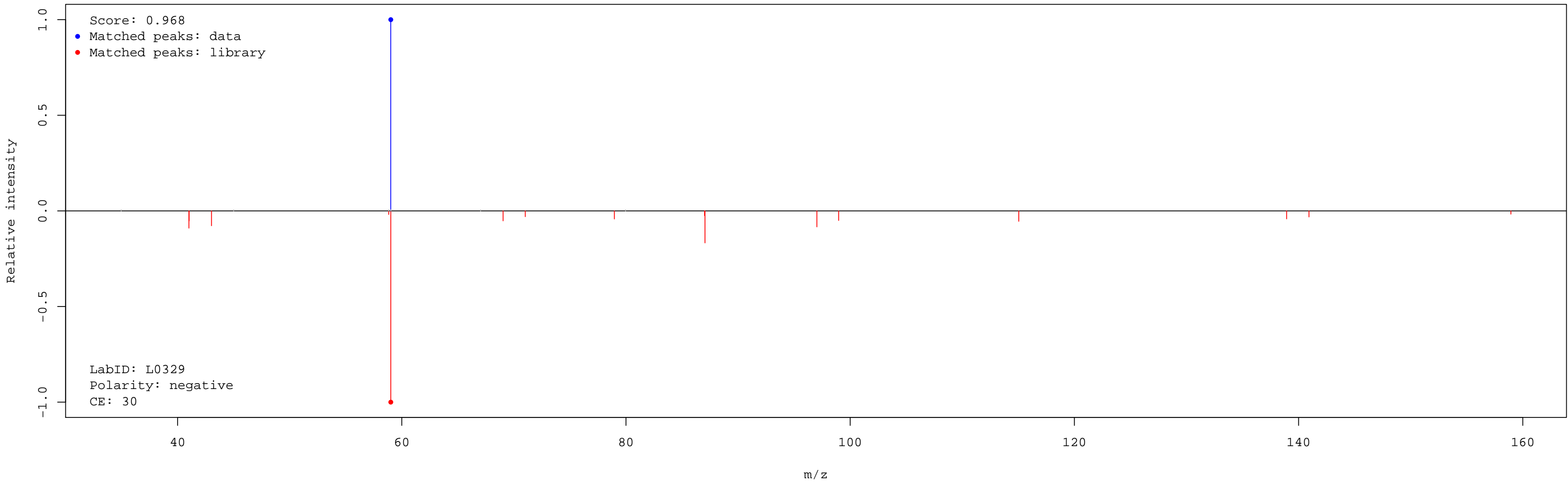

Supplement: Supplementary file 1 [file DataSheet1.ZIP › Supplementary table 1-10 and material 1-3/Material 3-Metlib-MSMS/NEG-Metlib-MSMS/Metlib-MSMS/M141T158_forward/0.968,2-Oxoadipic acid,(M-H2O-H)-.pdf]

2-Oxoadipic acid

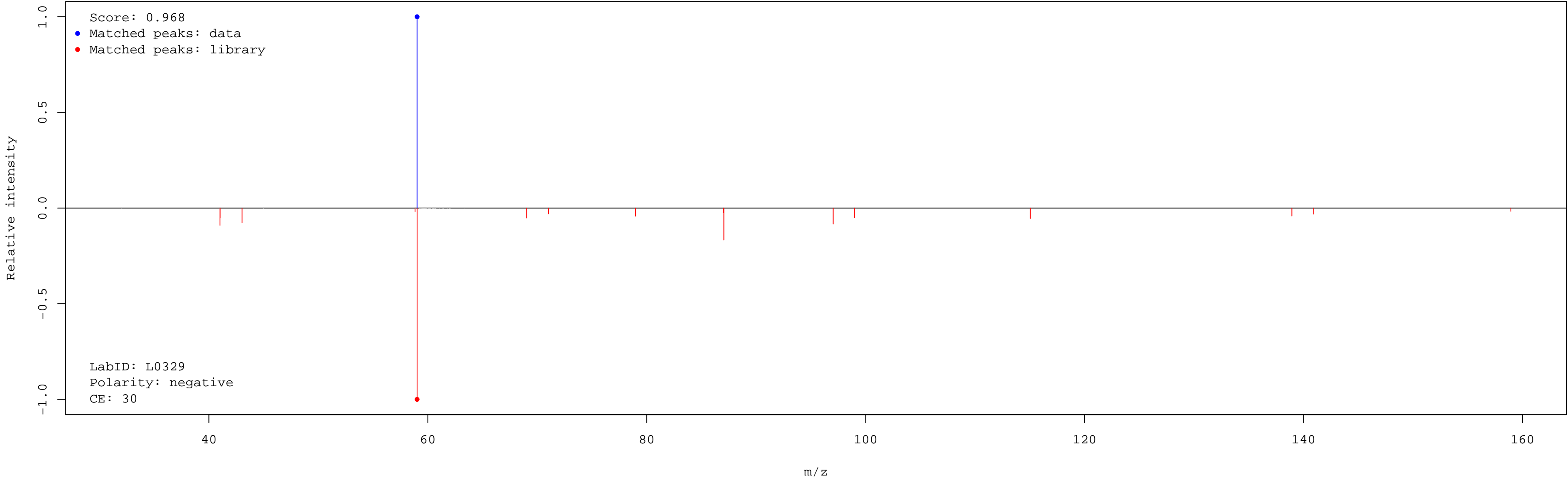

Supplement: Supplementary file 1 [file DataSheet1.ZIP › Supplementary table 1-10 and material 1-3/Material 3-Metlib-MSMS/NEG-Metlib-MSMS/Metlib-MSMS/M141T209_forward/0.968,2-Oxoadipic acid,(M-H2O-H)-.pdf]

2-Oxoadipic acid

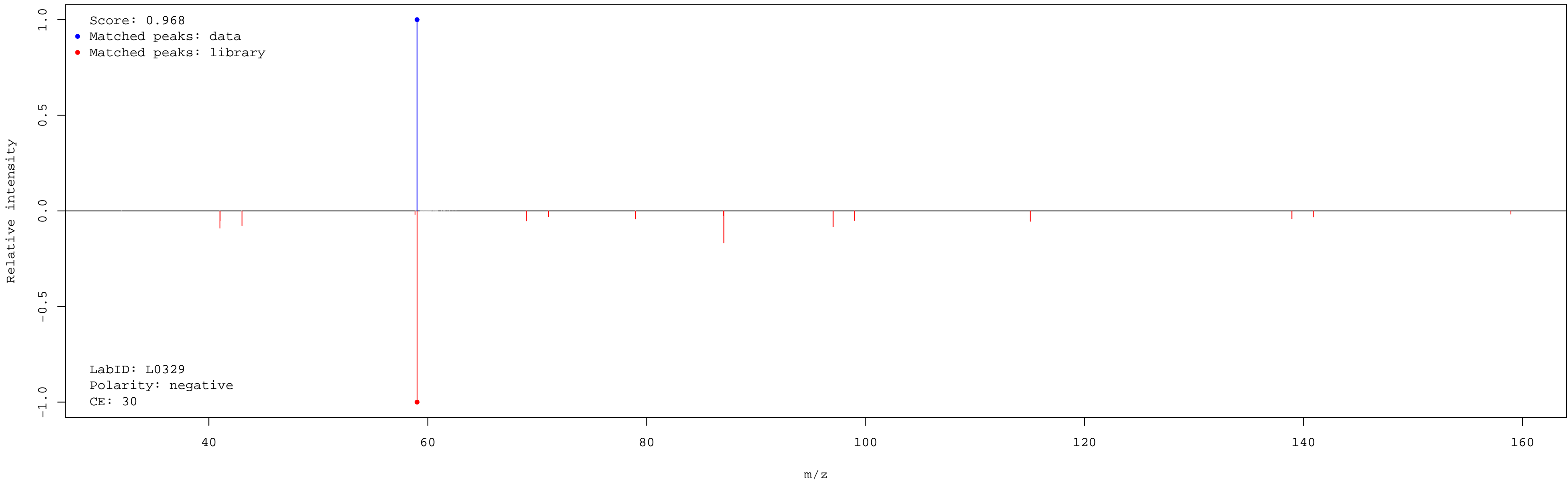

Supplement: Supplementary file 1 [file DataSheet1.ZIP › Supplementary table 1-10 and material 1-3/Material 3-Metlib-MSMS/NEG-Metlib-MSMS/Metlib-MSMS/M141T283_forward/0.968,2-Oxoadipic acid,(M-H2O-H)-.pdf]

2-Oxoadipic acid

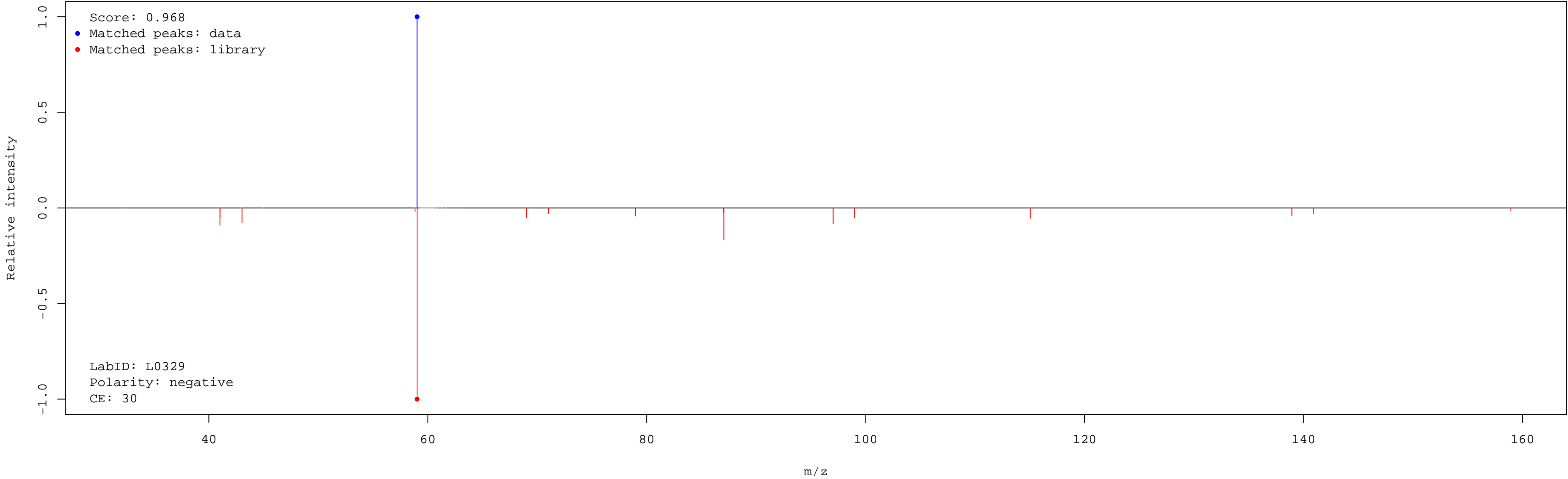

Supplement: Supplementary file 1 [file DataSheet1.ZIP › Supplementary table 1-10 and material 1-3/Material 3-Metlib-MSMS/NEG-Metlib-MSMS/Metlib-MSMS/M141T309_forward/0.968,2-Oxoadipic acid,(M-H2O-H)-.pdf]

2-Oxoadipic acid

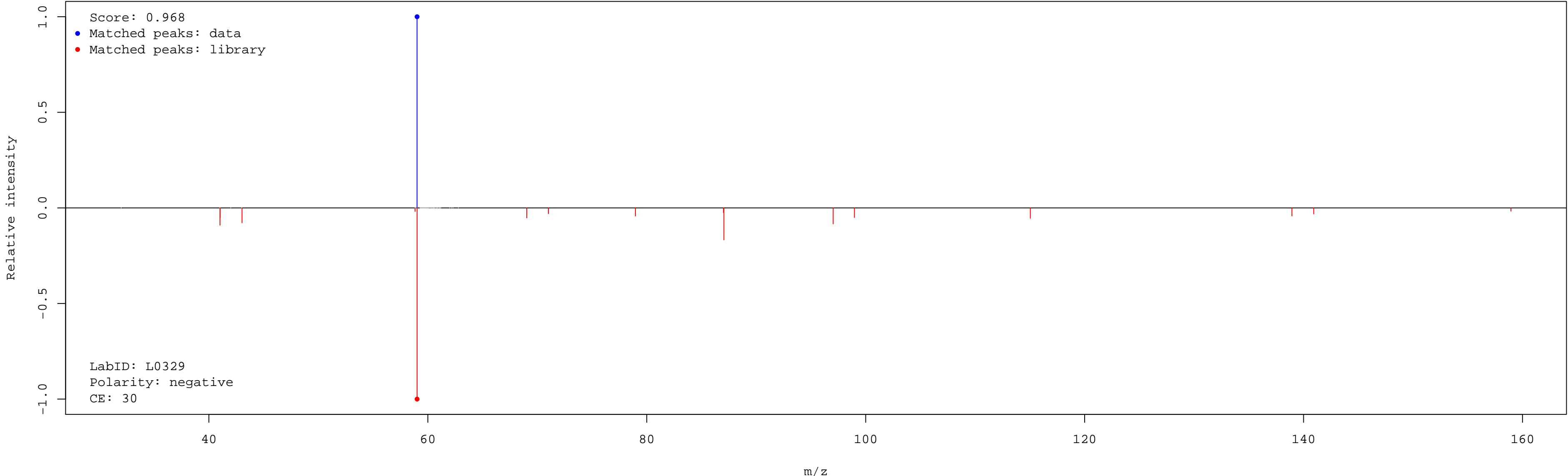

Supplement: Supplementary file 1 [file DataSheet1.ZIP › Supplementary table 1-10 and material 1-3/Material 3-Metlib-MSMS/NEG-Metlib-MSMS/Metlib-MSMS/M141T346_2_forward/0.968,2-Oxoadipic acid,(M-H2O-H)-.pdf]

# 4-Guanidinobutyric acid

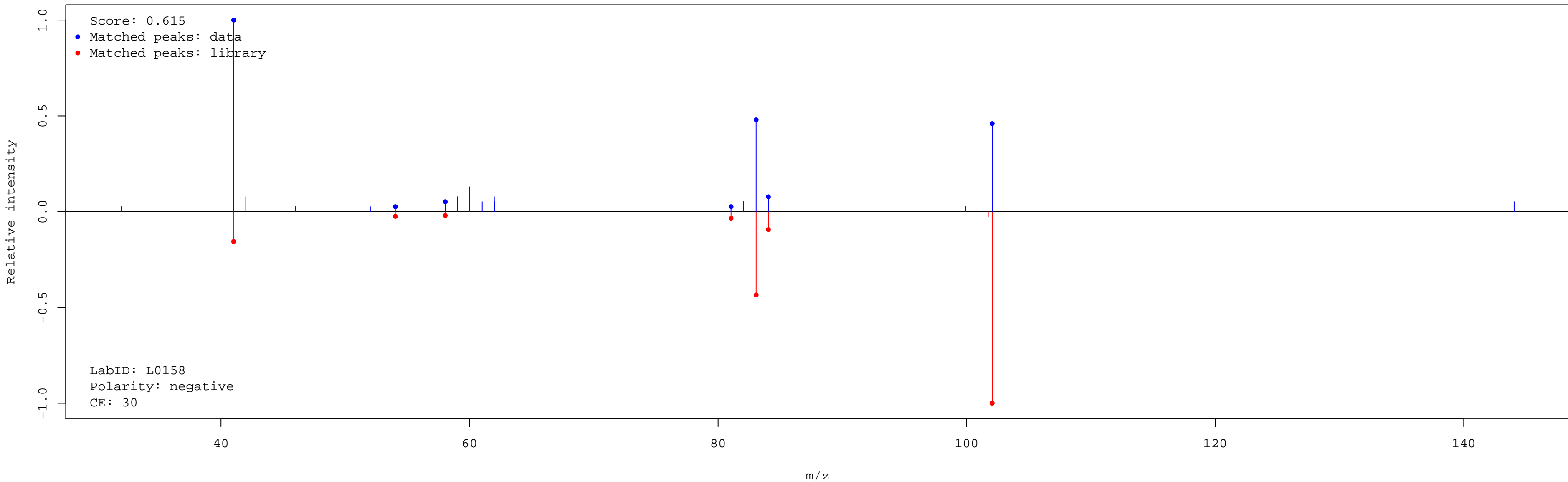

Supplement: Supplementary file 1 [file DataSheet1.ZIP › Supplementary table 1-10 and material 1-3/Material 3-Metlib-MSMS/NEG-Metlib-MSMS/Metlib-MSMS/M144T357_forward/0.615,4-Guanidinobutyric acid,(M-H)-.pdf]

# L-Glutamine

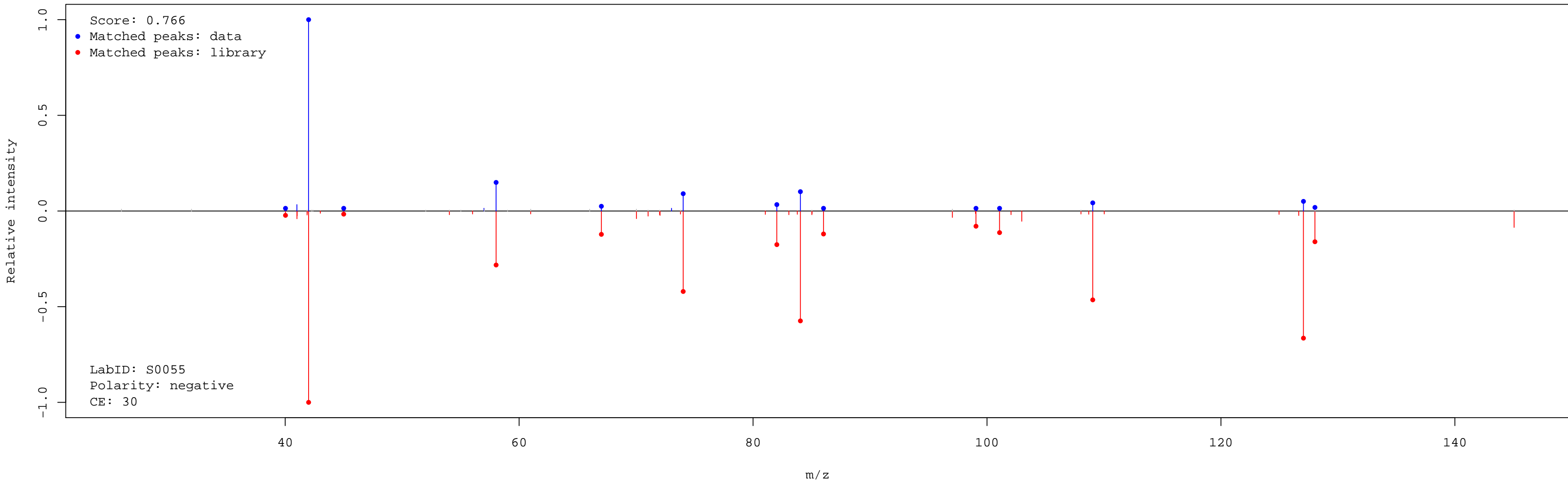

Supplement: Supplementary file 1 [file DataSheet1.ZIP › Supplementary table 1-10 and material 1-3/Material 3-Metlib-MSMS/NEG-Metlib-MSMS/Metlib-MSMS/M145T375_forward/0.766,L-Glutamine,(M-H)-.pdf]

# L-Glutamine

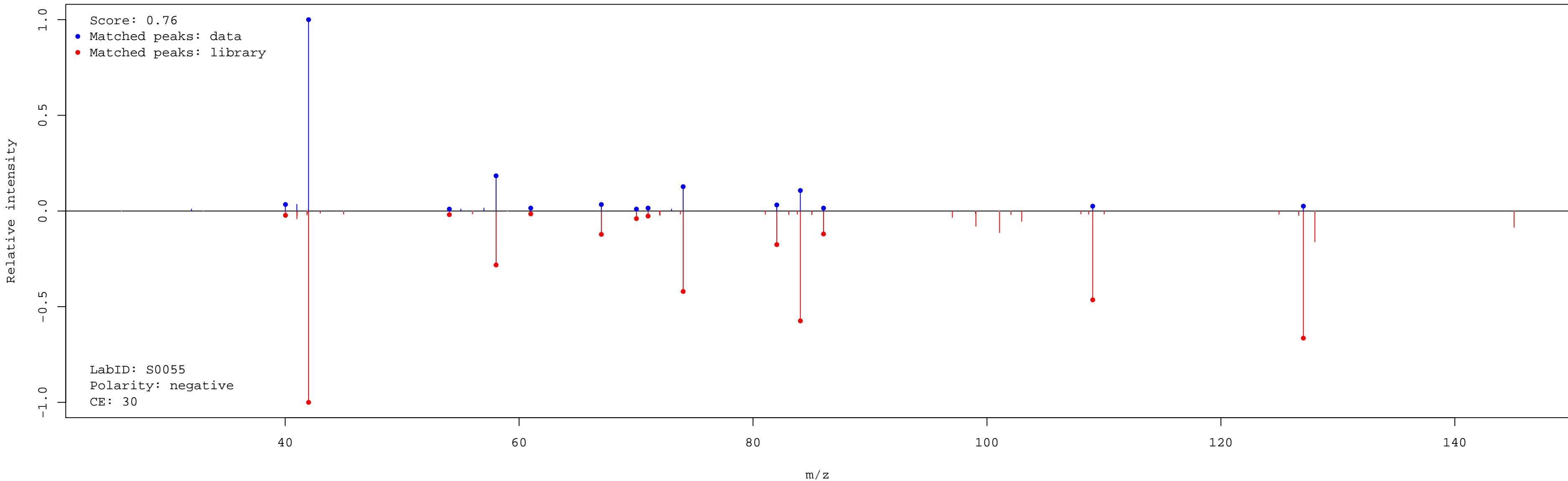

Supplement: Supplementary file 1 [file DataSheet1.ZIP › Supplementary table 1-10 and material 1-3/Material 3-Metlib-MSMS/NEG-Metlib-MSMS/Metlib-MSMS/M145T428_forward/0.76,L-Glutamine,(M-H)-.pdf]

# L-Glutamate

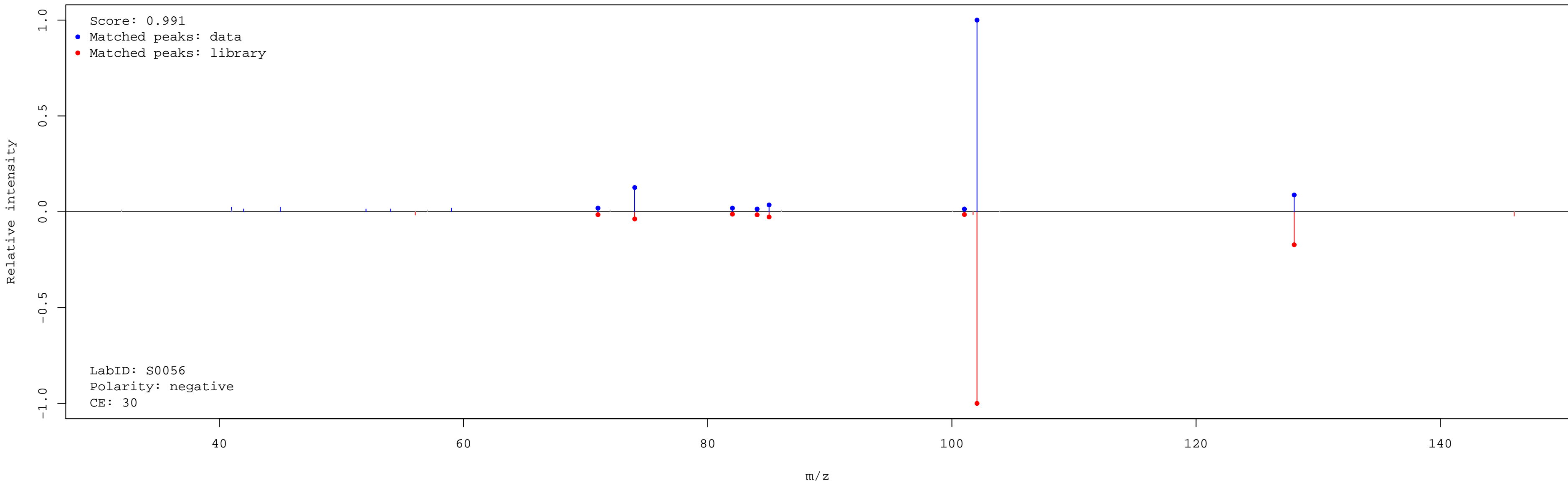

Supplement: Supplementary file 1 [file DataSheet1.ZIP › Supplementary table 1-10 and material 1-3/Material 3-Metlib-MSMS/NEG-Metlib-MSMS/Metlib-MSMS/M146T301_forward/0.991,L-Glutamate,(M-H)-.pdf]

# L-Glutamate

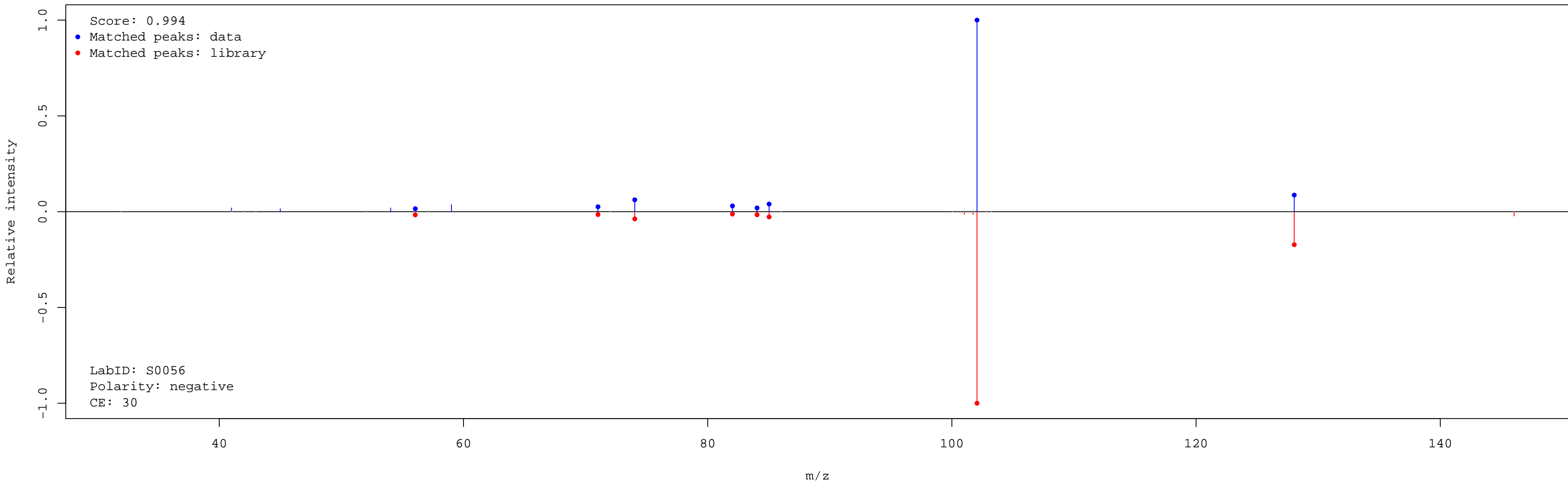

Supplement: Supplementary file 1 [file DataSheet1.ZIP › Supplementary table 1-10 and material 1-3/Material 3-Metlib-MSMS/NEG-Metlib-MSMS/Metlib-MSMS/M146T399_2_forward/0.994,L-Glutamate,(M-H)-.pdf]

# L-Glutamate

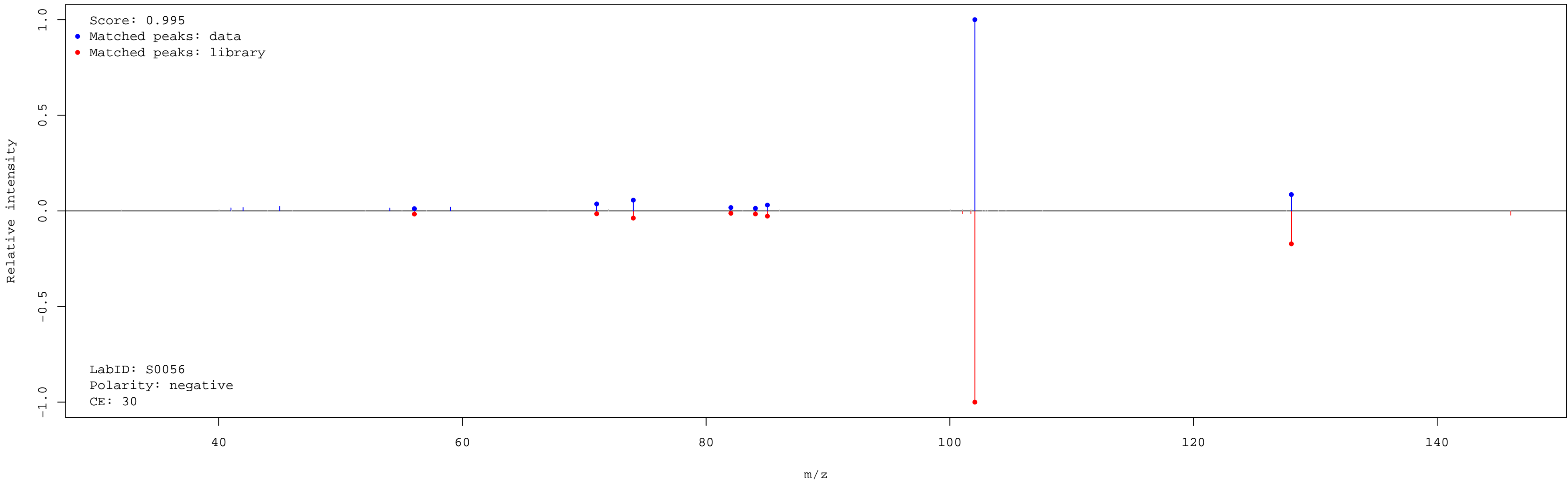

Supplement: Supplementary file 1 [file DataSheet1.ZIP › Supplementary table 1-10 and material 1-3/Material 3-Metlib-MSMS/NEG-Metlib-MSMS/Metlib-MSMS/M146T440_forward/0.995,L-Glutamate,(M-H)-.pdf]

# L-Glutamate

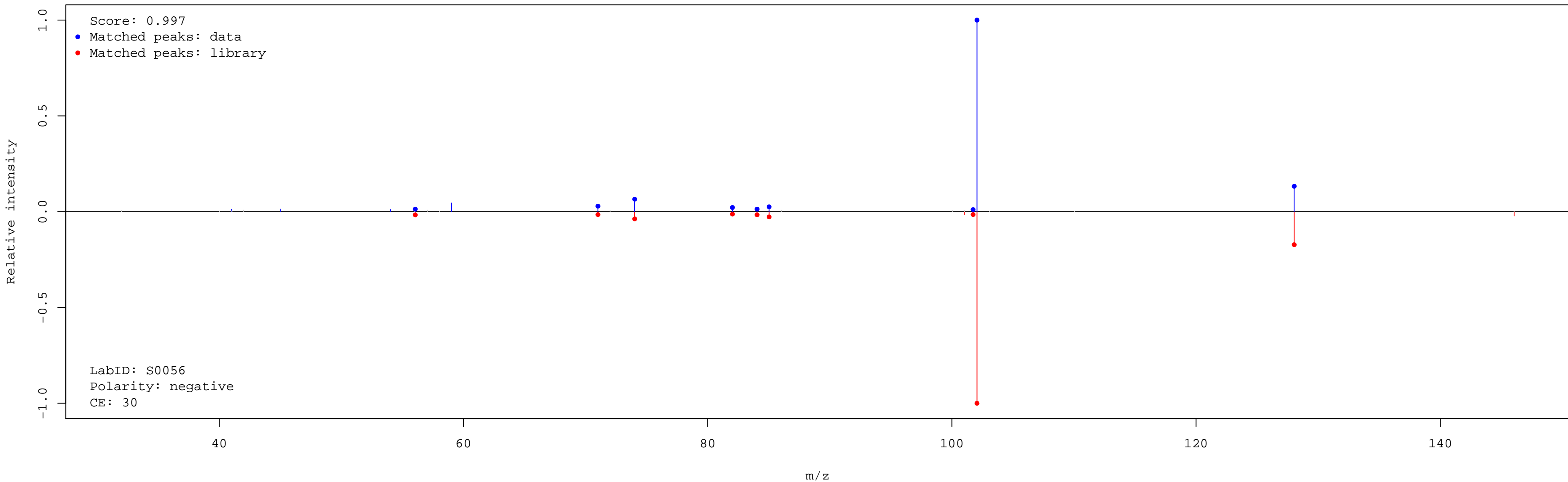

Supplement: Supplementary file 1 [file DataSheet1.ZIP › Supplementary table 1-10 and material 1-3/Material 3-Metlib-MSMS/NEG-Metlib-MSMS/Metlib-MSMS/M146T488_forward/0.997,L-Glutamate,(M-H)-.pdf]

# L-Glutamate

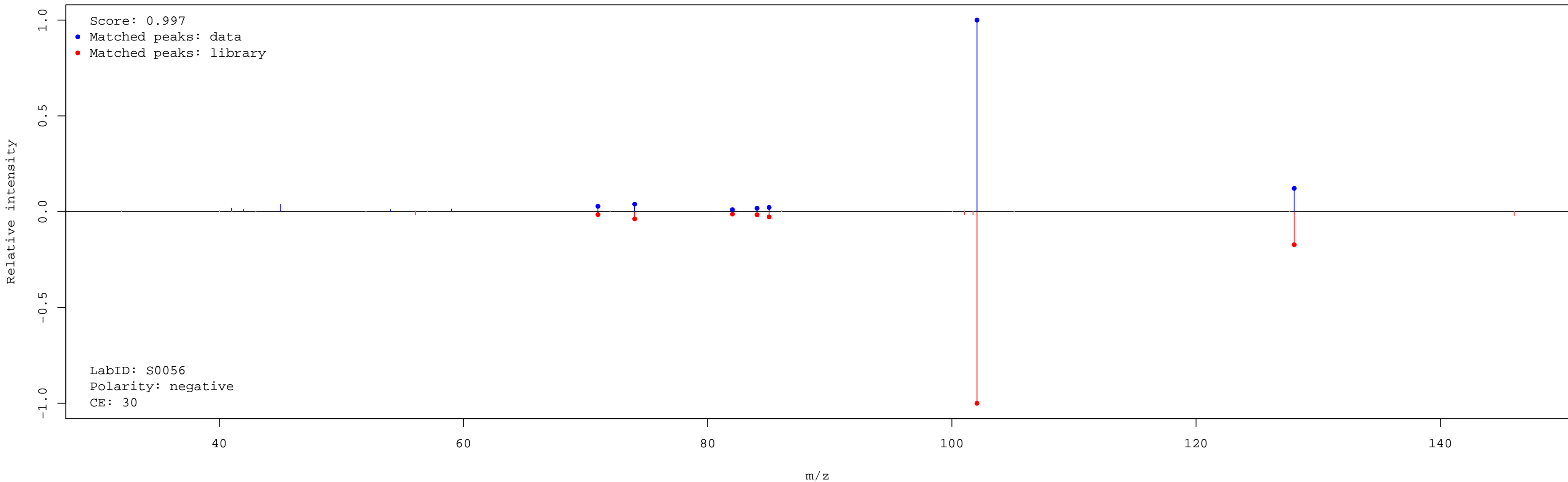

Supplement: Supplementary file 1 [file DataSheet1.ZIP › Supplementary table 1-10 and material 1-3/Material 3-Metlib-MSMS/NEG-Metlib-MSMS/Metlib-MSMS/M146T502_forward/0.997,L-Glutamate,(M-H)-.pdf]

Citramalic acid

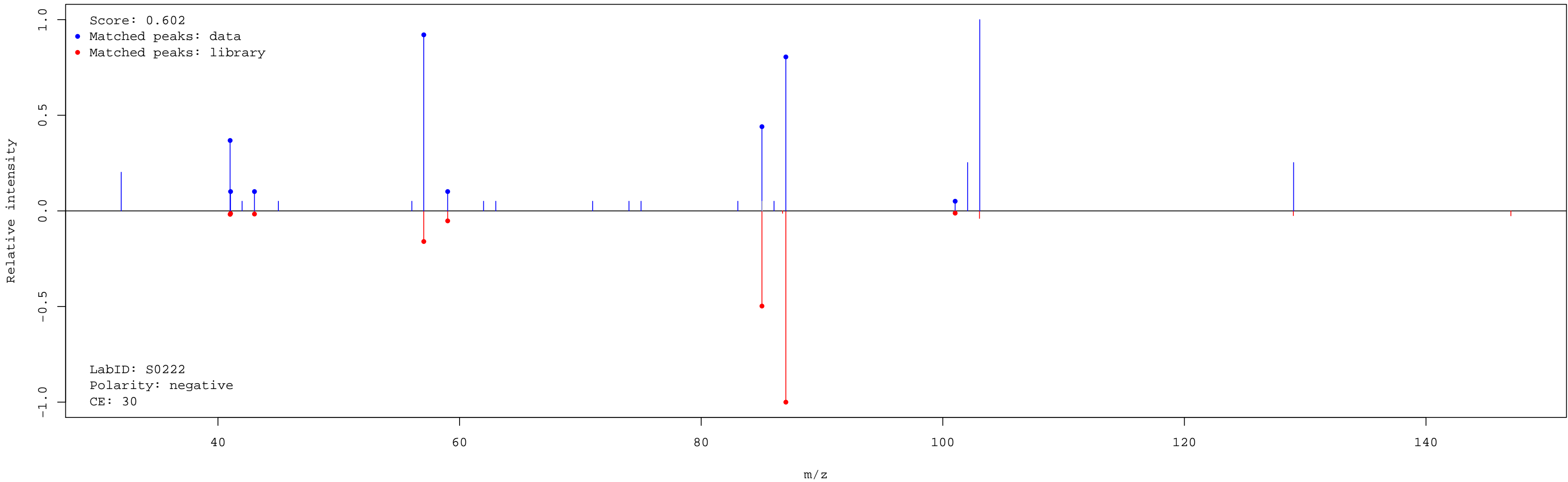

Supplement: Supplementary file 1 [file DataSheet1.ZIP › Supplementary table 1-10 and material 1-3/Material 3-Metlib-MSMS/NEG-Metlib-MSMS/Metlib-MSMS/M147T347_forward/0.602,Citramalic acid,(M-H)-.pdf]

N6-Methyladenine

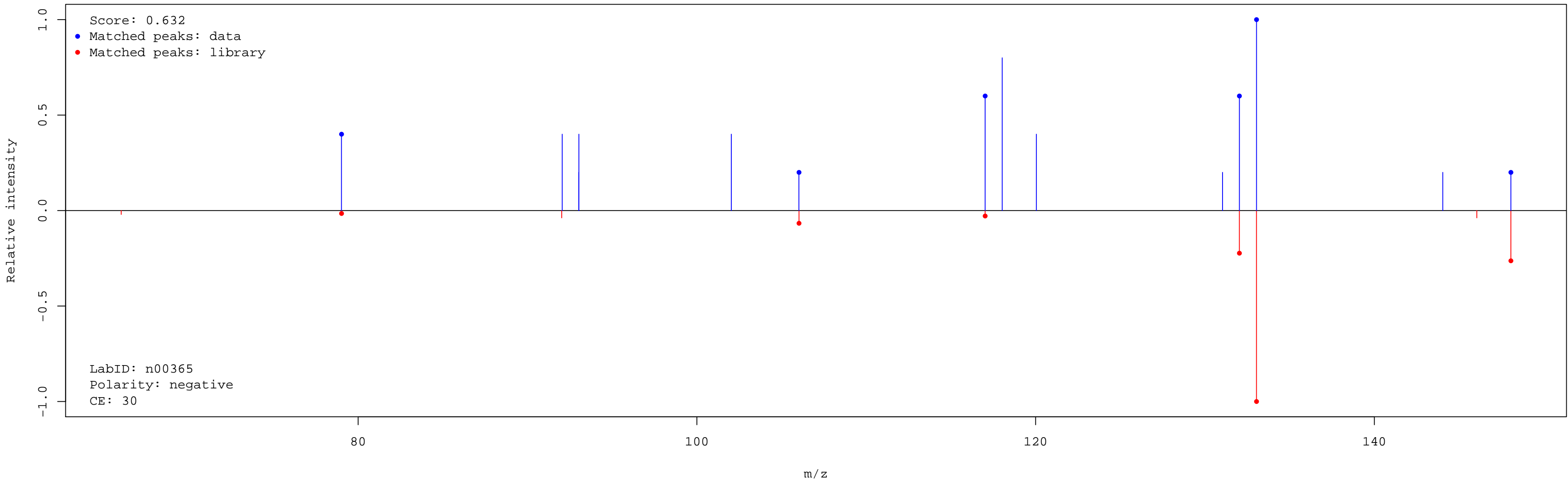

Supplement: Supplementary file 1 [file DataSheet1.ZIP › Supplementary table 1-10 and material 1-3/Material 3-Metlib-MSMS/NEG-Metlib-MSMS/Metlib-MSMS/M148T133_forward/0.632,N6-Methyladenine,(M-H)-.pdf]

L-Methionine

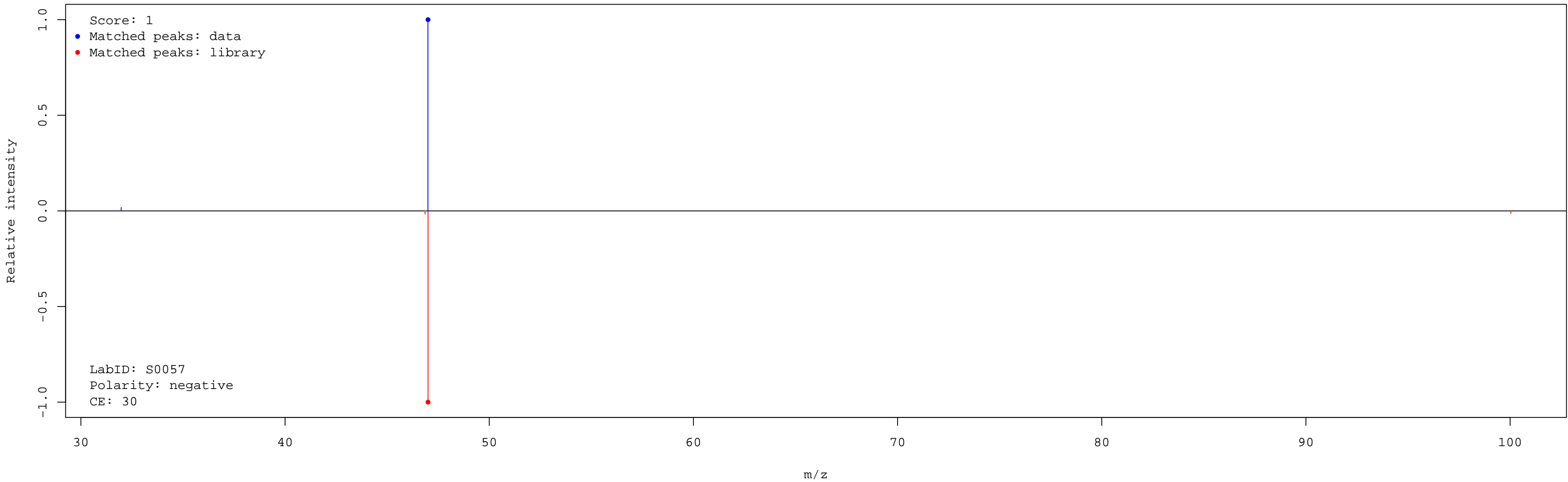

Supplement: Supplementary file 1 [file DataSheet1.ZIP › Supplementary table 1-10 and material 1-3/Material 3-Metlib-MSMS/NEG-Metlib-MSMS/Metlib-MSMS/M148T284_forward/1,L-Methionine,(M-H)-.pdf]

L-Methionine

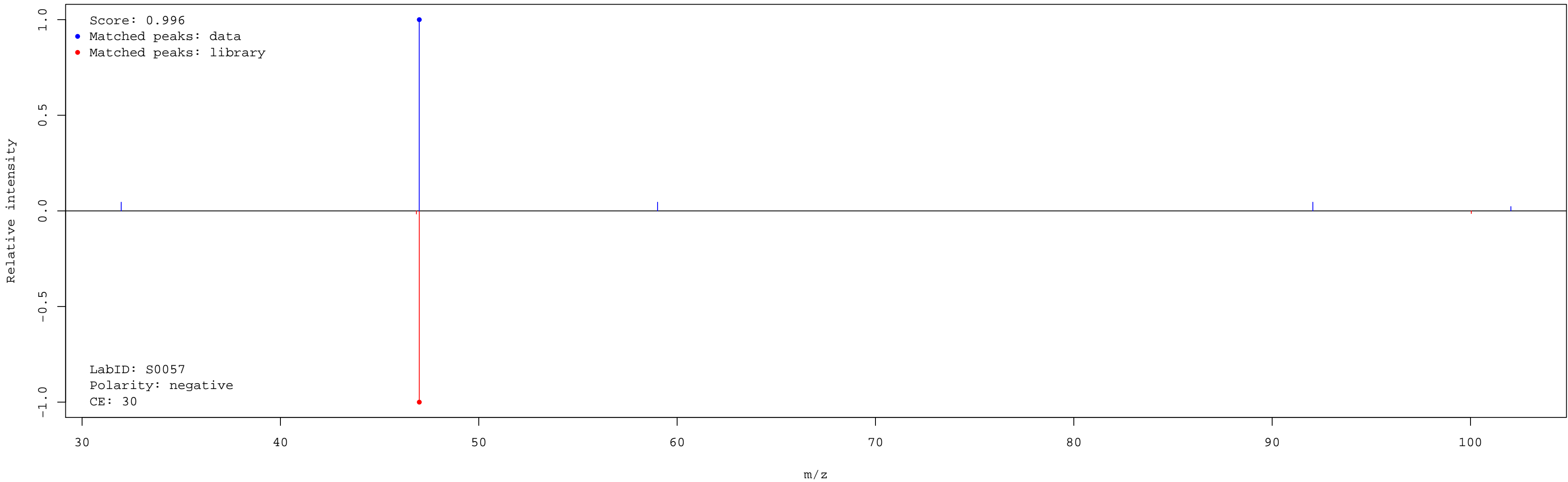

Supplement: Supplementary file 1 [file DataSheet1.ZIP › Supplementary table 1-10 and material 1-3/Material 3-Metlib-MSMS/NEG-Metlib-MSMS/Metlib-MSMS/M148T307_forward/0.996,L-Methionine,(M-H)-.pdf]

L-Methionine

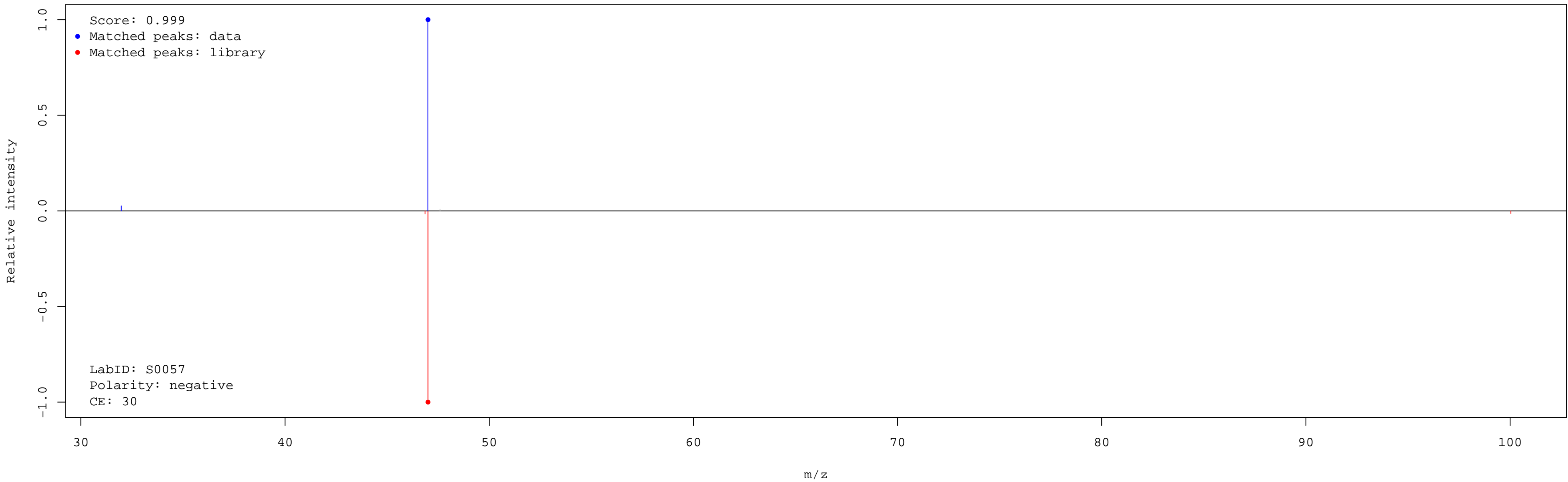

Supplement: Supplementary file 1 [file DataSheet1.ZIP › Supplementary table 1-10 and material 1-3/Material 3-Metlib-MSMS/NEG-Metlib-MSMS/Metlib-MSMS/M148T346_1_forward/0.999,L-Methionine,(M-H)-.pdf]

# Glyceraldehyde

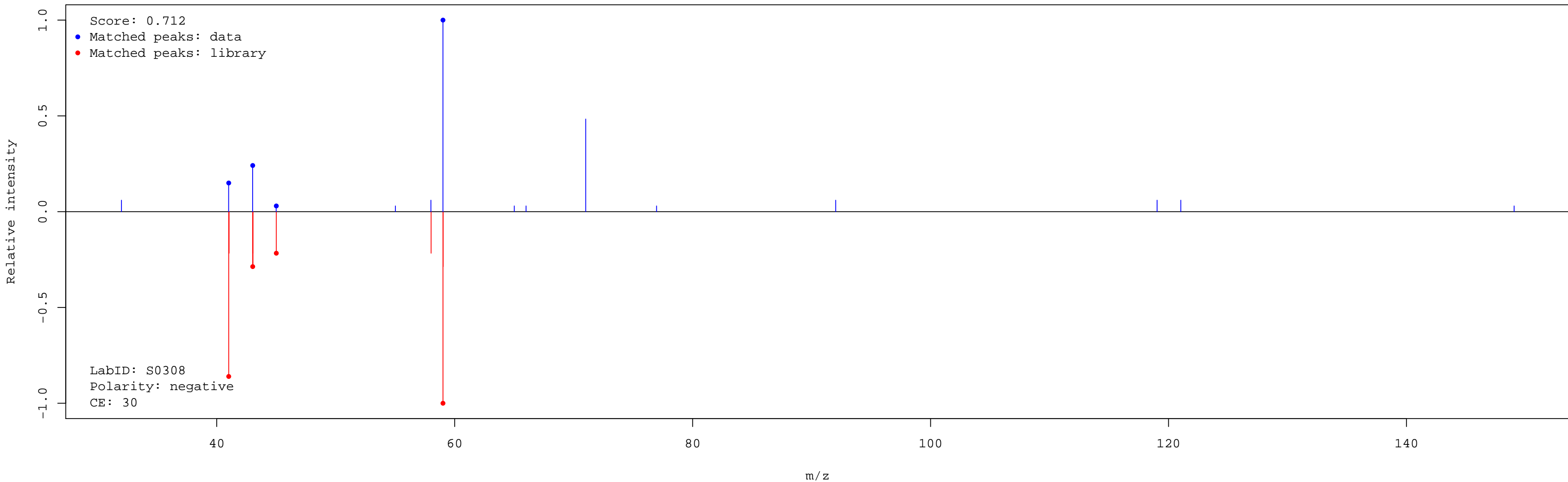

Supplement: Supplementary file 1 [file DataSheet1.ZIP › Supplementary table 1-10 and material 1-3/Material 3-Metlib-MSMS/NEG-Metlib-MSMS/Metlib-MSMS/M149T103_forward/0.712,Glyceraldehyde,(M+CH3COO)-.pdf]

# 3-Hydroxypropionic acid (beta-lactic acid)

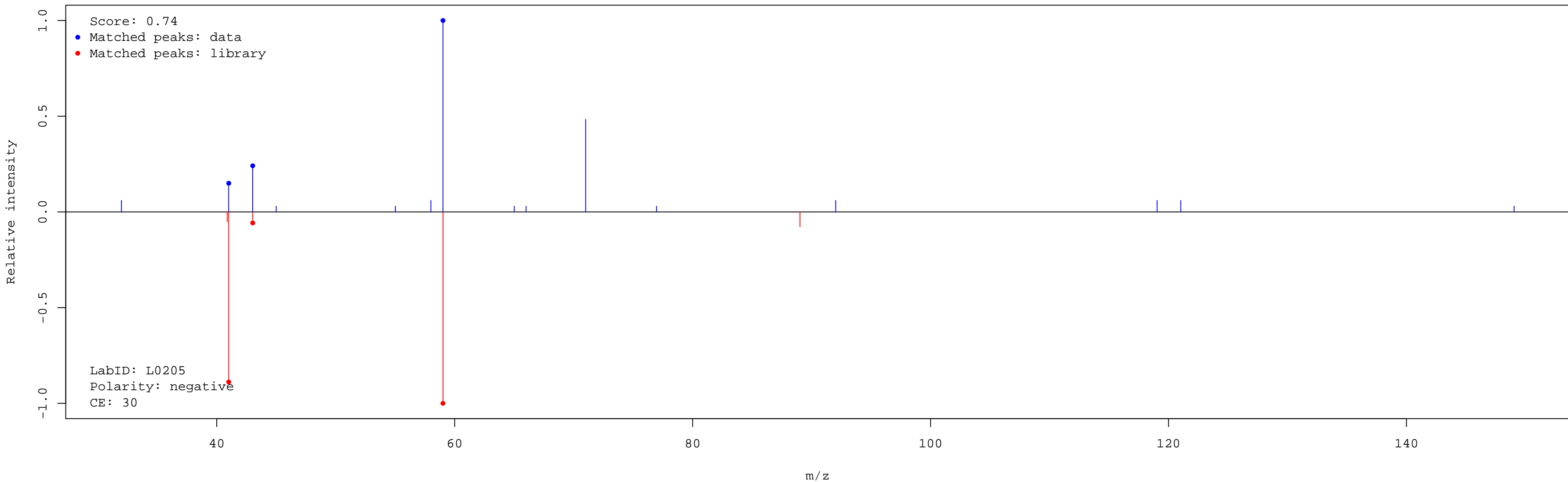

Supplement: Supplementary file 1 [file DataSheet1.ZIP › Supplementary table 1-10 and material 1-3/Material 3-Metlib-MSMS/NEG-Metlib-MSMS/Metlib-MSMS/M149T103_forward/0.74,3-Hydroxypropionic acid (beta-lactic acid),(M+CH3COO)-.pdf]

# D-Xylose

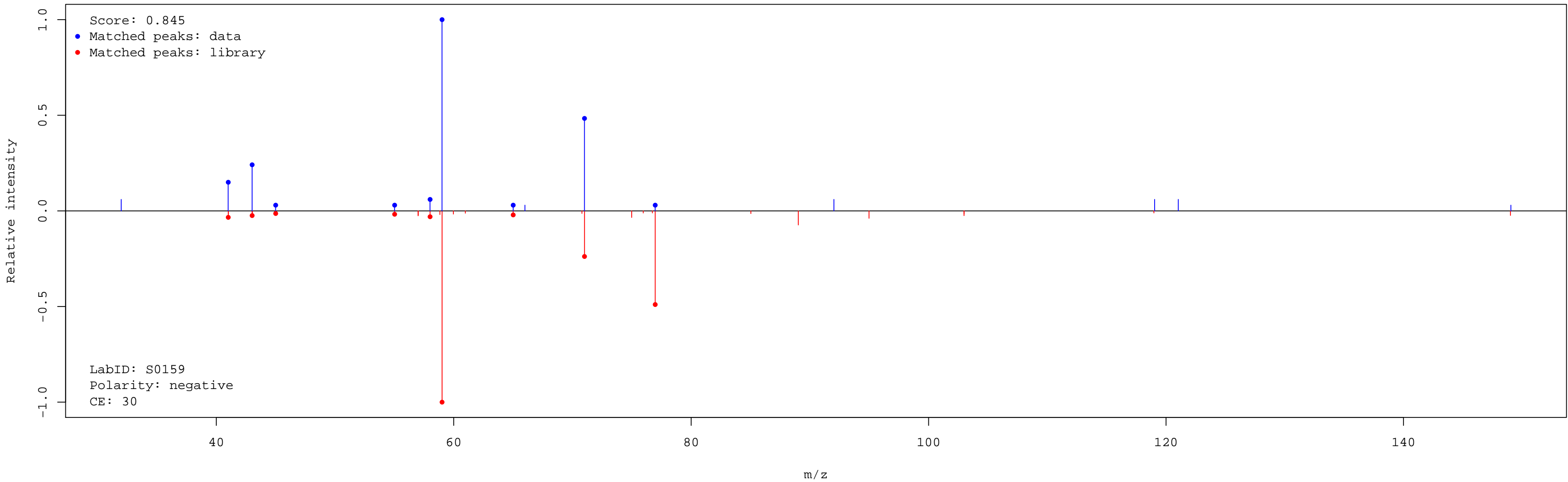

Supplement: Supplementary file 1 [file DataSheet1.ZIP › Supplementary table 1-10 and material 1-3/Material 3-Metlib-MSMS/NEG-Metlib-MSMS/Metlib-MSMS/M149T103_forward/0.845,D-Xylose,(M-H)-.pdf]

# D-Lyxose

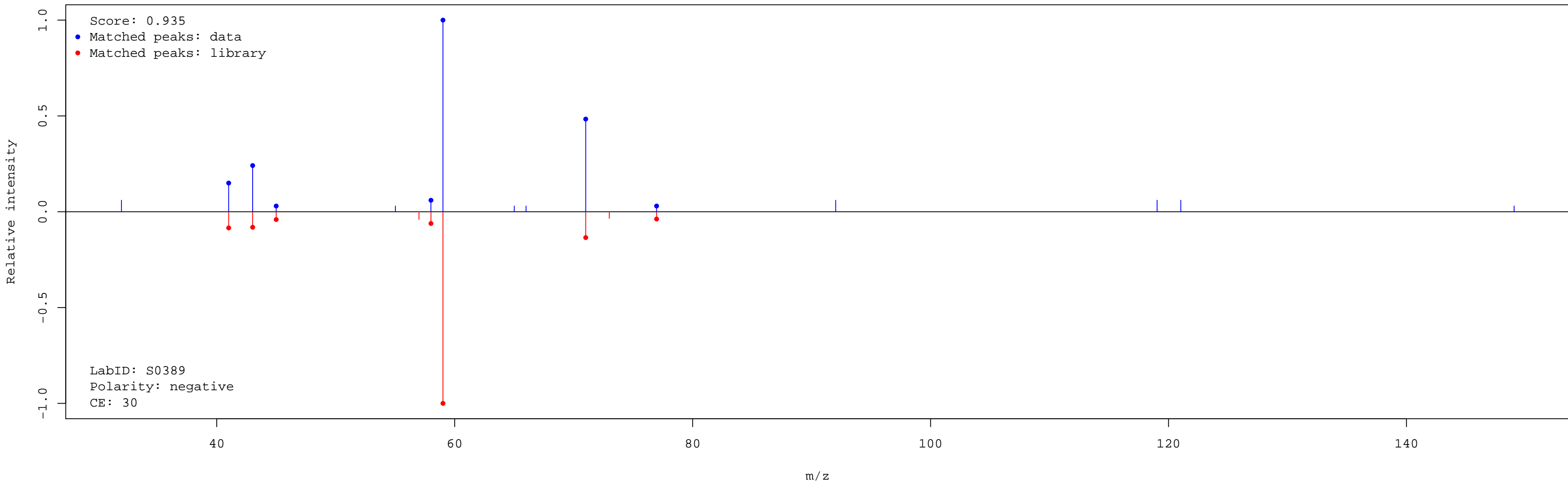

Supplement: Supplementary file 1 [file DataSheet1.ZIP › Supplementary table 1-10 and material 1-3/Material 3-Metlib-MSMS/NEG-Metlib-MSMS/Metlib-MSMS/M149T103_forward/0.935,D-Lyxose,(M-H)-.pdf]

# L-Arabinose

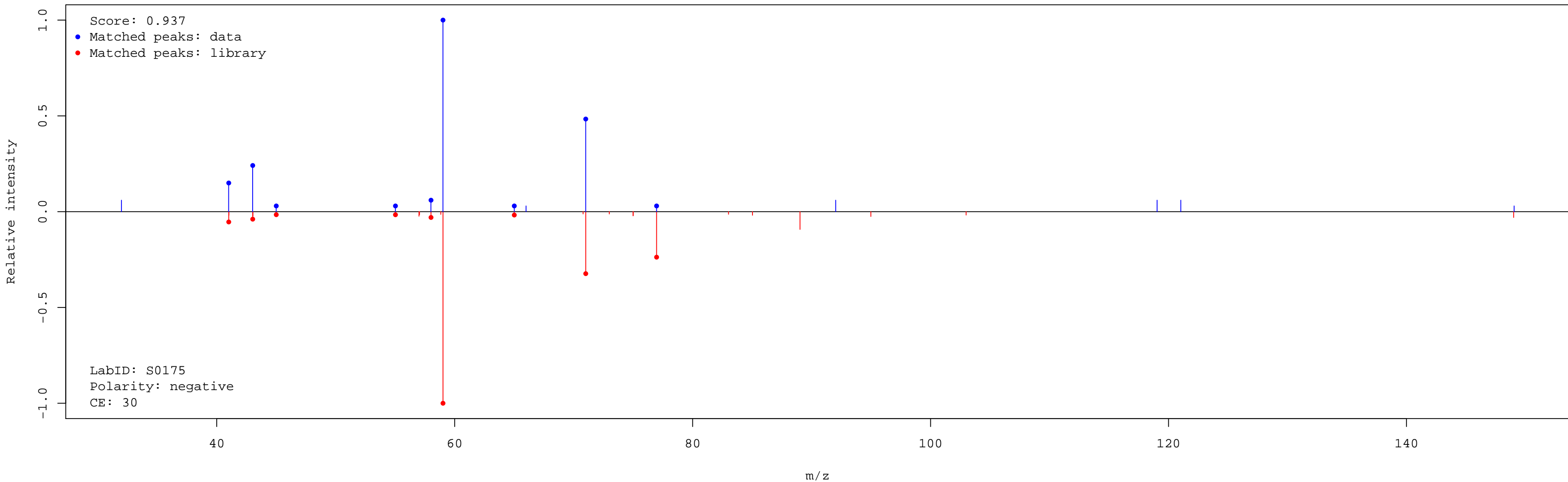

Supplement: Supplementary file 1 [file DataSheet1.ZIP › Supplementary table 1-10 and material 1-3/Material 3-Metlib-MSMS/NEG-Metlib-MSMS/Metlib-MSMS/M149T103_forward/0.937,L-Arabinose,(M-H)-.pdf]

# D-Ribose

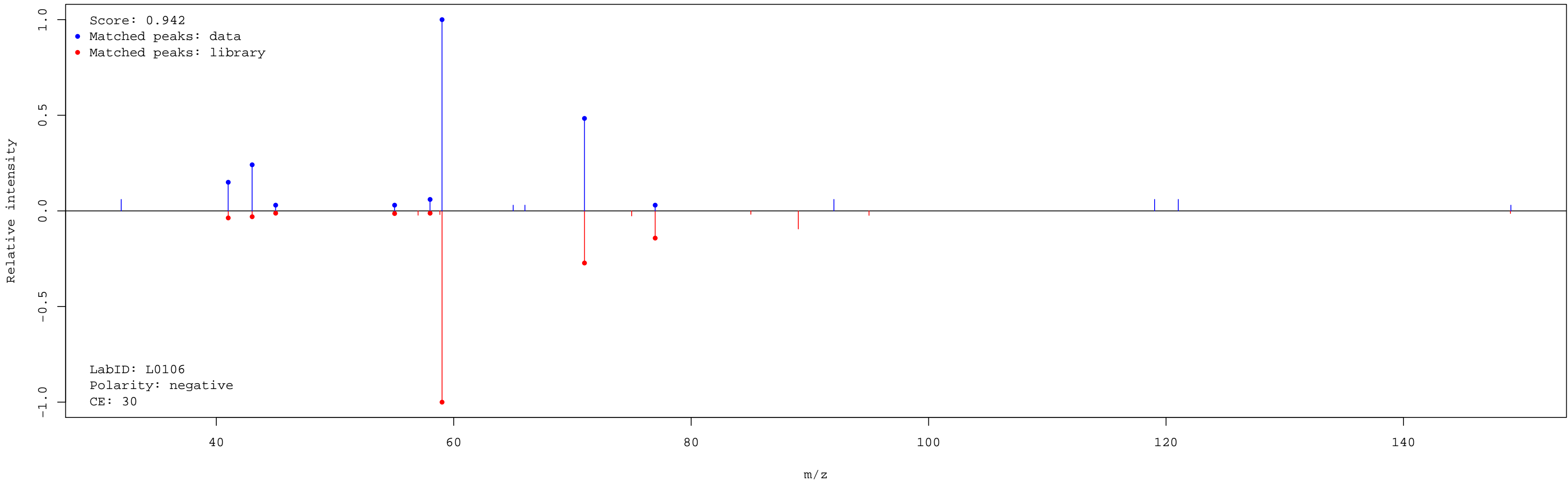

Supplement: Supplementary file 1 [file DataSheet1.ZIP › Supplementary table 1-10 and material 1-3/Material 3-Metlib-MSMS/NEG-Metlib-MSMS/Metlib-MSMS/M149T103_forward/0.942,D-Ribose,(M-H)-.pdf]

# Glyceraldehyde

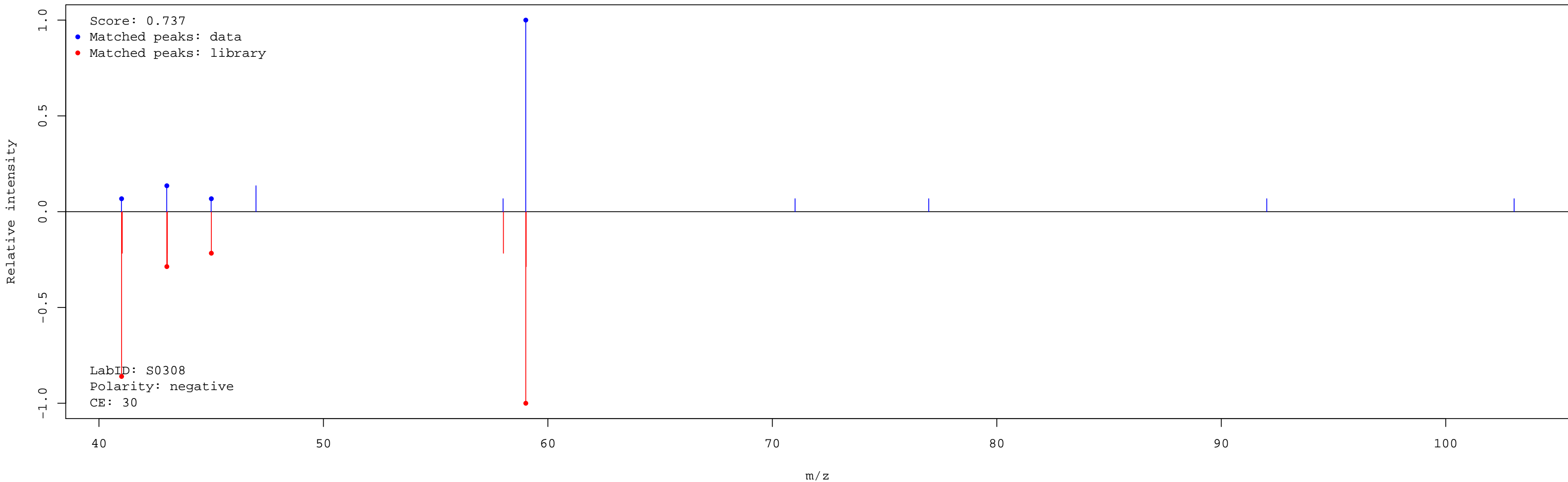

Supplement: Supplementary file 1 [file DataSheet1.ZIP › Supplementary table 1-10 and material 1-3/Material 3-Metlib-MSMS/NEG-Metlib-MSMS/Metlib-MSMS/M149T155_forward/0.737,Glyceraldehyde,(M+CH3COO)-.pdf]

# 3-Hydroxypropionic acid (beta-lactic acid)

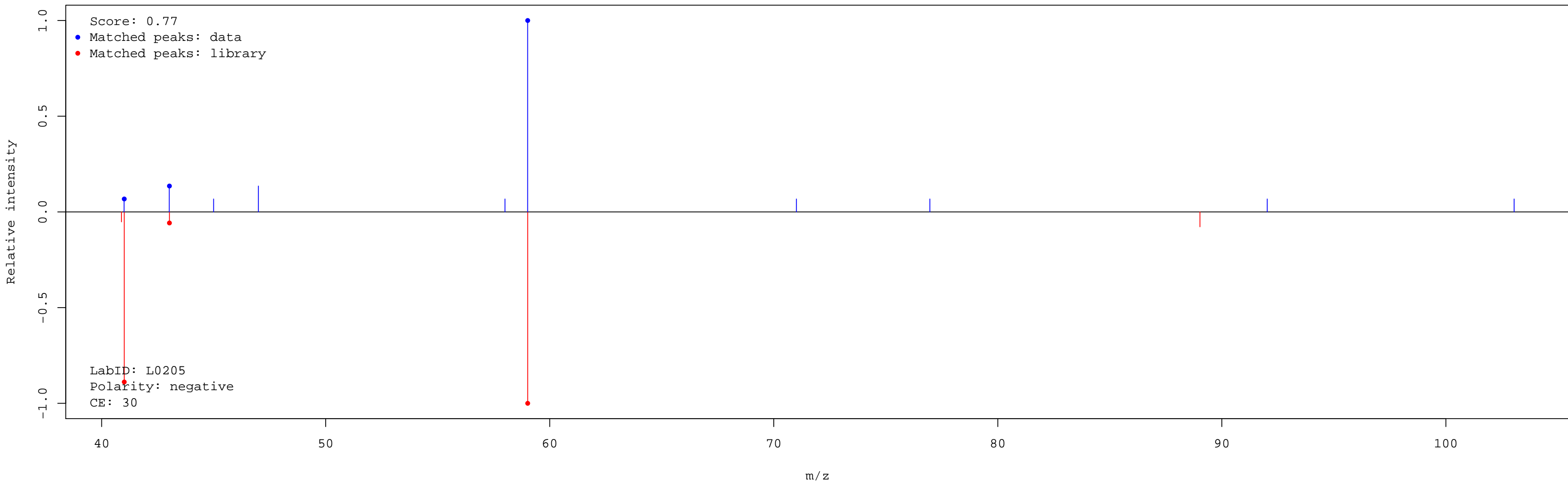

Supplement: Supplementary file 1 [file DataSheet1.ZIP › Supplementary table 1-10 and material 1-3/Material 3-Metlib-MSMS/NEG-Metlib-MSMS/Metlib-MSMS/M149T155_forward/0.77,3-Hydroxypropionic acid (beta-lactic acid),(M+CH3COO)-.pdf]

# D-Xylose

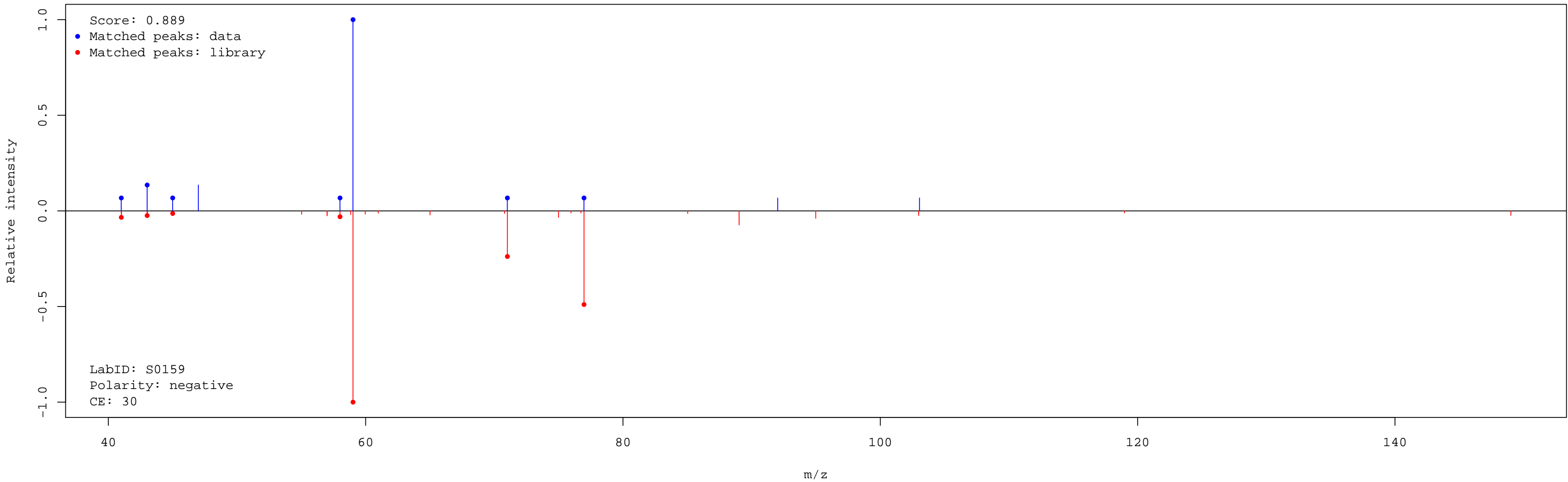

Supplement: Supplementary file 1 [file DataSheet1.ZIP › Supplementary table 1-10 and material 1-3/Material 3-Metlib-MSMS/NEG-Metlib-MSMS/Metlib-MSMS/M149T155_forward/0.889,D-Xylose,(M-H)-.pdf]

# L-Arabinose

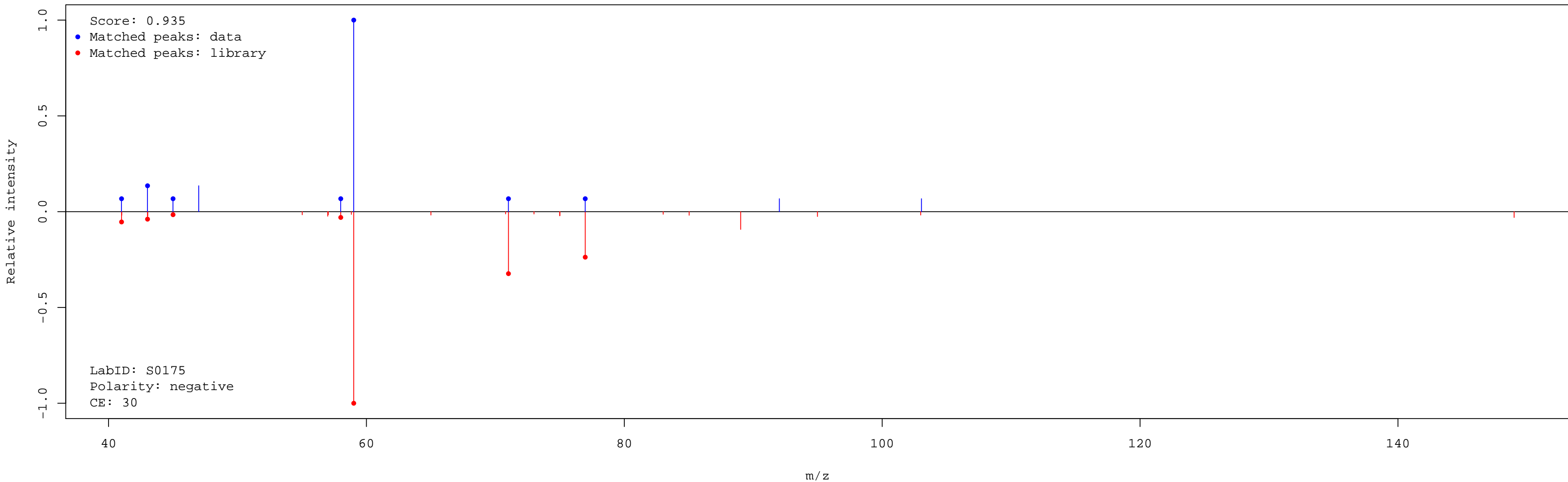

Supplement: Supplementary file 1 [file DataSheet1.ZIP › Supplementary table 1-10 and material 1-3/Material 3-Metlib-MSMS/NEG-Metlib-MSMS/Metlib-MSMS/M149T155_forward/0.935,L-Arabinose,(M-H)-.pdf]

# D-Ribose

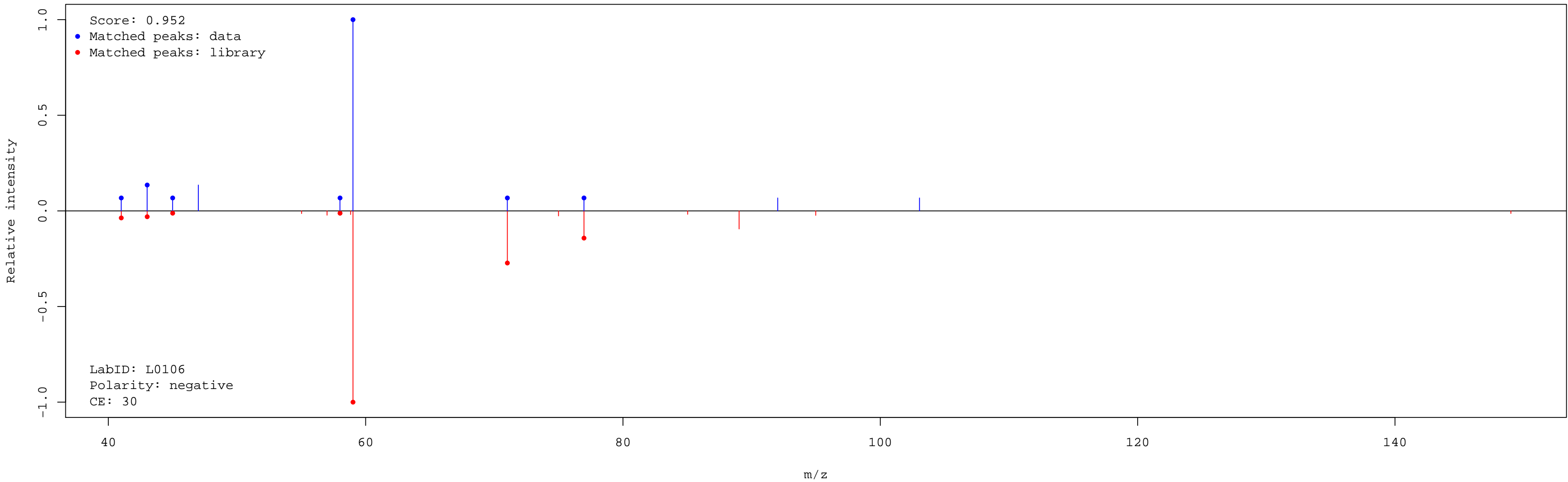

Supplement: Supplementary file 1 [file DataSheet1.ZIP › Supplementary table 1-10 and material 1-3/Material 3-Metlib-MSMS/NEG-Metlib-MSMS/Metlib-MSMS/M149T155_forward/0.952,D-Ribose,(M-H)-.pdf]

# D-Lyxose

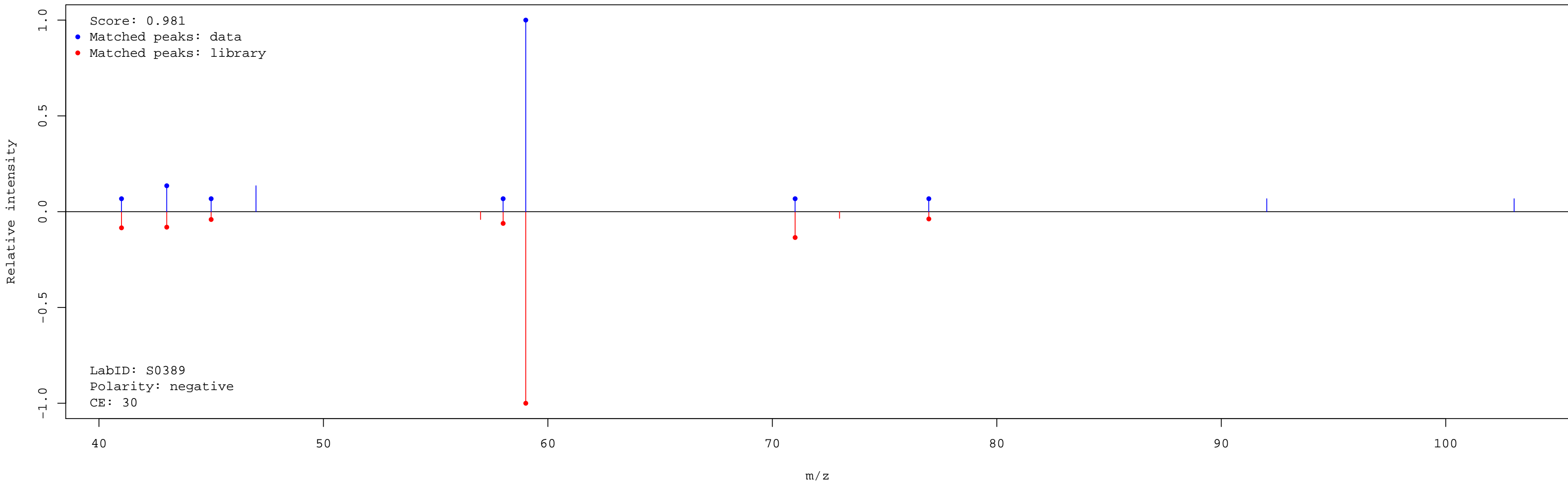

Supplement: Supplementary file 1 [file DataSheet1.ZIP › Supplementary table 1-10 and material 1-3/Material 3-Metlib-MSMS/NEG-Metlib-MSMS/Metlib-MSMS/M149T155_forward/0.981,D-Lyxose,(M-H)-.pdf]

# Glyceraldehyde

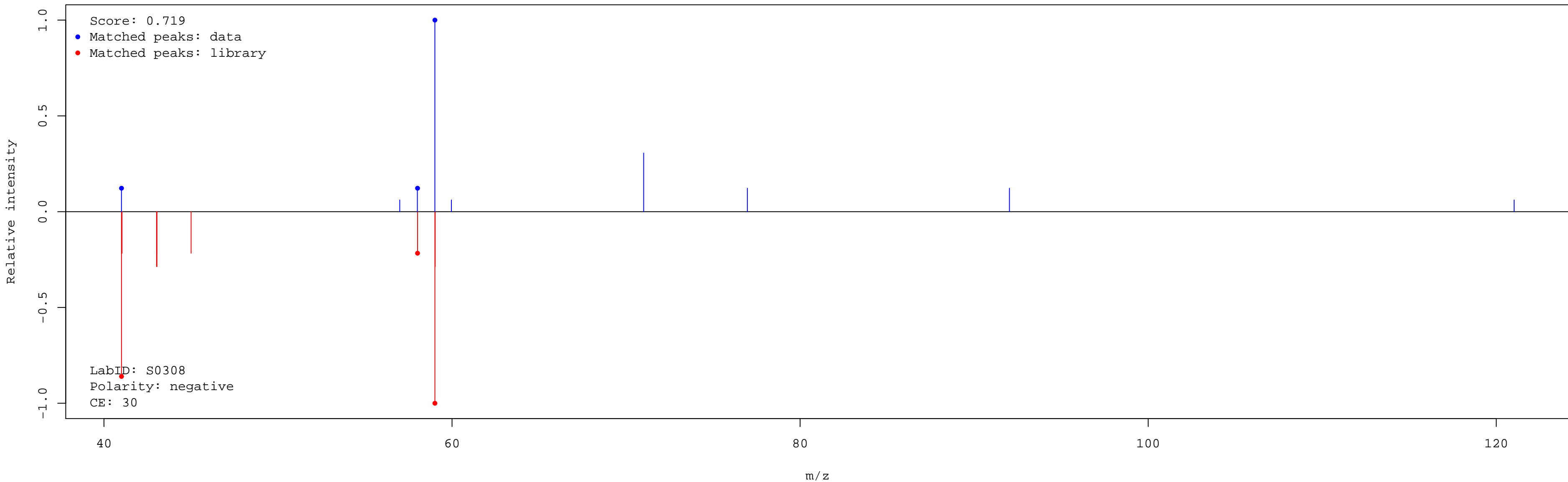

Supplement: Supplementary file 1 [file DataSheet1.ZIP › Supplementary table 1-10 and material 1-3/Material 3-Metlib-MSMS/NEG-Metlib-MSMS/Metlib-MSMS/M149T191_forward/0.719,Glyceraldehyde,(M+CH3COO)-.pdf]

# 3-Hydroxypropionic acid (beta-lactic acid)

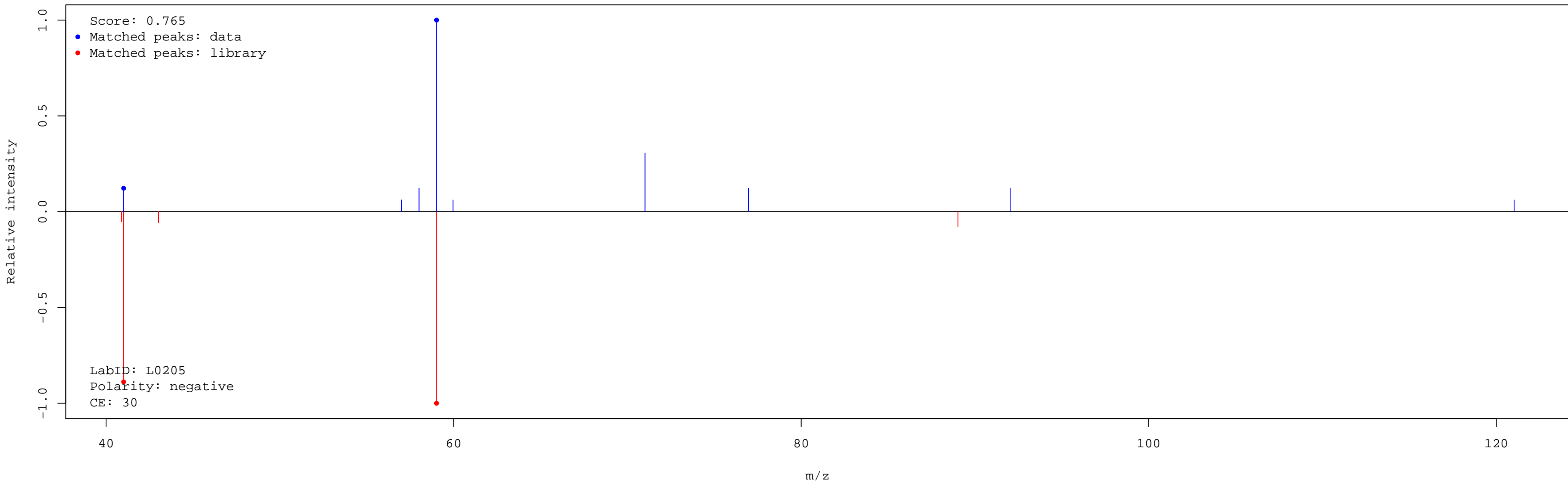

Supplement: Supplementary file 1 [file DataSheet1.ZIP › Supplementary table 1-10 and material 1-3/Material 3-Metlib-MSMS/NEG-Metlib-MSMS/Metlib-MSMS/M149T191_forward/0.765,3-Hydroxypropionic acid (beta-lactic acid),(M+CH3COO)-.pdf]

# D-Xylose

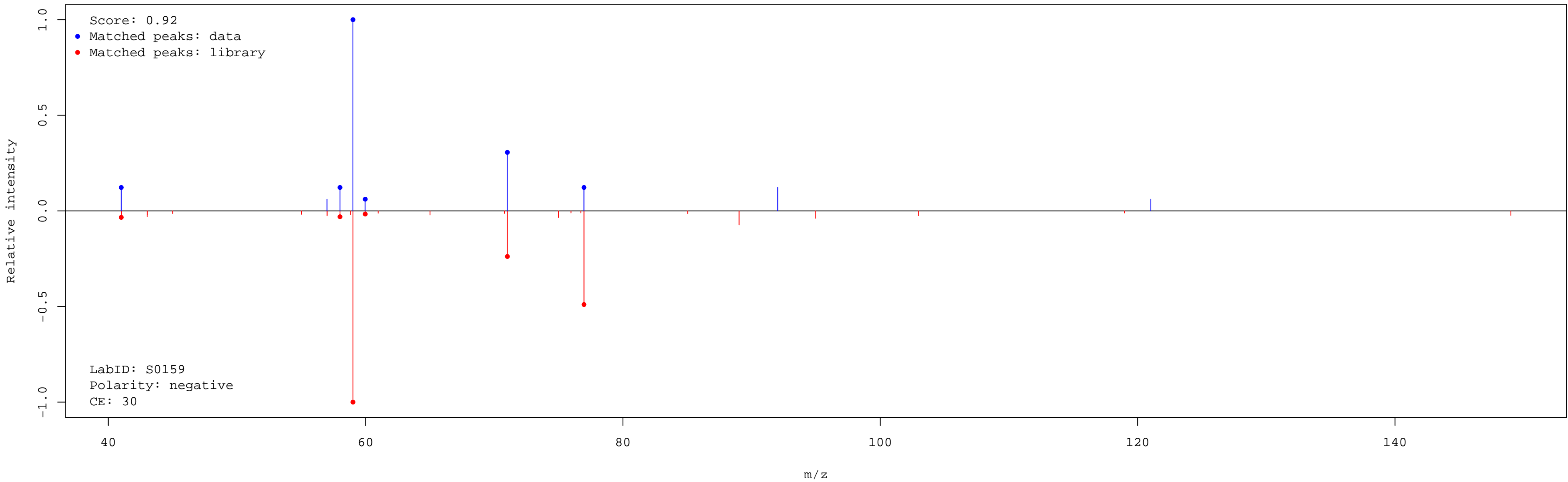

Supplement: Supplementary file 1 [file DataSheet1.ZIP › Supplementary table 1-10 and material 1-3/Material 3-Metlib-MSMS/NEG-Metlib-MSMS/Metlib-MSMS/M149T191_forward/0.92,D-Xylose,(M-H)-.pdf]

# D-Lyxose

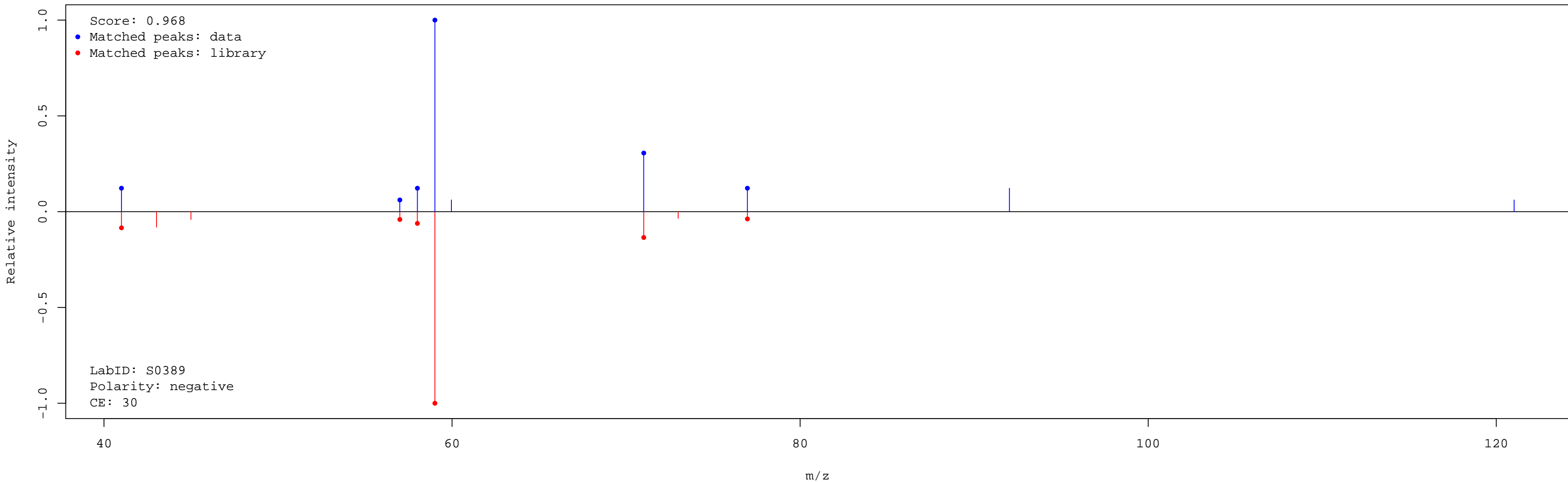

Supplement: Supplementary file 1 [file DataSheet1.ZIP › Supplementary table 1-10 and material 1-3/Material 3-Metlib-MSMS/NEG-Metlib-MSMS/Metlib-MSMS/M149T191_forward/0.968,D-Lyxose,(M-H)-.pdf]

# L-Arabinose

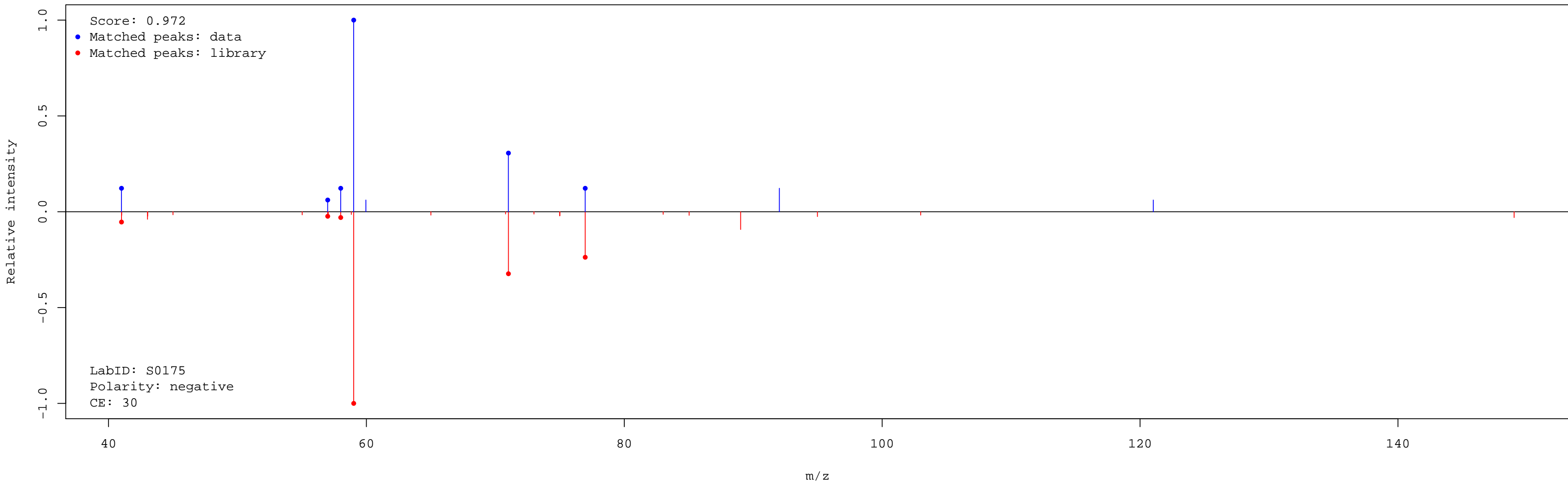

Supplement: Supplementary file 1 [file DataSheet1.ZIP › Supplementary table 1-10 and material 1-3/Material 3-Metlib-MSMS/NEG-Metlib-MSMS/Metlib-MSMS/M149T191_forward/0.972,L-Arabinose,(M-H)-.pdf]

# D-Ribose

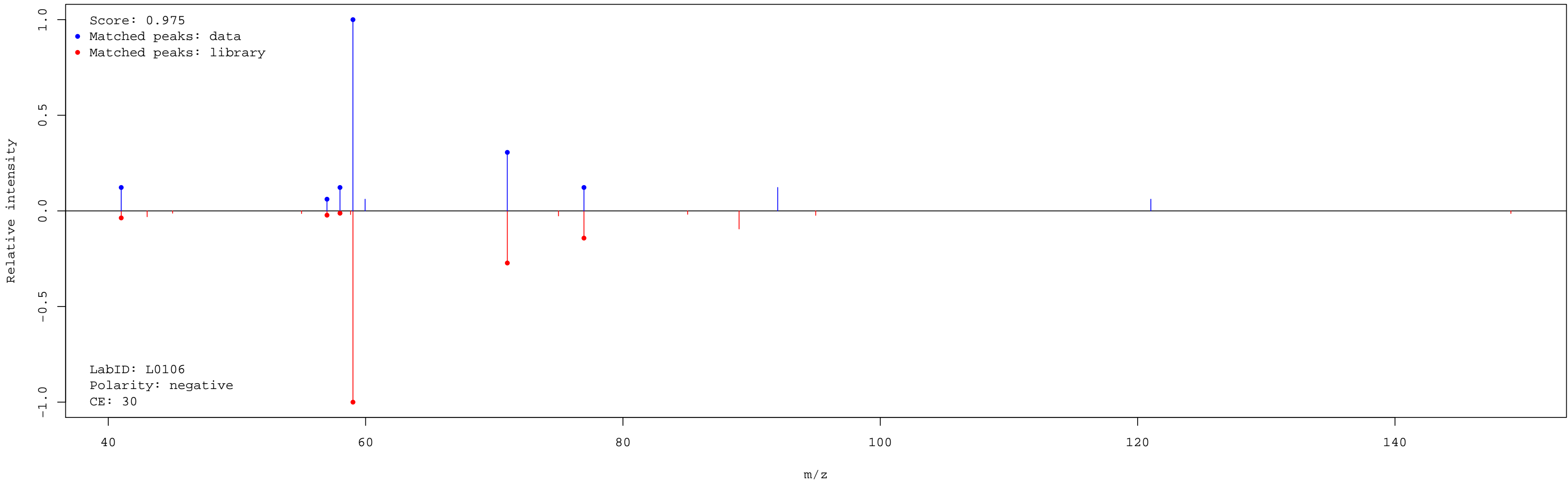

Supplement: Supplementary file 1 [file DataSheet1.ZIP › Supplementary table 1-10 and material 1-3/Material 3-Metlib-MSMS/NEG-Metlib-MSMS/Metlib-MSMS/M149T191_forward/0.975,D-Ribose,(M-H)-.pdf]

# Glyceraldehyde

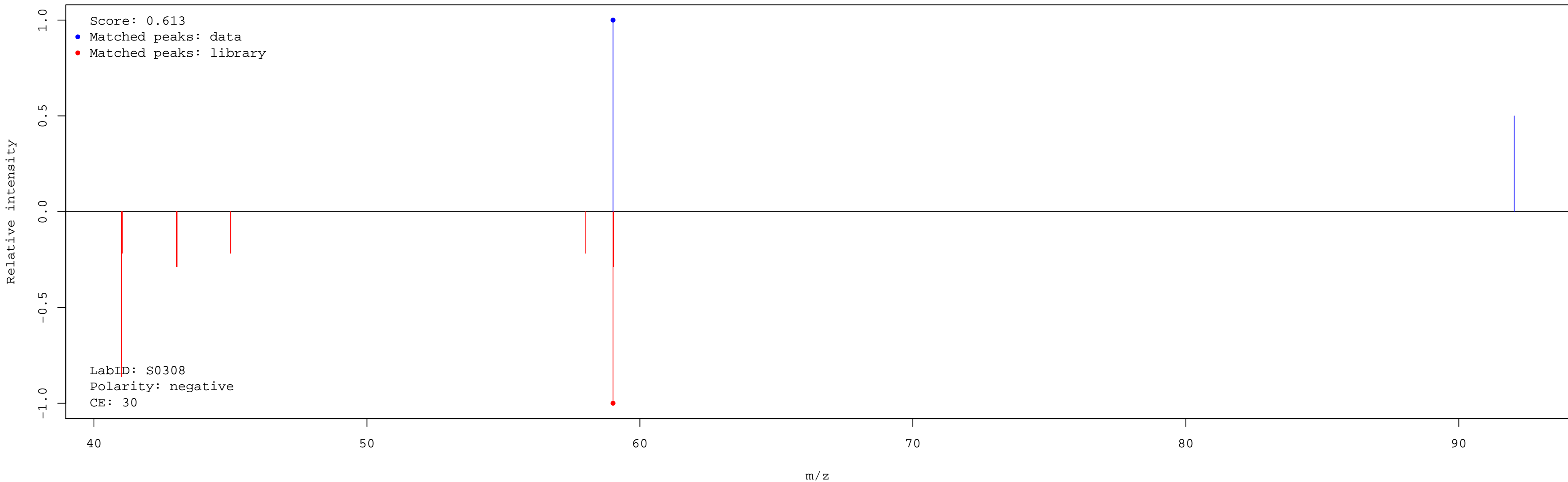

Supplement: Supplementary file 1 [file DataSheet1.ZIP › Supplementary table 1-10 and material 1-3/Material 3-Metlib-MSMS/NEG-Metlib-MSMS/Metlib-MSMS/M149T347_forward/0.613,Glyceraldehyde,(M+CH3COO)-.pdf]

# 3-Hydroxypropionic acid (beta-lactic acid)

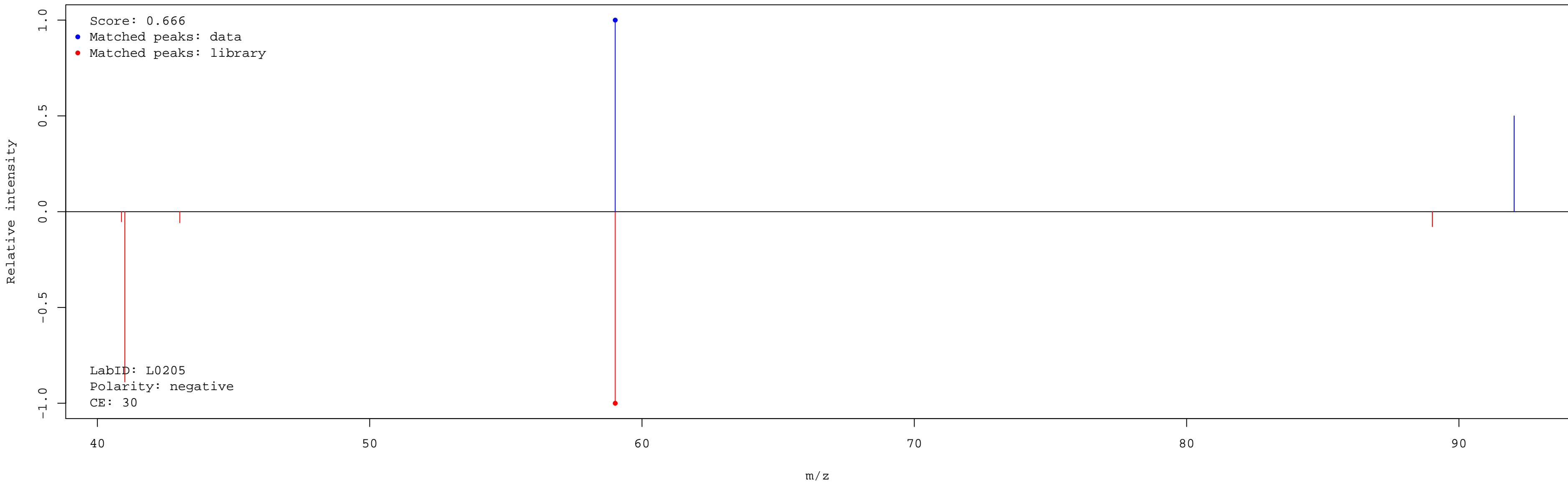

Supplement: Supplementary file 1 [file DataSheet1.ZIP › Supplementary table 1-10 and material 1-3/Material 3-Metlib-MSMS/NEG-Metlib-MSMS/Metlib-MSMS/M149T347_forward/0.666,3-Hydroxypropionic acid (beta-lactic acid),(M+CH3COO)-.pdf]

# D-Xylose

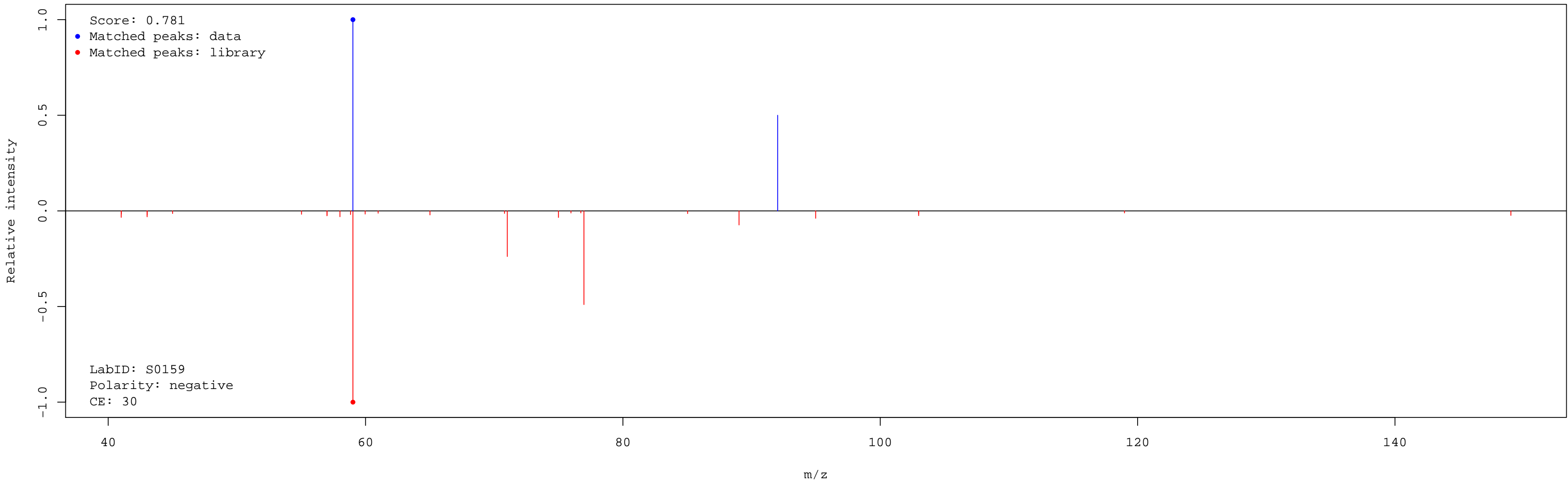

Supplement: Supplementary file 1 [file DataSheet1.ZIP › Supplementary table 1-10 and material 1-3/Material 3-Metlib-MSMS/NEG-Metlib-MSMS/Metlib-MSMS/M149T347_forward/0.781,D-Xylose,(M-H)-.pdf]

# L-Arabinose

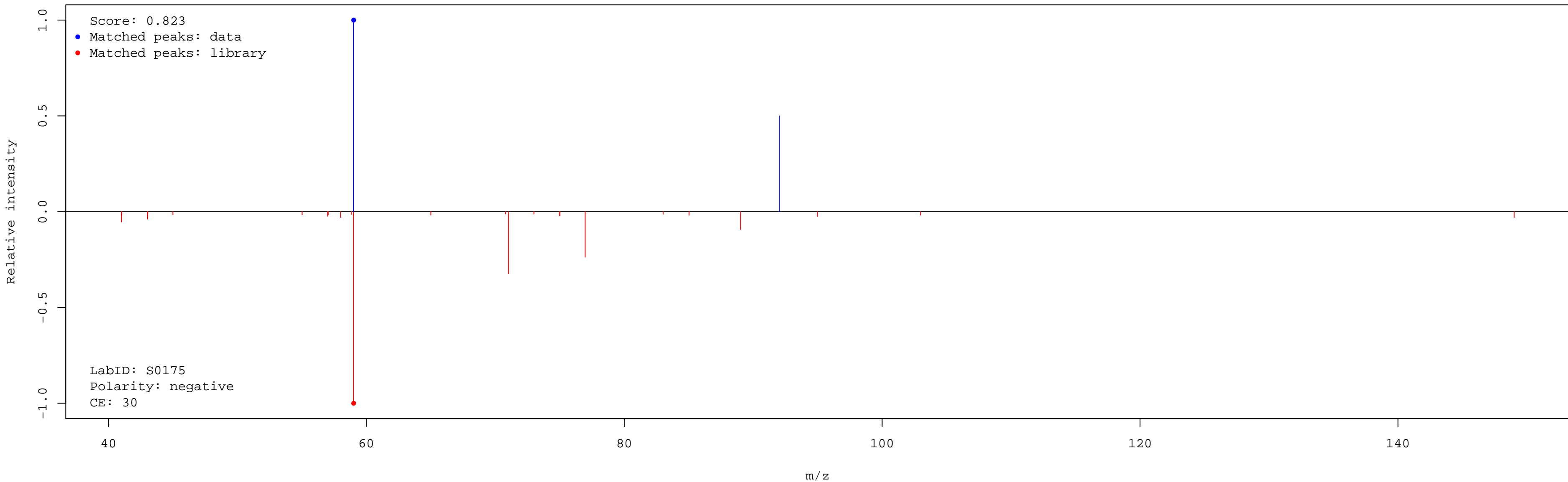

Supplement: Supplementary file 1 [file DataSheet1.ZIP › Supplementary table 1-10 and material 1-3/Material 3-Metlib-MSMS/NEG-Metlib-MSMS/Metlib-MSMS/M149T347_forward/0.823,L-Arabinose,(M-H)-.pdf]

# D-Ribose

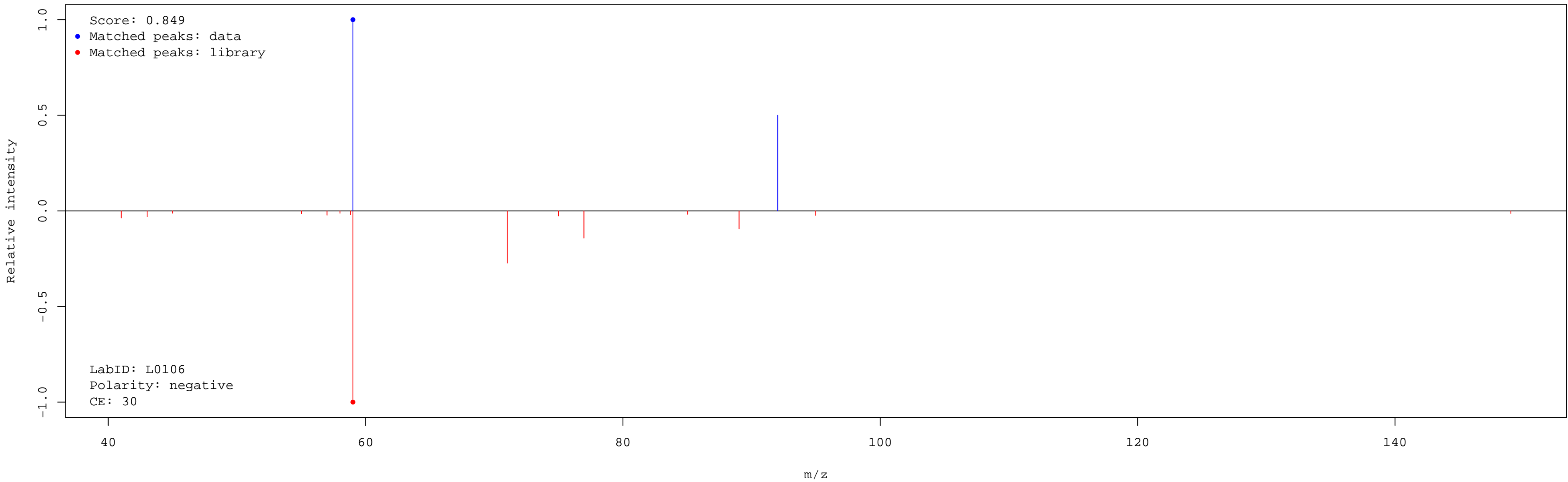

Supplement: Supplementary file 1 [file DataSheet1.ZIP › Supplementary table 1-10 and material 1-3/Material 3-Metlib-MSMS/NEG-Metlib-MSMS/Metlib-MSMS/M149T347_forward/0.849,D-Ribose,(M-H)-.pdf]

# D-Lyxose

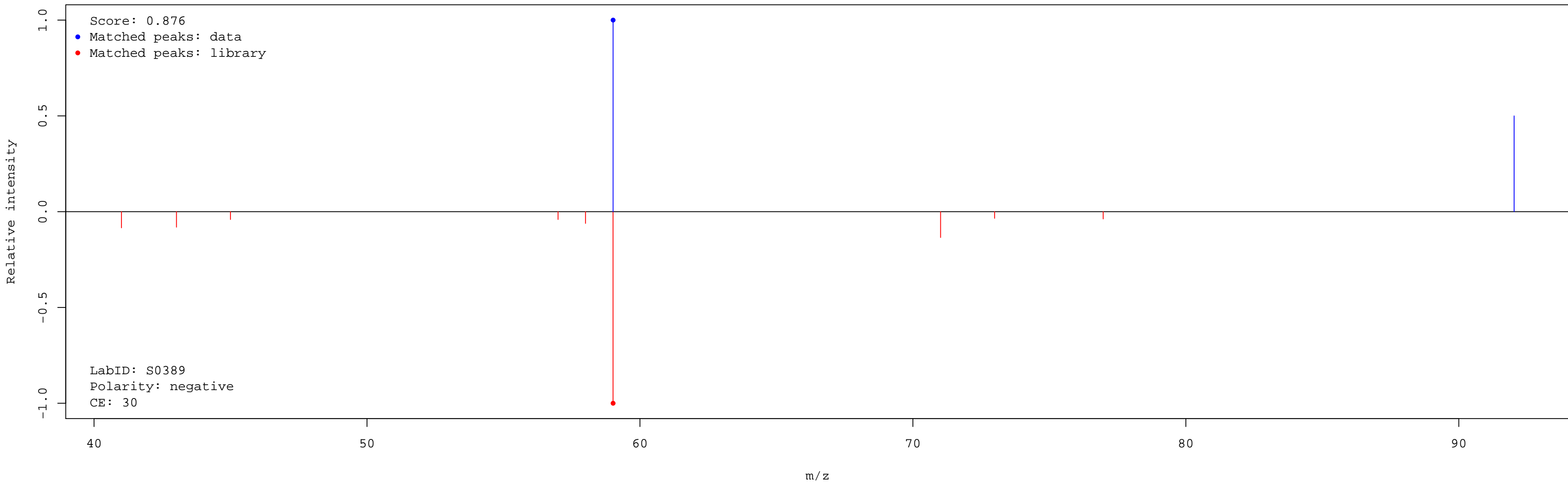

Supplement: Supplementary file 1 [file DataSheet1.ZIP › Supplementary table 1-10 and material 1-3/Material 3-Metlib-MSMS/NEG-Metlib-MSMS/Metlib-MSMS/M149T347_forward/0.876,D-Lyxose,(M-H)-.pdf]

L-Methionine

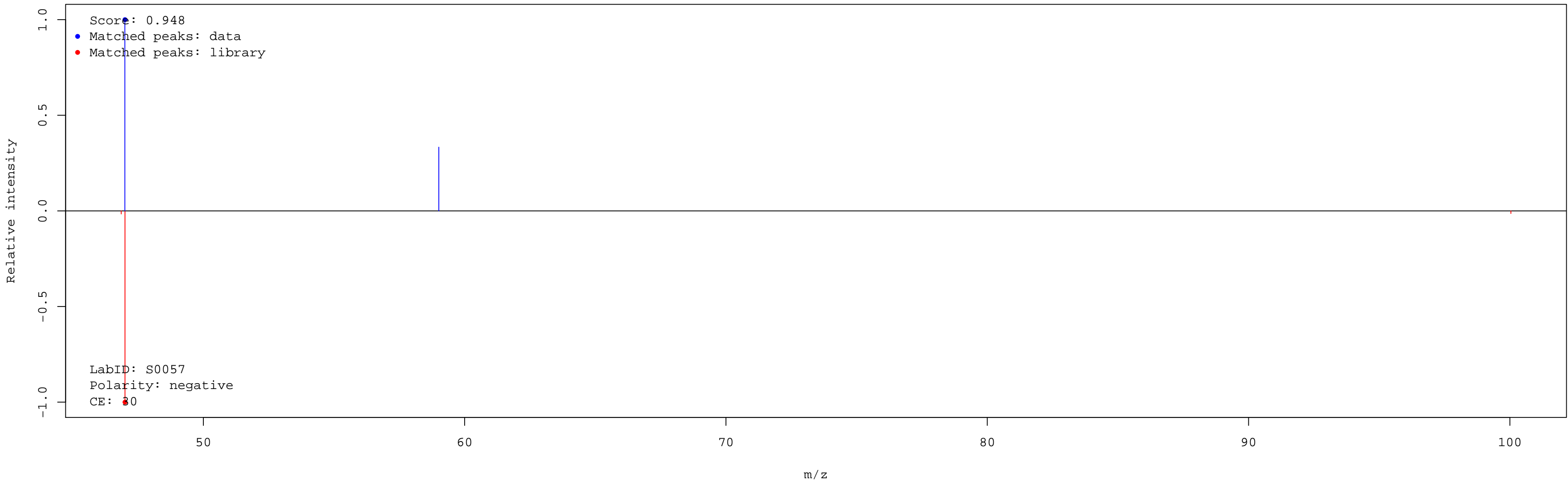

Supplement: Supplementary file 1 [file DataSheet1.ZIP › Supplementary table 1-10 and material 1-3/Material 3-Metlib-MSMS/NEG-Metlib-MSMS/Metlib-MSMS/M149T399_forward/0.948,L-Methionine,M-.pdf]

Phosphoenolpyruvate

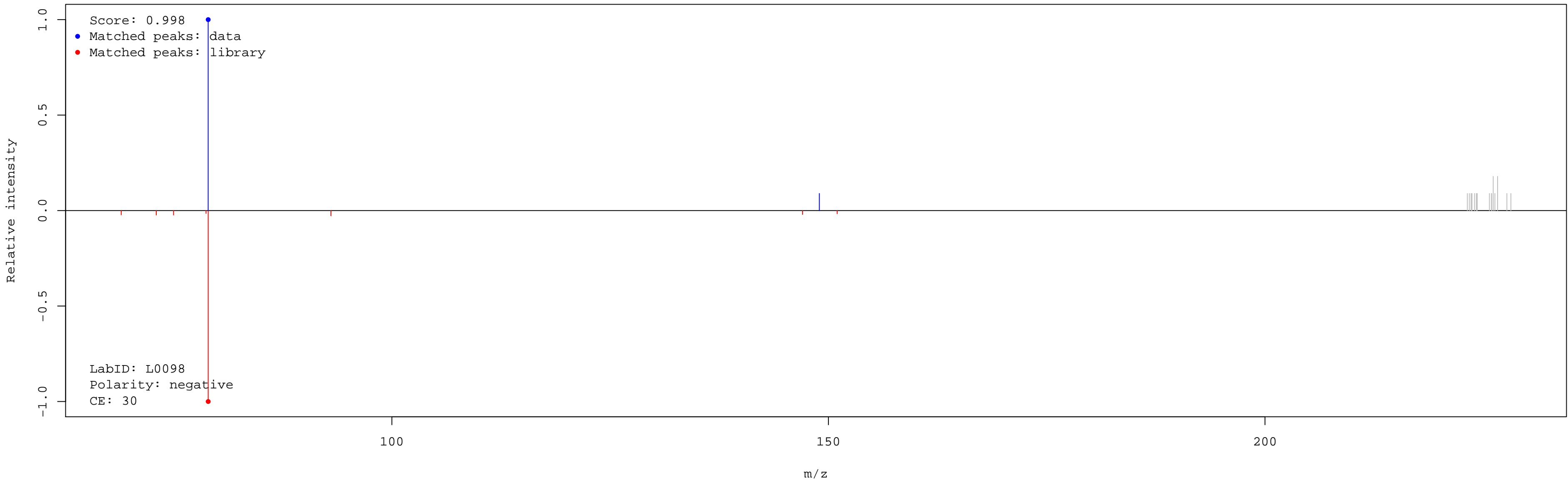

Supplement: Supplementary file 1 [file DataSheet1.ZIP › Supplementary table 1-10 and material 1-3/Material 3-Metlib-MSMS/NEG-Metlib-MSMS/Metlib-MSMS/M149T464_forward/0.998,Phosphoenolpyruvate,(M-H2O-H)-.pdf]

# D-Xylose

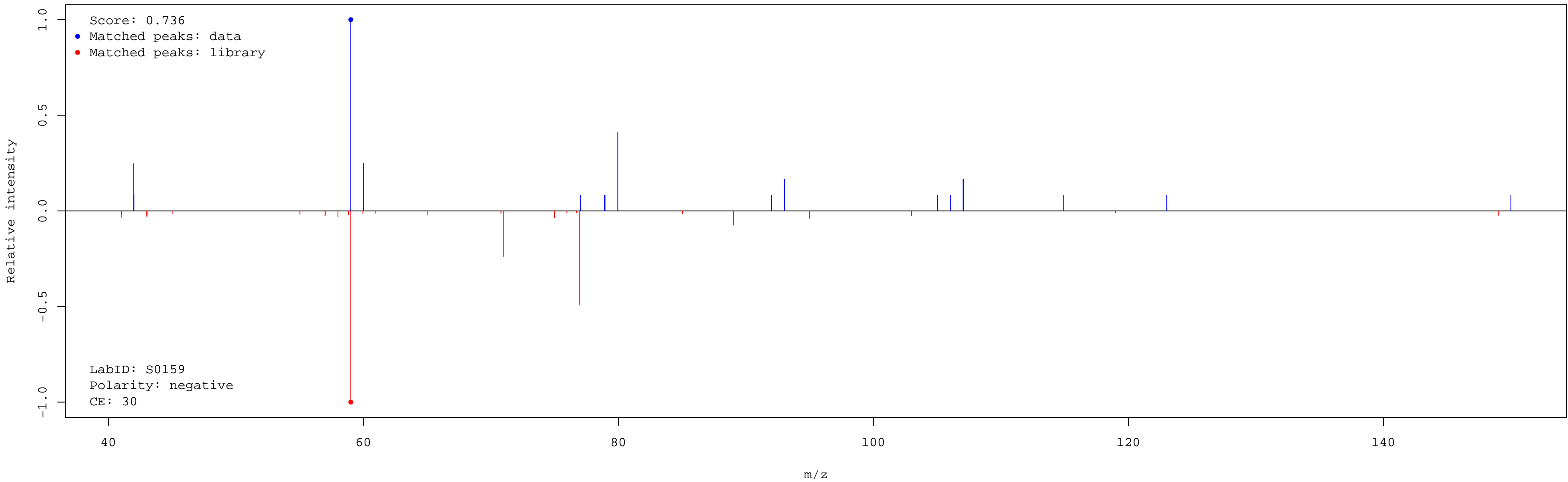

Supplement: Supplementary file 1 [file DataSheet1.ZIP › Supplementary table 1-10 and material 1-3/Material 3-Metlib-MSMS/NEG-Metlib-MSMS/Metlib-MSMS/M150T103_forward/0.736,D-Xylose,M-.pdf]

# L-Arabinose

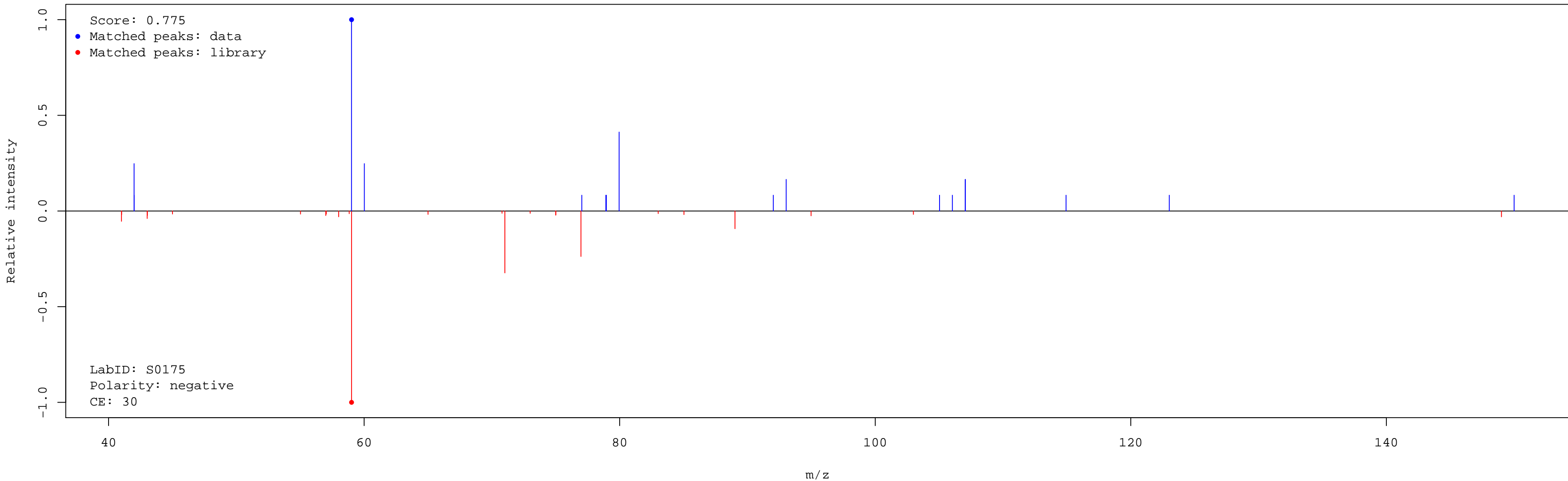

Supplement: Supplementary file 1 [file DataSheet1.ZIP › Supplementary table 1-10 and material 1-3/Material 3-Metlib-MSMS/NEG-Metlib-MSMS/Metlib-MSMS/M150T103_forward/0.775,L-Arabinose,M-.pdf]

# D-Ribose

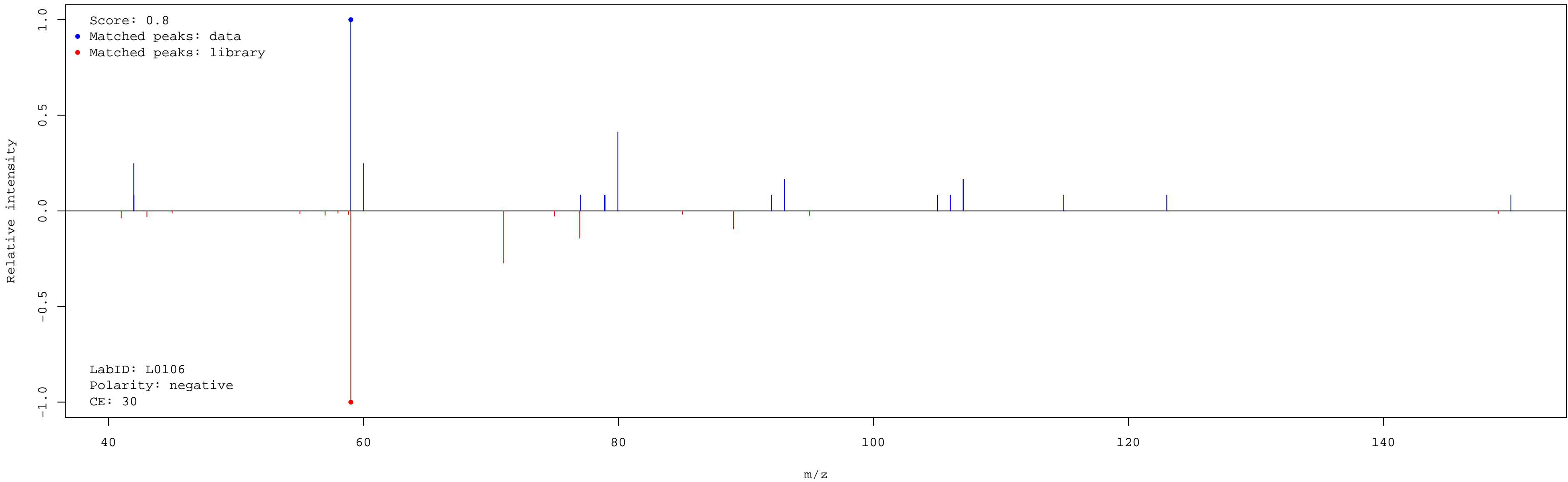

Supplement: Supplementary file 1 [file DataSheet1.ZIP › Supplementary table 1-10 and material 1-3/Material 3-Metlib-MSMS/NEG-Metlib-MSMS/Metlib-MSMS/M150T103_forward/0.8,D-Ribose,M-.pdf]

# D-Lyxose

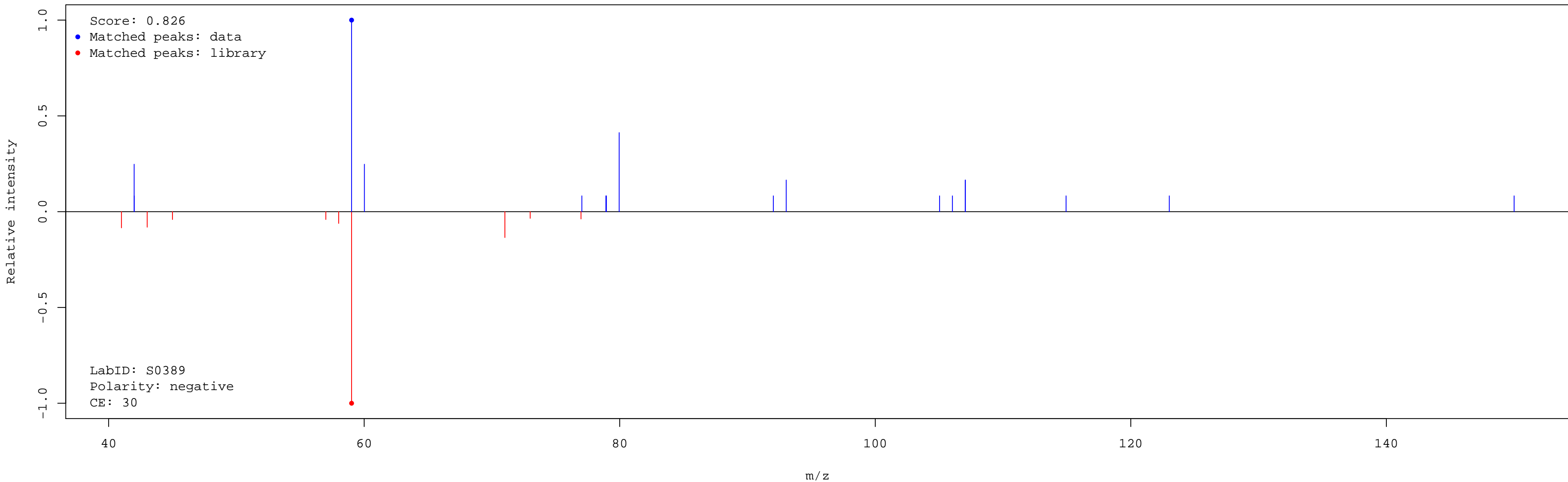

Supplement: Supplementary file 1 [file DataSheet1.ZIP › Supplementary table 1-10 and material 1-3/Material 3-Metlib-MSMS/NEG-Metlib-MSMS/Metlib-MSMS/M150T103_forward/0.826,D-Lyxose,M-.pdf]

# Oxypurinol

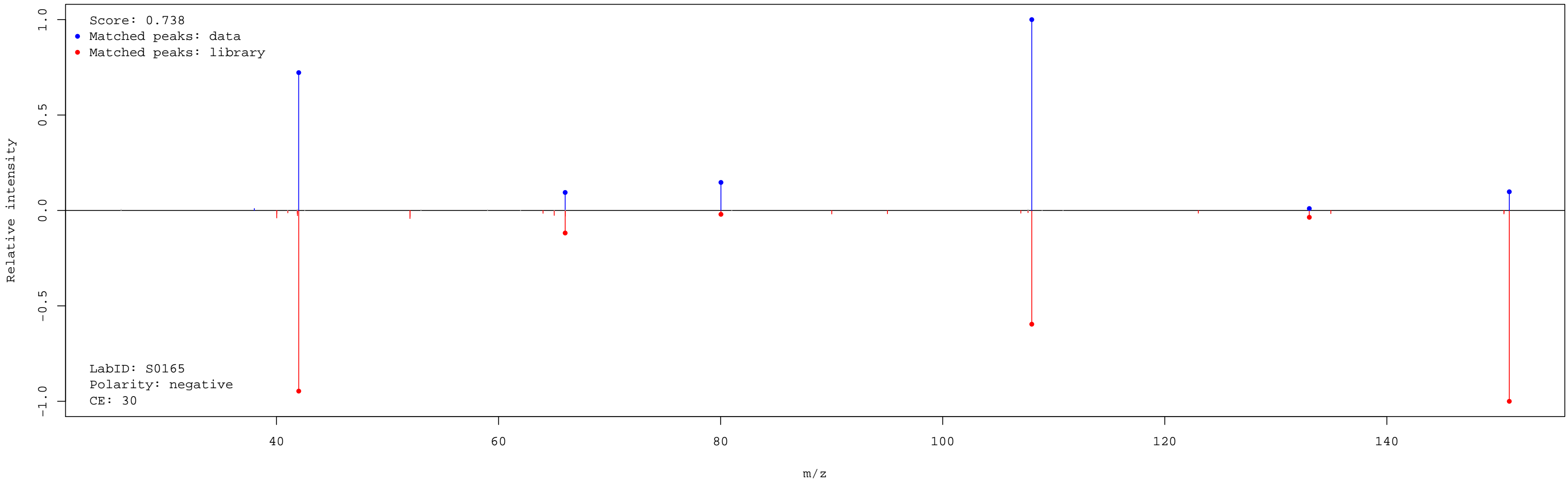

Supplement: Supplementary file 1 [file DataSheet1.ZIP › Supplementary table 1-10 and material 1-3/Material 3-Metlib-MSMS/NEG-Metlib-MSMS/Metlib-MSMS/M151T219_2_forward/0.738,Oxypurinol,(M-H)-.pdf]

# Xanthine

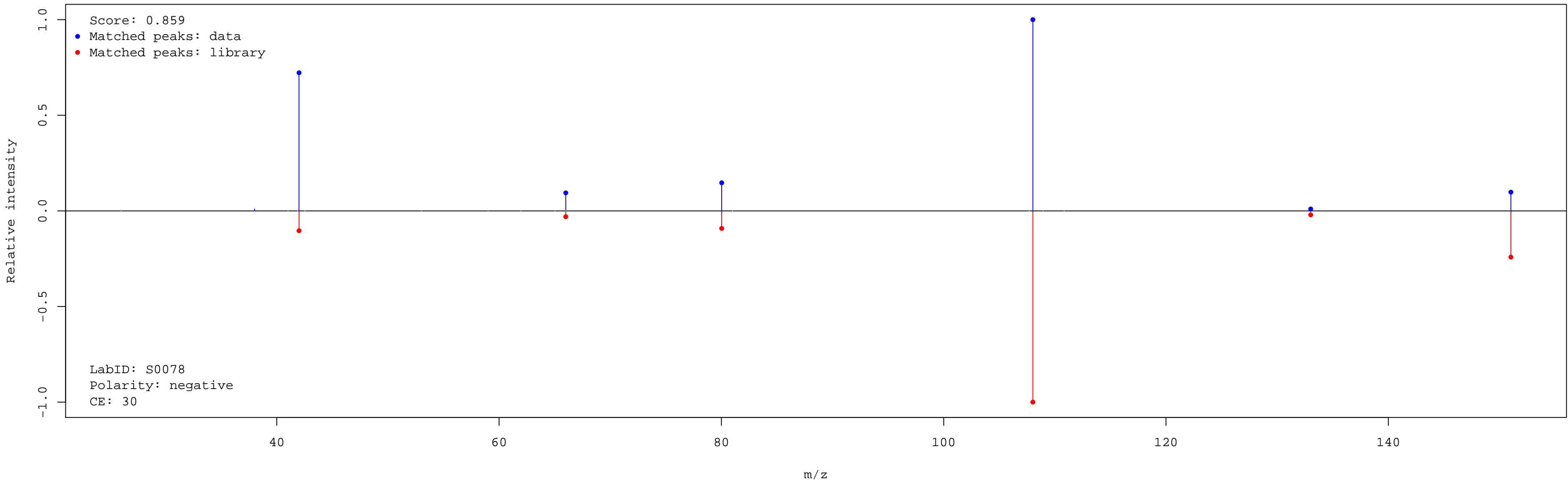

Supplement: Supplementary file 1 [file DataSheet1.ZIP › Supplementary table 1-10 and material 1-3/Material 3-Metlib-MSMS/NEG-Metlib-MSMS/Metlib-MSMS/M151T219_2_forward/0.859,Xanthine,(M-H)-.pdf]

# Ribitol

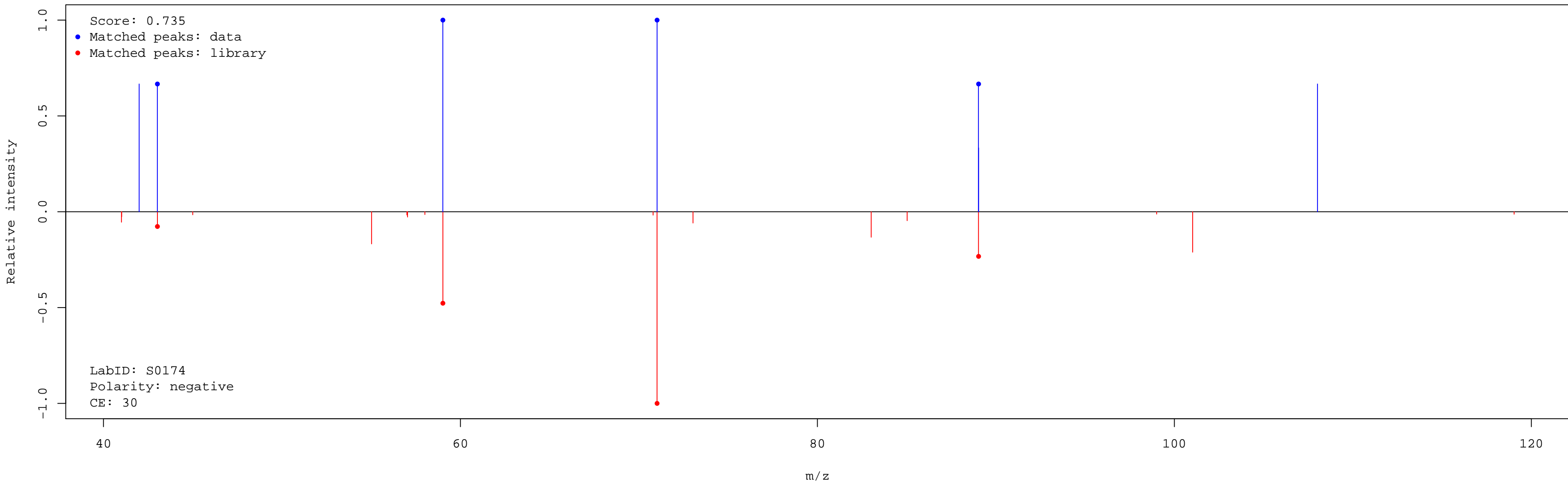

Supplement: Supplementary file 1 [file DataSheet1.ZIP › Supplementary table 1-10 and material 1-3/Material 3-Metlib-MSMS/NEG-Metlib-MSMS/Metlib-MSMS/M151T241_forward/0.735,Ribitol,(M-H)-.pdf]

# Xylitol

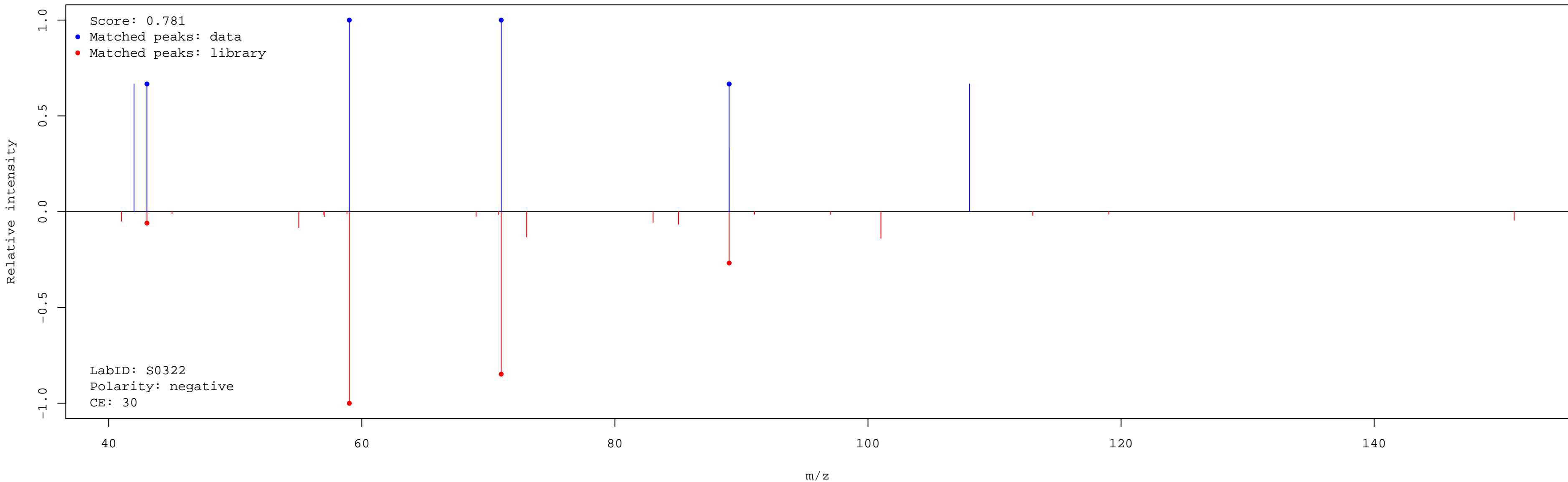

Supplement: Supplementary file 1 [file DataSheet1.ZIP › Supplementary table 1-10 and material 1-3/Material 3-Metlib-MSMS/NEG-Metlib-MSMS/Metlib-MSMS/M151T241_forward/0.781,Xylitol,(M-H)-.pdf]

# Oxypurinol

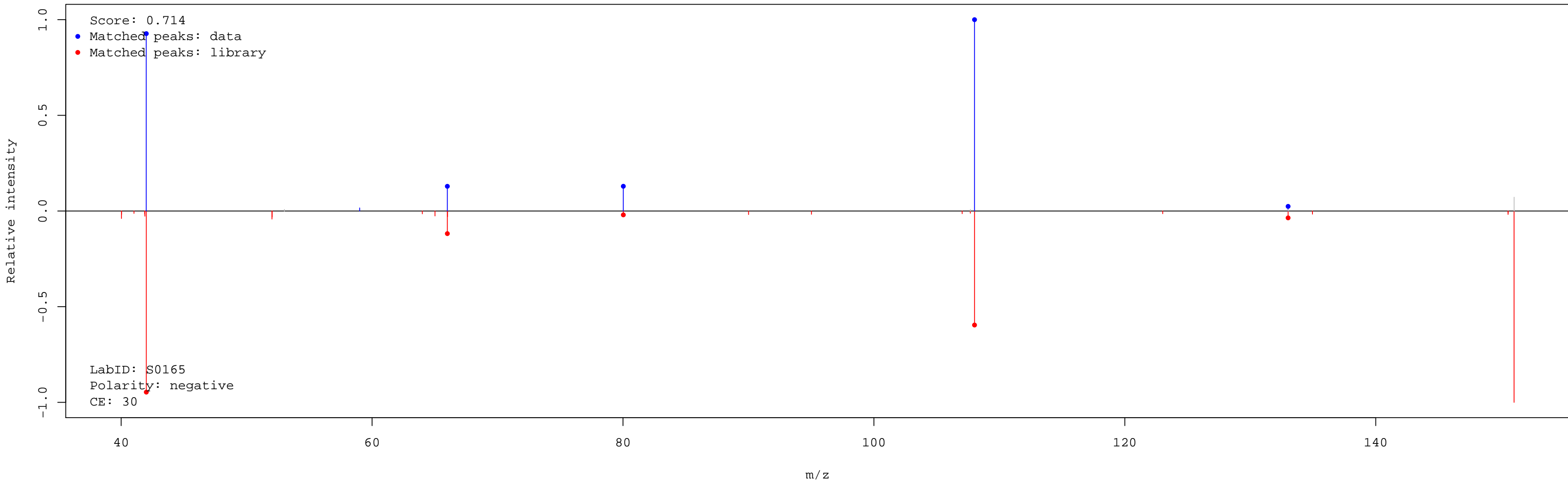

Supplement: Supplementary file 1 [file DataSheet1.ZIP › Supplementary table 1-10 and material 1-3/Material 3-Metlib-MSMS/NEG-Metlib-MSMS/Metlib-MSMS/M151T317_forward/0.714,Oxypurinol,(M-H)-.pdf]

# Xanthine

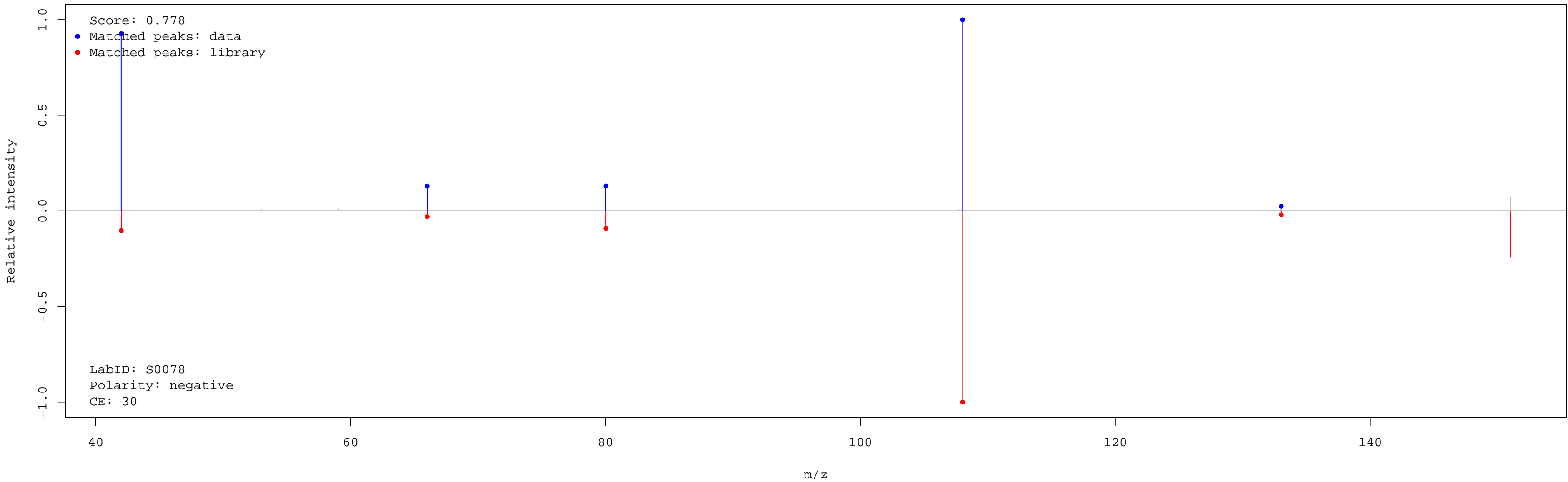

Supplement: Supplementary file 1 [file DataSheet1.ZIP › Supplementary table 1-10 and material 1-3/Material 3-Metlib-MSMS/NEG-Metlib-MSMS/Metlib-MSMS/M151T317_forward/0.778,Xanthine,(M-H)-.pdf]

# Oxypurinol

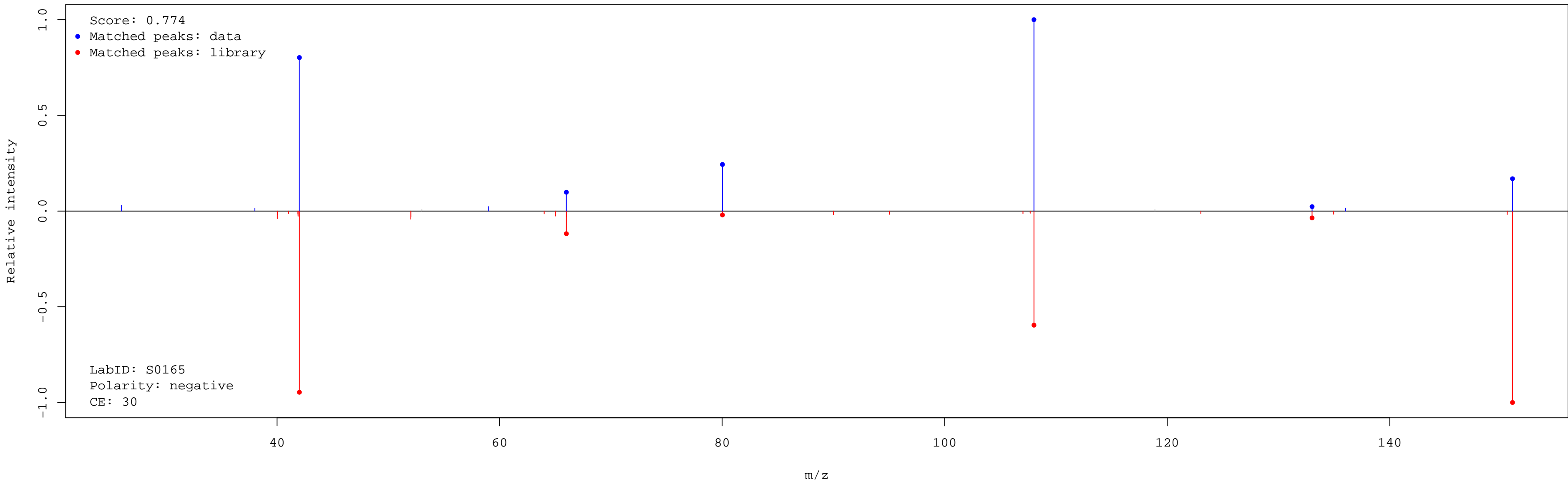

Supplement: Supplementary file 1 [file DataSheet1.ZIP › Supplementary table 1-10 and material 1-3/Material 3-Metlib-MSMS/NEG-Metlib-MSMS/Metlib-MSMS/M151T343_forward/0.774,Oxypurinol,(M-H)-.pdf]

# Xanthine

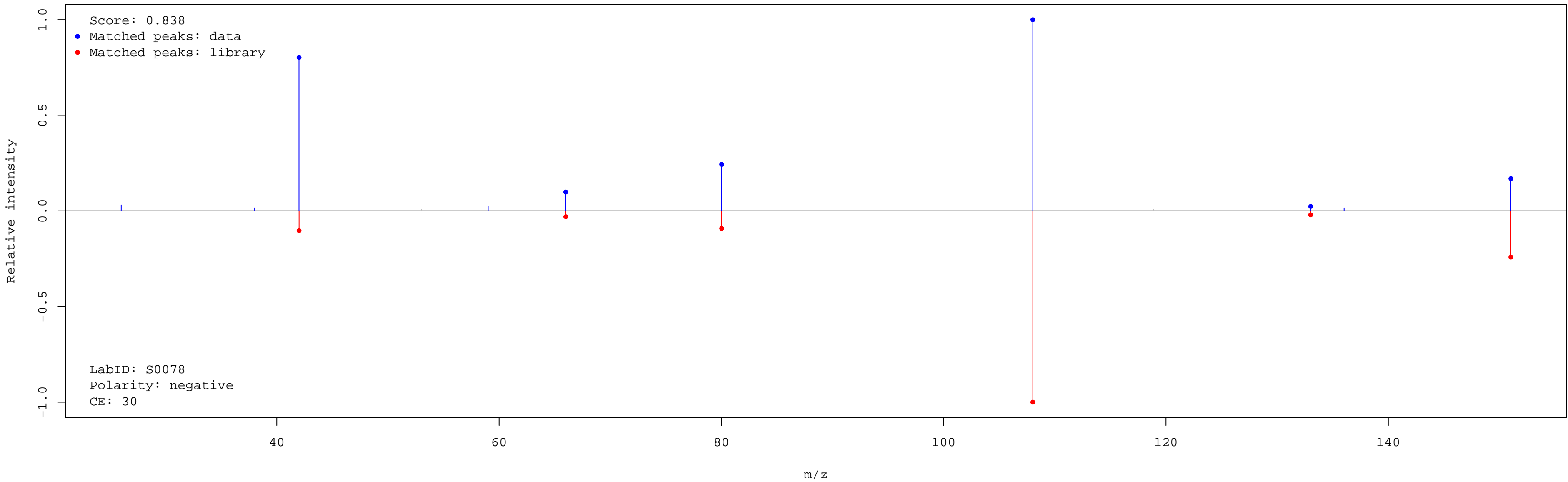

Supplement: Supplementary file 1 [file DataSheet1.ZIP › Supplementary table 1-10 and material 1-3/Material 3-Metlib-MSMS/NEG-Metlib-MSMS/Metlib-MSMS/M151T343_forward/0.838,Xanthine,(M-H)-.pdf]

# Xylitol

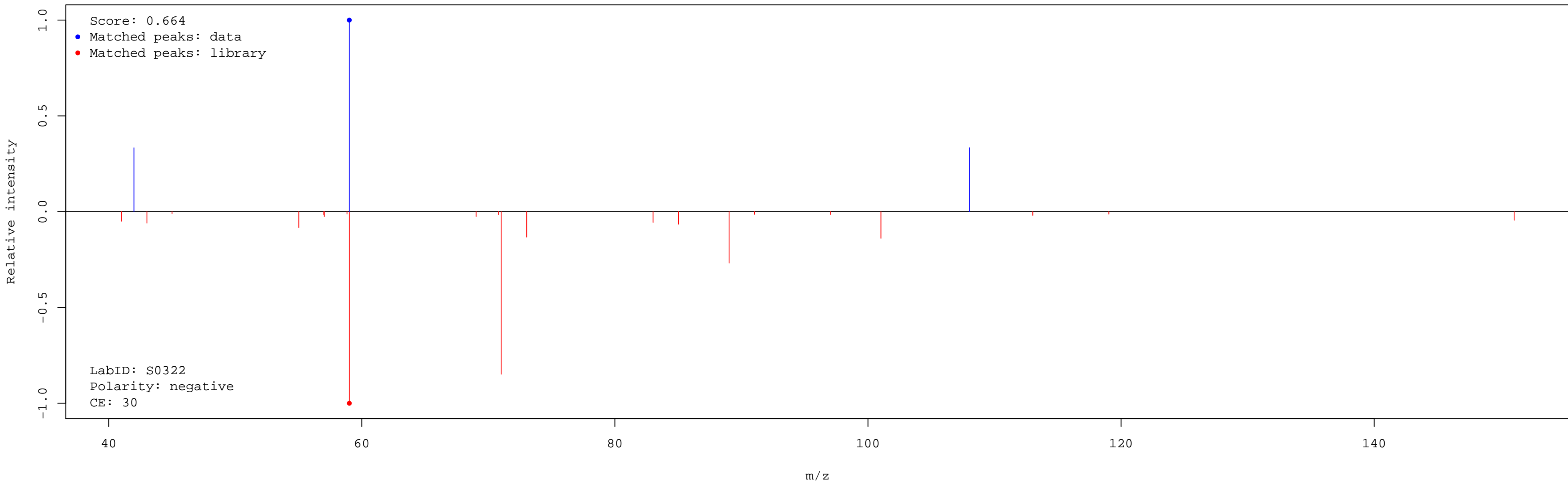

Supplement: Supplementary file 1 [file DataSheet1.ZIP › Supplementary table 1-10 and material 1-3/Material 3-Metlib-MSMS/NEG-Metlib-MSMS/Metlib-MSMS/M151T362_forward/0.664,Xylitol,(M-H)-.pdf]

# Glycerol

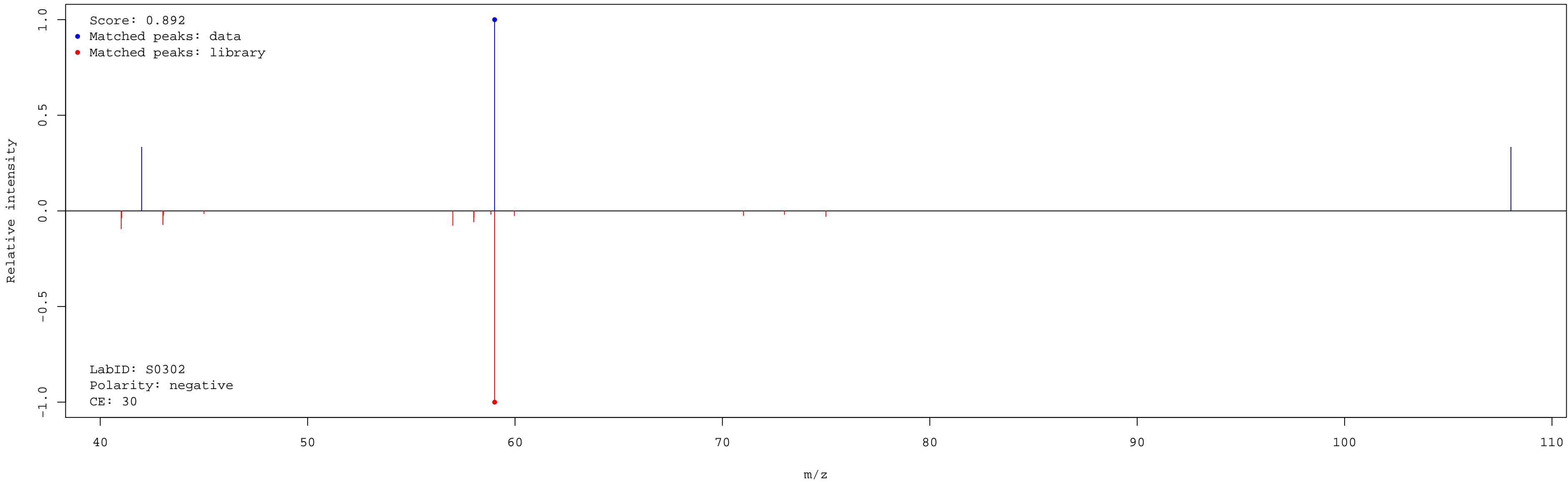

Supplement: Supplementary file 1 [file DataSheet1.ZIP › Supplementary table 1-10 and material 1-3/Material 3-Metlib-MSMS/NEG-Metlib-MSMS/Metlib-MSMS/M151T362_forward/0.892,Glycerol,(M+CH3COO)-.pdf]

L-Asparagine

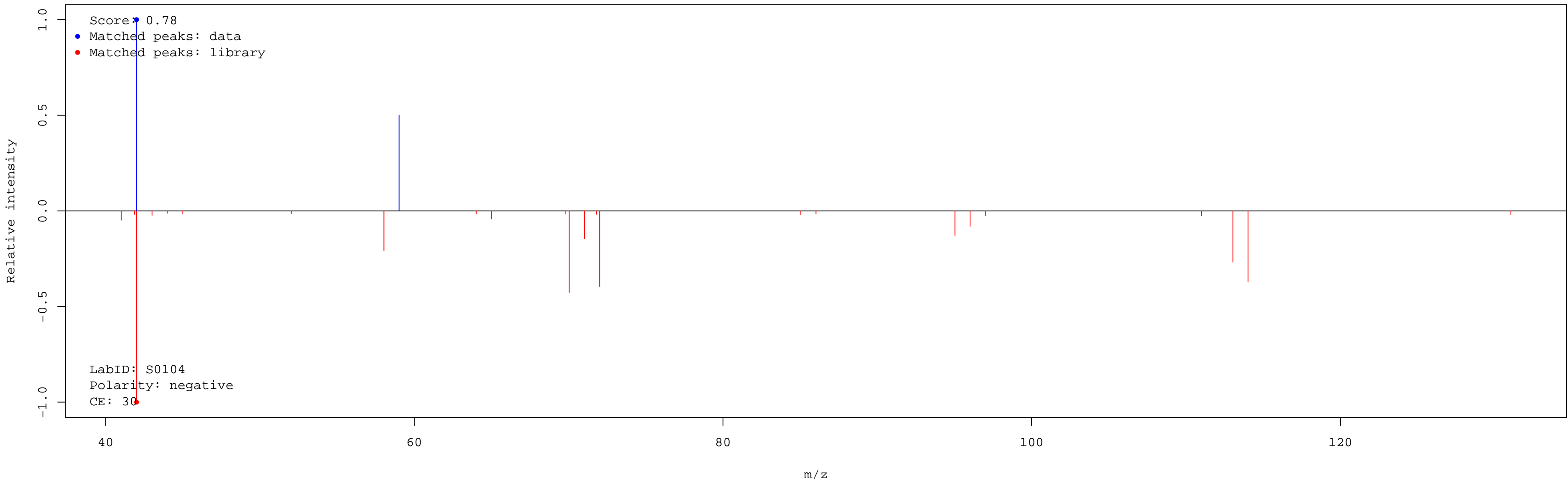

Supplement: Supplementary file 1 [file DataSheet1.ZIP › Supplementary table 1-10 and material 1-3/Material 3-Metlib-MSMS/NEG-Metlib-MSMS/Metlib-MSMS/M153T163_2_forward/0.78,L-Asparagine,(M+Na-2H)-.pdf]

Glycerol 3-phosphate

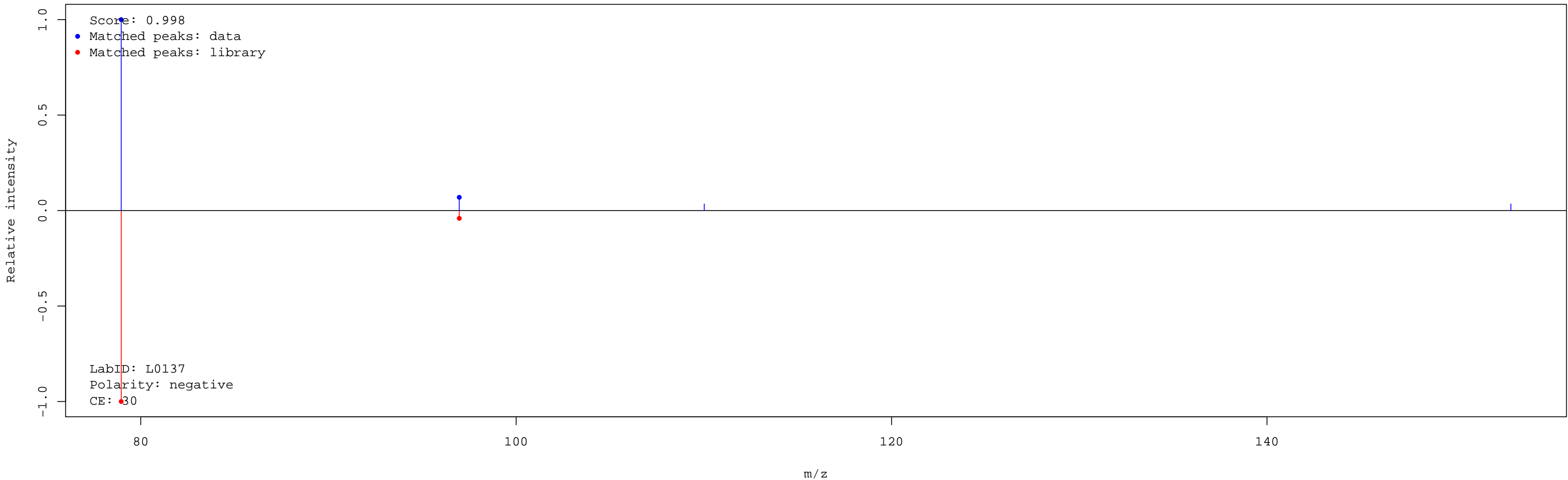

Supplement: Supplementary file 1 [file DataSheet1.ZIP › Supplementary table 1-10 and material 1-3/Material 3-Metlib-MSMS/NEG-Metlib-MSMS/Metlib-MSMS/M153T275_forward/0.998,Glycerol 3-phosphate,(M-H2O-H)-.pdf]
