# Supplementary figures and images for: Integrated Proteomics and Metabolomics Link Acne to the Action Mechanisms of Cryptotanshinone Intervention (part 1 of 2)
Source: Front Pharmacol. 2021 Sep 1;12:700696. doi: 10.3389/fphar.2021.700696 (PMC8440807; doi:10.3389/fphar.2021.700696)

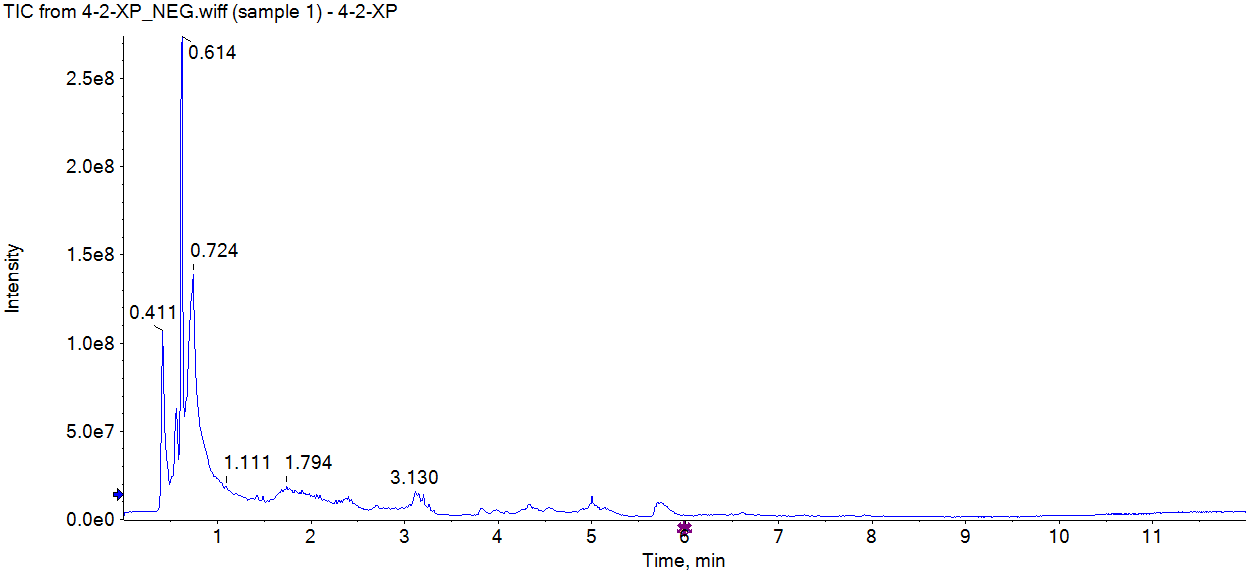

Supplement: Supplementary file 1 [file DataSheet1.ZIP › Supplementary table 1-10 and material 1-3/Material 2-QC samples and skin samples TIC.docx]

Acetoacetic acid

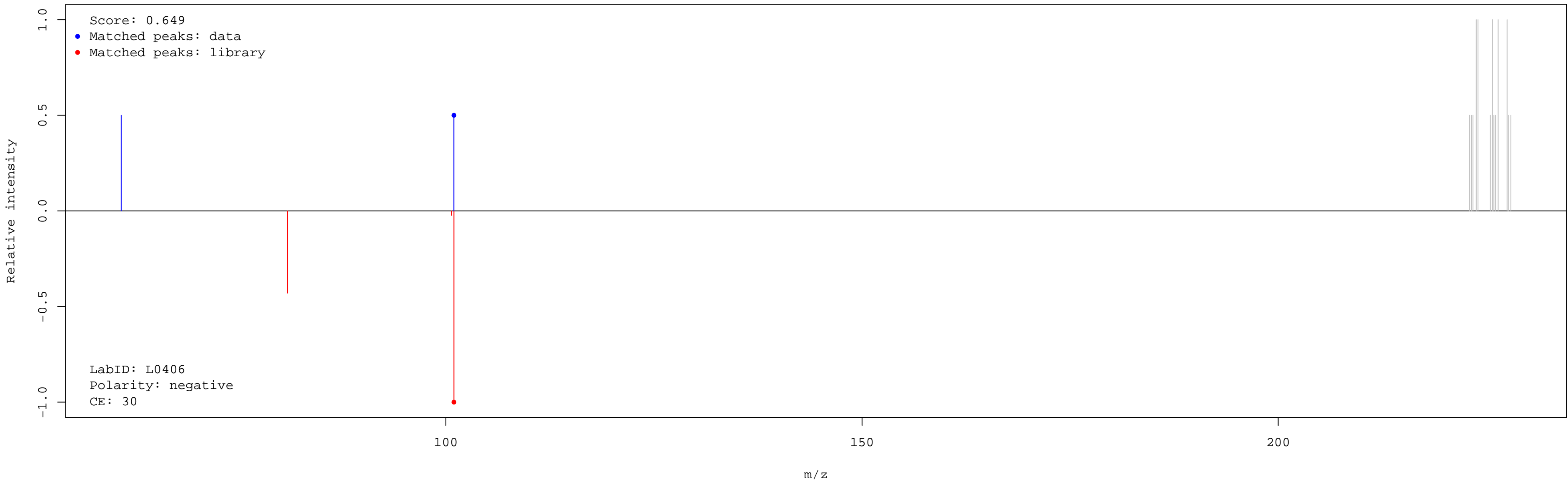

Supplement: Supplementary file 1 [file DataSheet1.ZIP › Supplementary table 1-10 and material 1-3/Material 3-Metlib-MSMS/NEG-Metlib-MSMS/Metlib-MSMS/M101T400_forward/0.649,Acetoacetic acid,(M-H)-.pdf]

Valeric acid

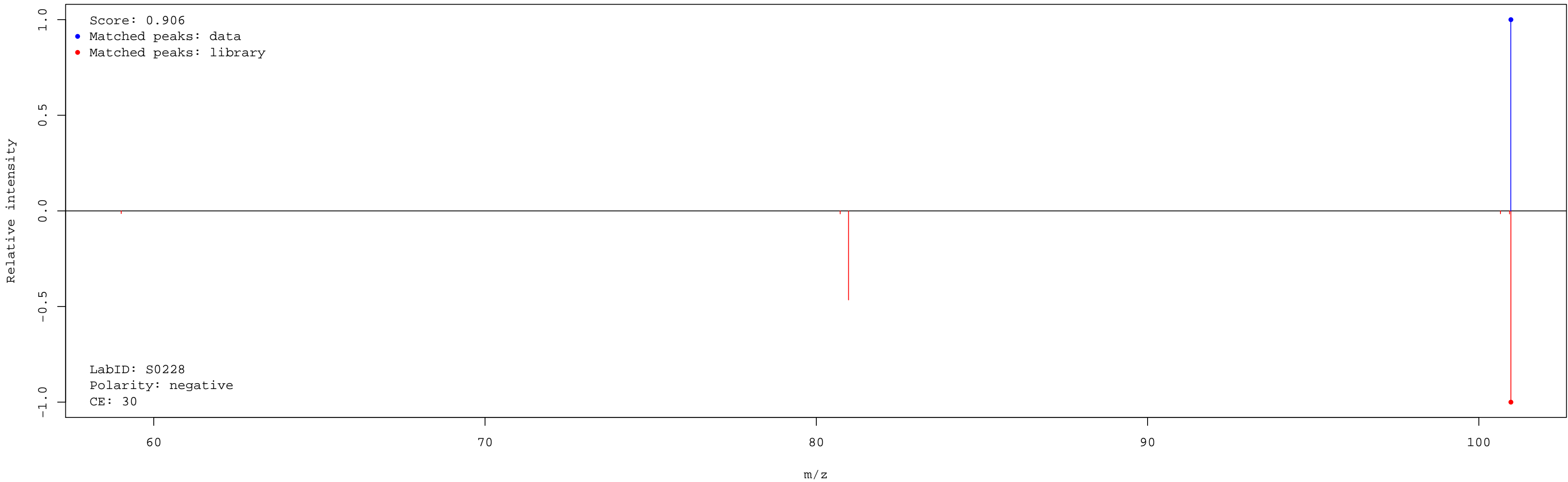

Supplement: Supplementary file 1 [file DataSheet1.ZIP › Supplementary table 1-10 and material 1-3/Material 3-Metlib-MSMS/NEG-Metlib-MSMS/Metlib-MSMS/M101T60_forward/0.906,Valeric acid,(M-H)-.pdf]

Valeric acid

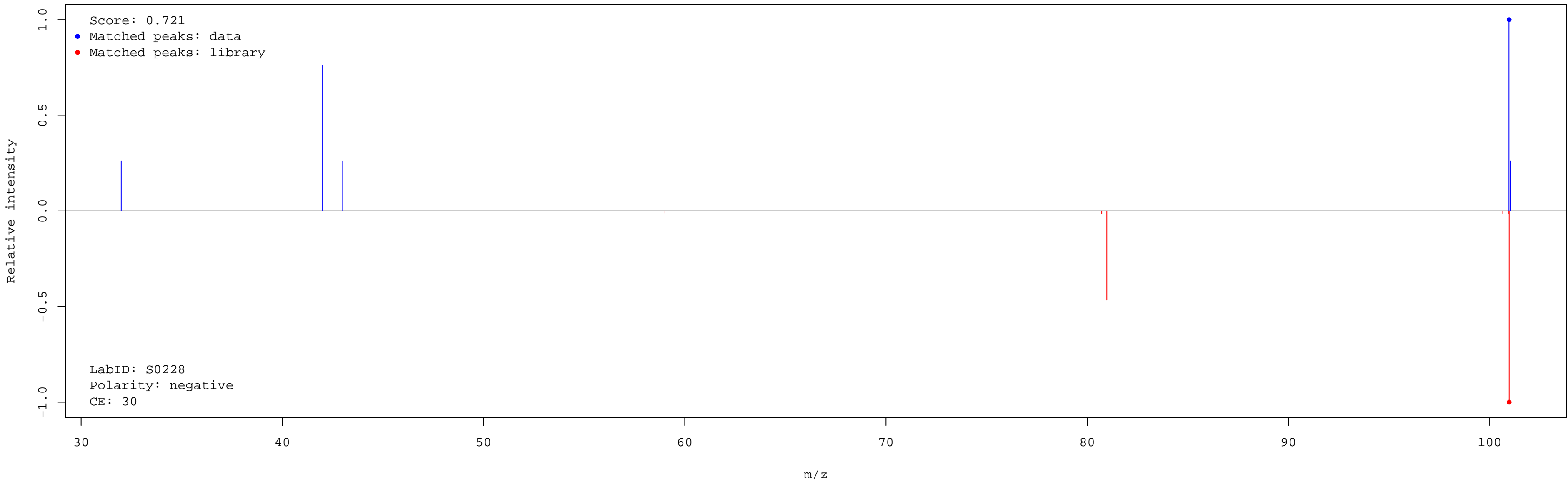

Supplement: Supplementary file 1 [file DataSheet1.ZIP › Supplementary table 1-10 and material 1-3/Material 3-Metlib-MSMS/NEG-Metlib-MSMS/Metlib-MSMS/M101T91_forward/0.721,Valeric acid,(M-H)-.pdf]

# L-Serine

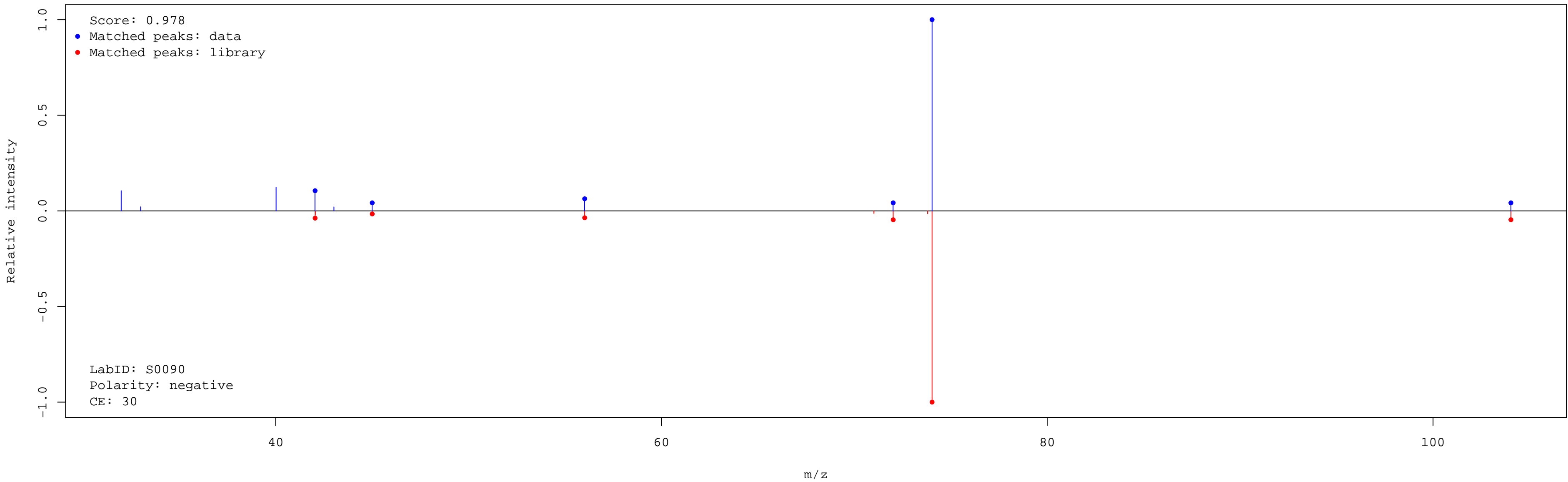

Supplement: Supplementary file 1 [file DataSheet1.ZIP › Supplementary table 1-10 and material 1-3/Material 3-Metlib-MSMS/NEG-Metlib-MSMS/Metlib-MSMS/M104T301_forward/0.978,L-Serine,(M-H)-.pdf]

# DL-Serine

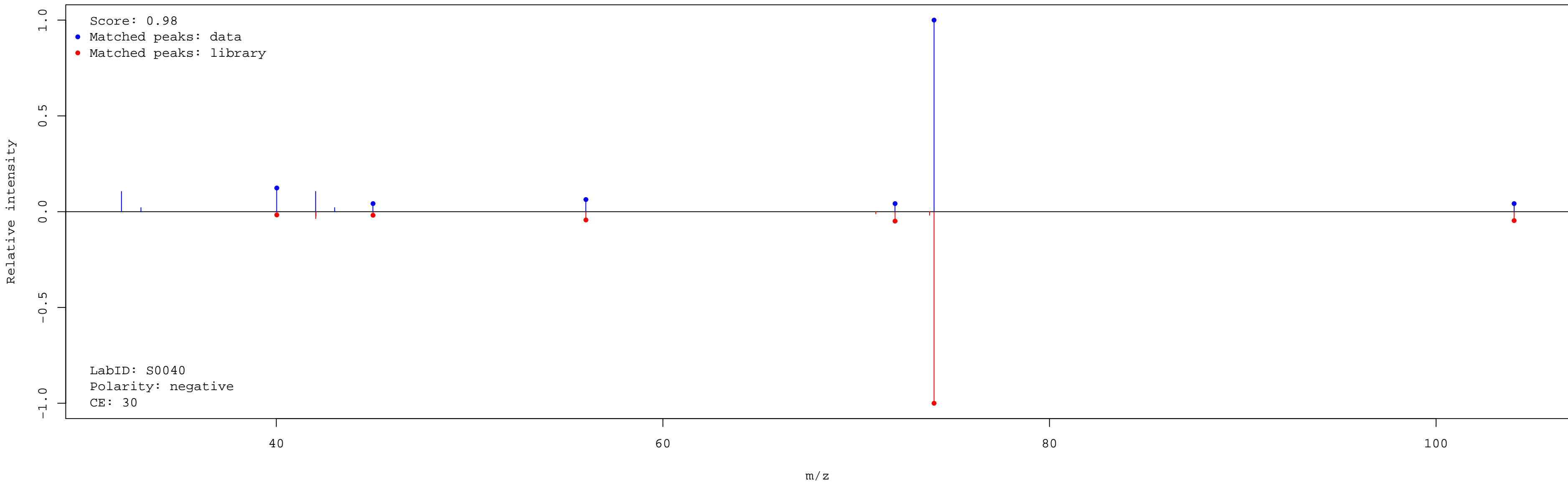

Supplement: Supplementary file 1 [file DataSheet1.ZIP › Supplementary table 1-10 and material 1-3/Material 3-Metlib-MSMS/NEG-Metlib-MSMS/Metlib-MSMS/M104T301_forward/0.98,DL-Serine,(M-H)-.pdf]

# L-Serine

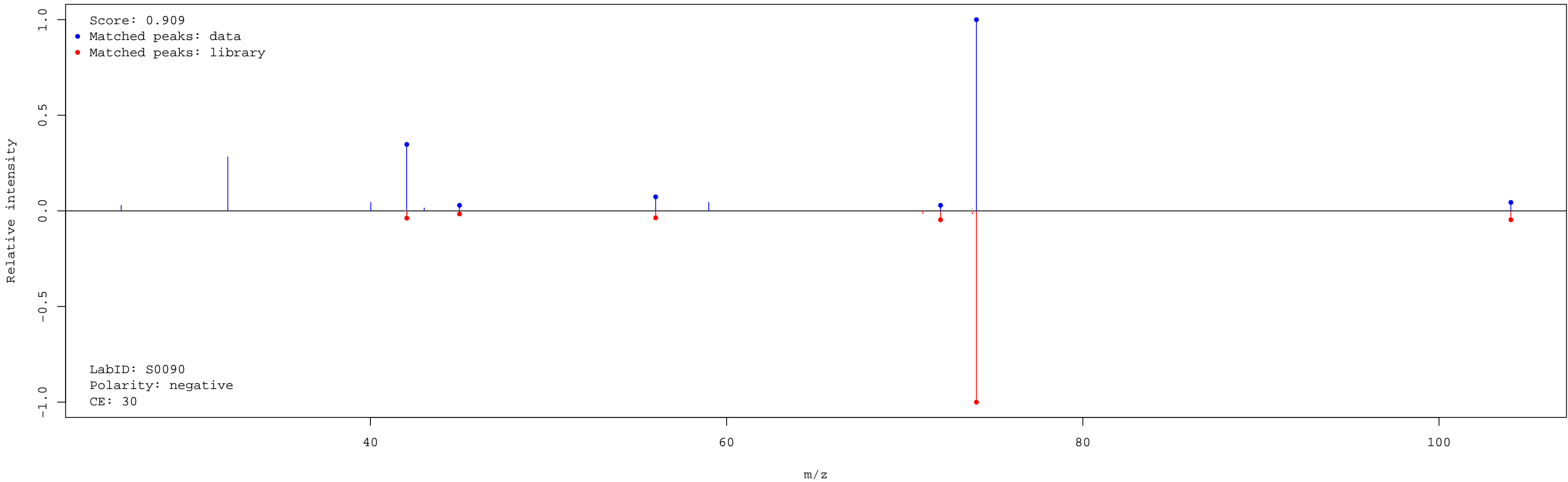

Supplement: Supplementary file 1 [file DataSheet1.ZIP › Supplementary table 1-10 and material 1-3/Material 3-Metlib-MSMS/NEG-Metlib-MSMS/Metlib-MSMS/M104T380_forward/0.909,L-Serine,(M-H)-.pdf]

# DL-Serine

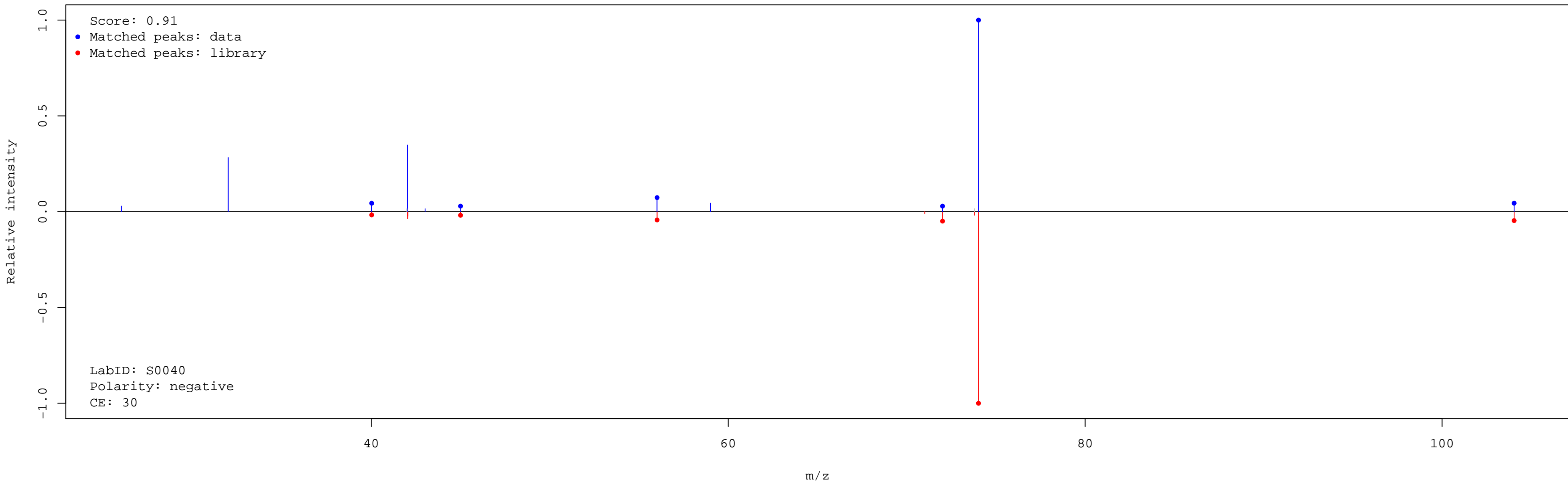

Supplement: Supplementary file 1 [file DataSheet1.ZIP › Supplementary table 1-10 and material 1-3/Material 3-Metlib-MSMS/NEG-Metlib-MSMS/Metlib-MSMS/M104T380_forward/0.91,DL-Serine,(M-H)-.pdf]

# L-Serine

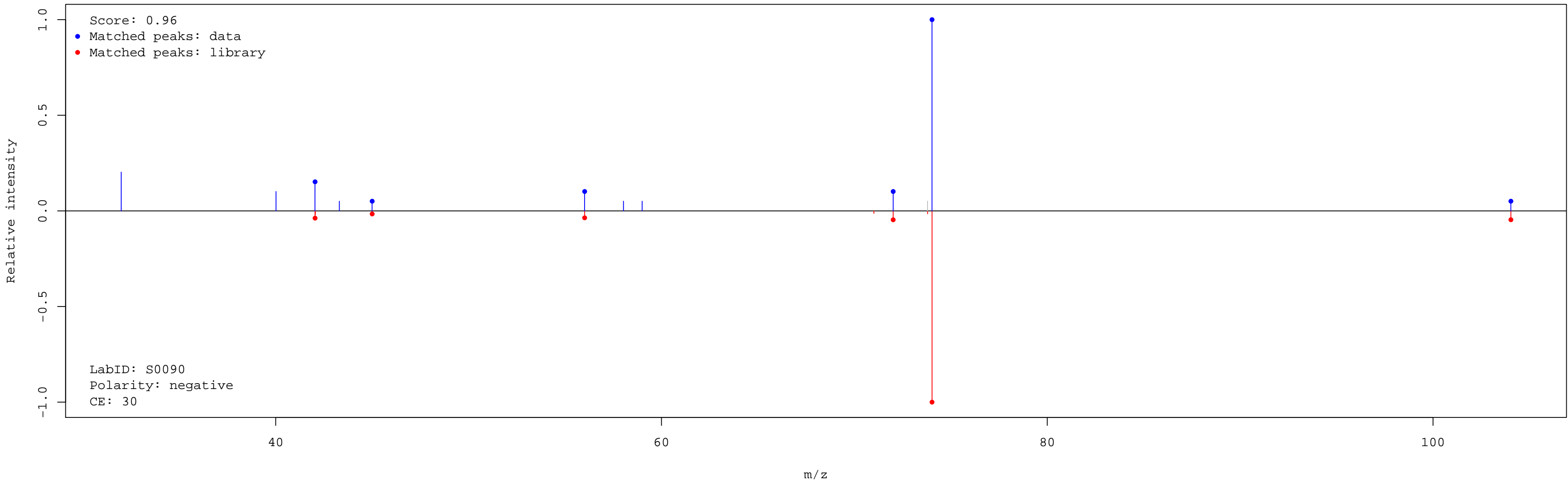

Supplement: Supplementary file 1 [file DataSheet1.ZIP › Supplementary table 1-10 and material 1-3/Material 3-Metlib-MSMS/NEG-Metlib-MSMS/Metlib-MSMS/M104T426_forward/0.96,L-Serine,(M-H)-.pdf]

# DL-Serine

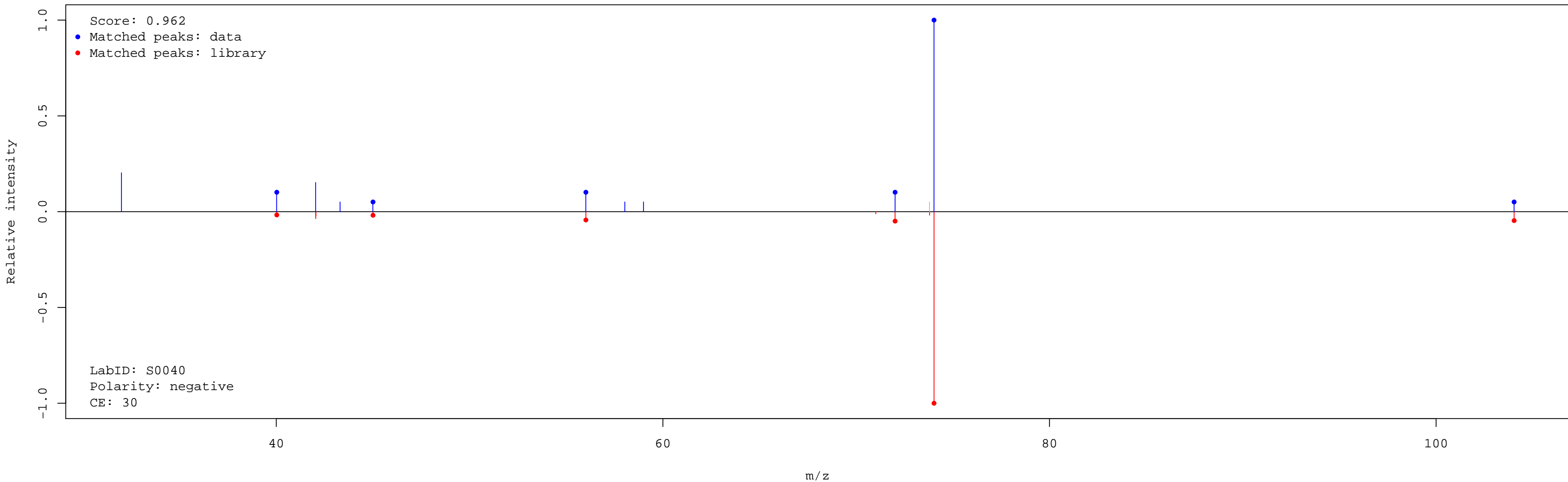

Supplement: Supplementary file 1 [file DataSheet1.ZIP › Supplementary table 1-10 and material 1-3/Material 3-Metlib-MSMS/NEG-Metlib-MSMS/Metlib-MSMS/M104T426_forward/0.962,DL-Serine,(M-H)-.pdf]

# Glyceric acid

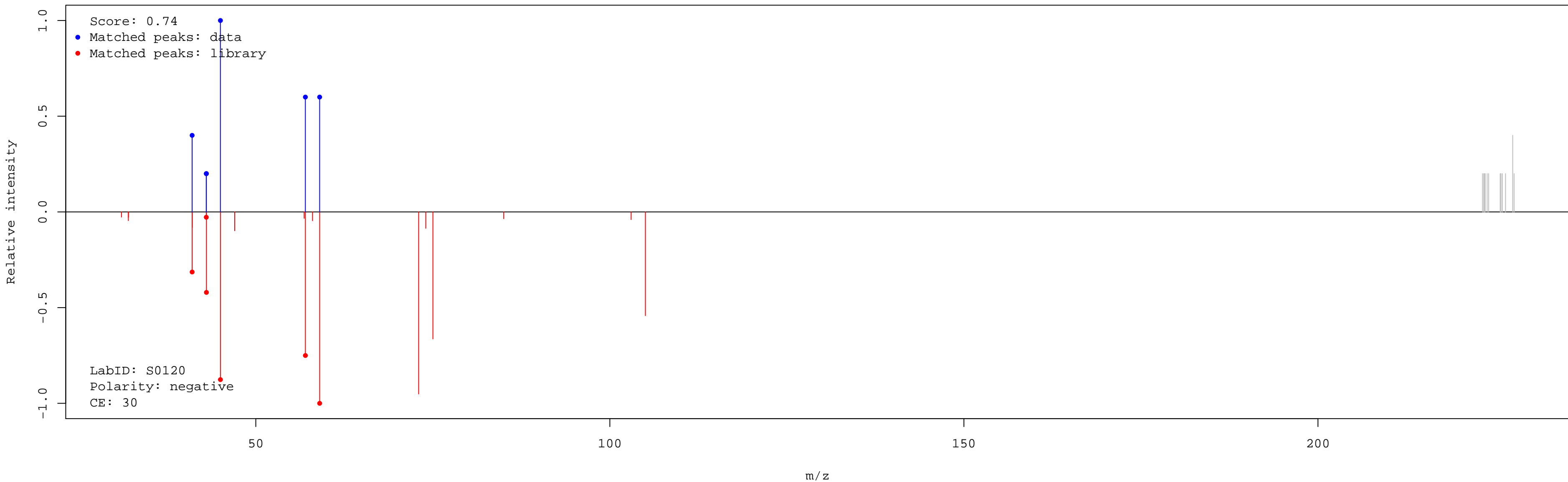

Supplement: Supplementary file 1 [file DataSheet1.ZIP › Supplementary table 1-10 and material 1-3/Material 3-Metlib-MSMS/NEG-Metlib-MSMS/Metlib-MSMS/M105T302_forward/0.74,Glyceric acid,(M-H)-.pdf]

# Glyceric acid

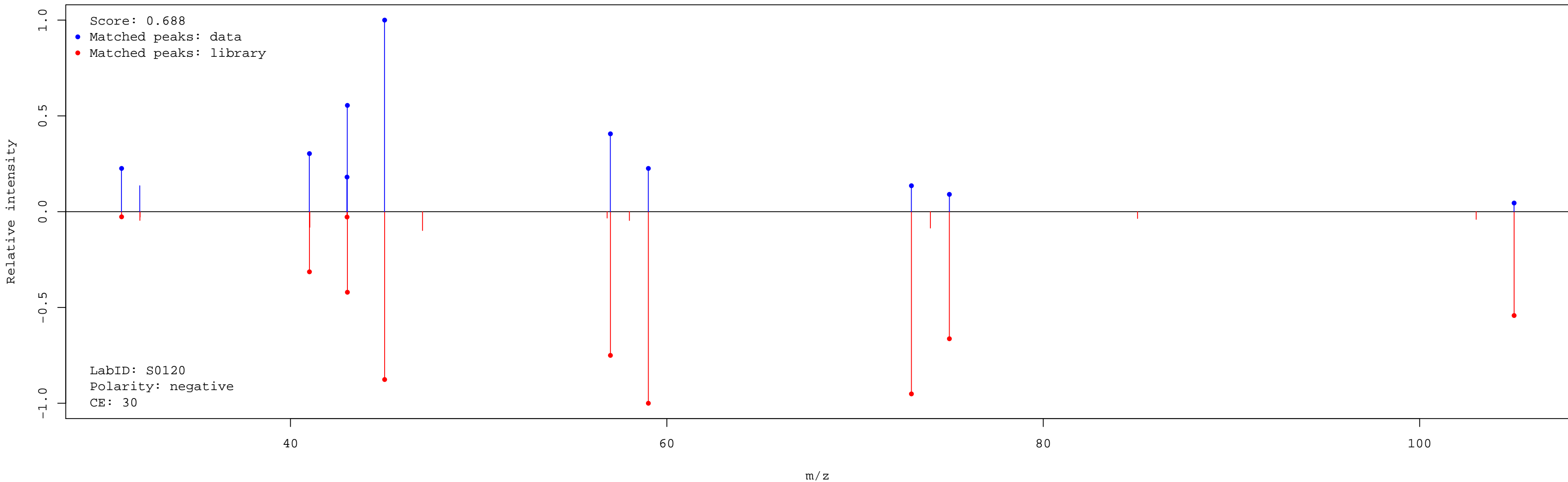

Supplement: Supplementary file 1 [file DataSheet1.ZIP › Supplementary table 1-10 and material 1-3/Material 3-Metlib-MSMS/NEG-Metlib-MSMS/Metlib-MSMS/M105T319_forward/0.688,Glyceric acid,(M-H)-.pdf]

# Cytosine

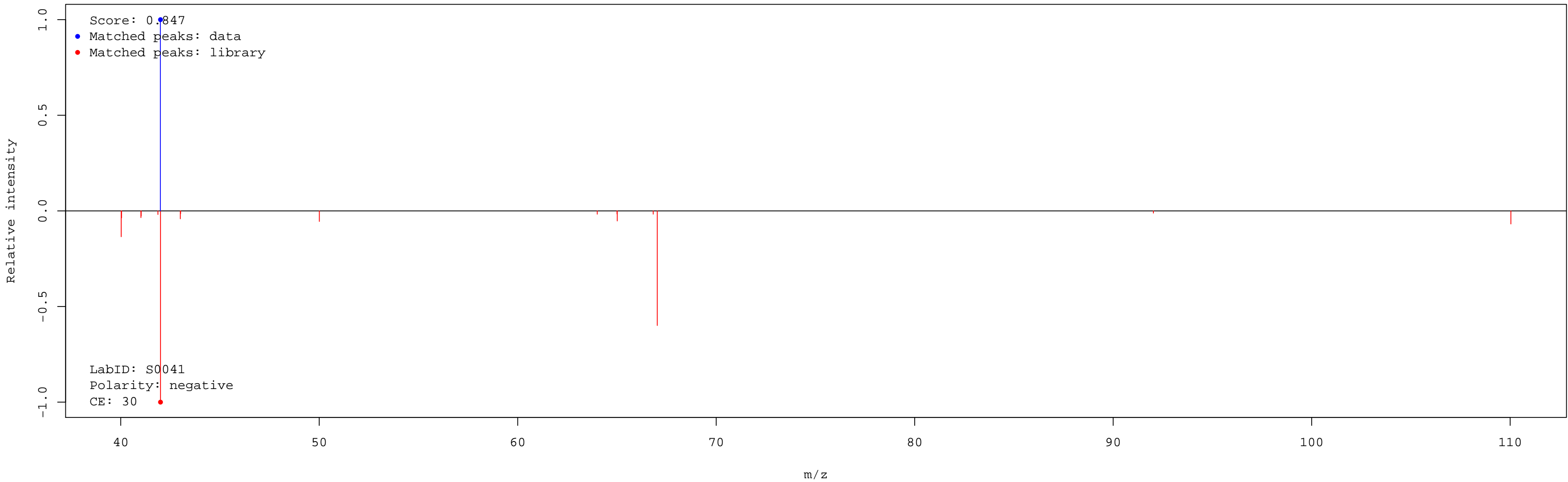

Supplement: Supplementary file 1 [file DataSheet1.ZIP › Supplementary table 1-10 and material 1-3/Material 3-Metlib-MSMS/NEG-Metlib-MSMS/Metlib-MSMS/M110T196_forward/0.847,Cytosine,(M-H)-.pdf]

# Uracil

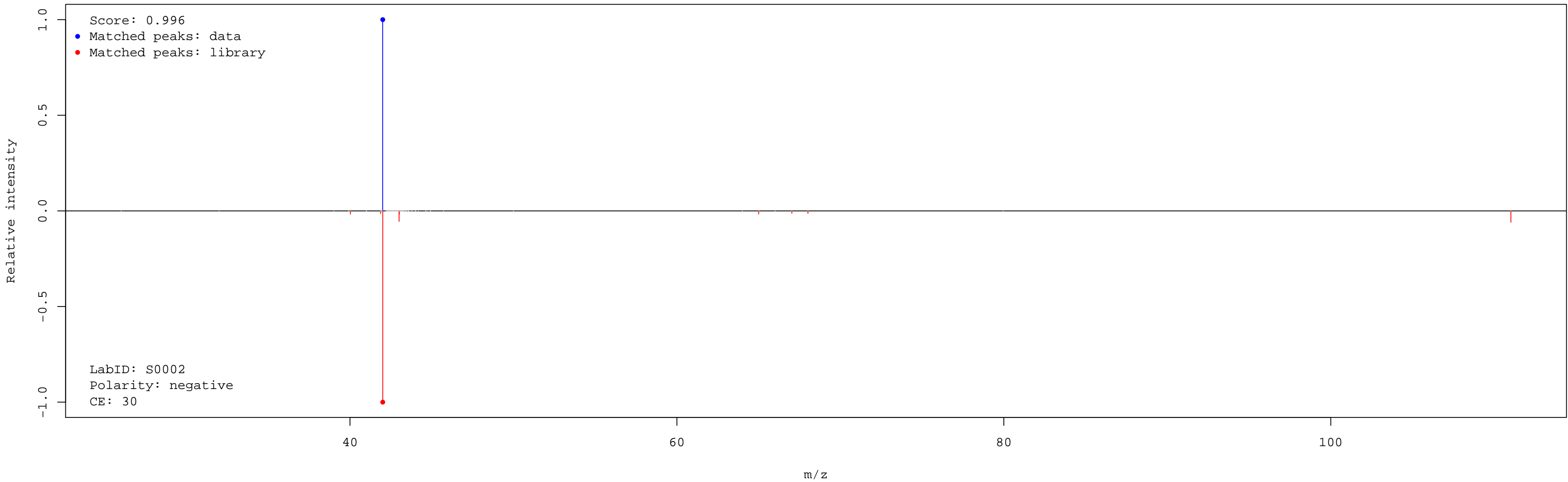

Supplement: Supplementary file 1 [file DataSheet1.ZIP › Supplementary table 1-10 and material 1-3/Material 3-Metlib-MSMS/NEG-Metlib-MSMS/Metlib-MSMS/M111T103_2_forward/0.996,Uracil,(M-H)-.pdf]

# Uracil

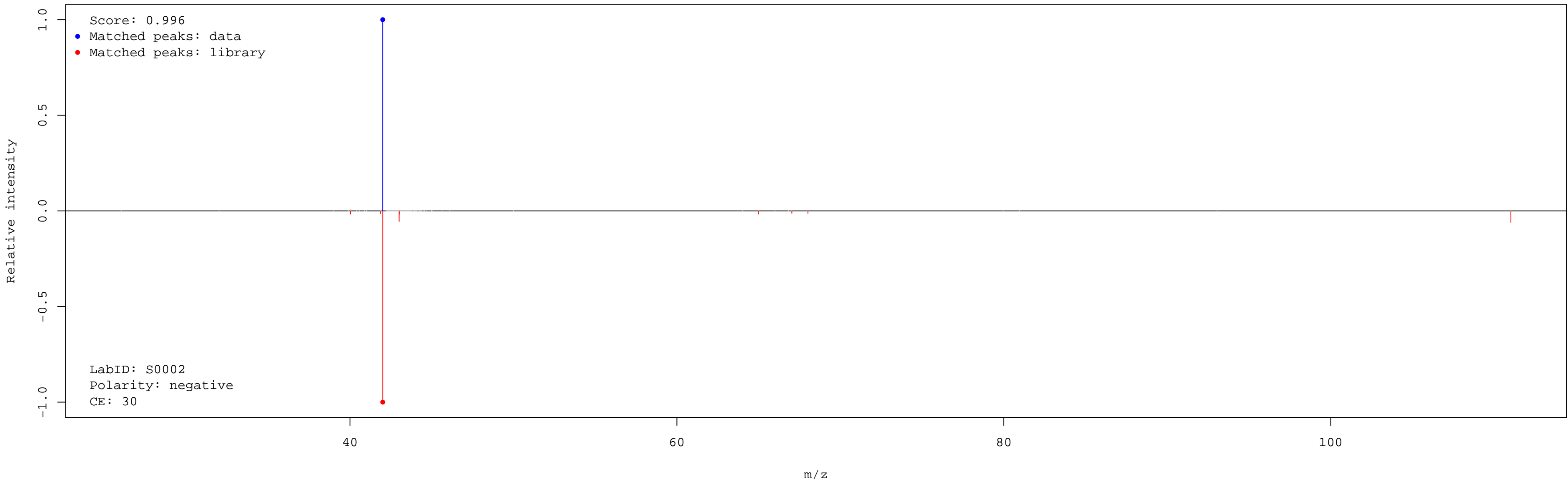

Supplement: Supplementary file 1 [file DataSheet1.ZIP › Supplementary table 1-10 and material 1-3/Material 3-Metlib-MSMS/NEG-Metlib-MSMS/Metlib-MSMS/M111T163_forward/0.996,Uracil,(M-H)-.pdf]

# Uracil

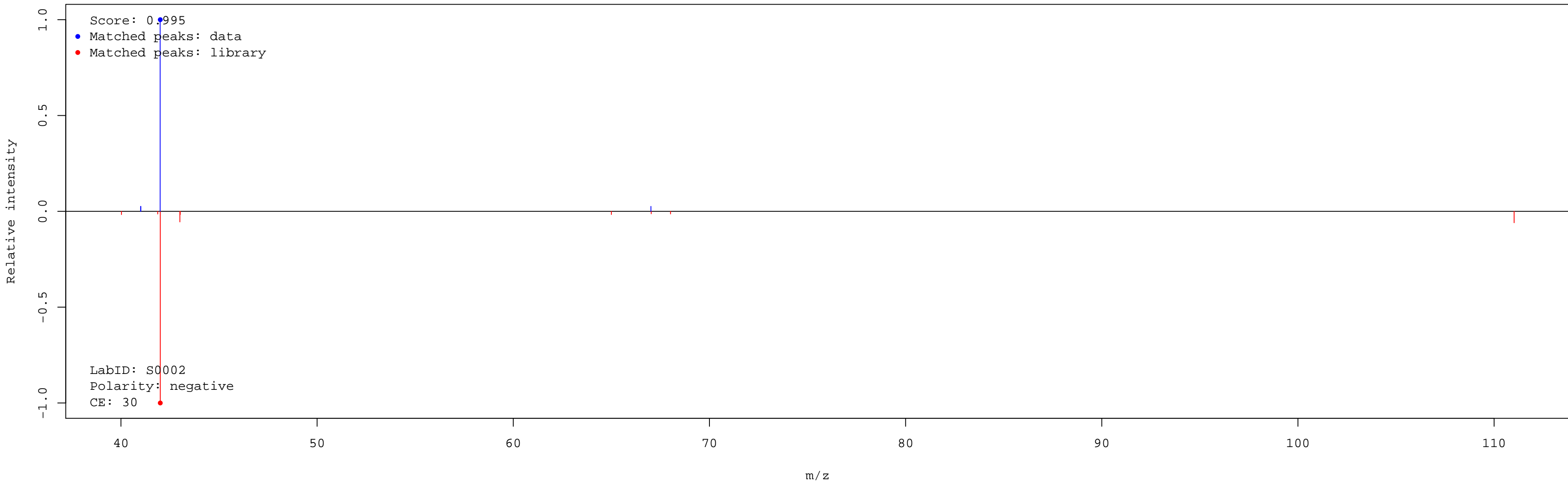

Supplement: Supplementary file 1 [file DataSheet1.ZIP › Supplementary table 1-10 and material 1-3/Material 3-Metlib-MSMS/NEG-Metlib-MSMS/Metlib-MSMS/M111T234_forward/0.995,Uracil,(M-H)-.pdf]

# Uracil

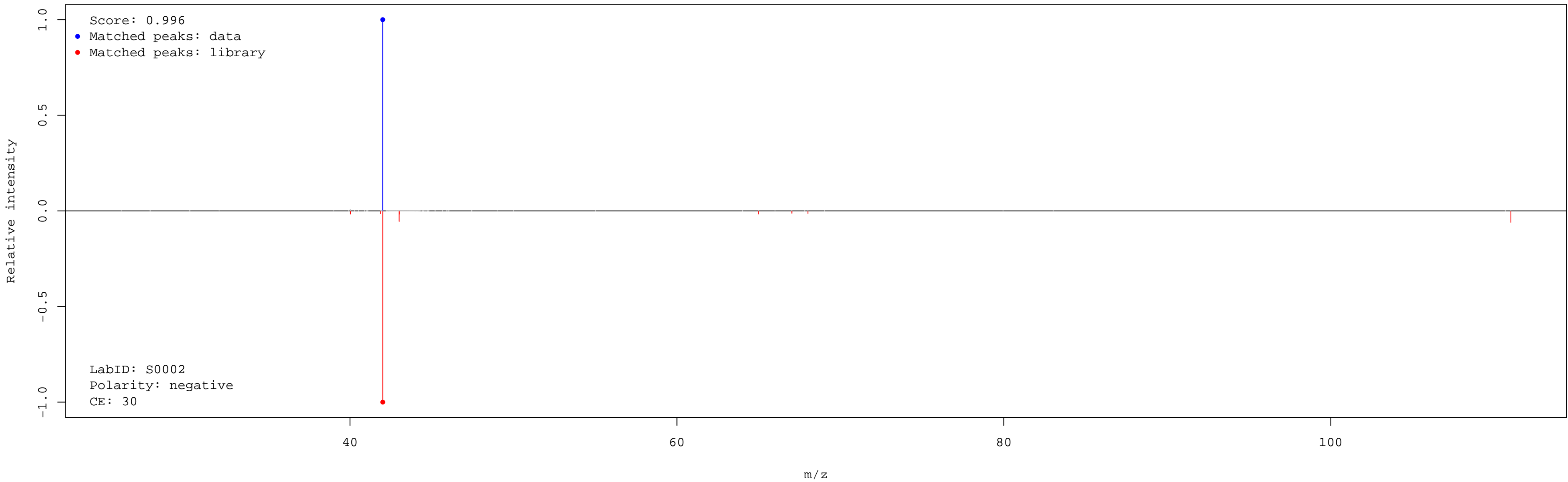

Supplement: Supplementary file 1 [file DataSheet1.ZIP › Supplementary table 1-10 and material 1-3/Material 3-Metlib-MSMS/NEG-Metlib-MSMS/Metlib-MSMS/M111T87_2_forward/0.996,Uracil,(M-H)-.pdf]

# L-Proline

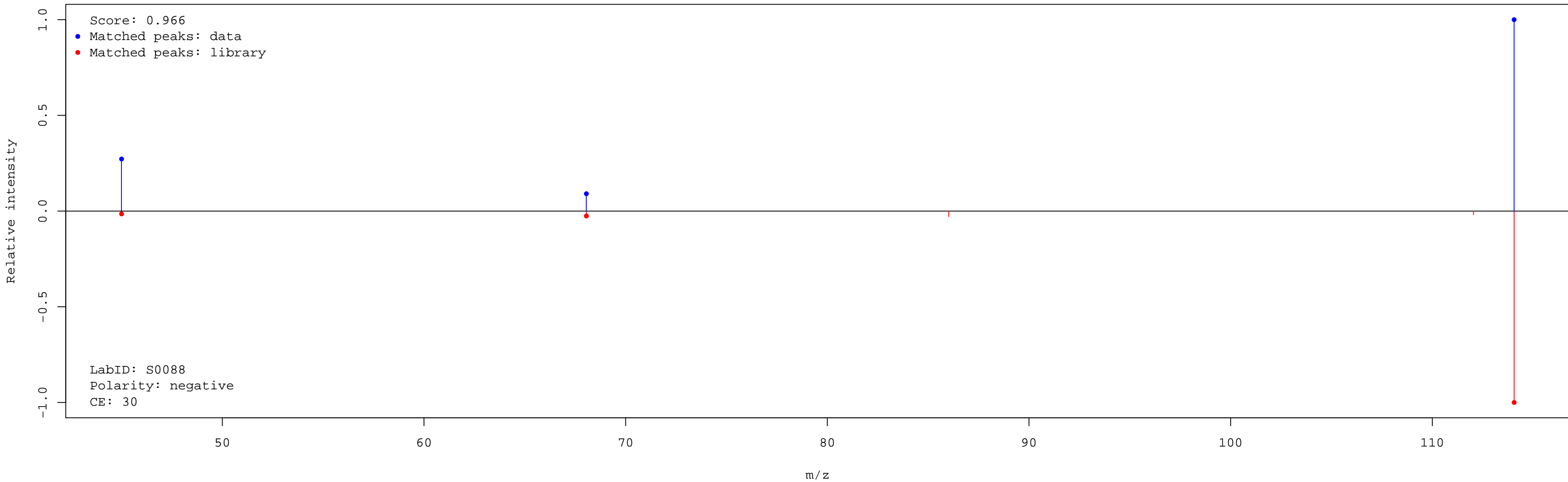

Supplement: Supplementary file 1 [file DataSheet1.ZIP › Supplementary table 1-10 and material 1-3/Material 3-Metlib-MSMS/NEG-Metlib-MSMS/Metlib-MSMS/M114T346_forward/0.966,L-Proline,(M-H)-.pdf]

# D-Proline

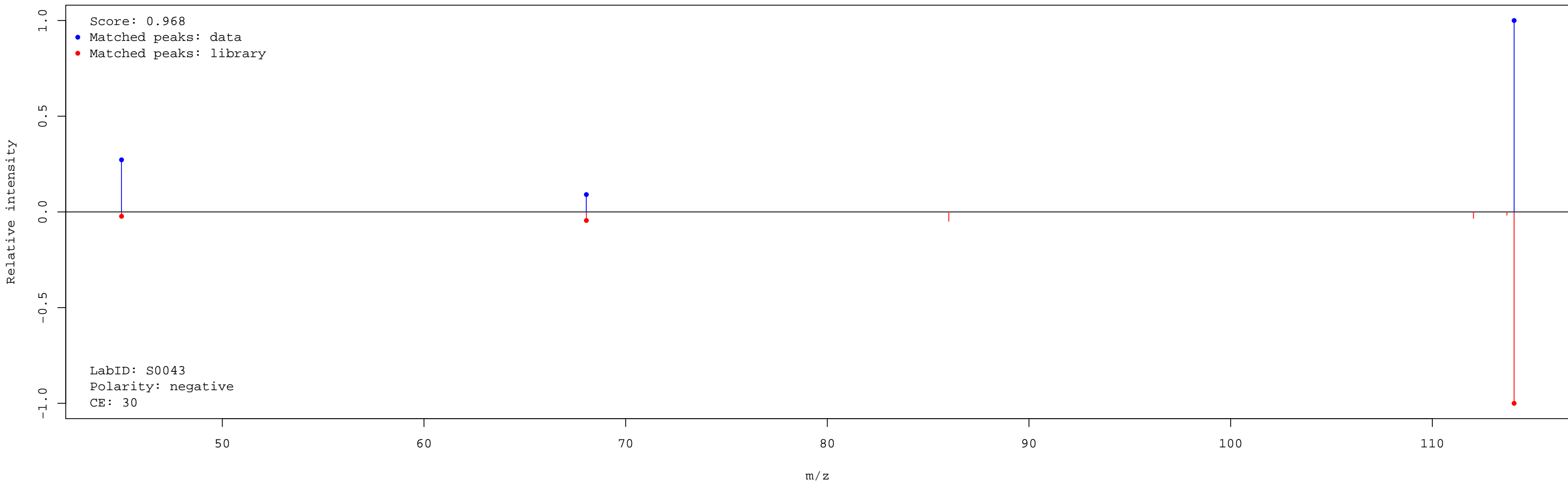

Supplement: Supplementary file 1 [file DataSheet1.ZIP › Supplementary table 1-10 and material 1-3/Material 3-Metlib-MSMS/NEG-Metlib-MSMS/Metlib-MSMS/M114T346_forward/0.968,D-Proline,(M-H)-.pdf]

# L-Proline

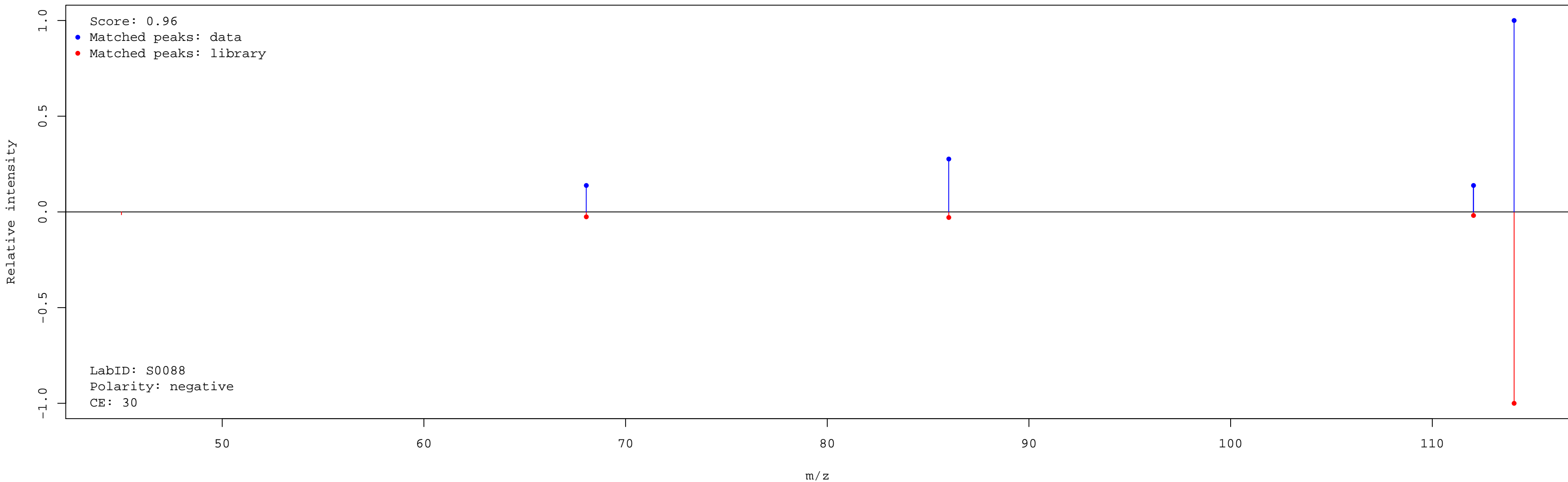

Supplement: Supplementary file 1 [file DataSheet1.ZIP › Supplementary table 1-10 and material 1-3/Material 3-Metlib-MSMS/NEG-Metlib-MSMS/Metlib-MSMS/M114T368_forward/0.96,L-Proline,(M-H)-.pdf]

# D-Proline

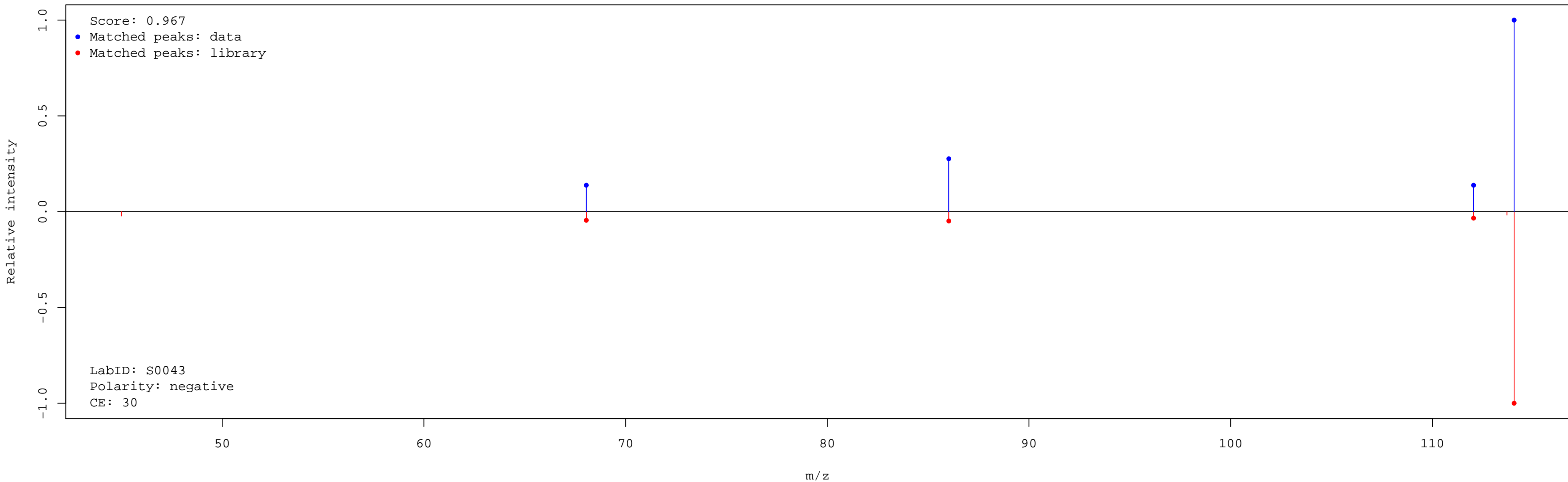

Supplement: Supplementary file 1 [file DataSheet1.ZIP › Supplementary table 1-10 and material 1-3/Material 3-Metlib-MSMS/NEG-Metlib-MSMS/Metlib-MSMS/M114T368_forward/0.967,D-Proline,(M-H)-.pdf]

# 5-Aminopentanoic acid

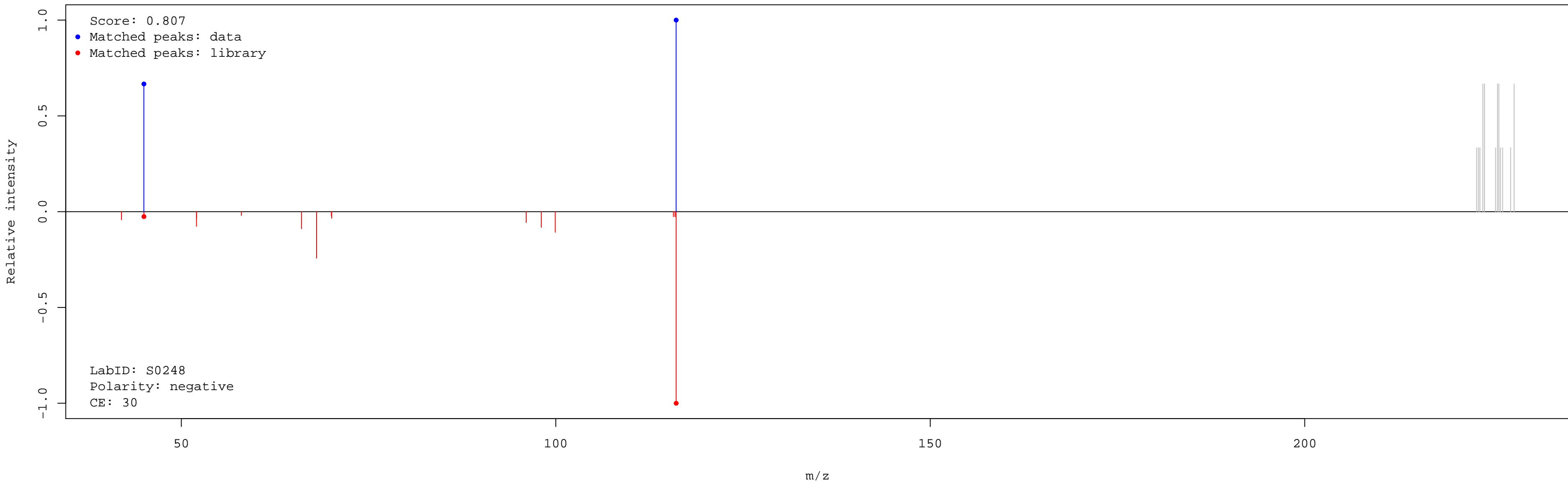

Supplement: Supplementary file 1 [file DataSheet1.ZIP › Supplementary table 1-10 and material 1-3/Material 3-Metlib-MSMS/NEG-Metlib-MSMS/Metlib-MSMS/M116T214_forward/0.807,5-Aminopentanoic acid,(M-H)-.pdf]

# L-Valine

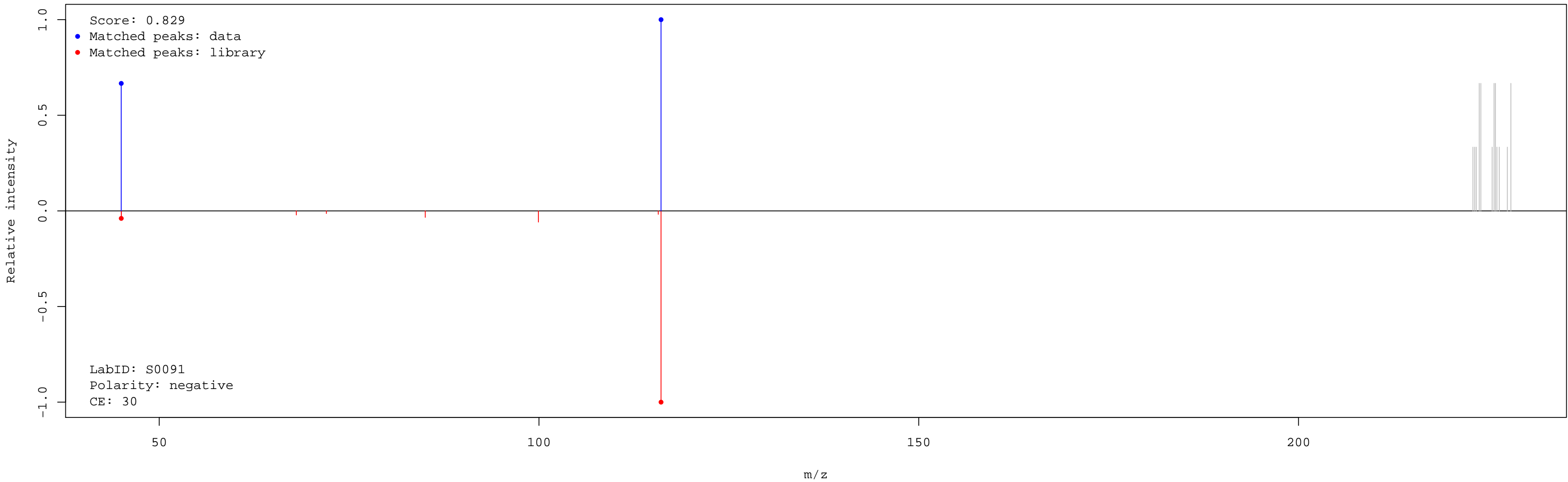

Supplement: Supplementary file 1 [file DataSheet1.ZIP › Supplementary table 1-10 and material 1-3/Material 3-Metlib-MSMS/NEG-Metlib-MSMS/Metlib-MSMS/M116T214_forward/0.829,L-Valine,(M-H)-.pdf]

# Indole

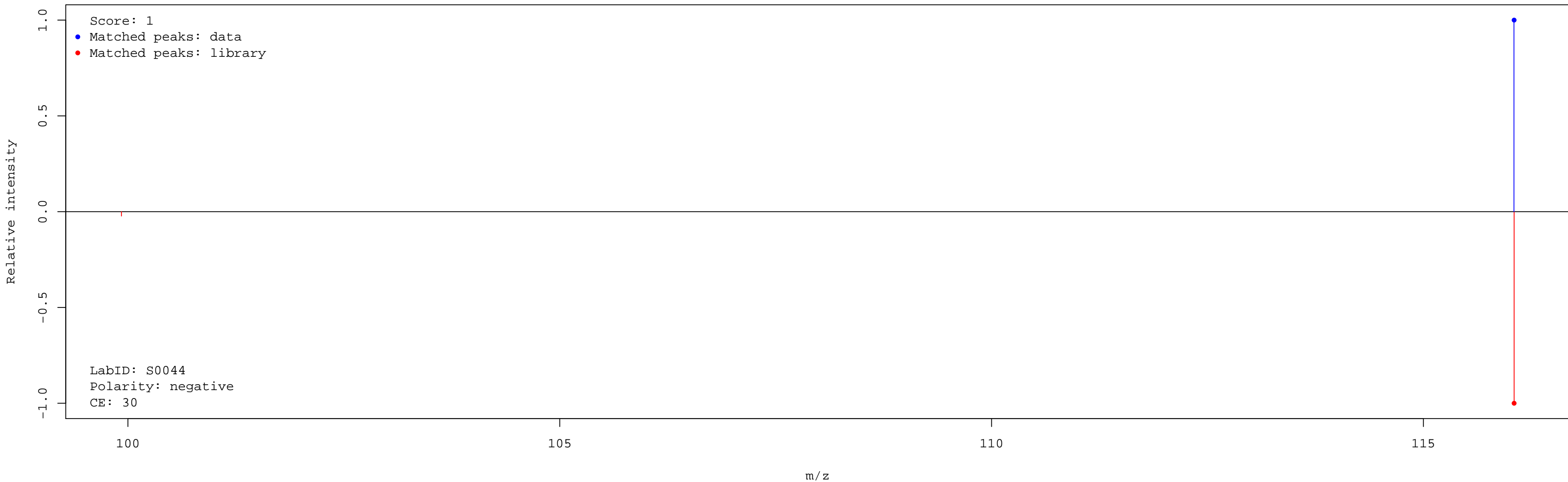

Supplement: Supplementary file 1 [file DataSheet1.ZIP › Supplementary table 1-10 and material 1-3/Material 3-Metlib-MSMS/NEG-Metlib-MSMS/Metlib-MSMS/M116T258_forward/1,Indole,(M-H)-.pdf]

# 5-Aminopentanoic acid

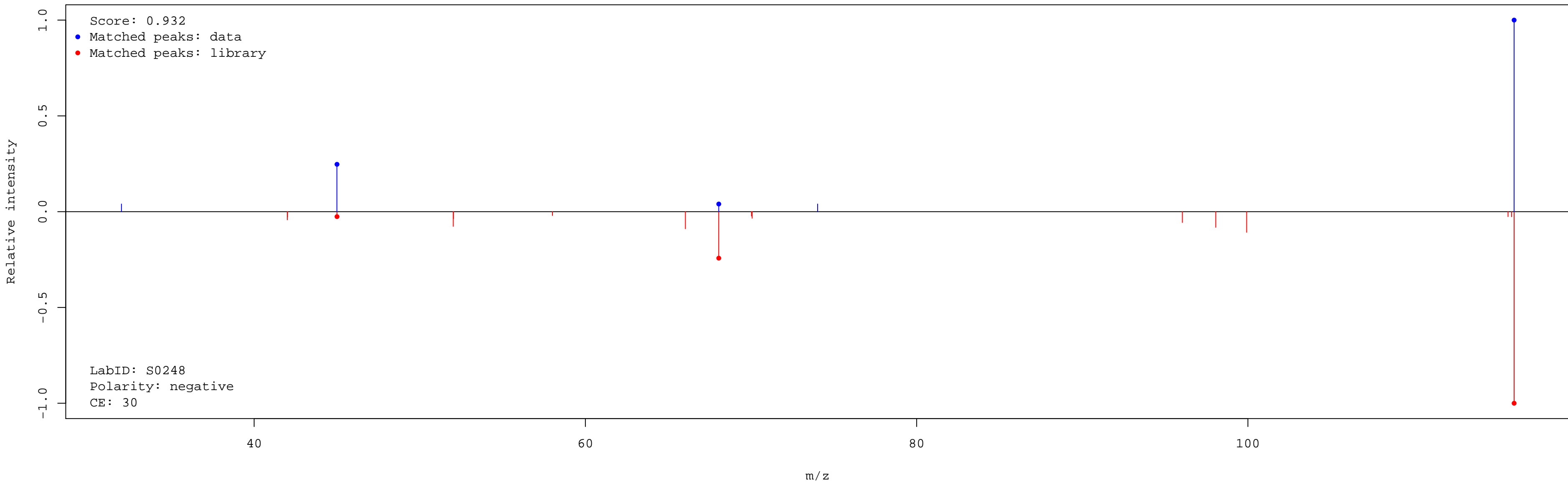

Supplement: Supplementary file 1 [file DataSheet1.ZIP › Supplementary table 1-10 and material 1-3/Material 3-Metlib-MSMS/NEG-Metlib-MSMS/Metlib-MSMS/M116T346_forward/0.932,5-Aminopentanoic acid,(M-H)-.pdf]

# L-Valine

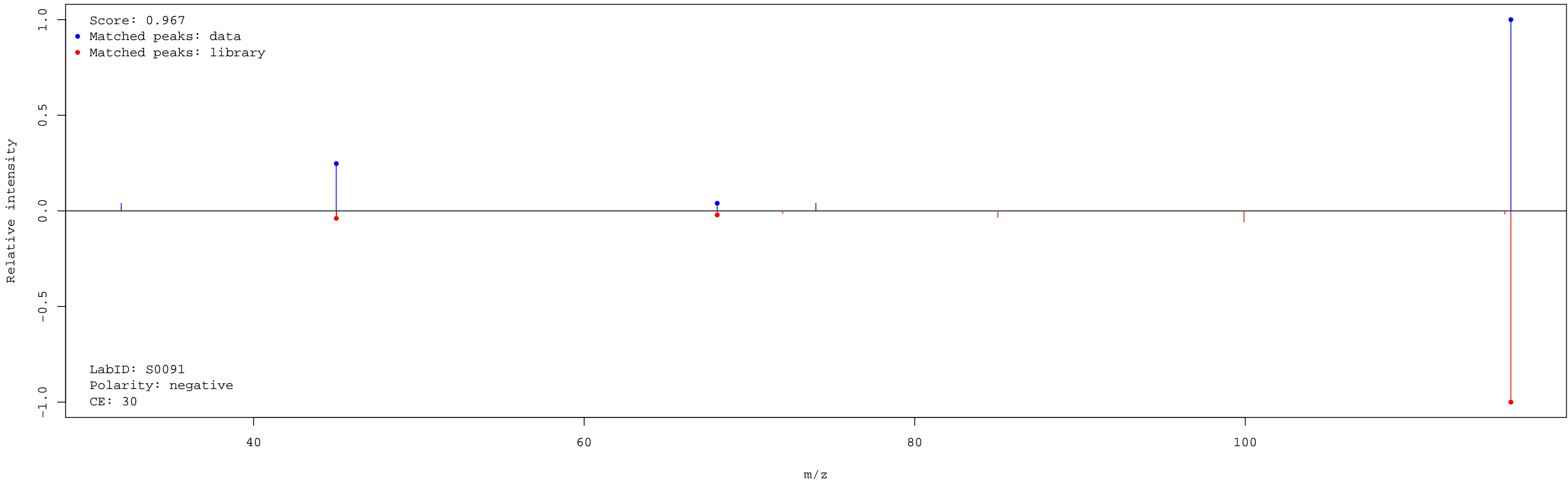

Supplement: Supplementary file 1 [file DataSheet1.ZIP › Supplementary table 1-10 and material 1-3/Material 3-Metlib-MSMS/NEG-Metlib-MSMS/Metlib-MSMS/M116T346_forward/0.967,L-Valine,(M-H)-.pdf]

# Indole

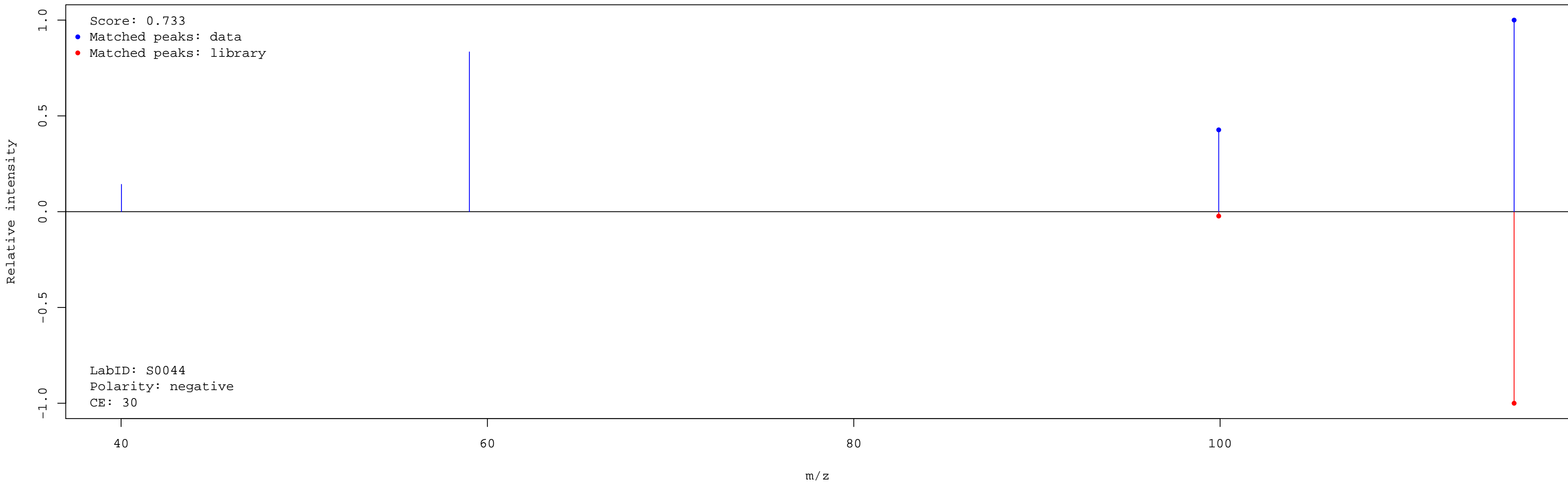

Supplement: Supplementary file 1 [file DataSheet1.ZIP › Supplementary table 1-10 and material 1-3/Material 3-Metlib-MSMS/NEG-Metlib-MSMS/Metlib-MSMS/M116T87_forward/0.733,Indole,(M-H)-.pdf]

# Methylmalonic acid

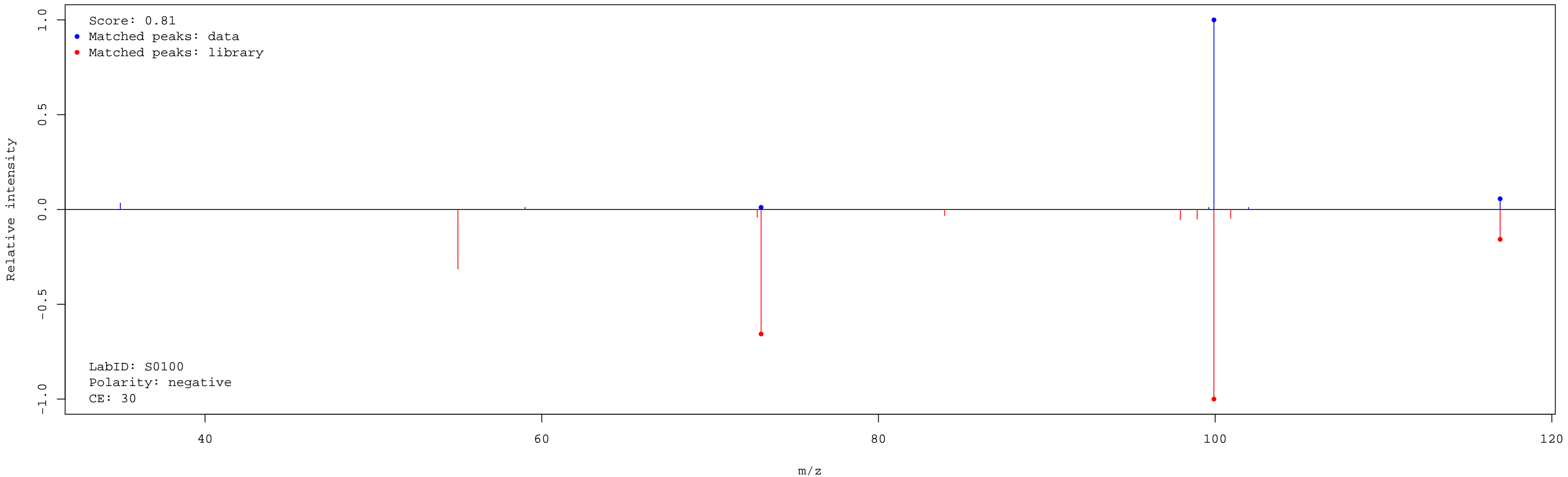

Supplement: Supplementary file 1 [file DataSheet1.ZIP › Supplementary table 1-10 and material 1-3/Material 3-Metlib-MSMS/NEG-Metlib-MSMS/Metlib-MSMS/M117T117_forward/0.81,Methylmalonic acid,(M-H)-.pdf]

# Methylmalonic acid

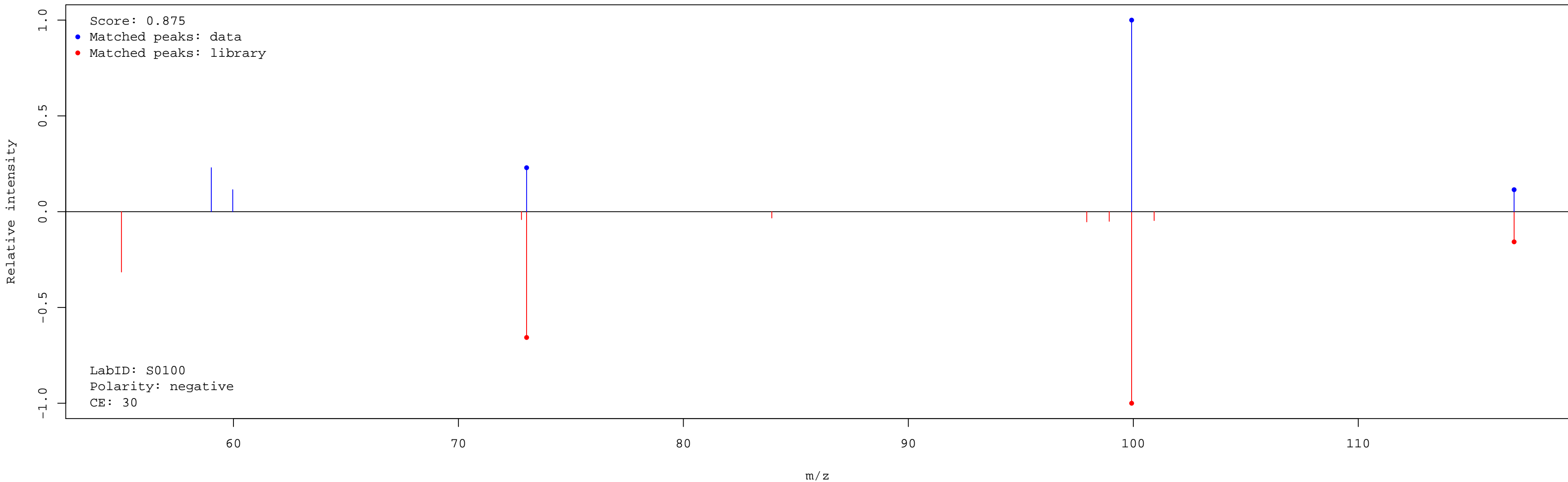

Supplement: Supplementary file 1 [file DataSheet1.ZIP › Supplementary table 1-10 and material 1-3/Material 3-Metlib-MSMS/NEG-Metlib-MSMS/Metlib-MSMS/M117T273_forward/0.875,Methylmalonic acid,(M-H)-.pdf]

# Succinate

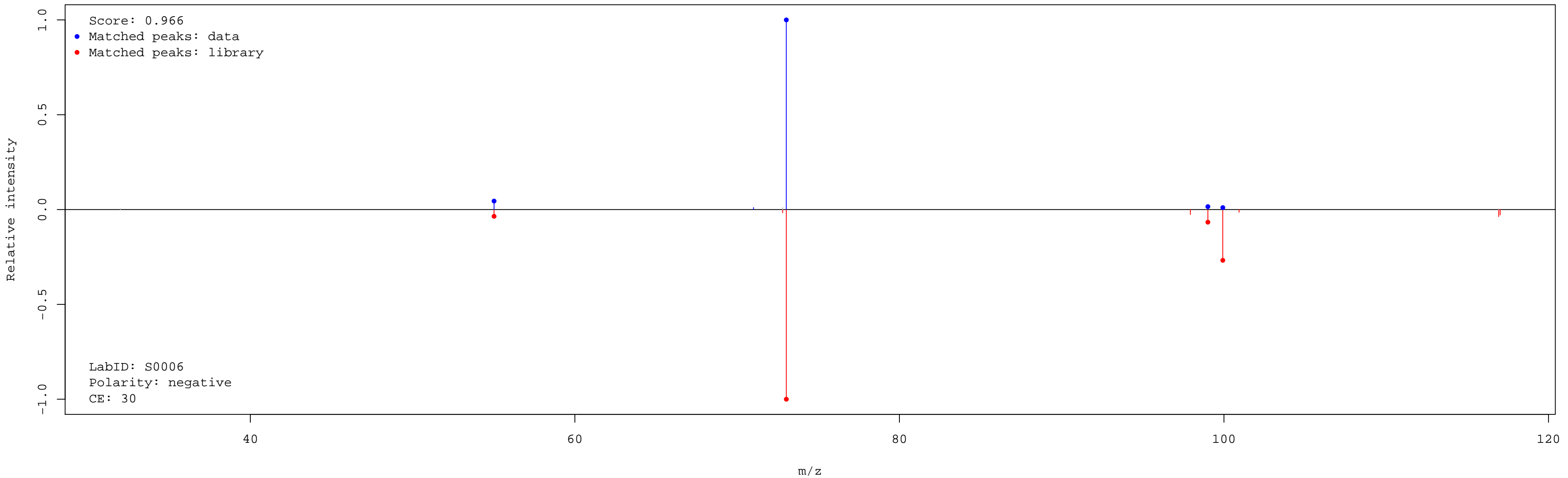

Supplement: Supplementary file 1 [file DataSheet1.ZIP › Supplementary table 1-10 and material 1-3/Material 3-Metlib-MSMS/NEG-Metlib-MSMS/Metlib-MSMS/M117T391_2_forward/0.966,Succinate,(M-H)-.pdf]

# Methylmalonic acid

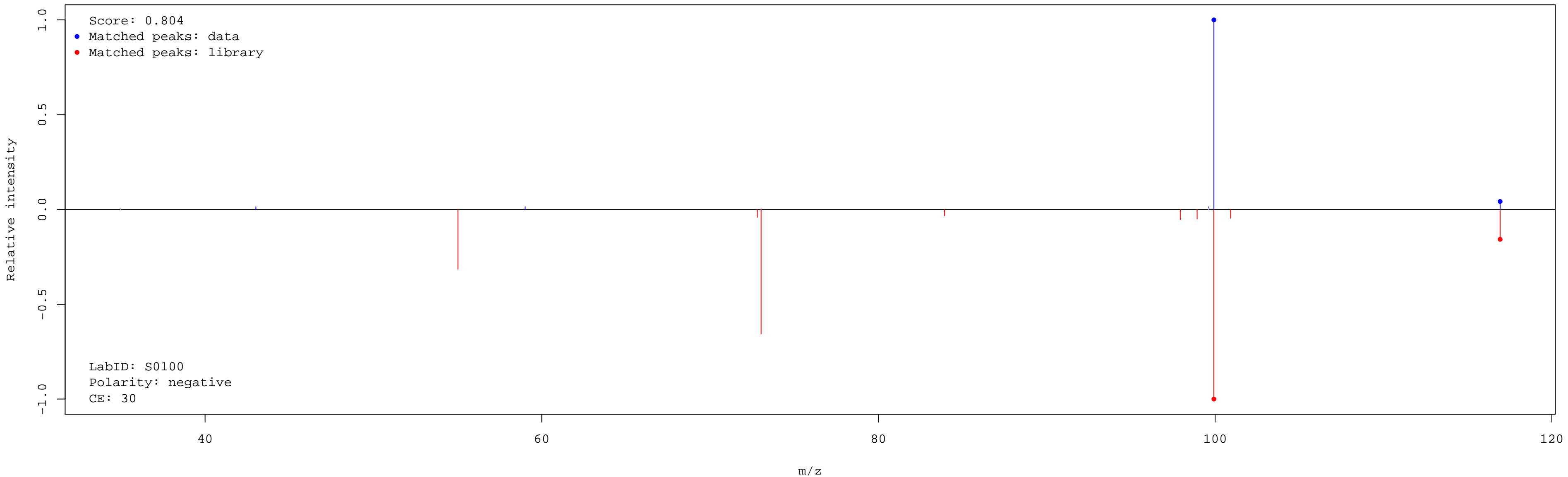

Supplement: Supplementary file 1 [file DataSheet1.ZIP › Supplementary table 1-10 and material 1-3/Material 3-Metlib-MSMS/NEG-Metlib-MSMS/Metlib-MSMS/M117T87_1_forward/0.804,Methylmalonic acid,(M-H)-.pdf]

# L-Threonine

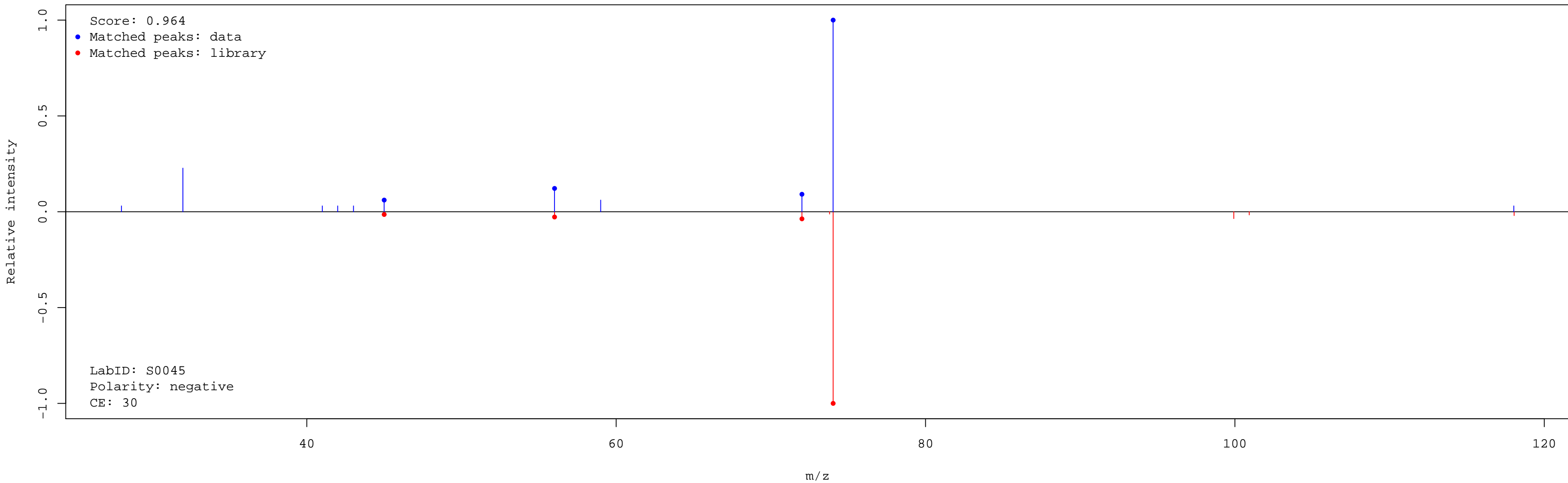

Supplement: Supplementary file 1 [file DataSheet1.ZIP › Supplementary table 1-10 and material 1-3/Material 3-Metlib-MSMS/NEG-Metlib-MSMS/Metlib-MSMS/M118T350_forward/0.964,L-Threonine,(M-H)-.pdf]

# L-Threonine

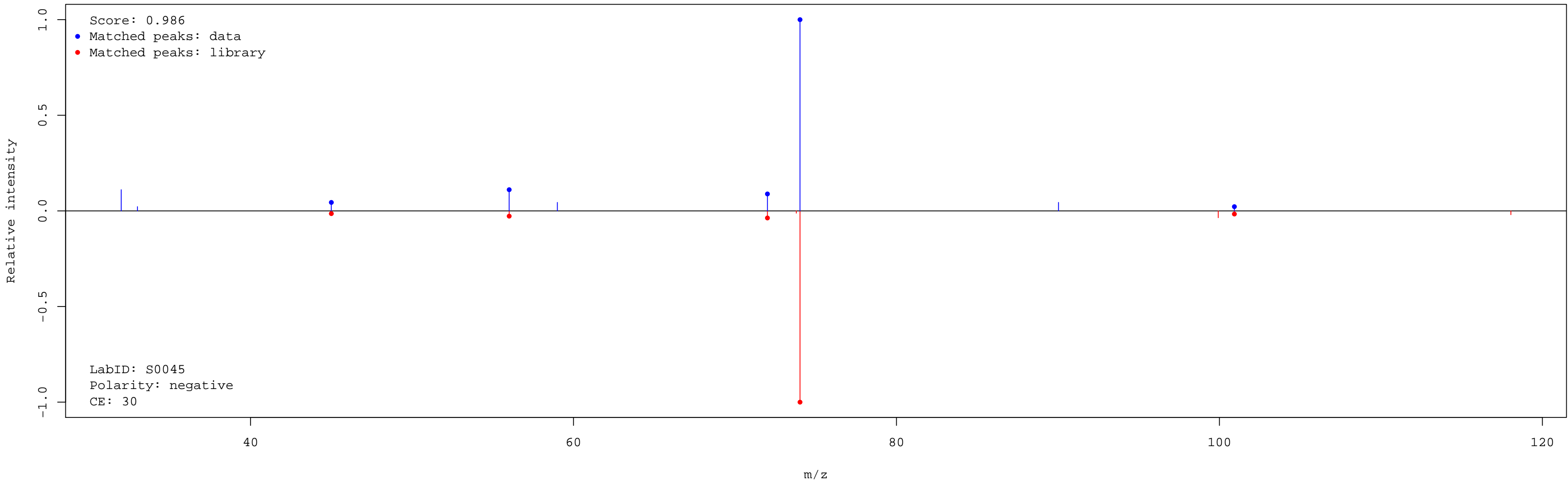

Supplement: Supplementary file 1 [file DataSheet1.ZIP › Supplementary table 1-10 and material 1-3/Material 3-Metlib-MSMS/NEG-Metlib-MSMS/Metlib-MSMS/M118T390_forward/0.986,L-Threonine,(M-H)-.pdf]

# L-Threonine

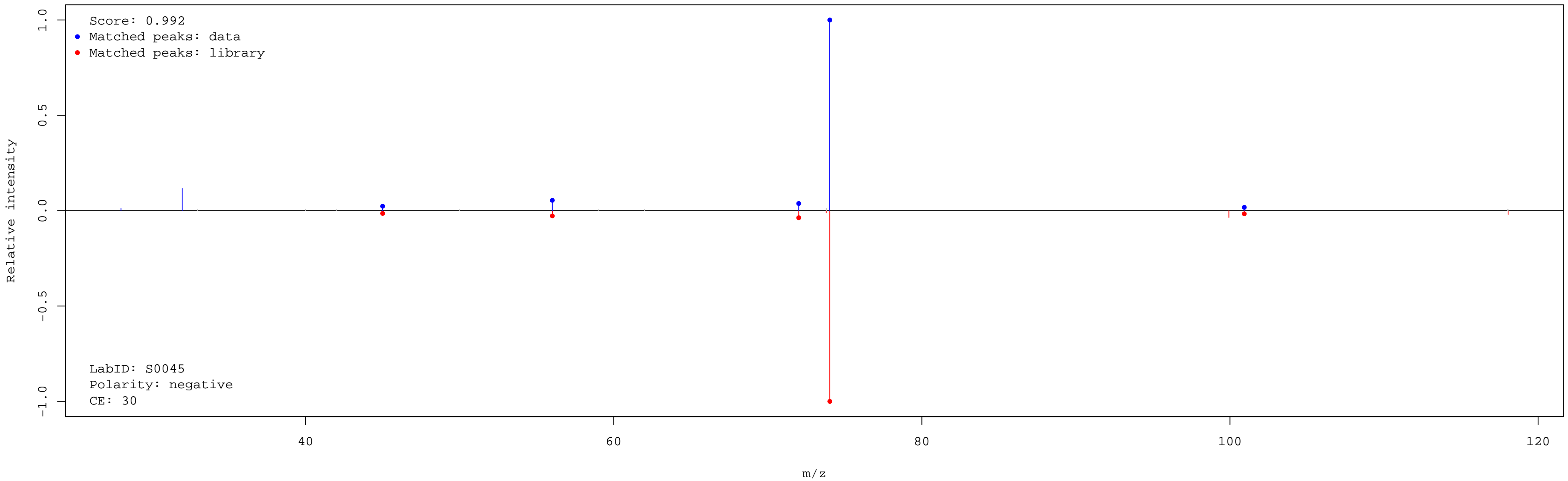

Supplement: Supplementary file 1 [file DataSheet1.ZIP › Supplementary table 1-10 and material 1-3/Material 3-Metlib-MSMS/NEG-Metlib-MSMS/Metlib-MSMS/M118T427_forward/0.992,L-Threonine,(M-H)-.pdf]

# Nicotinate

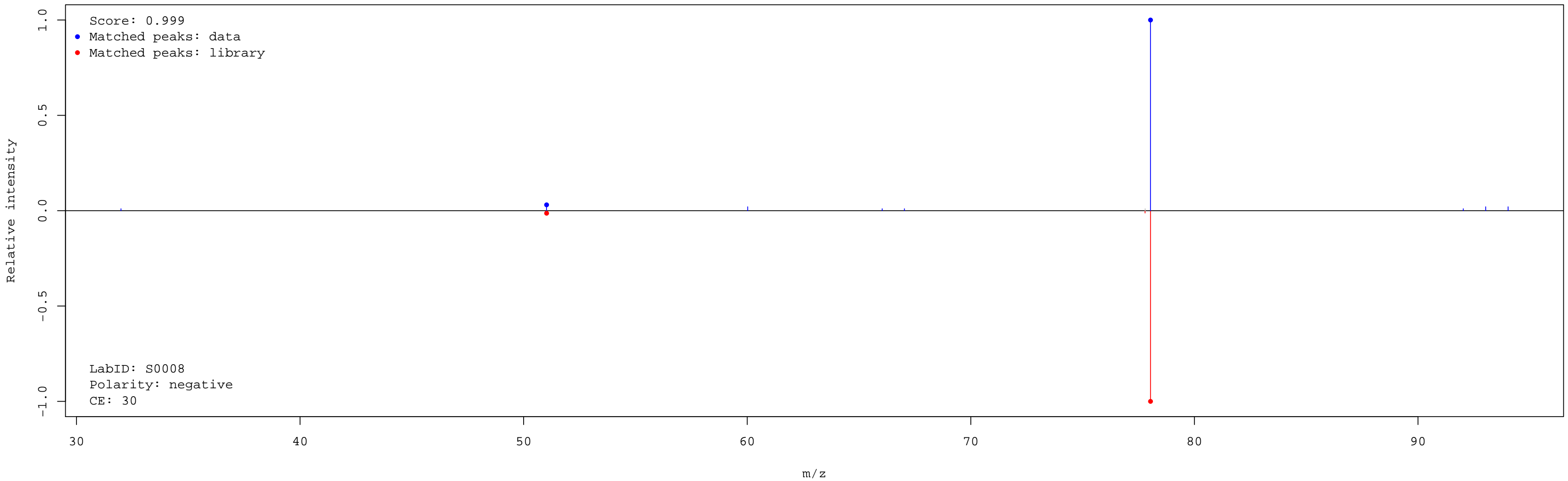

Supplement: Supplementary file 1 [file DataSheet1.ZIP › Supplementary table 1-10 and material 1-3/Material 3-Metlib-MSMS/NEG-Metlib-MSMS/Metlib-MSMS/M122T222_1_forward/0.999,Nicotinate,(M-H)-.pdf]

# Taurine

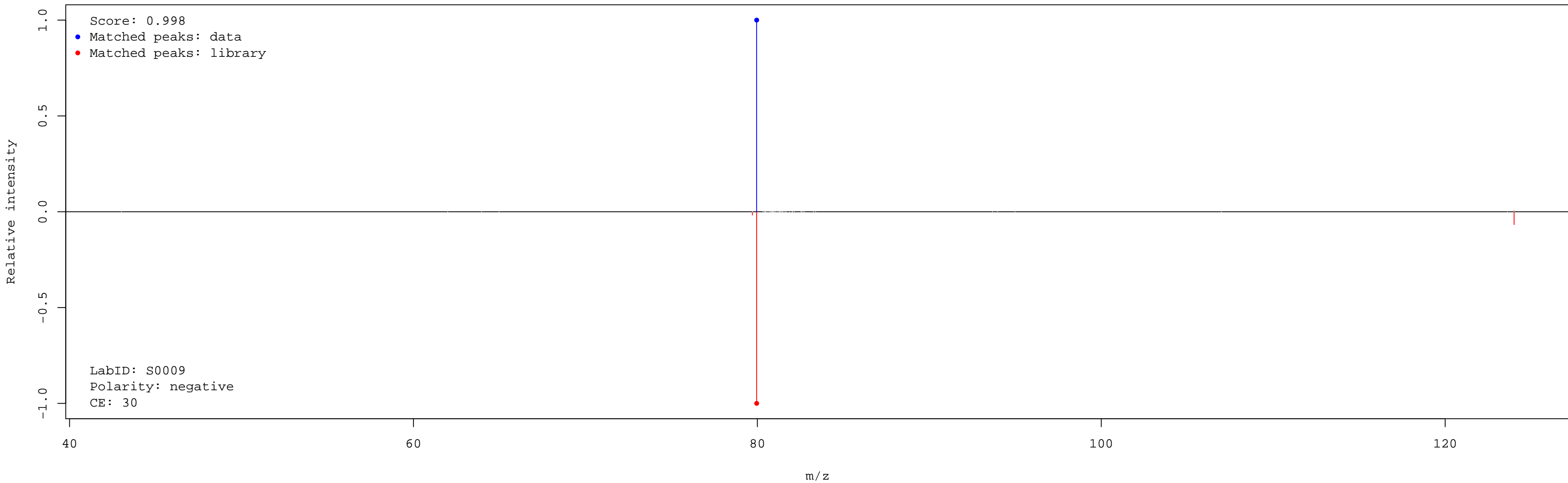

Supplement: Supplementary file 1 [file DataSheet1.ZIP › Supplementary table 1-10 and material 1-3/Material 3-Metlib-MSMS/NEG-Metlib-MSMS/Metlib-MSMS/M124T295_2_forward/0.998,Taurine,(M-H)-.pdf]

# Taurine

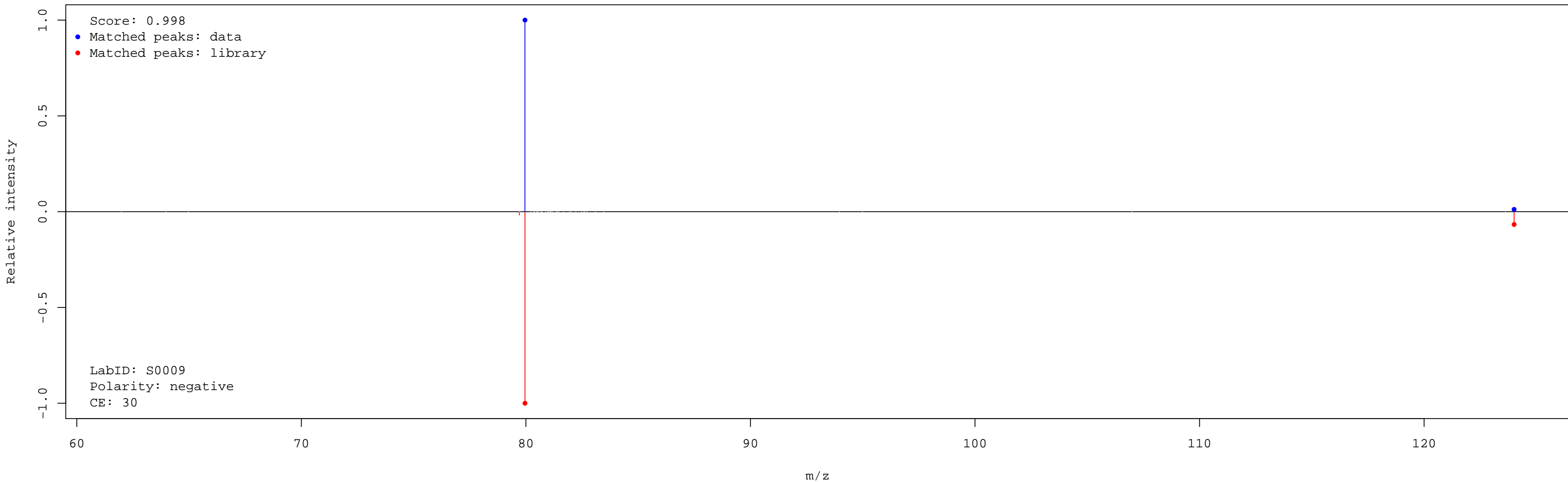

Supplement: Supplementary file 1 [file DataSheet1.ZIP › Supplementary table 1-10 and material 1-3/Material 3-Metlib-MSMS/NEG-Metlib-MSMS/Metlib-MSMS/M124T345_forward/0.998,Taurine,(M-H)-.pdf]

# Taurine

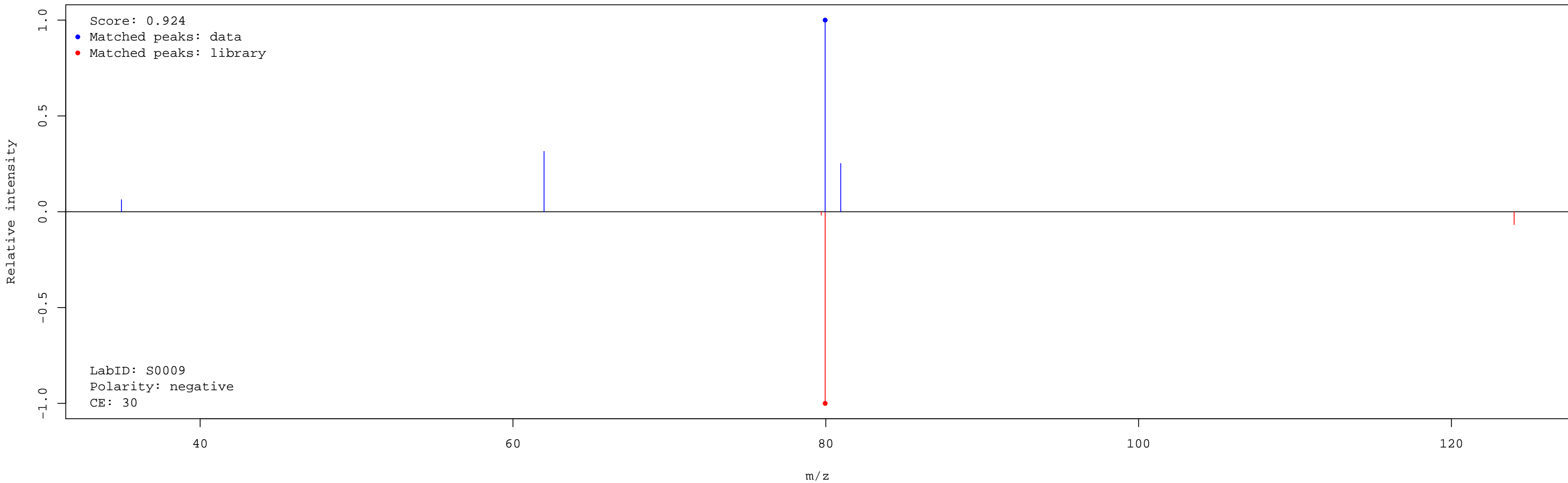

Supplement: Supplementary file 1 [file DataSheet1.ZIP › Supplementary table 1-10 and material 1-3/Material 3-Metlib-MSMS/NEG-Metlib-MSMS/Metlib-MSMS/M124T60_forward/0.924,Taurine,(M-H)-.pdf]

# Thymine

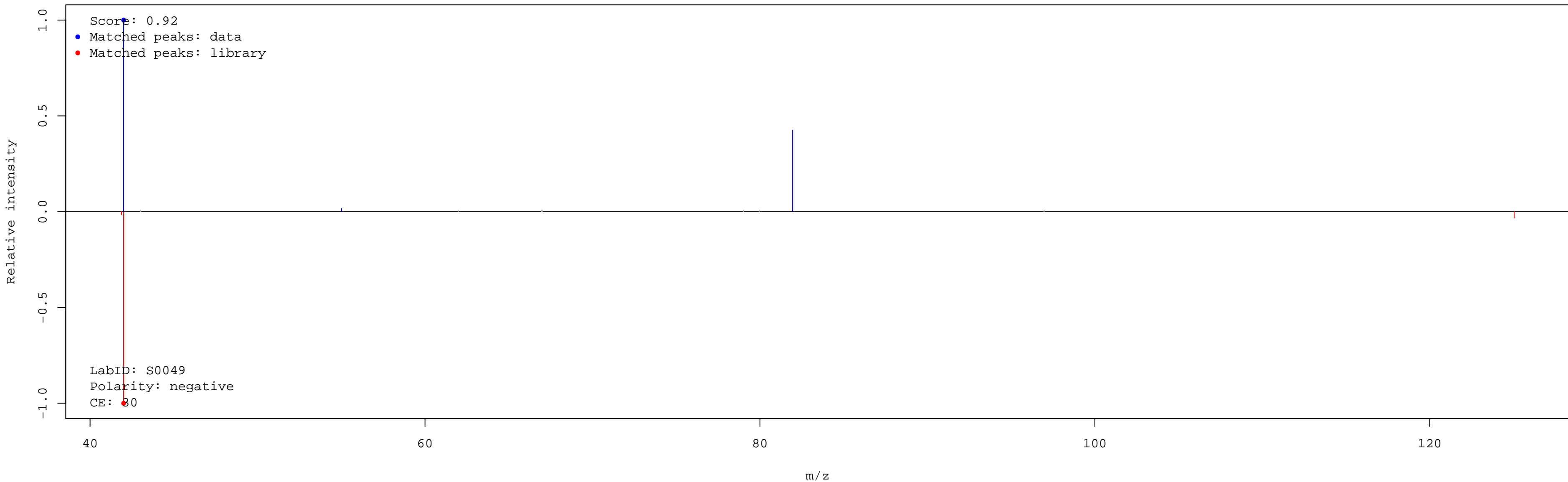

Supplement: Supplementary file 1 [file DataSheet1.ZIP › Supplementary table 1-10 and material 1-3/Material 3-Metlib-MSMS/NEG-Metlib-MSMS/Metlib-MSMS/M125T101_forward/0.92,Thymine,(M-H)-.pdf]

# Thymine

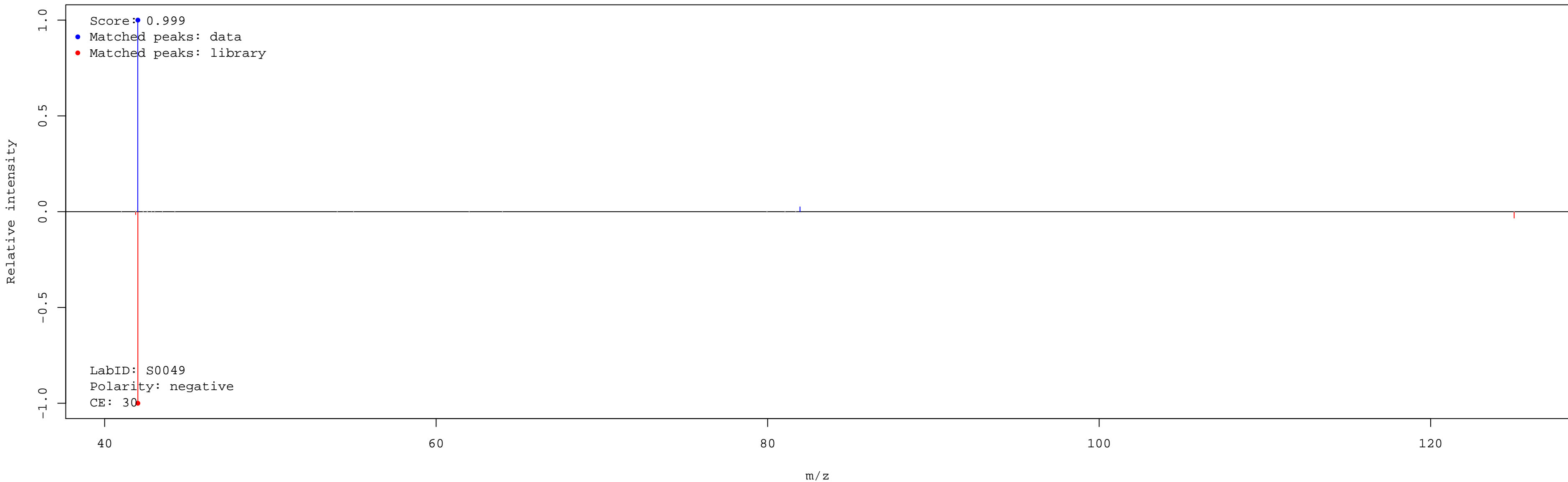

Supplement: Supplementary file 1 [file DataSheet1.ZIP › Supplementary table 1-10 and material 1-3/Material 3-Metlib-MSMS/NEG-Metlib-MSMS/Metlib-MSMS/M125T75_2_forward/0.999,Thymine,(M-H)-.pdf]

# Barbituric acid

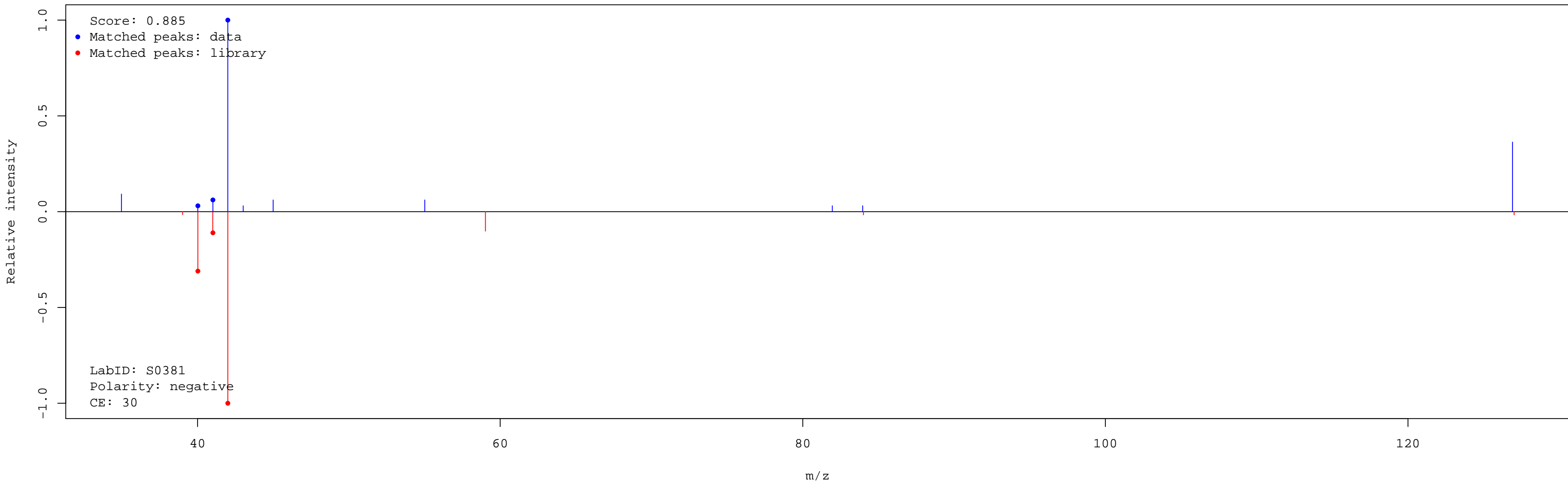

Supplement: Supplementary file 1 [file DataSheet1.ZIP › Supplementary table 1-10 and material 1-3/Material 3-Metlib-MSMS/NEG-Metlib-MSMS/Metlib-MSMS/M127T146_forward/0.885,Barbituric acid,(M-H)-.pdf]

# L-Glutamine

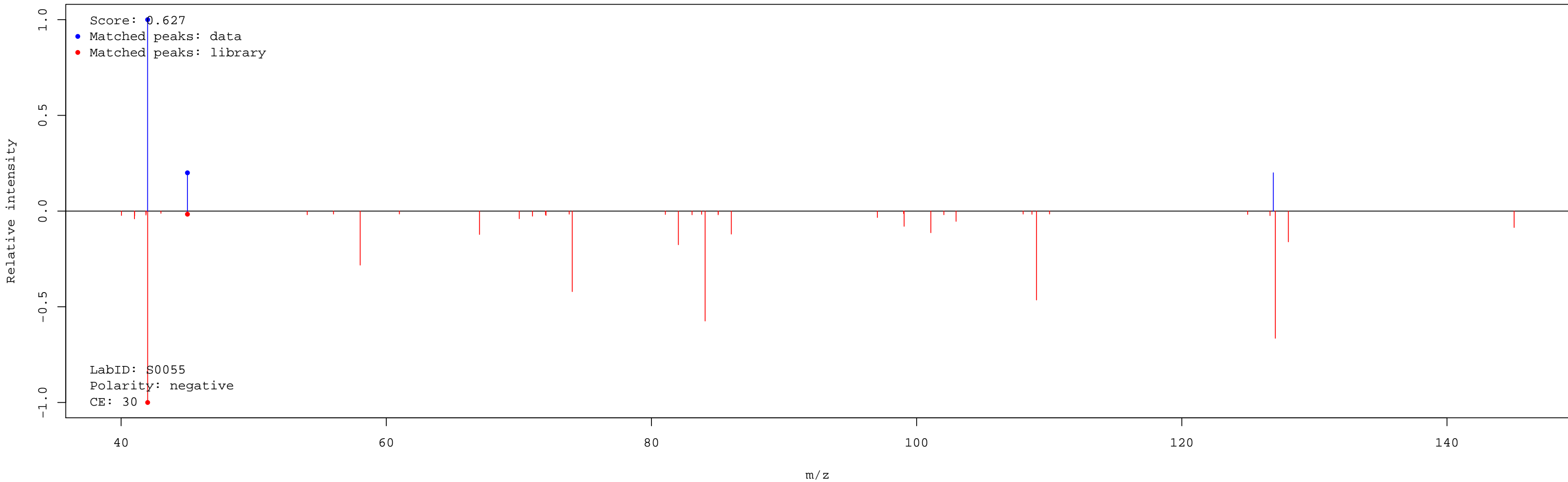

Supplement: Supplementary file 1 [file DataSheet1.ZIP › Supplementary table 1-10 and material 1-3/Material 3-Metlib-MSMS/NEG-Metlib-MSMS/Metlib-MSMS/M127T375_forward/0.627,L-Glutamine,(M-H2O-H)-.pdf]

# Dihydrothymine

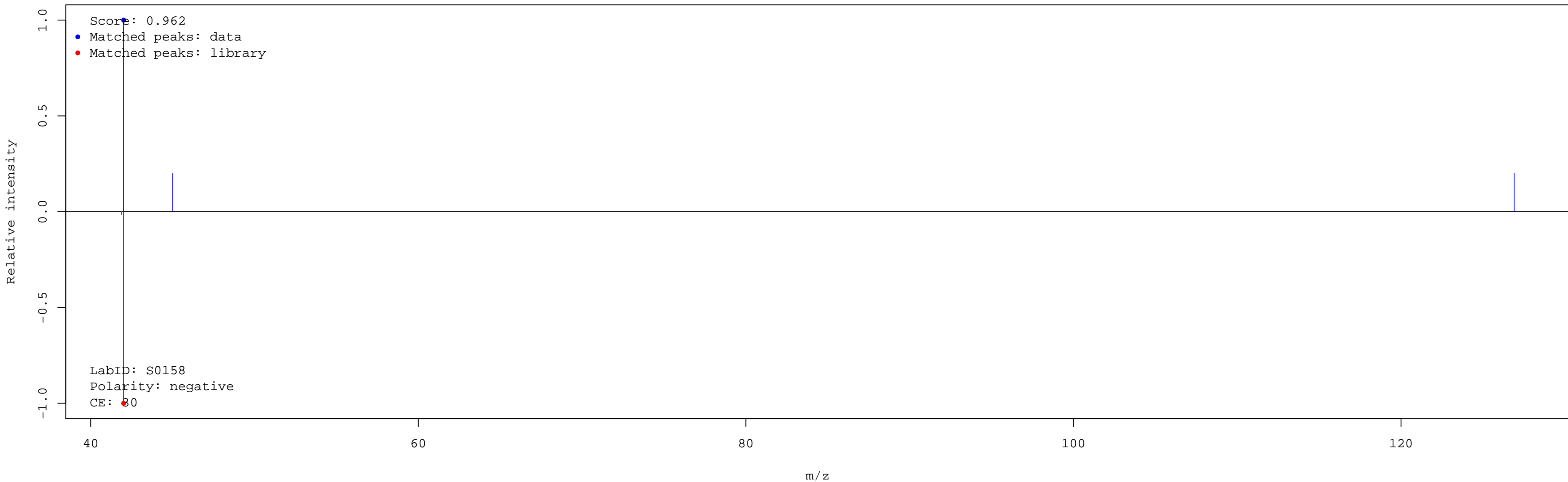

Supplement: Supplementary file 1 [file DataSheet1.ZIP › Supplementary table 1-10 and material 1-3/Material 3-Metlib-MSMS/NEG-Metlib-MSMS/Metlib-MSMS/M127T375_forward/0.962,Dihydrothymine,(M-H)-.pdf]

L-Pyroglutamic acid

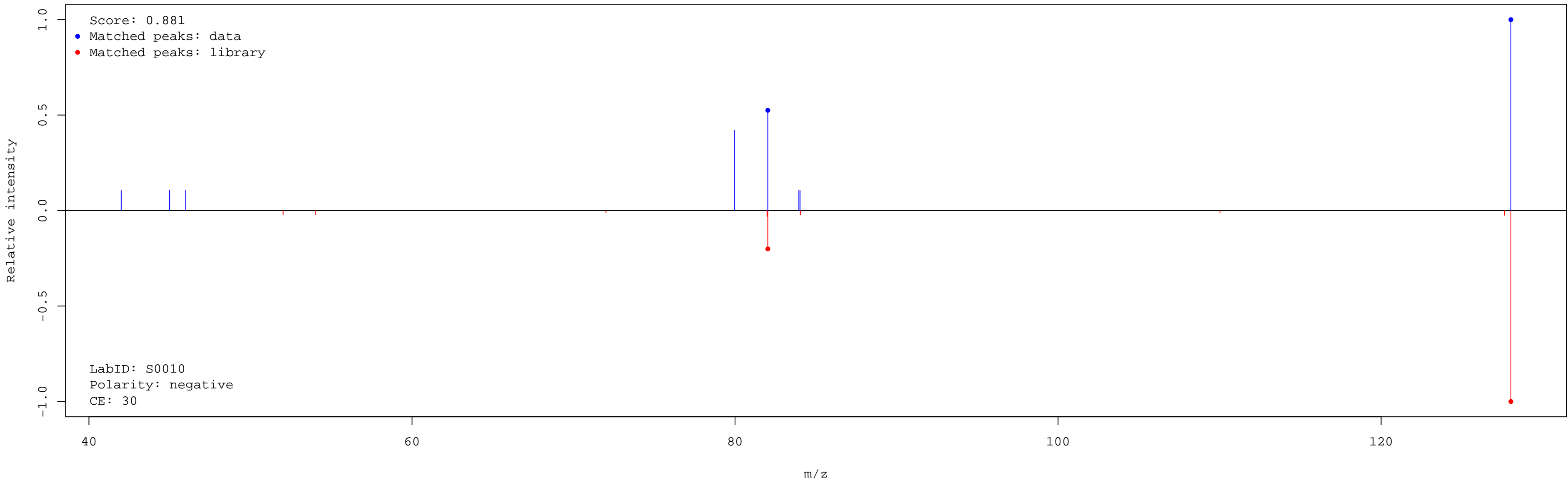

Supplement: Supplementary file 1 [file DataSheet1.ZIP › Supplementary table 1-10 and material 1-3/Material 3-Metlib-MSMS/NEG-Metlib-MSMS/Metlib-MSMS/M128T191_forward/0.881,L-Pyroglutamic acid,(M-H)-.pdf]

# L-Pyroglutamic acid

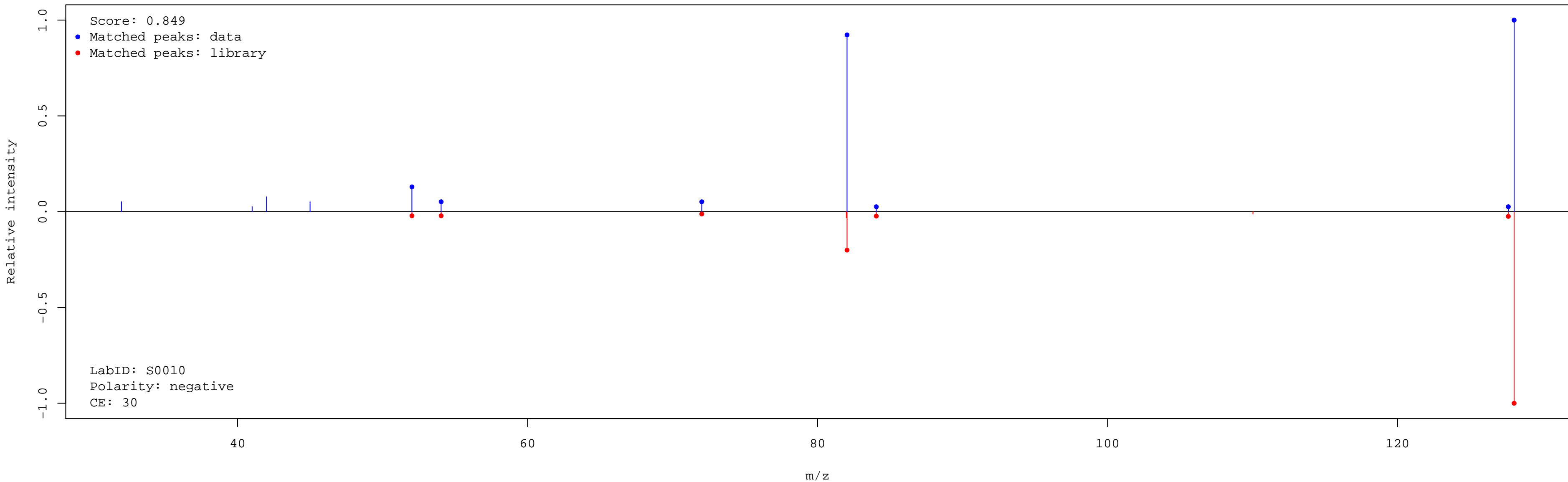

Supplement: Supplementary file 1 [file DataSheet1.ZIP › Supplementary table 1-10 and material 1-3/Material 3-Metlib-MSMS/NEG-Metlib-MSMS/Metlib-MSMS/M128T301_2_forward/0.849,L-Pyroglutamic acid,(M-H)-.pdf]

# L-Pyroglutamic acid

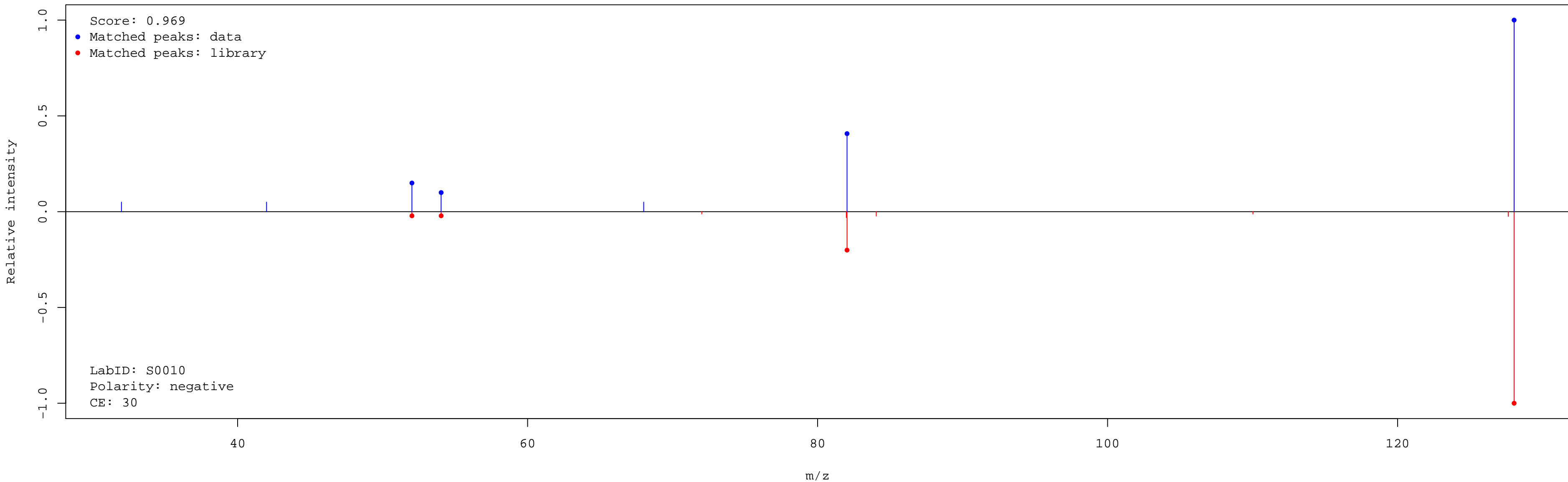

Supplement: Supplementary file 1 [file DataSheet1.ZIP › Supplementary table 1-10 and material 1-3/Material 3-Metlib-MSMS/NEG-Metlib-MSMS/Metlib-MSMS/M128T374_forward/0.969,L-Pyroglutamic acid,(M-H)-.pdf]

# L-Pyroglutamic acid

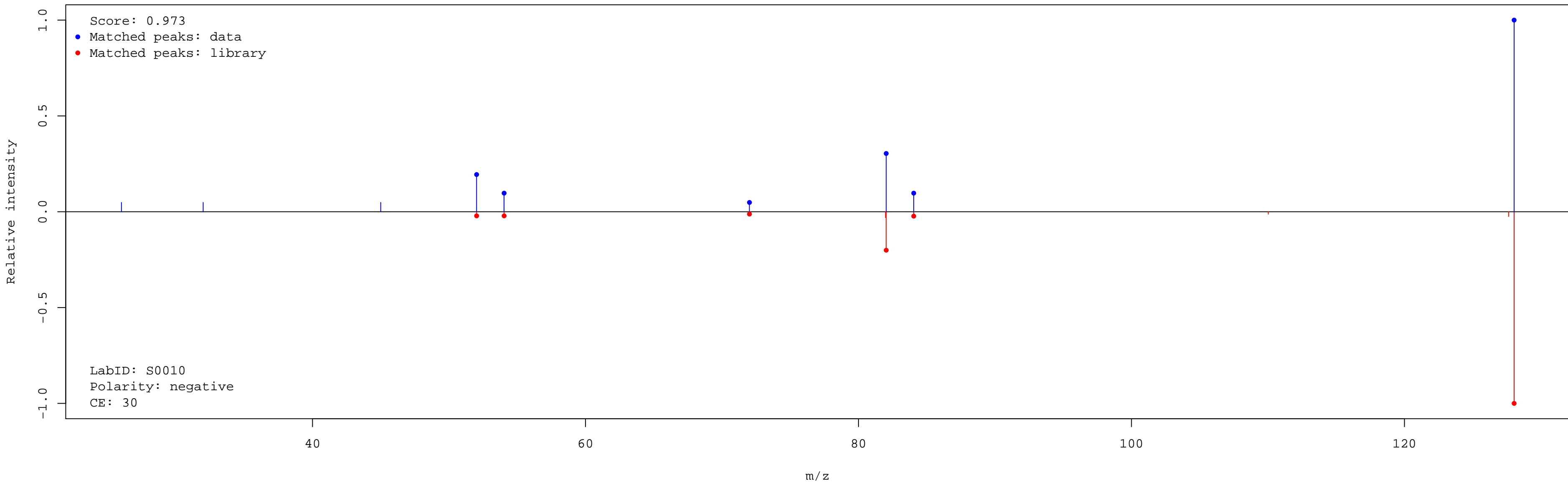

Supplement: Supplementary file 1 [file DataSheet1.ZIP › Supplementary table 1-10 and material 1-3/Material 3-Metlib-MSMS/NEG-Metlib-MSMS/Metlib-MSMS/M128T399_2_forward/0.973,L-Pyroglutamic acid,(M-H)-.pdf]

L-Isoleucine

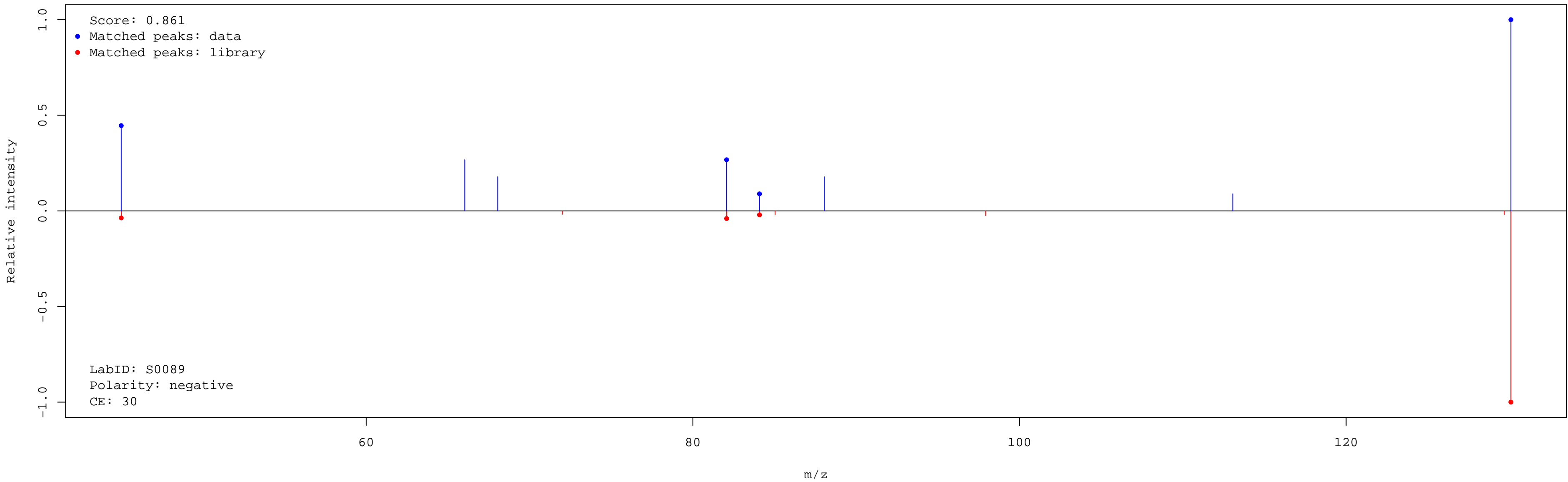

Supplement: Supplementary file 1 [file DataSheet1.ZIP › Supplementary table 1-10 and material 1-3/Material 3-Metlib-MSMS/NEG-Metlib-MSMS/Metlib-MSMS/M130T179_forward/0.861,L-Isoleucine,(M-H)-.pdf]

L-Norleucine

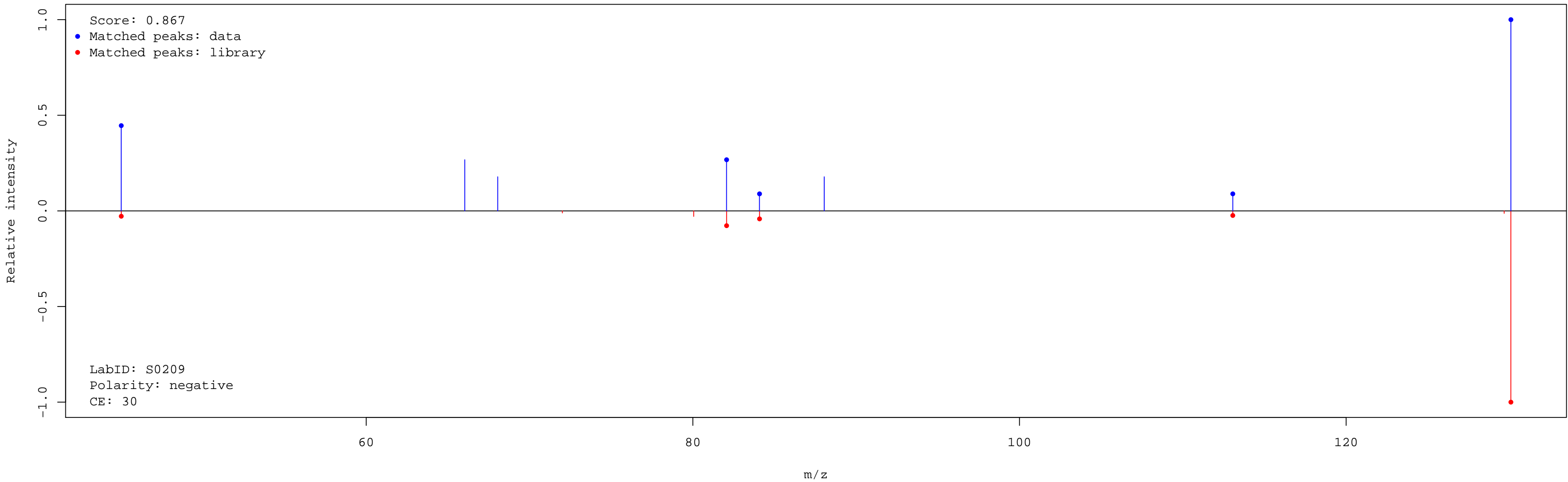

Supplement: Supplementary file 1 [file DataSheet1.ZIP › Supplementary table 1-10 and material 1-3/Material 3-Metlib-MSMS/NEG-Metlib-MSMS/Metlib-MSMS/M130T179_forward/0.867,L-Norleucine,(M-H)-.pdf]

# L-Leucine

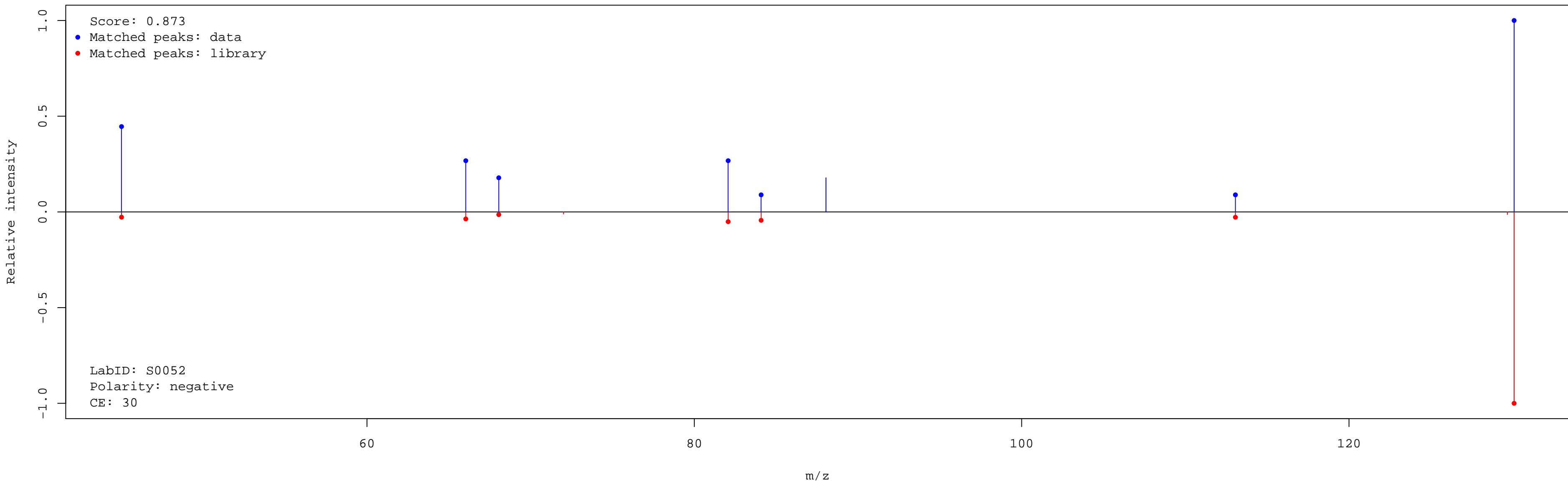

Supplement: Supplementary file 1 [file DataSheet1.ZIP › Supplementary table 1-10 and material 1-3/Material 3-Metlib-MSMS/NEG-Metlib-MSMS/Metlib-MSMS/M130T179_forward/0.873,L-Leucine,(M-H)-.pdf]

L-Isoleucine

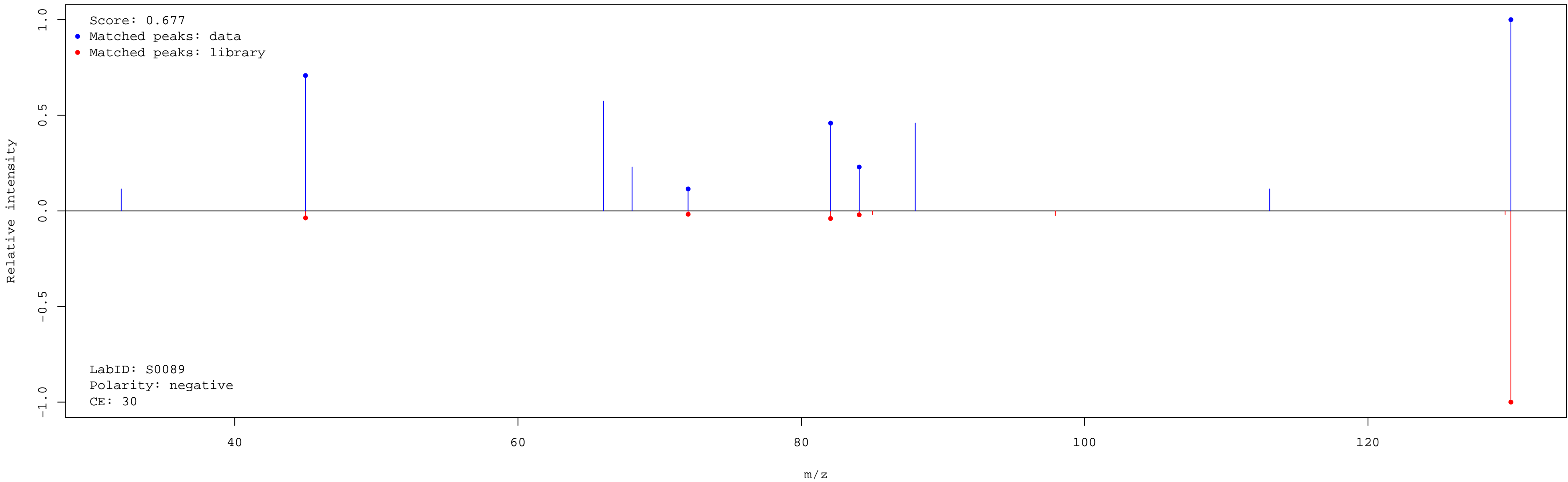

Supplement: Supplementary file 1 [file DataSheet1.ZIP › Supplementary table 1-10 and material 1-3/Material 3-Metlib-MSMS/NEG-Metlib-MSMS/Metlib-MSMS/M130T195_forward/0.677,L-Isoleucine,(M-H)-.pdf]

L-Norleucine

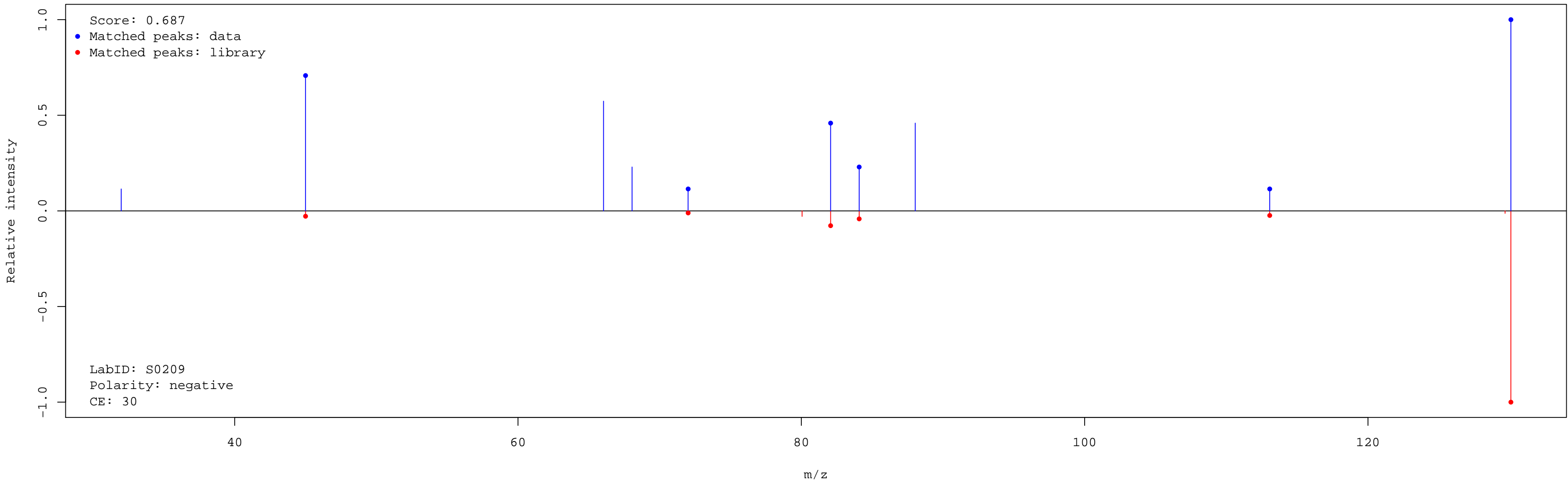

Supplement: Supplementary file 1 [file DataSheet1.ZIP › Supplementary table 1-10 and material 1-3/Material 3-Metlib-MSMS/NEG-Metlib-MSMS/Metlib-MSMS/M130T195_forward/0.687,L-Norleucine,(M-H)-.pdf]

# L-Leucine

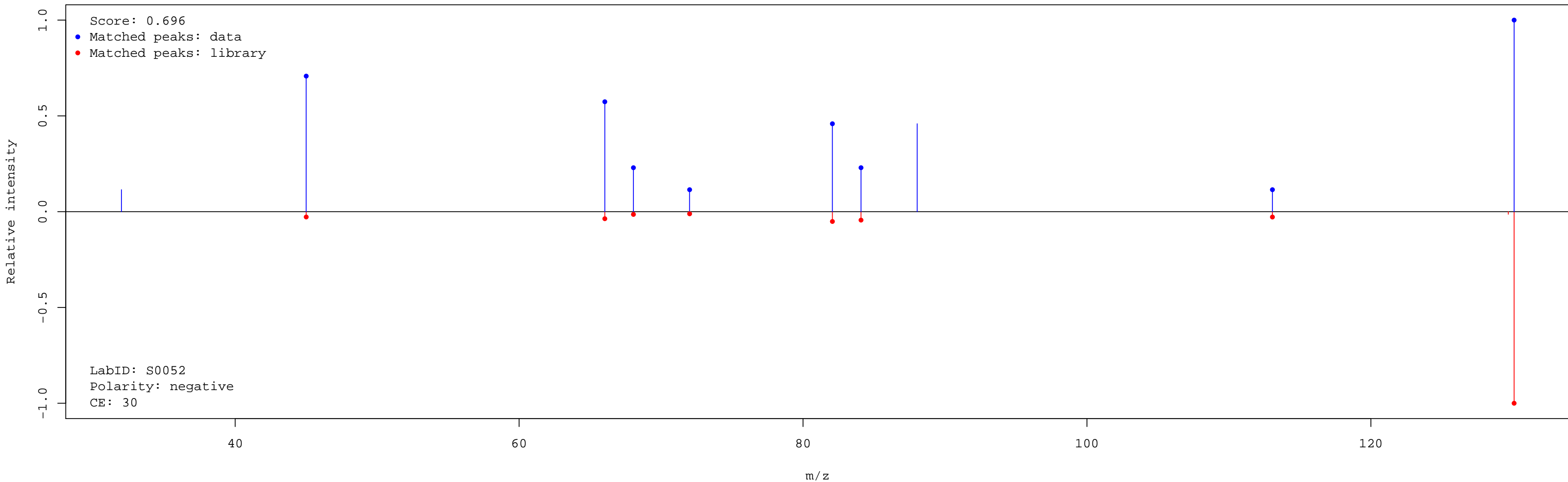

Supplement: Supplementary file 1 [file DataSheet1.ZIP › Supplementary table 1-10 and material 1-3/Material 3-Metlib-MSMS/NEG-Metlib-MSMS/Metlib-MSMS/M130T195_forward/0.696,L-Leucine,(M-H)-.pdf]

# N-Acetyl-L-alanine

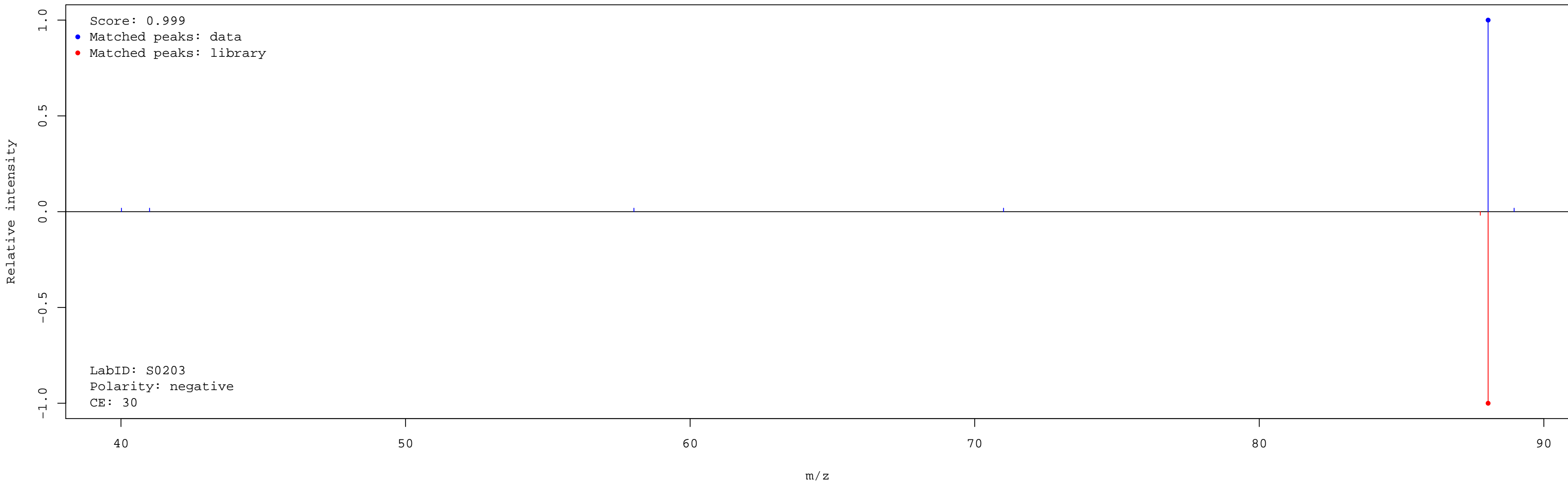

Supplement: Supplementary file 1 [file DataSheet1.ZIP › Supplementary table 1-10 and material 1-3/Material 3-Metlib-MSMS/NEG-Metlib-MSMS/Metlib-MSMS/M130T255_forward/0.999,N-Acetyl-L-alanine,(M-H)-.pdf]

L-Isoleucine

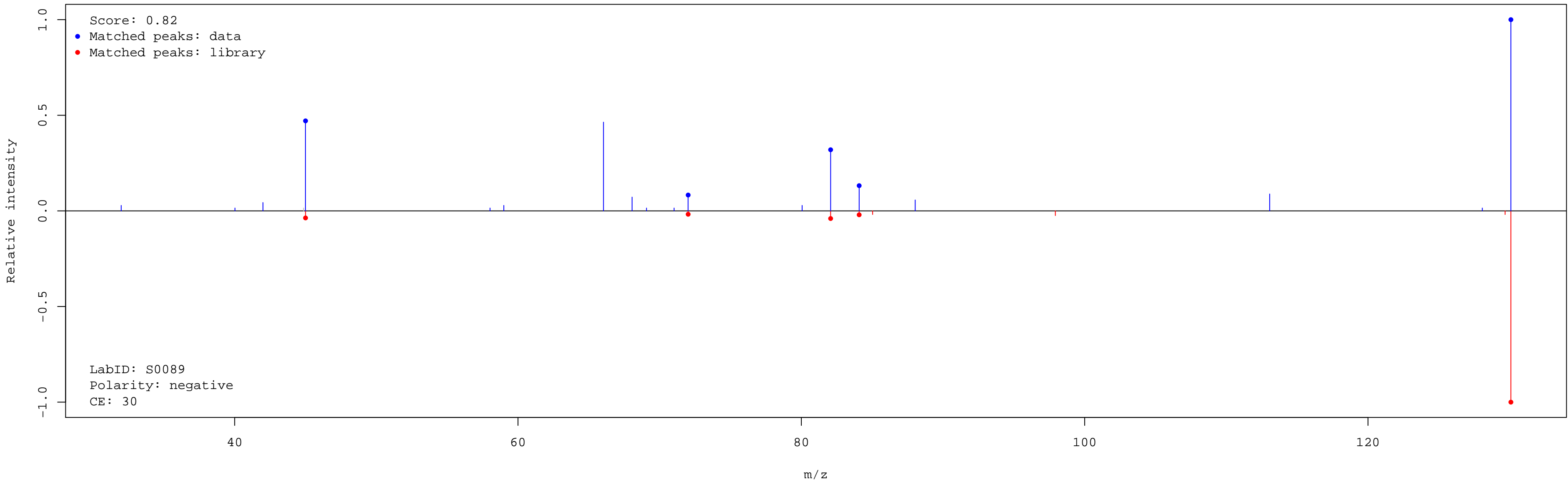

Supplement: Supplementary file 1 [file DataSheet1.ZIP › Supplementary table 1-10 and material 1-3/Material 3-Metlib-MSMS/NEG-Metlib-MSMS/Metlib-MSMS/M130T262_2_forward/0.82,L-Isoleucine,(M-H)-.pdf]

L-Norleucine

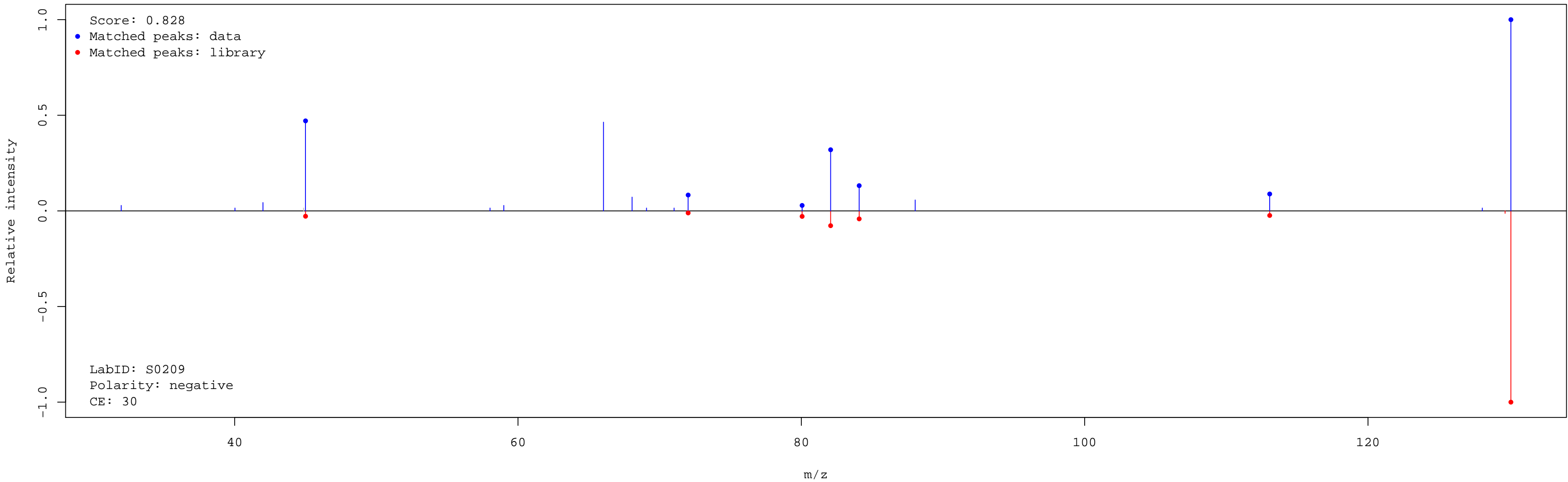

Supplement: Supplementary file 1 [file DataSheet1.ZIP › Supplementary table 1-10 and material 1-3/Material 3-Metlib-MSMS/NEG-Metlib-MSMS/Metlib-MSMS/M130T262_2_forward/0.828,L-Norleucine,(M-H)-.pdf]

# L-Leucine

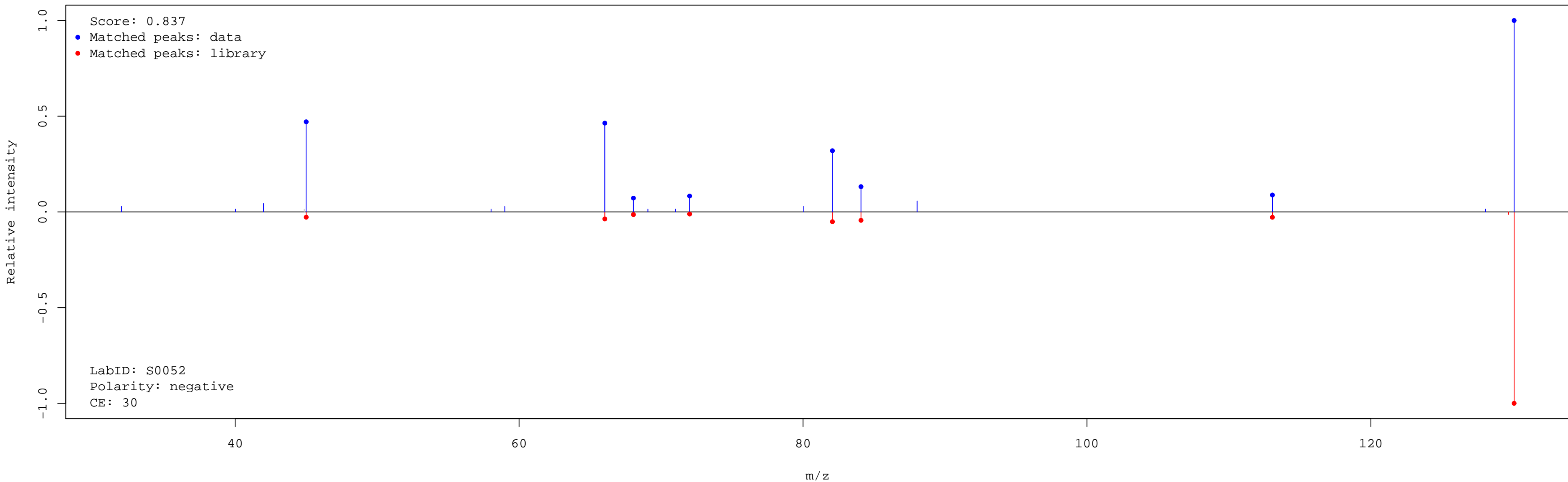

Supplement: Supplementary file 1 [file DataSheet1.ZIP › Supplementary table 1-10 and material 1-3/Material 3-Metlib-MSMS/NEG-Metlib-MSMS/Metlib-MSMS/M130T262_2_forward/0.837,L-Leucine,(M-H)-.pdf]

L-Isoleucine

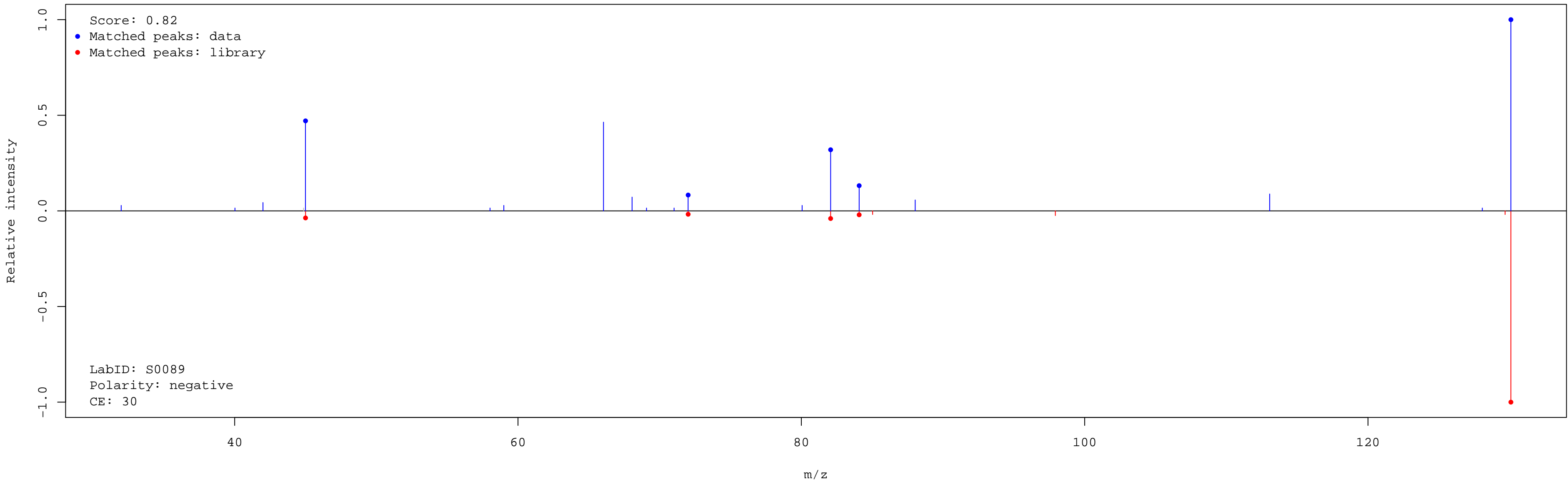

Supplement: Supplementary file 1 [file DataSheet1.ZIP › Supplementary table 1-10 and material 1-3/Material 3-Metlib-MSMS/NEG-Metlib-MSMS/Metlib-MSMS/M130T309_forward/0.82,L-Isoleucine,(M-H)-.pdf]

L-Norleucine

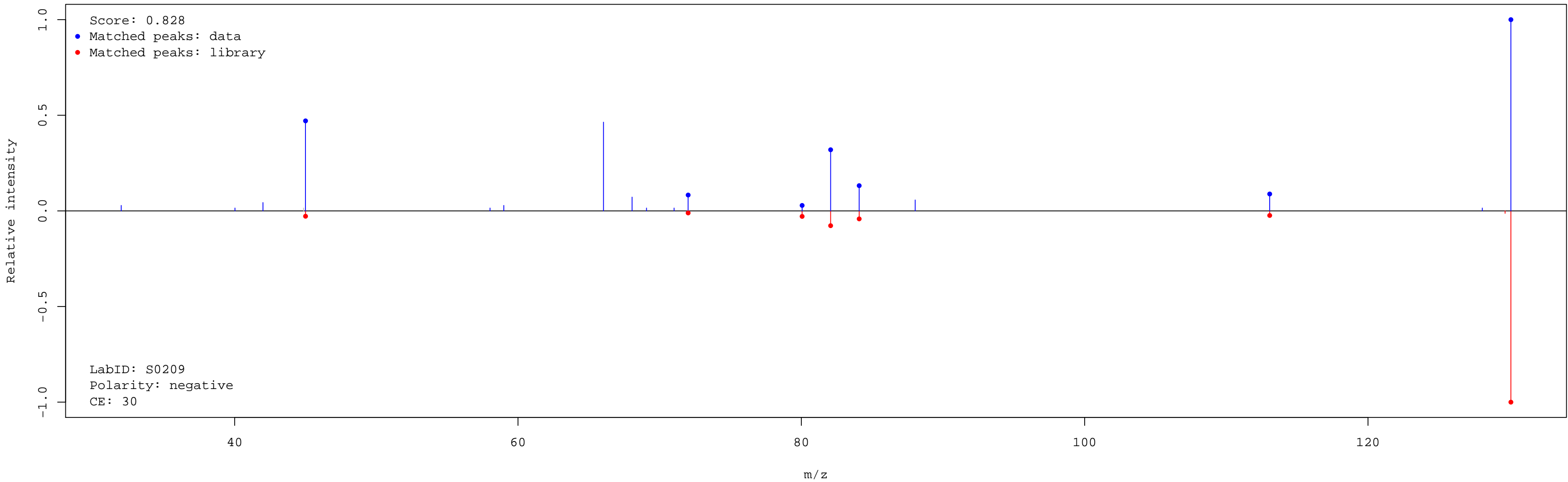

Supplement: Supplementary file 1 [file DataSheet1.ZIP › Supplementary table 1-10 and material 1-3/Material 3-Metlib-MSMS/NEG-Metlib-MSMS/Metlib-MSMS/M130T309_forward/0.828,L-Norleucine,(M-H)-.pdf]

# L-Leucine

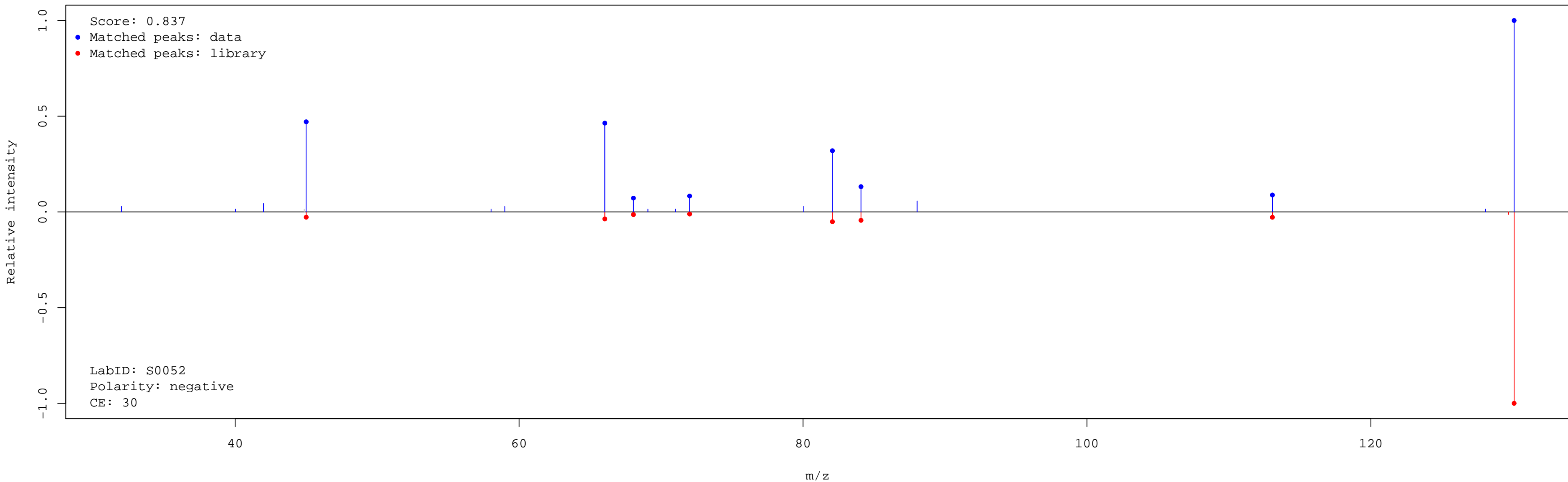

Supplement: Supplementary file 1 [file DataSheet1.ZIP › Supplementary table 1-10 and material 1-3/Material 3-Metlib-MSMS/NEG-Metlib-MSMS/Metlib-MSMS/M130T309_forward/0.837,L-Leucine,(M-H)-.pdf]

# Creatine

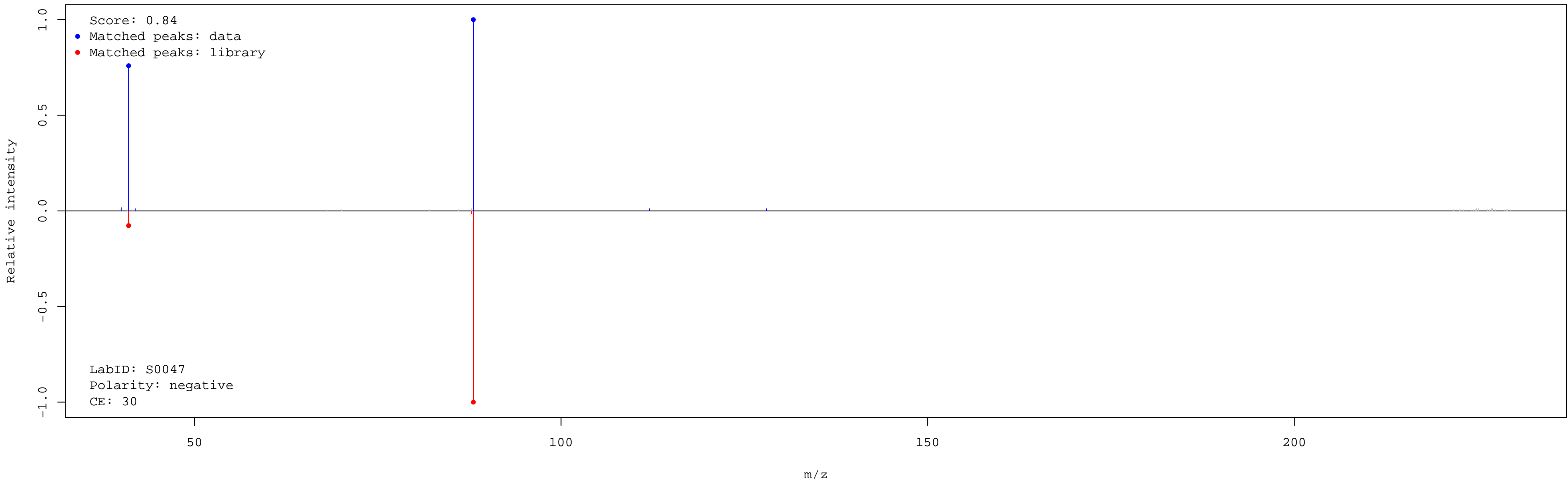

Supplement: Supplementary file 1 [file DataSheet1.ZIP › Supplementary table 1-10 and material 1-3/Material 3-Metlib-MSMS/NEG-Metlib-MSMS/Metlib-MSMS/M130T347_2_forward/0.84,Creatine,(M-H)-.pdf]

L-Isoleucine

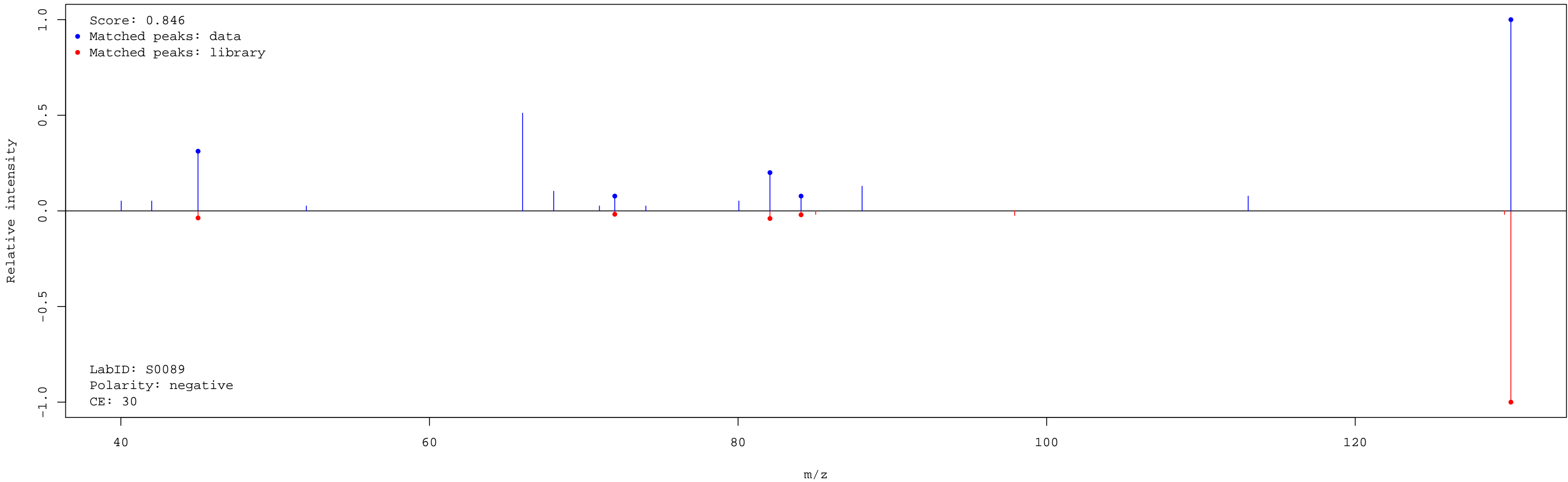

Supplement: Supplementary file 1 [file DataSheet1.ZIP › Supplementary table 1-10 and material 1-3/Material 3-Metlib-MSMS/NEG-Metlib-MSMS/Metlib-MSMS/M130T355_forward/0.846,L-Isoleucine,(M-H)-.pdf]

L-Norleucine

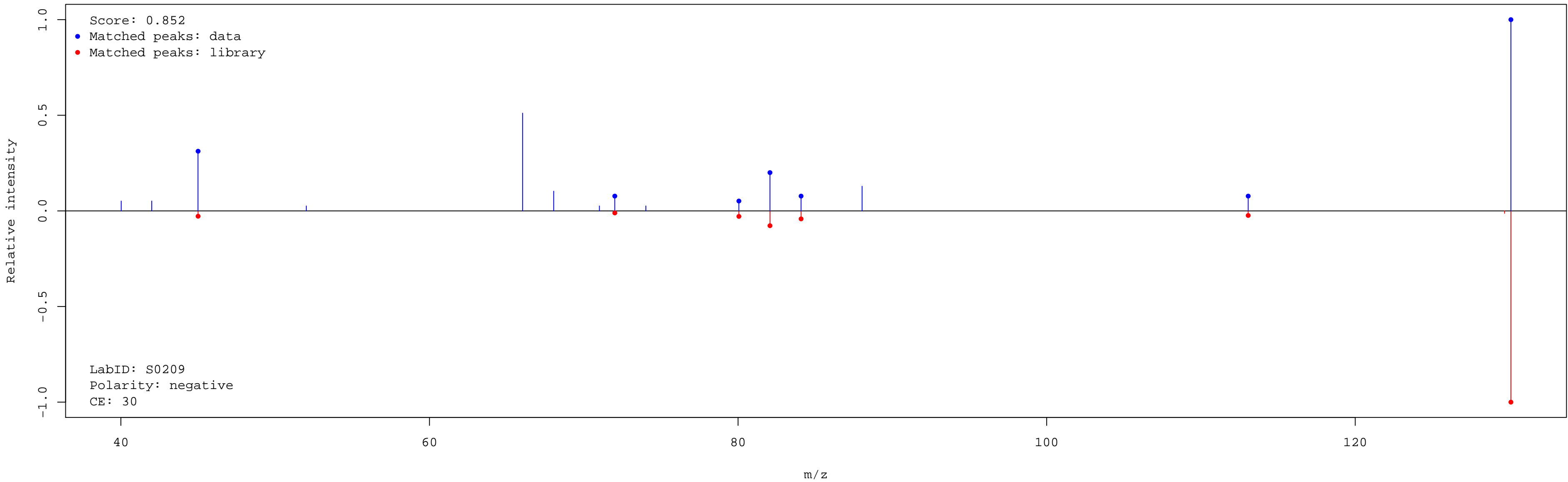

Supplement: Supplementary file 1 [file DataSheet1.ZIP › Supplementary table 1-10 and material 1-3/Material 3-Metlib-MSMS/NEG-Metlib-MSMS/Metlib-MSMS/M130T355_forward/0.852,L-Norleucine,(M-H)-.pdf]

# L-Leucine

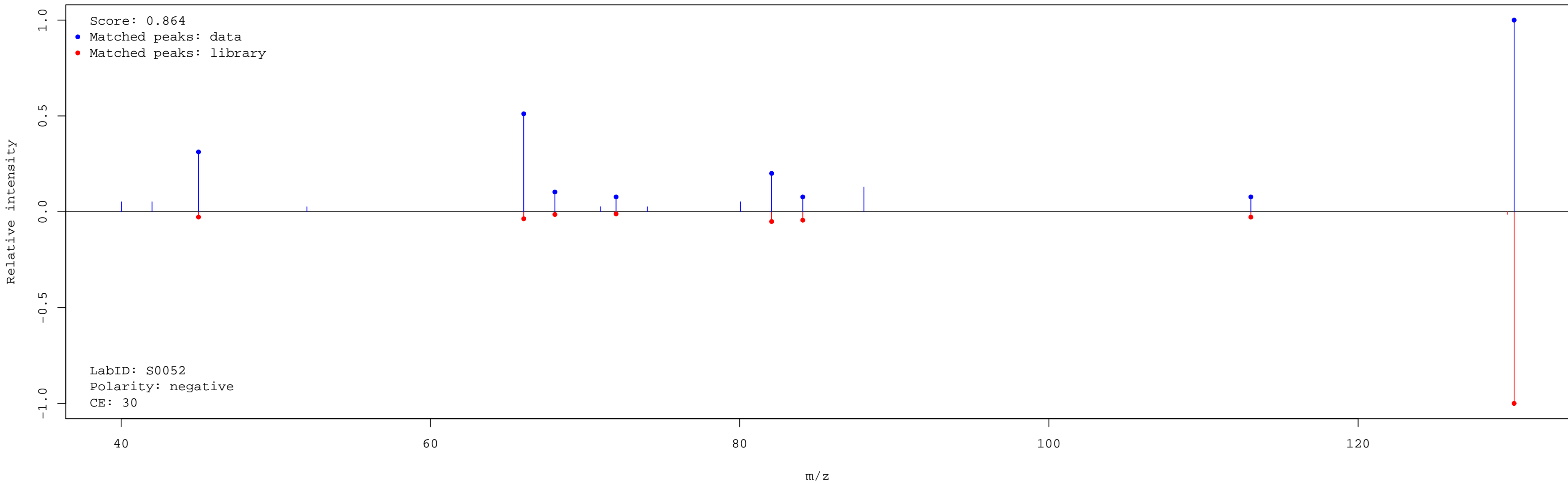

Supplement: Supplementary file 1 [file DataSheet1.ZIP › Supplementary table 1-10 and material 1-3/Material 3-Metlib-MSMS/NEG-Metlib-MSMS/Metlib-MSMS/M130T355_forward/0.864,L-Leucine,(M-H)-.pdf]

# N-Acetyl-L-alanine

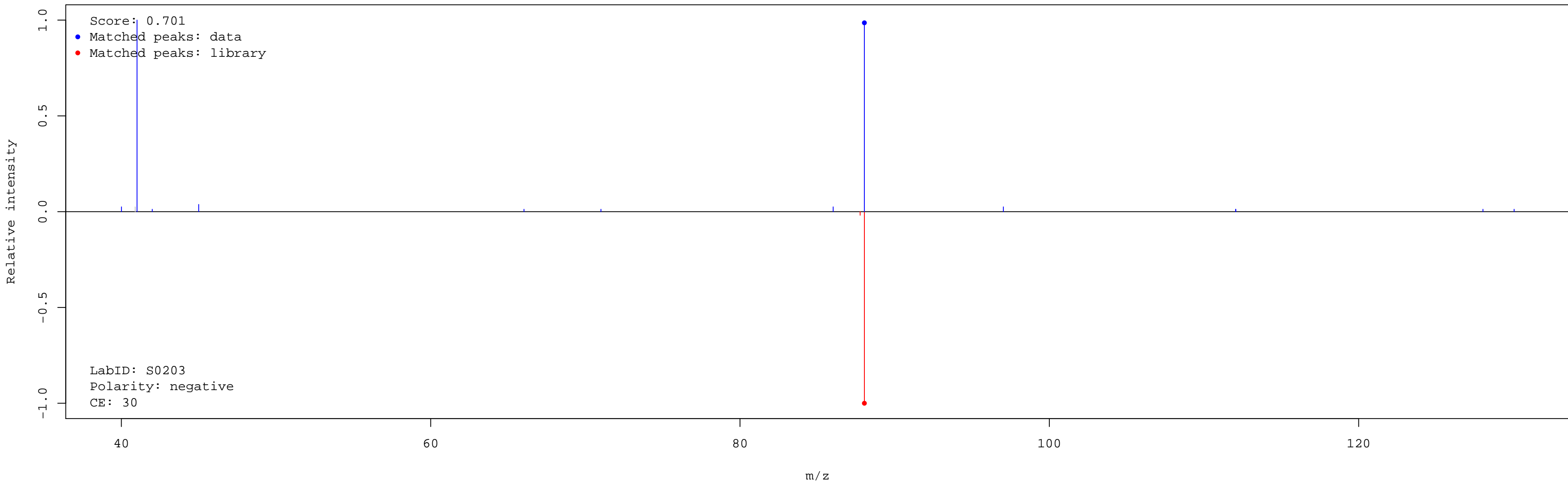

Supplement: Supplementary file 1 [file DataSheet1.ZIP › Supplementary table 1-10 and material 1-3/Material 3-Metlib-MSMS/NEG-Metlib-MSMS/Metlib-MSMS/M130T367_forward/0.701,N-Acetyl-L-alanine,(M-H)-.pdf]

# 5-Hydroxyhexanoic acid

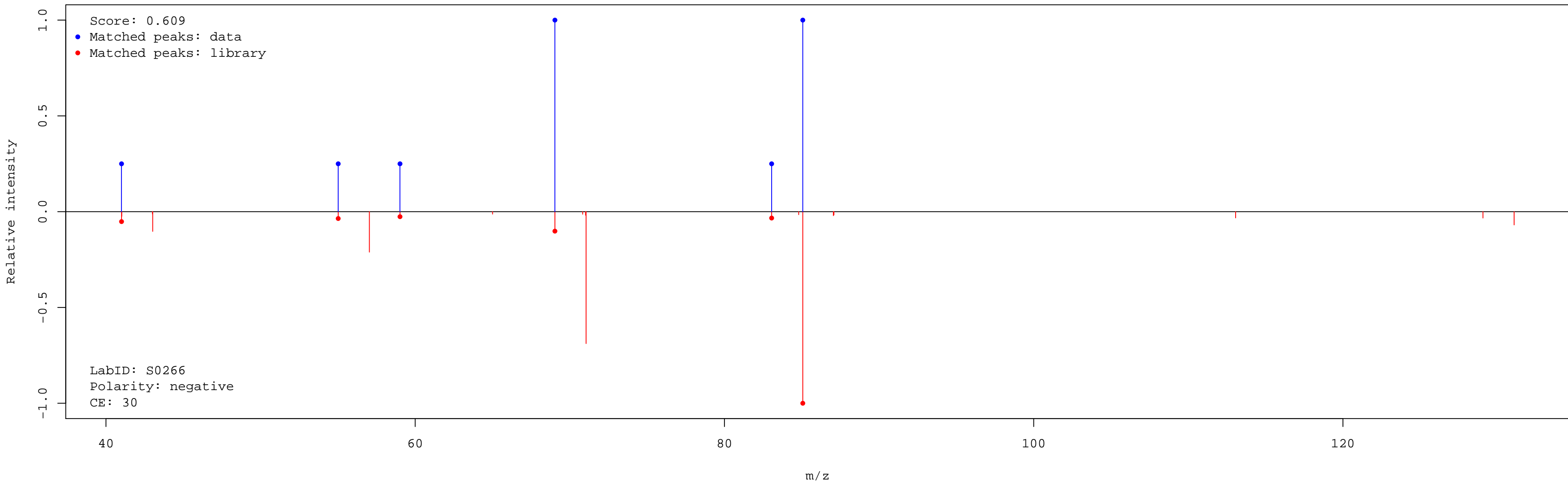

Supplement: Supplementary file 1 [file DataSheet1.ZIP › Supplementary table 1-10 and material 1-3/Material 3-Metlib-MSMS/NEG-Metlib-MSMS/Metlib-MSMS/M131T133_forward/0.609,5-Hydroxyhexanoic acid,(M-H)-.pdf]

# 2-Ethyl-2-Hydroxybutyric acid

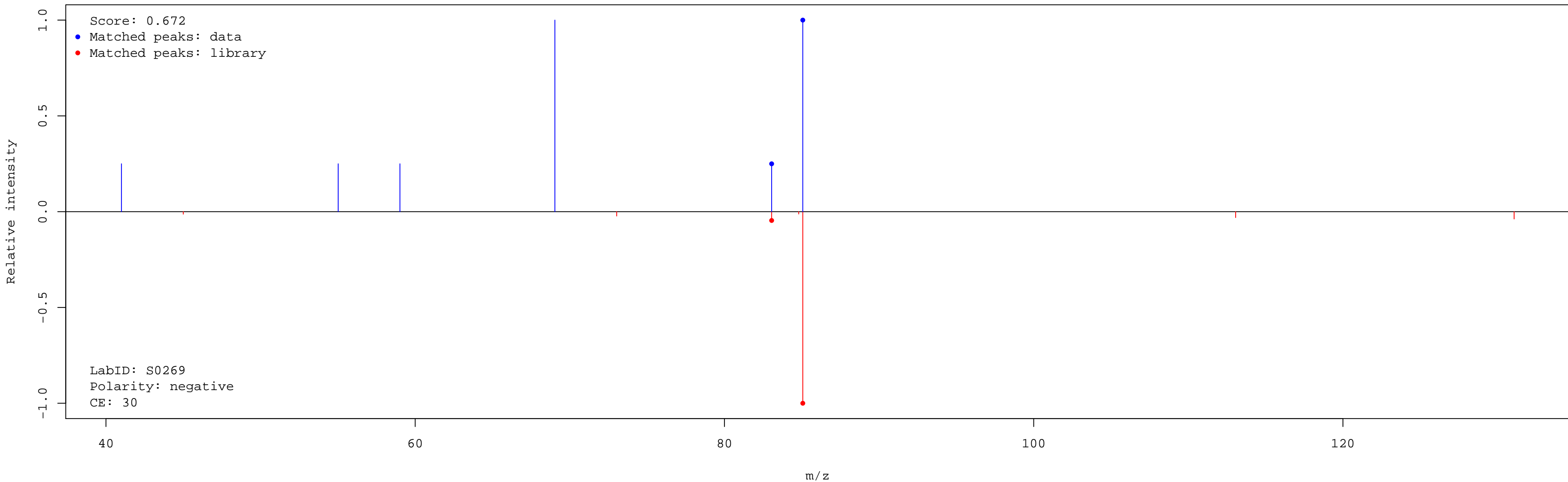

Supplement: Supplementary file 1 [file DataSheet1.ZIP › Supplementary table 1-10 and material 1-3/Material 3-Metlib-MSMS/NEG-Metlib-MSMS/Metlib-MSMS/M131T133_forward/0.672,2-Ethyl-2-Hydroxybutyric acid,(M-H)-.pdf]

Hydroxyisocaproic acid

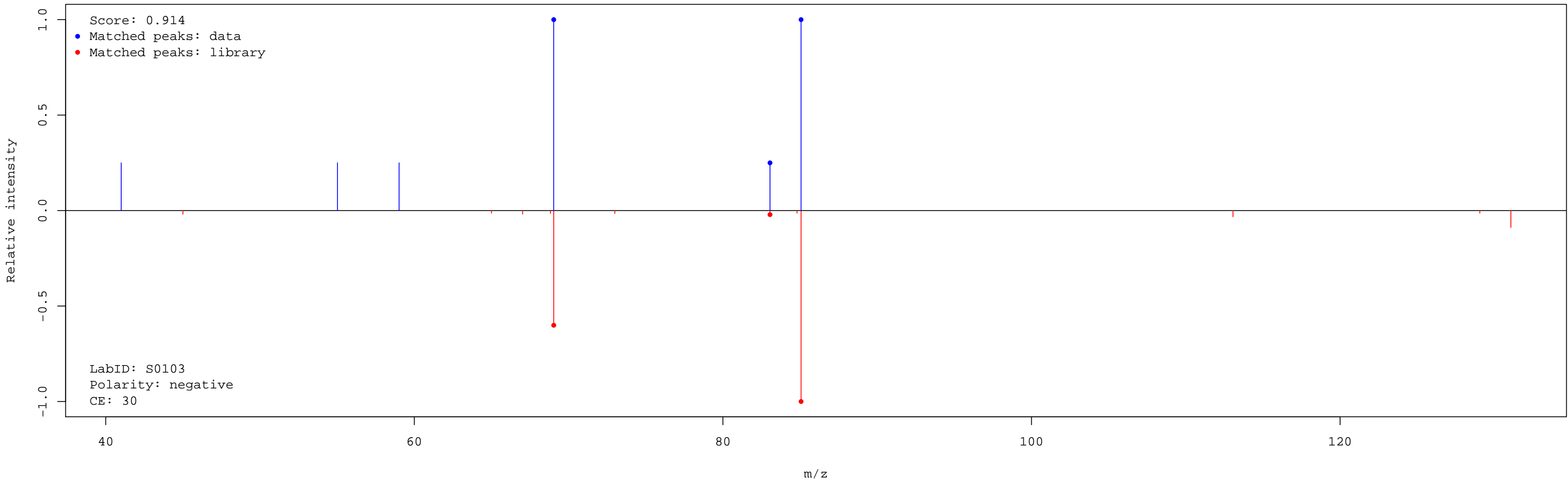

Supplement: Supplementary file 1 [file DataSheet1.ZIP › Supplementary table 1-10 and material 1-3/Material 3-Metlib-MSMS/NEG-Metlib-MSMS/Metlib-MSMS/M131T133_forward/0.914,Hydroxyisocaproic acid,(M-H)-.pdf]

L-Asparagine

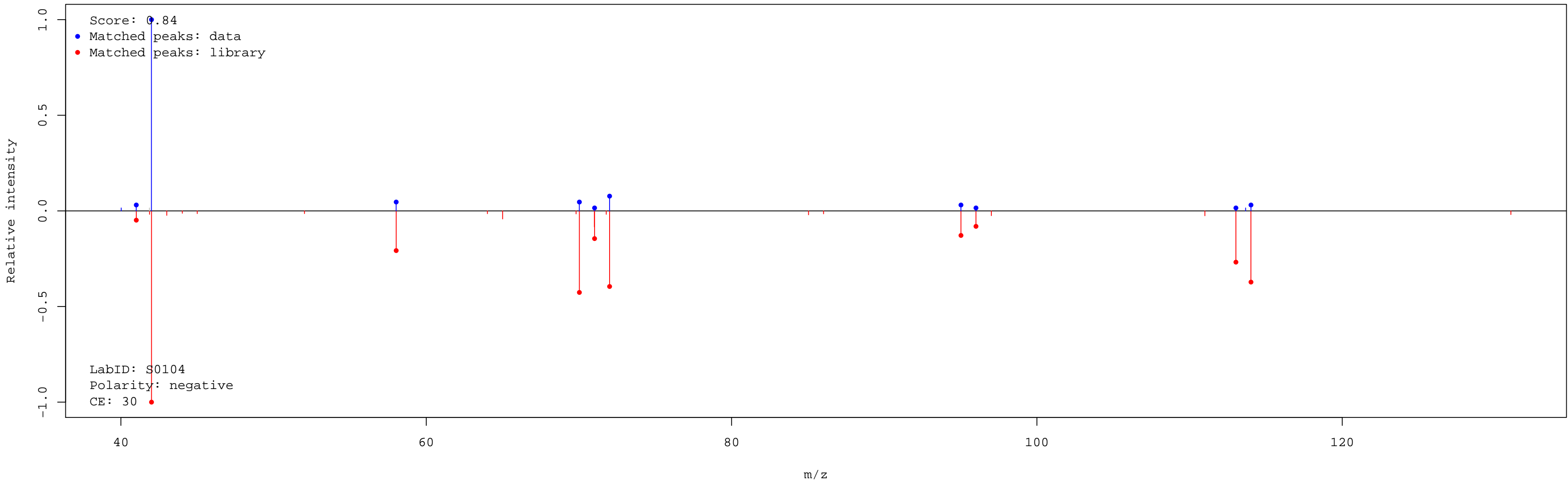

Supplement: Supplementary file 1 [file DataSheet1.ZIP › Supplementary table 1-10 and material 1-3/Material 3-Metlib-MSMS/NEG-Metlib-MSMS/Metlib-MSMS/M131T347_1_forward/0.84,L-Asparagine,(M-H)-.pdf]

L-Asparagine

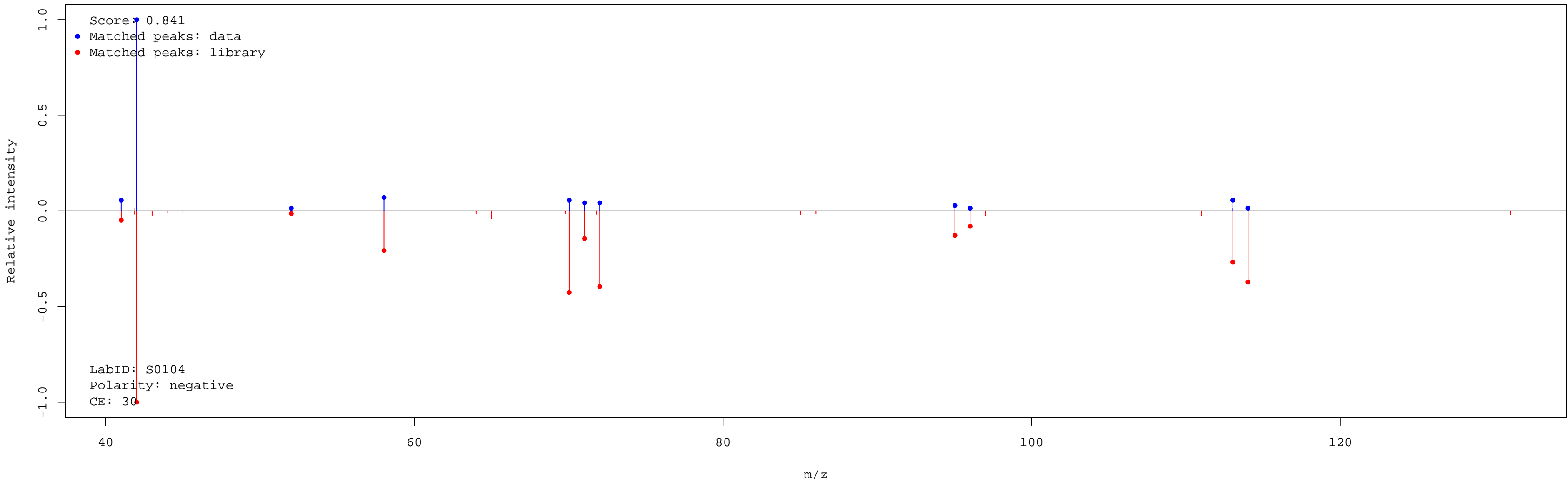

Supplement: Supplementary file 1 [file DataSheet1.ZIP › Supplementary table 1-10 and material 1-3/Material 3-Metlib-MSMS/NEG-Metlib-MSMS/Metlib-MSMS/M131T381_forward/0.841,L-Asparagine,(M-H)-.pdf]

L-Asparagine

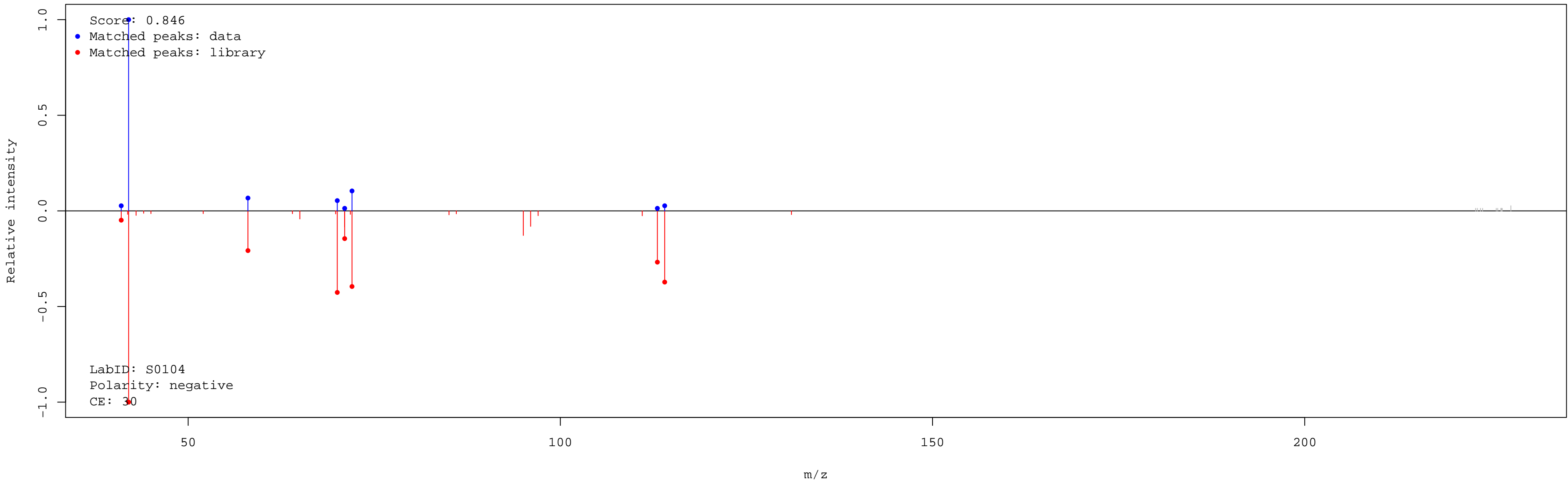

Supplement: Supplementary file 1 [file DataSheet1.ZIP › Supplementary table 1-10 and material 1-3/Material 3-Metlib-MSMS/NEG-Metlib-MSMS/Metlib-MSMS/M131T426_forward/0.846,L-Asparagine,(M-H)-.pdf]

L-Asparagine

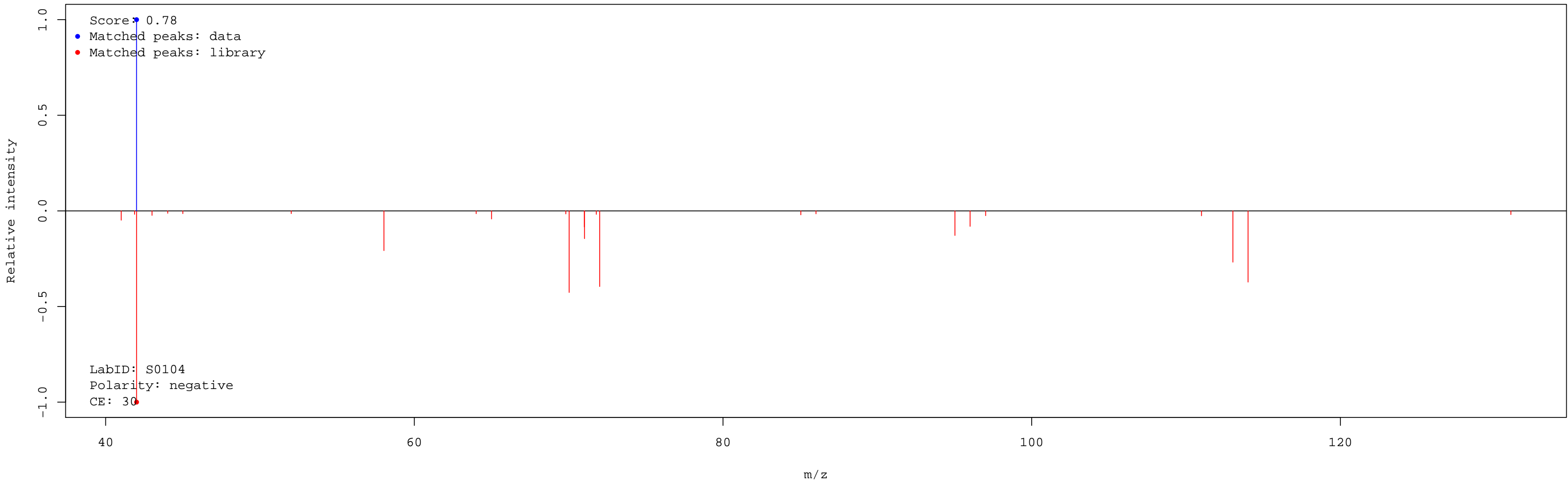

Supplement: Supplementary file 1 [file DataSheet1.ZIP › Supplementary table 1-10 and material 1-3/Material 3-Metlib-MSMS/NEG-Metlib-MSMS/Metlib-MSMS/M132T255_forward/0.78,L-Asparagine,M-.pdf]

# L-Aspartate

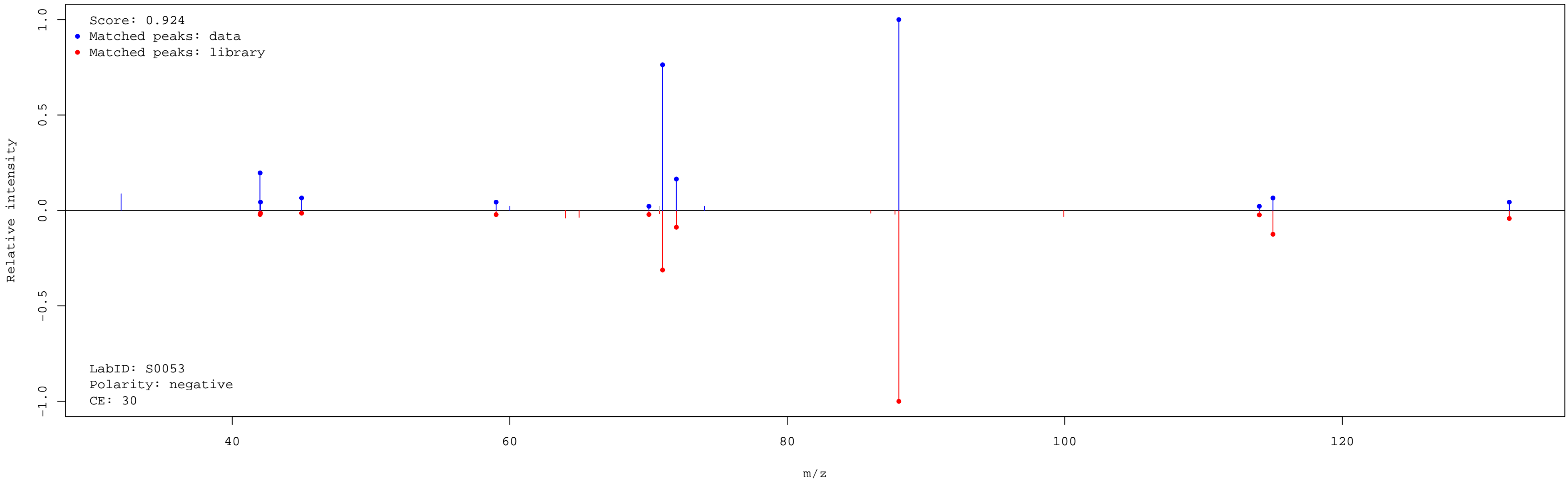

Supplement: Supplementary file 1 [file DataSheet1.ZIP › Supplementary table 1-10 and material 1-3/Material 3-Metlib-MSMS/NEG-Metlib-MSMS/Metlib-MSMS/M132T306_forward/0.924,L-Aspartate,(M-H)-.pdf]

# D-Aspartic acid

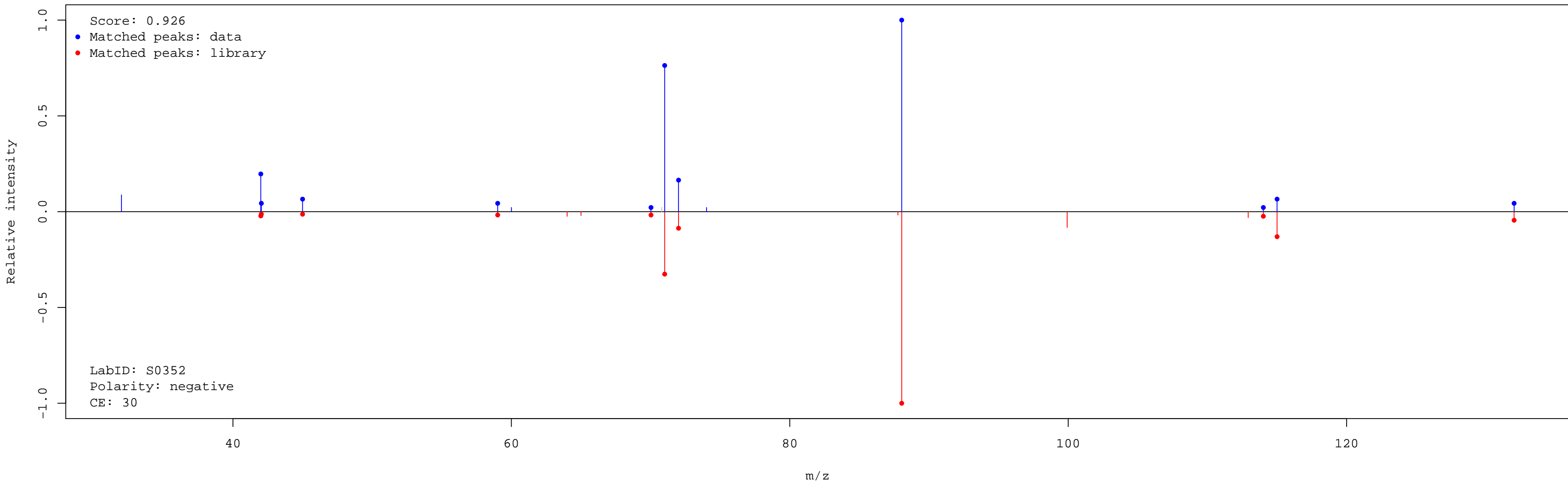

Supplement: Supplementary file 1 [file DataSheet1.ZIP › Supplementary table 1-10 and material 1-3/Material 3-Metlib-MSMS/NEG-Metlib-MSMS/Metlib-MSMS/M132T306_forward/0.926,D-Aspartic acid,(M-H)-.pdf]

L-Asparagine

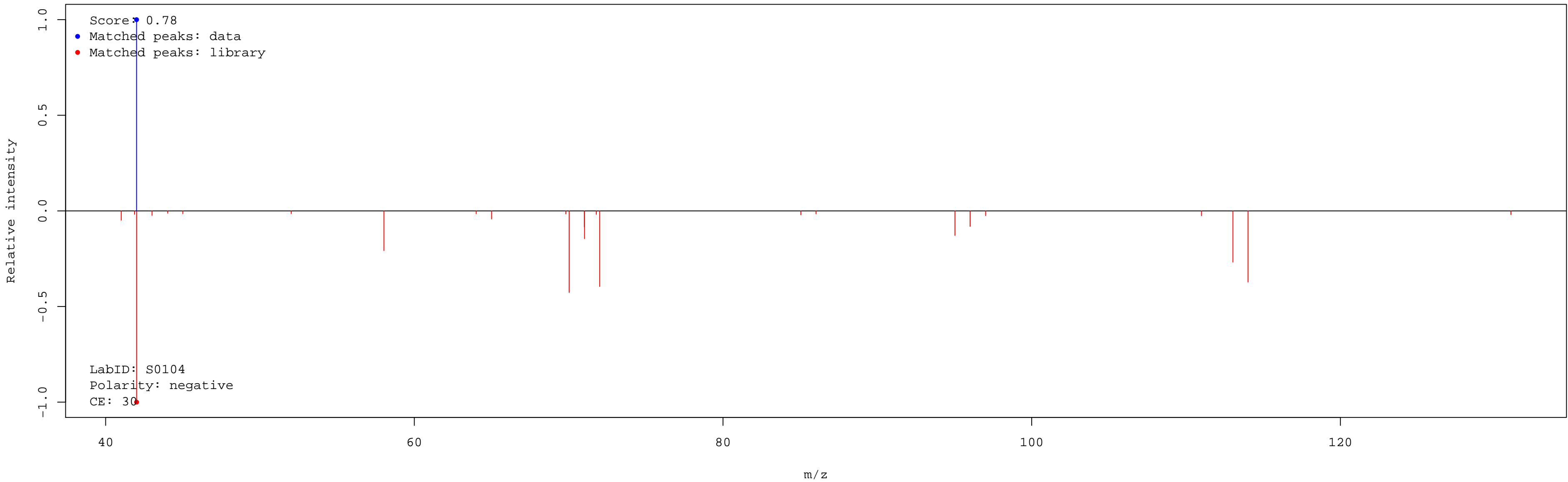

Supplement: Supplementary file 1 [file DataSheet1.ZIP › Supplementary table 1-10 and material 1-3/Material 3-Metlib-MSMS/NEG-Metlib-MSMS/Metlib-MSMS/M132T320_forward/0.78,L-Asparagine,M-.pdf]

# Fumarate

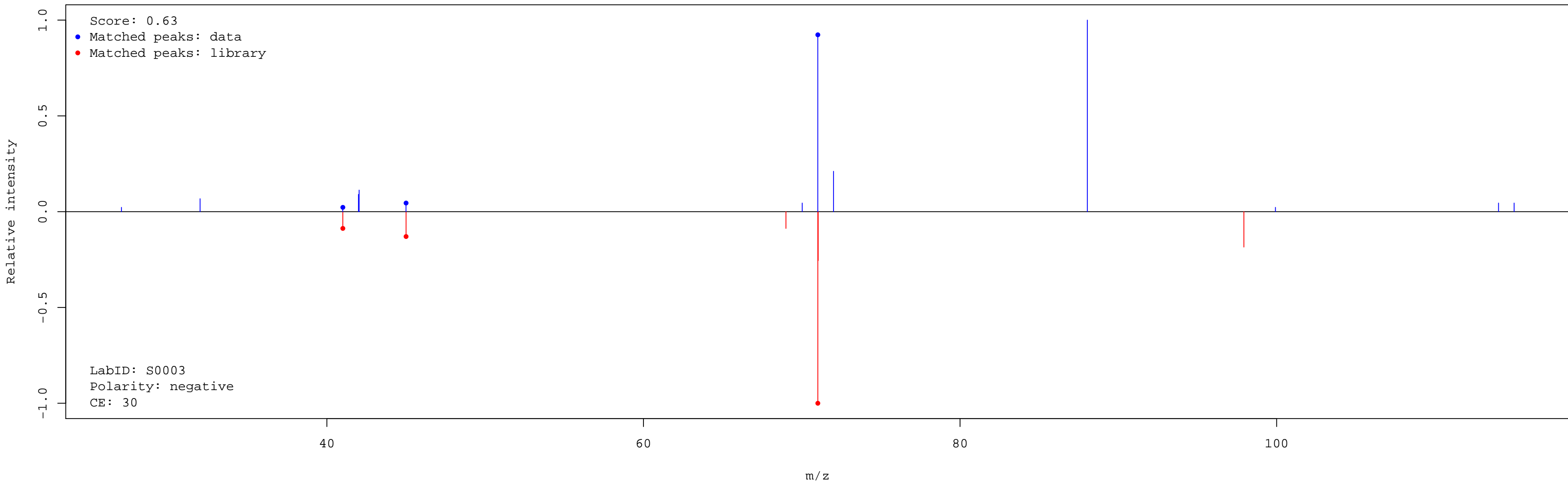

Supplement: Supplementary file 1 [file DataSheet1.ZIP › Supplementary table 1-10 and material 1-3/Material 3-Metlib-MSMS/NEG-Metlib-MSMS/Metlib-MSMS/M132T405_forward/0.63,Fumarate,(M+NH4-2H)-.pdf]

# Maleic acid

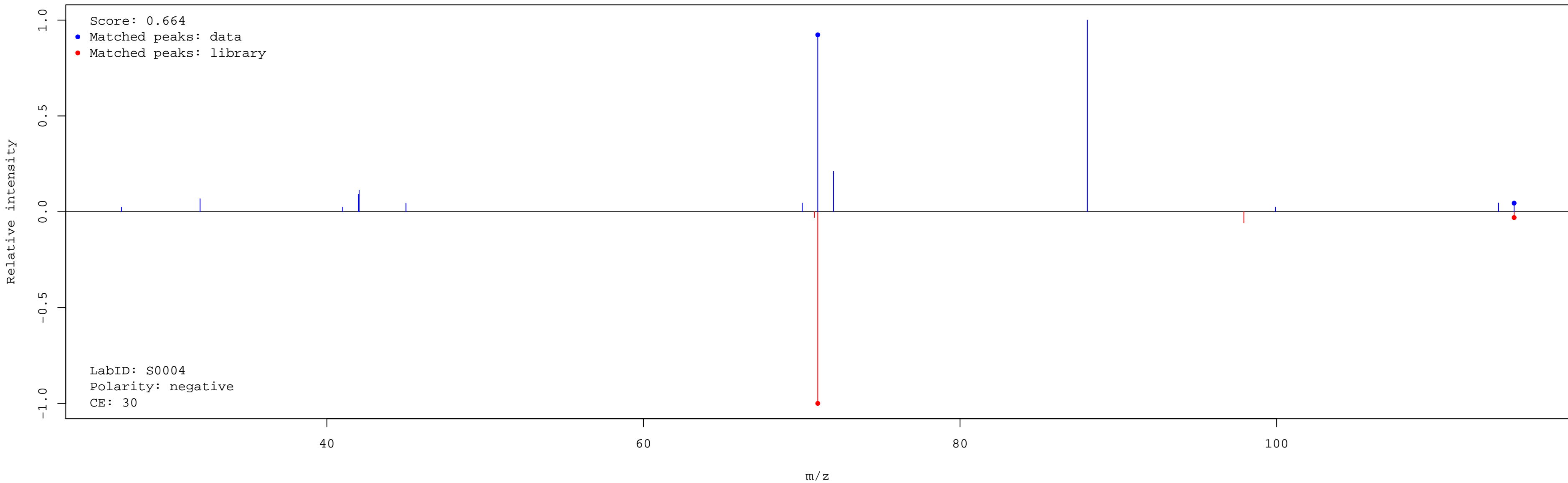

Supplement: Supplementary file 1 [file DataSheet1.ZIP › Supplementary table 1-10 and material 1-3/Material 3-Metlib-MSMS/NEG-Metlib-MSMS/Metlib-MSMS/M132T405_forward/0.664,Maleic acid,(M+NH4-2H)-.pdf]

# L-Aspartate

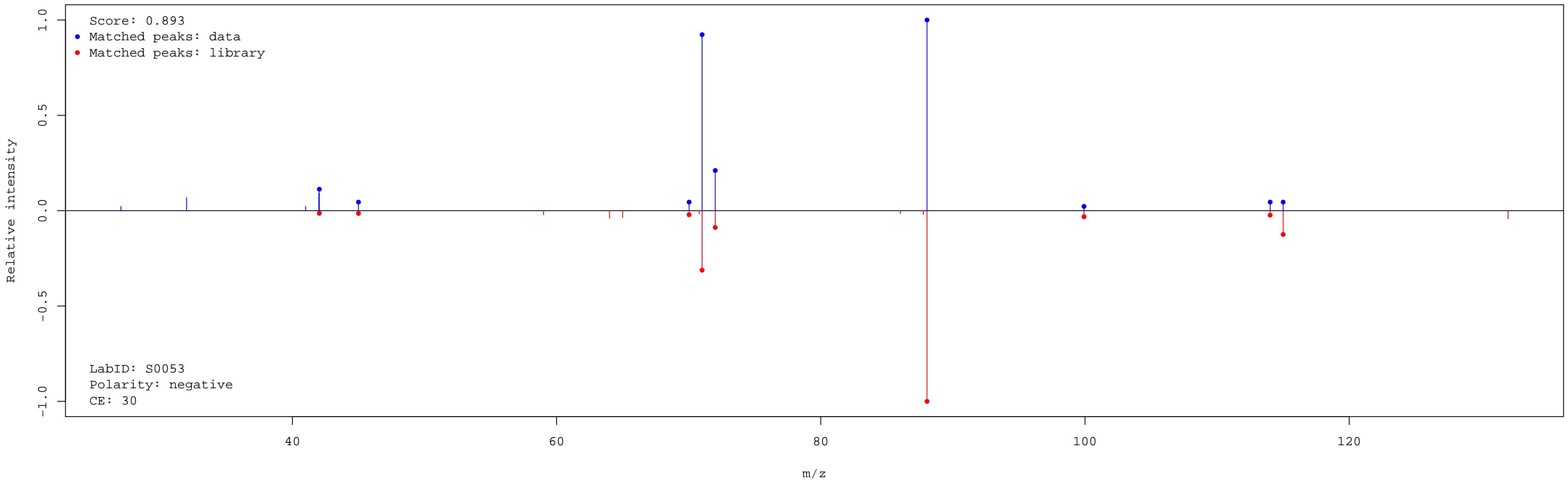

Supplement: Supplementary file 1 [file DataSheet1.ZIP › Supplementary table 1-10 and material 1-3/Material 3-Metlib-MSMS/NEG-Metlib-MSMS/Metlib-MSMS/M132T405_forward/0.893,L-Aspartate,(M-H)-.pdf]

# D-Aspartic acid

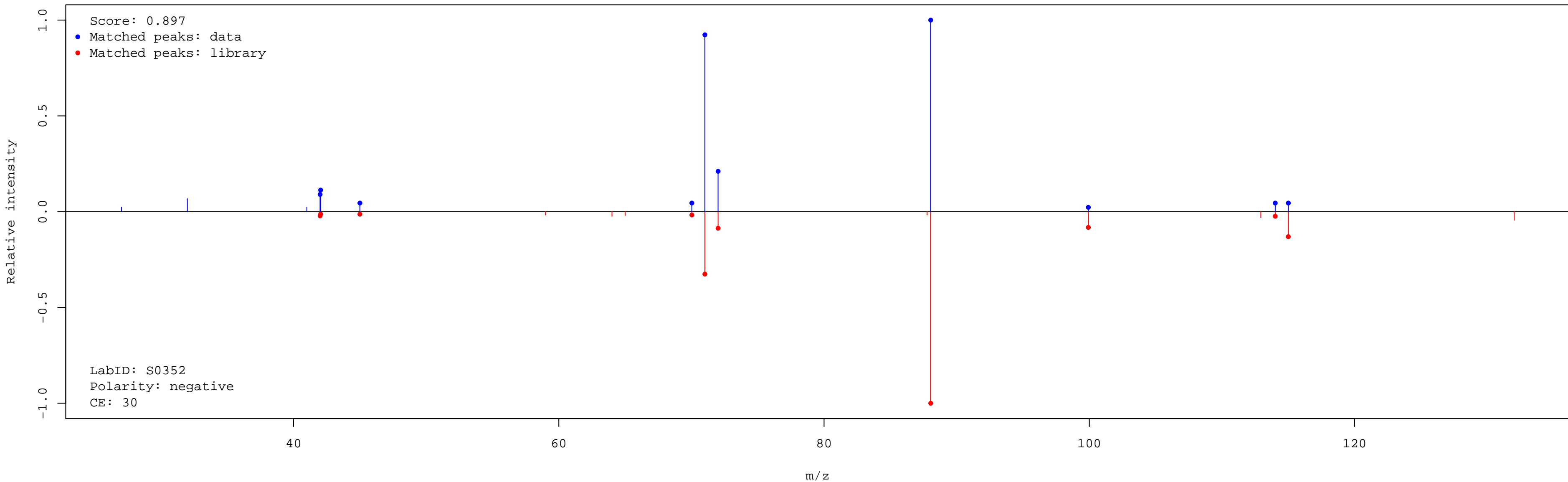

Supplement: Supplementary file 1 [file DataSheet1.ZIP › Supplementary table 1-10 and material 1-3/Material 3-Metlib-MSMS/NEG-Metlib-MSMS/Metlib-MSMS/M132T405_forward/0.897,D-Aspartic acid,(M-H)-.pdf]

# L-Aspartate

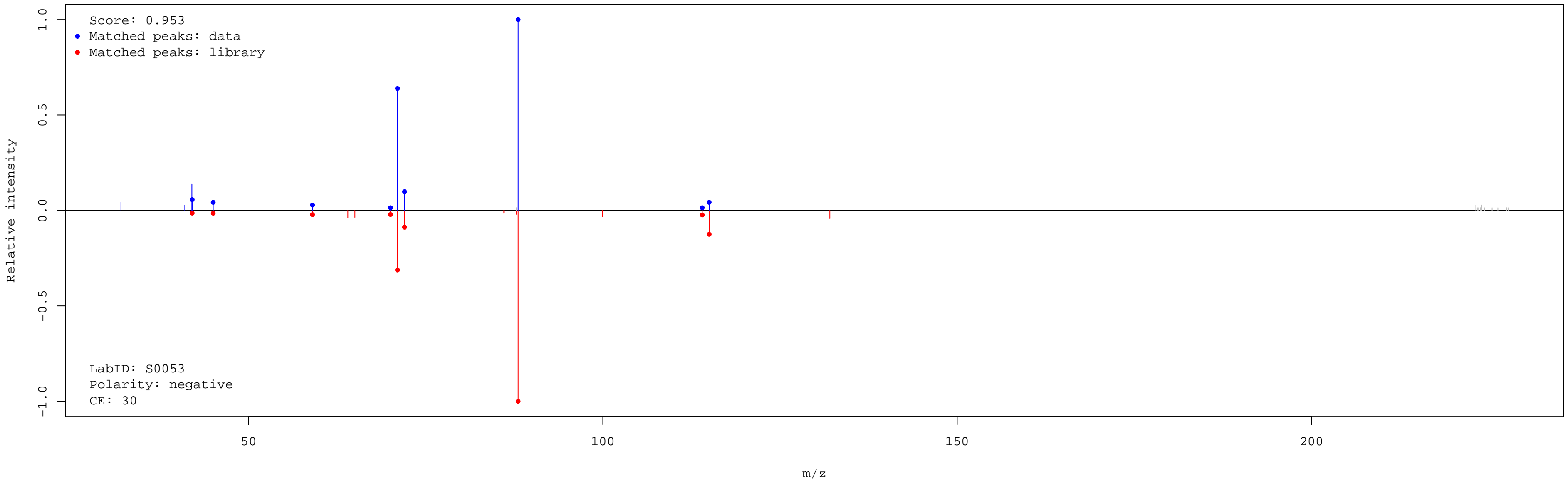

Supplement: Supplementary file 1 [file DataSheet1.ZIP › Supplementary table 1-10 and material 1-3/Material 3-Metlib-MSMS/NEG-Metlib-MSMS/Metlib-MSMS/M132T469_forward/0.953,L-Aspartate,(M-H)-.pdf]

# D-Aspartic acid

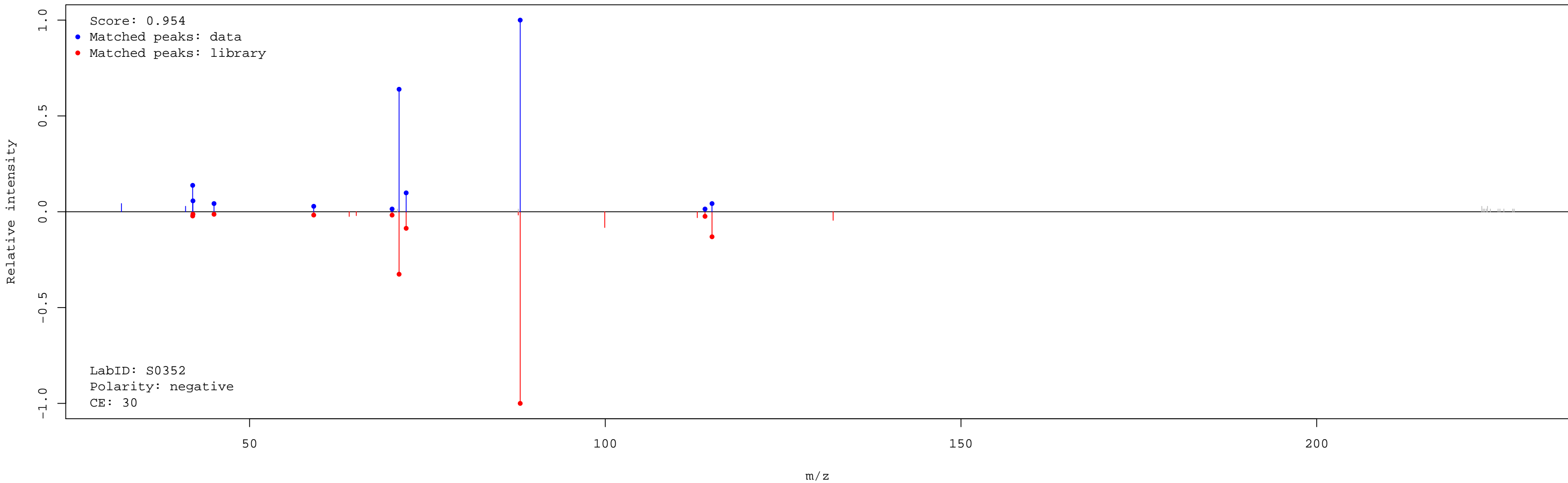

Supplement: Supplementary file 1 [file DataSheet1.ZIP › Supplementary table 1-10 and material 1-3/Material 3-Metlib-MSMS/NEG-Metlib-MSMS/Metlib-MSMS/M132T469_forward/0.954,D-Aspartic acid,(M-H)-.pdf]

# L-Aspartate

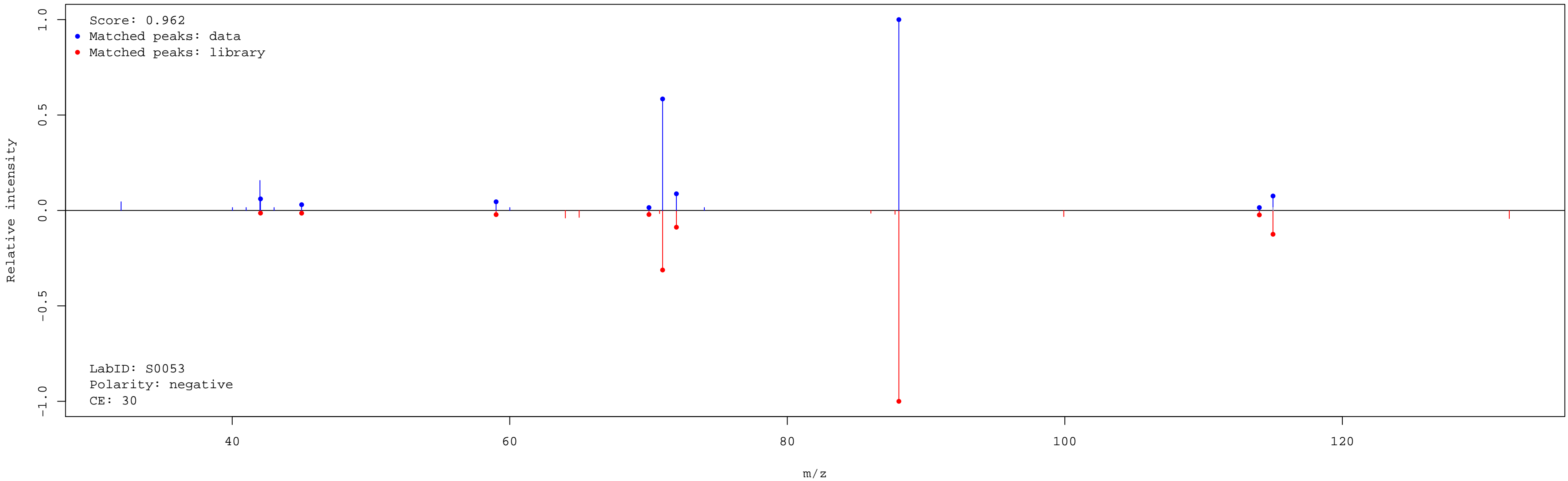

Supplement: Supplementary file 1 [file DataSheet1.ZIP › Supplementary table 1-10 and material 1-3/Material 3-Metlib-MSMS/NEG-Metlib-MSMS/Metlib-MSMS/M132T500_forward/0.962,L-Aspartate,(M-H)-.pdf]

# D-Aspartic acid

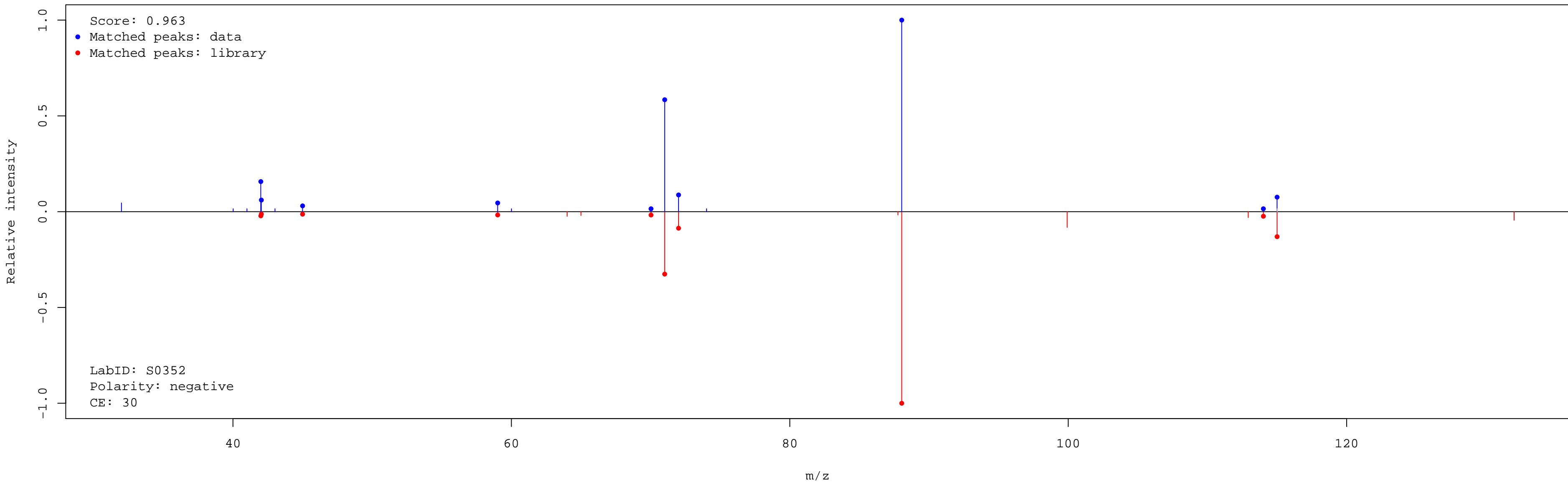

Supplement: Supplementary file 1 [file DataSheet1.ZIP › Supplementary table 1-10 and material 1-3/Material 3-Metlib-MSMS/NEG-Metlib-MSMS/Metlib-MSMS/M132T500_forward/0.963,D-Aspartic acid,(M-H)-.pdf]

# 2,3-Dihydroxy-3-methylbutyric acid

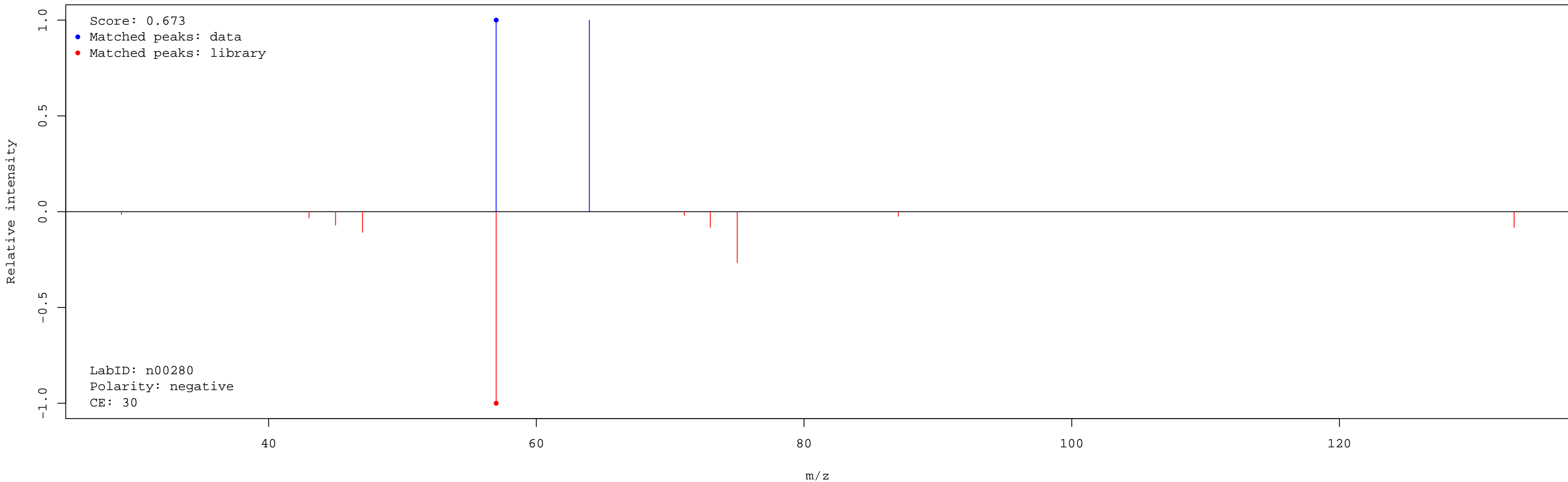

Supplement: Supplementary file 1 [file DataSheet1.ZIP › Supplementary table 1-10 and material 1-3/Material 3-Metlib-MSMS/NEG-Metlib-MSMS/Metlib-MSMS/M133T108_forward/0.673,2,3-Dihydroxy-3-methylbutyric acid,(M-H)-.pdf]

# Hydroxyacetone

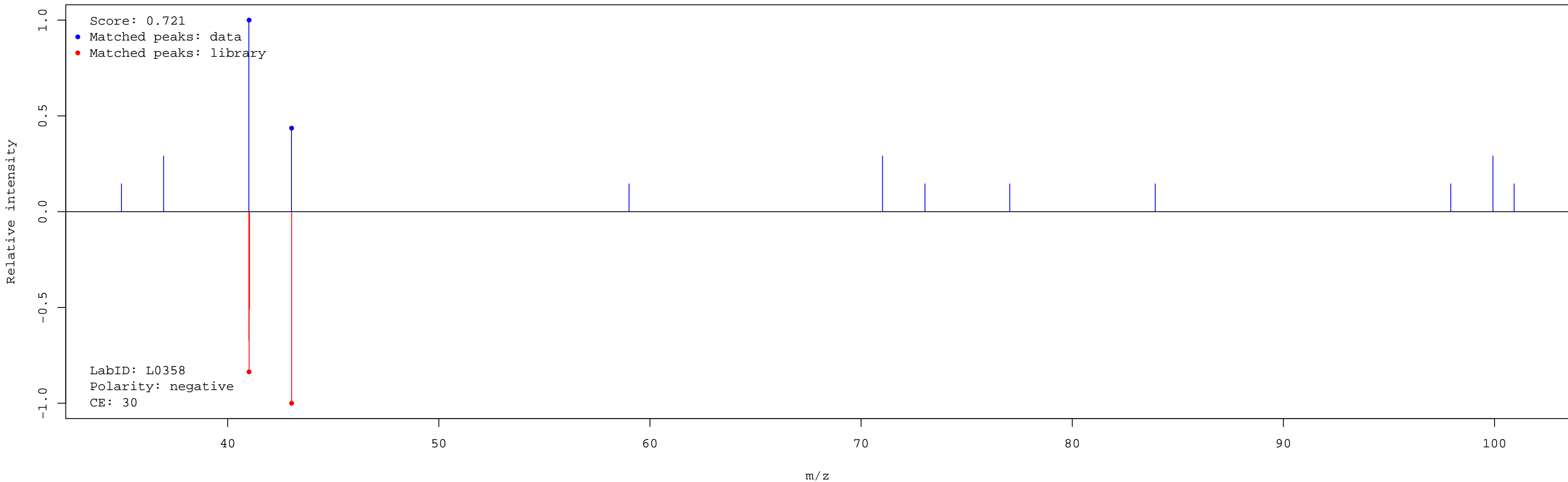

Supplement: Supplementary file 1 [file DataSheet1.ZIP › Supplementary table 1-10 and material 1-3/Material 3-Metlib-MSMS/NEG-Metlib-MSMS/Metlib-MSMS/M133T31_2_forward/0.721,Hydroxyacetone,(M+CH3COO)-.pdf]

L-Malic acid

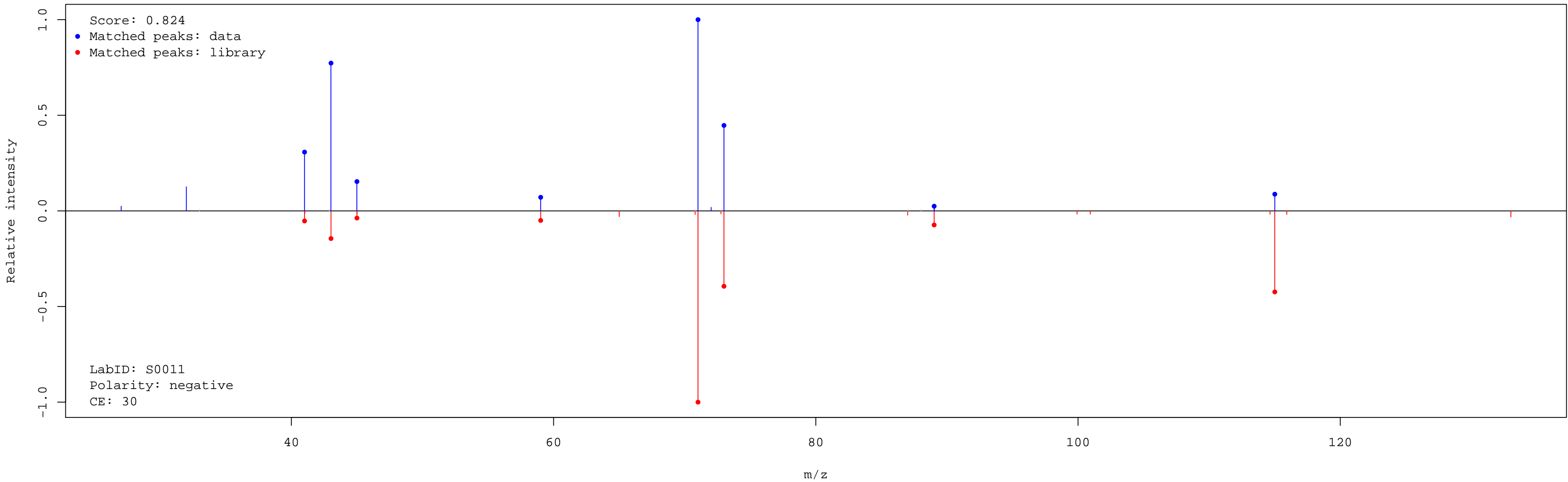

Supplement: Supplementary file 1 [file DataSheet1.ZIP › Supplementary table 1-10 and material 1-3/Material 3-Metlib-MSMS/NEG-Metlib-MSMS/Metlib-MSMS/M133T411_forward/0.824,L-Malic acid,(M-H)-.pdf]

# Adenine

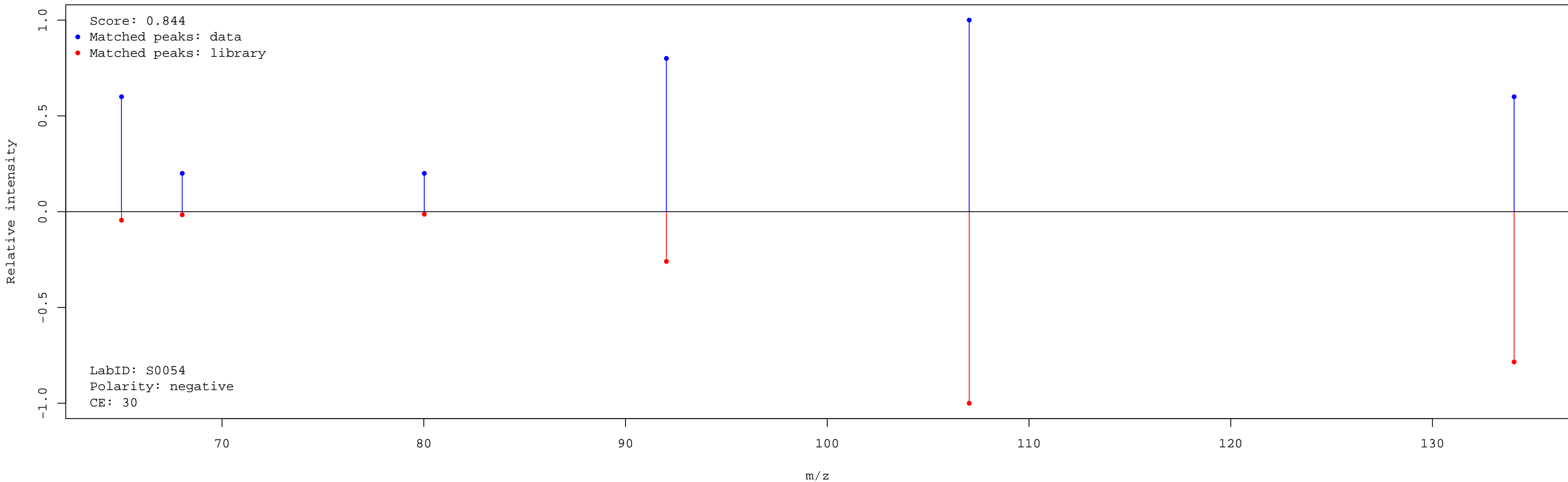

Supplement: Supplementary file 1 [file DataSheet1.ZIP › Supplementary table 1-10 and material 1-3/Material 3-Metlib-MSMS/NEG-Metlib-MSMS/Metlib-MSMS/M134T106_2_forward/0.844,Adenine,(M-H)-.pdf]

# Hypoxanthine

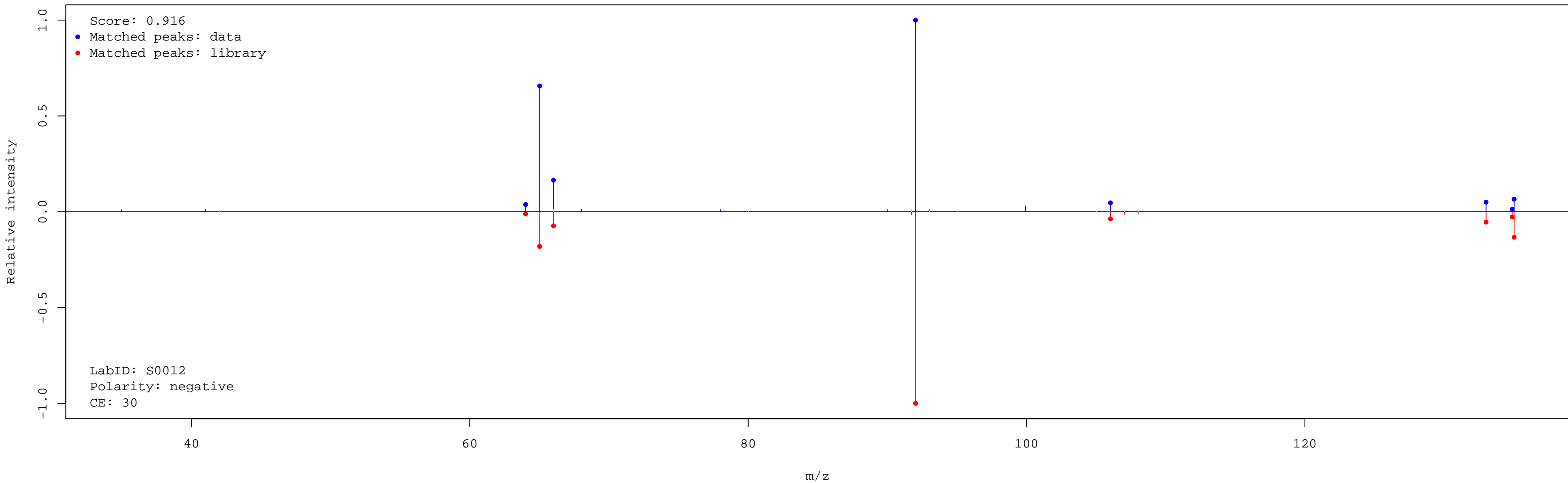

Supplement: Supplementary file 1 [file DataSheet1.ZIP › Supplementary table 1-10 and material 1-3/Material 3-Metlib-MSMS/NEG-Metlib-MSMS/Metlib-MSMS/M135T171_2_forward/0.916,Hypoxanthine,(M-H)-.pdf]

# Glycolate

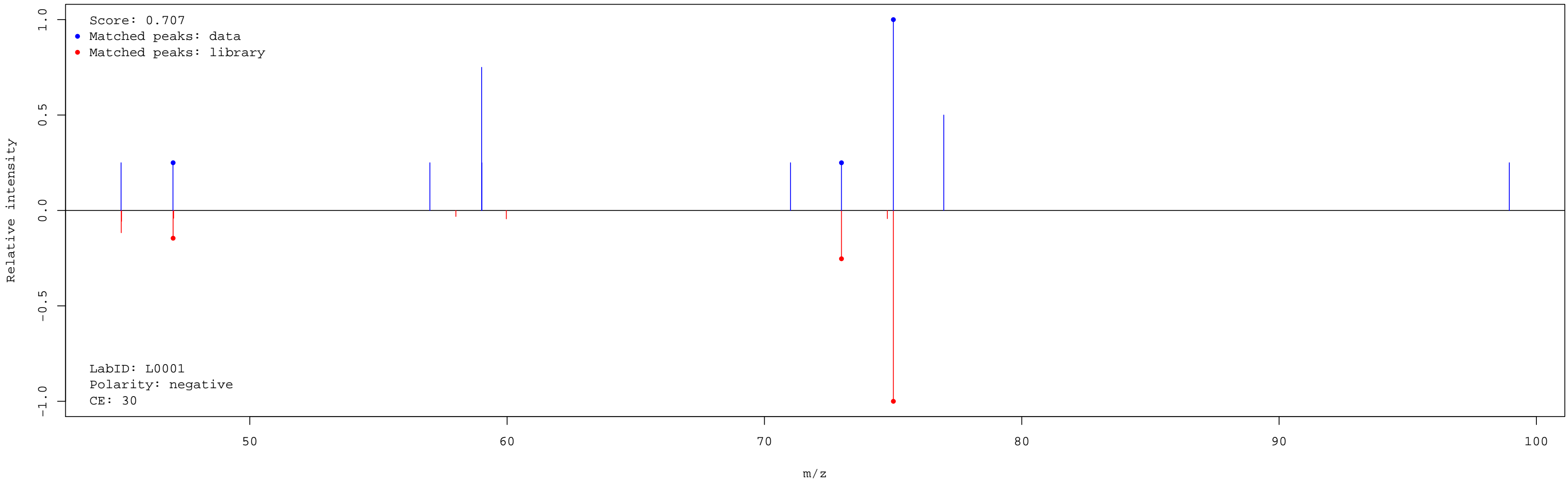

Supplement: Supplementary file 1 [file DataSheet1.ZIP › Supplementary table 1-10 and material 1-3/Material 3-Metlib-MSMS/NEG-Metlib-MSMS/Metlib-MSMS/M135T386_forward/0.707,Glycolate,(M+CH3COO)-.pdf]

# L-Threonate

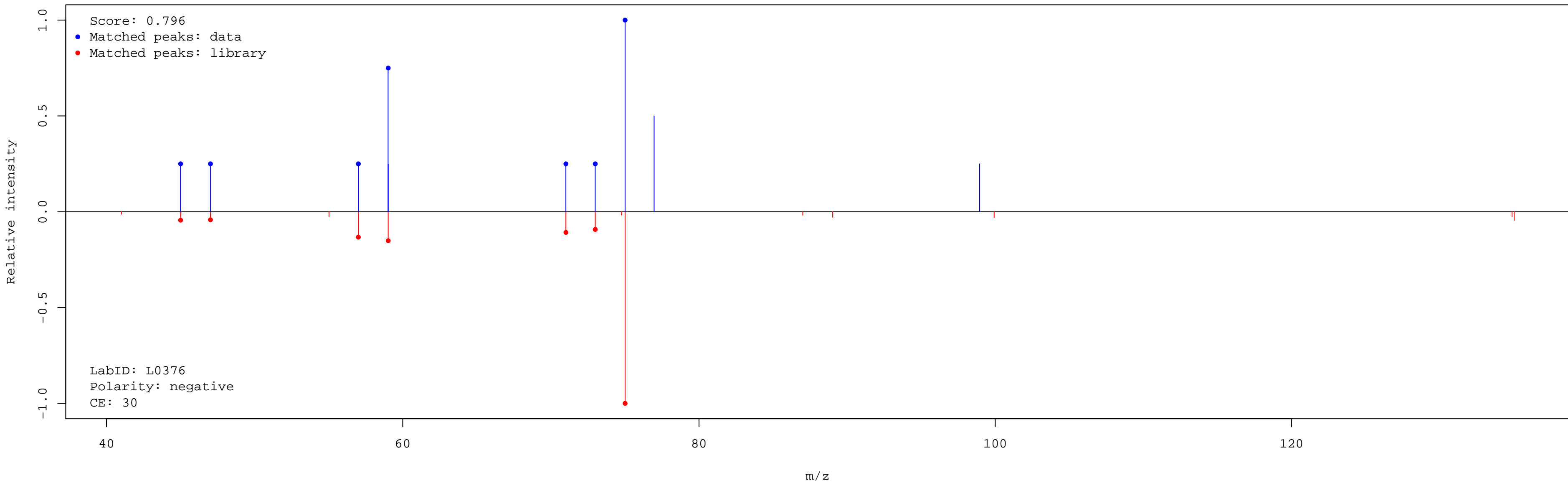

Supplement: Supplementary file 1 [file DataSheet1.ZIP › Supplementary table 1-10 and material 1-3/Material 3-Metlib-MSMS/NEG-Metlib-MSMS/Metlib-MSMS/M135T386_forward/0.796,L-Threonate,(M-H)-.pdf]

# 4-Aminobenzoate

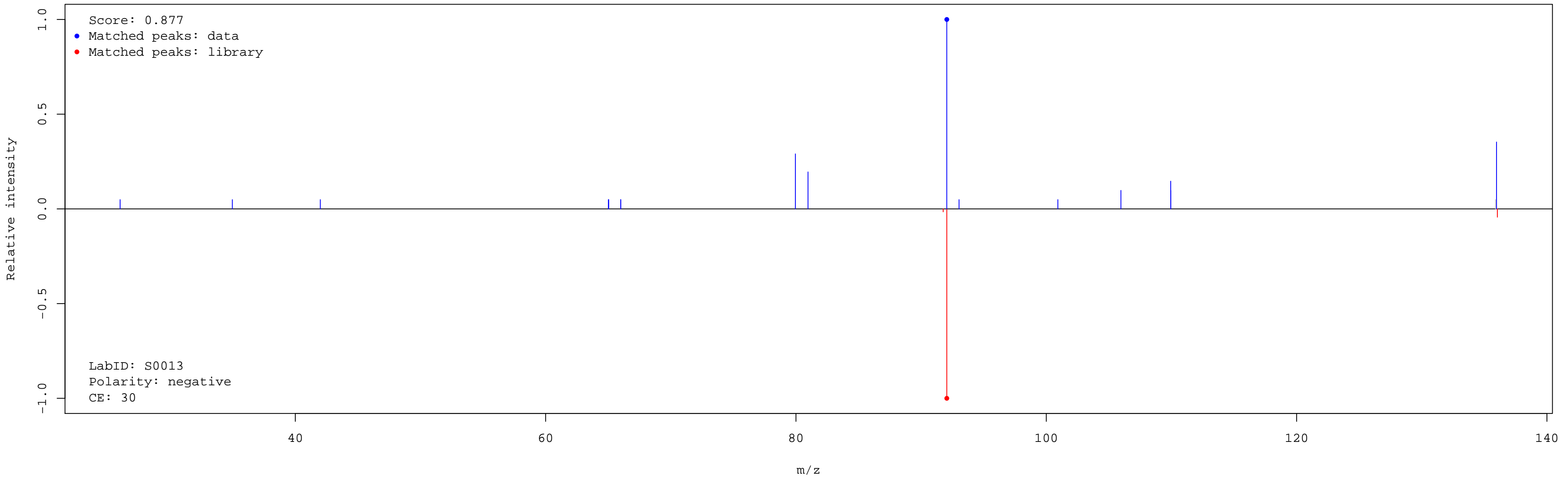

Supplement: Supplementary file 1 [file DataSheet1.ZIP › Supplementary table 1-10 and material 1-3/Material 3-Metlib-MSMS/NEG-Metlib-MSMS/Metlib-MSMS/M136T101_2_forward/0.877,4-Aminobenzoate,(M-H)-.pdf]

Anthranilic acid (Vitamin L1)

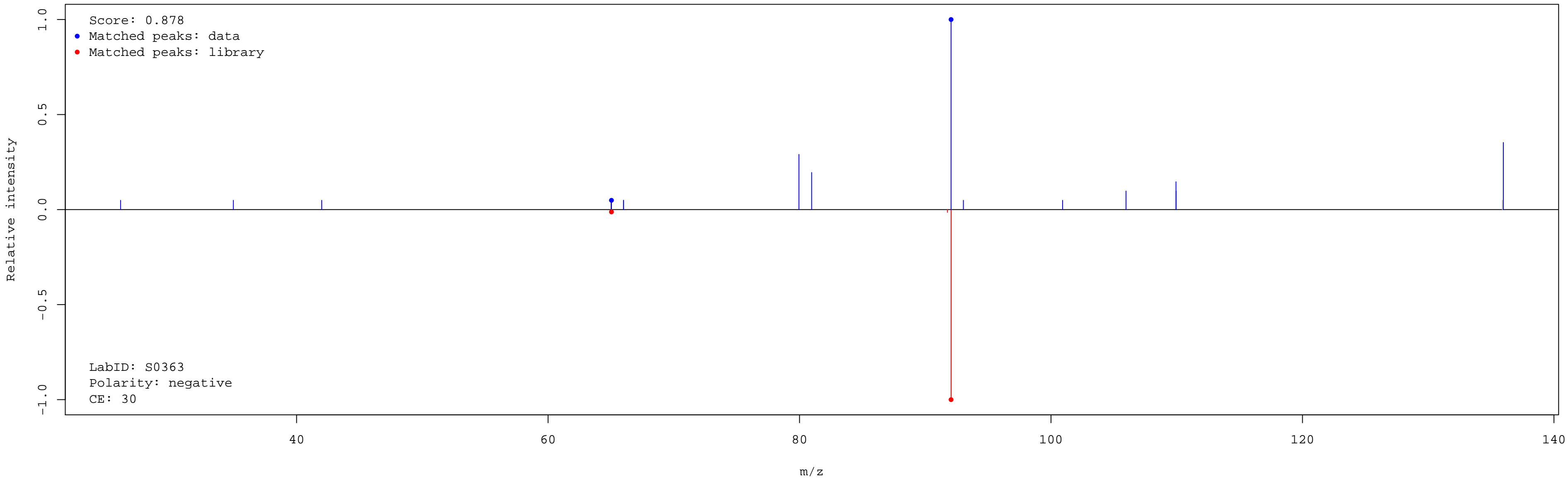

Supplement: Supplementary file 1 [file DataSheet1.ZIP › Supplementary table 1-10 and material 1-3/Material 3-Metlib-MSMS/NEG-Metlib-MSMS/Metlib-MSMS/M136T101_2_forward/0.878,Anthranilic acid (Vitamin L1),(M-H)-.pdf]

# Urocanic acid

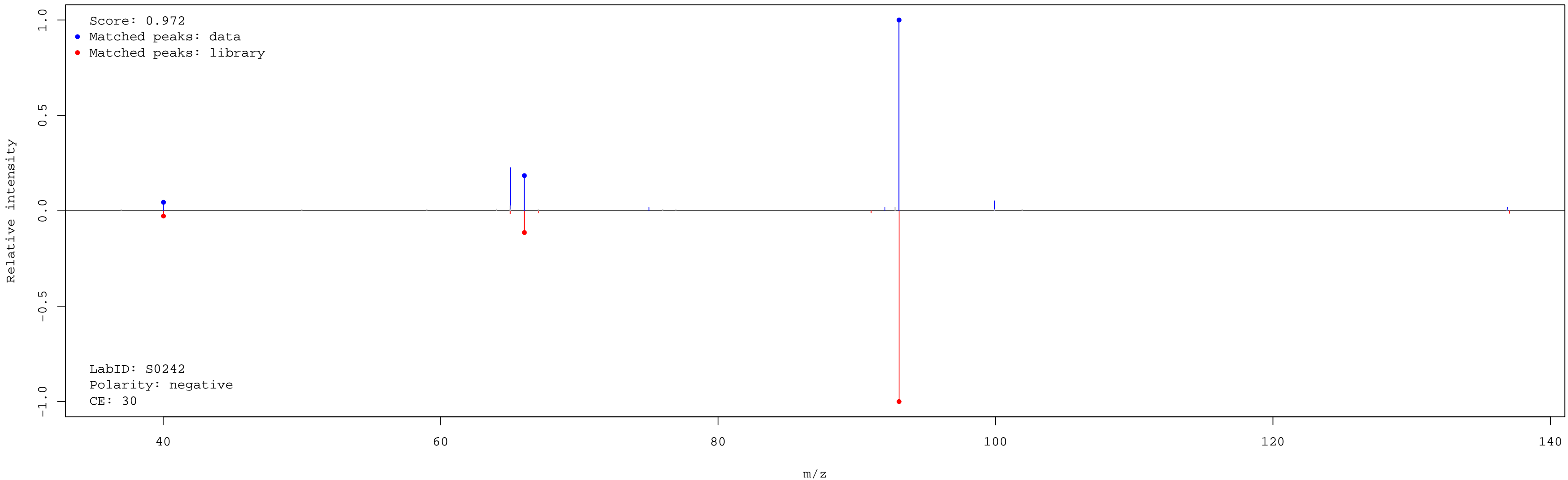

Supplement: Supplementary file 1 [file DataSheet1.ZIP › Supplementary table 1-10 and material 1-3/Material 3-Metlib-MSMS/NEG-Metlib-MSMS/Metlib-MSMS/M137T186_forward/0.972,Urocanic acid,(M-H)-.pdf]

# Urocanic acid

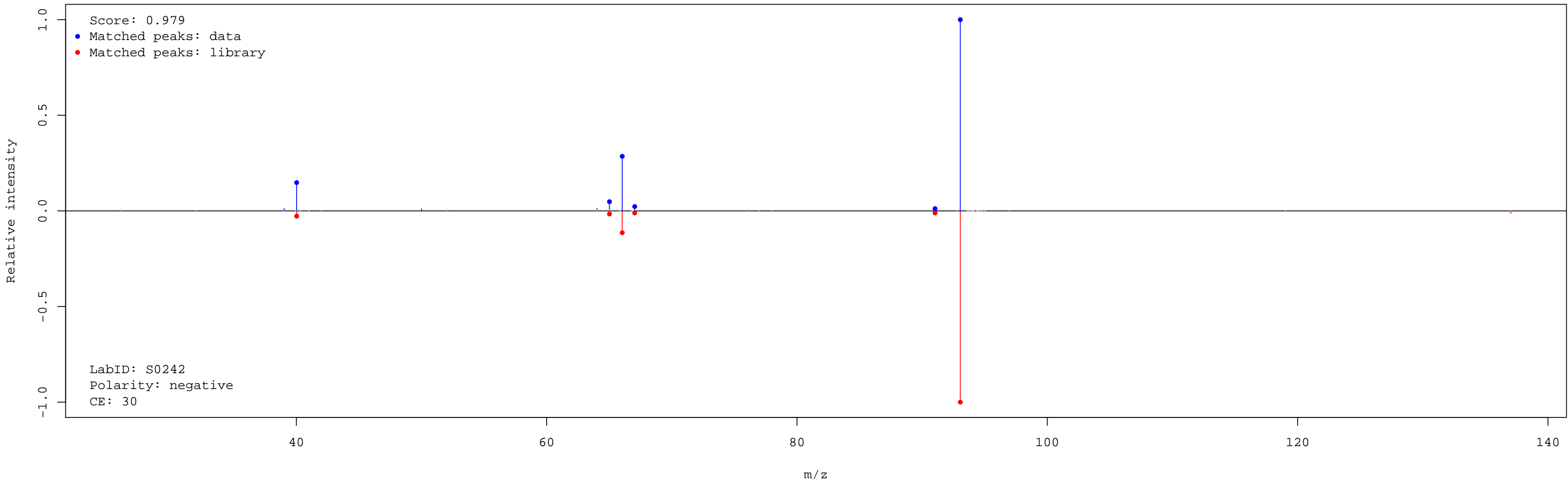

Supplement: Supplementary file 1 [file DataSheet1.ZIP › Supplementary table 1-10 and material 1-3/Material 3-Metlib-MSMS/NEG-Metlib-MSMS/Metlib-MSMS/M137T290_forward/0.979,Urocanic acid,(M-H)-.pdf]

# Urocanic acid

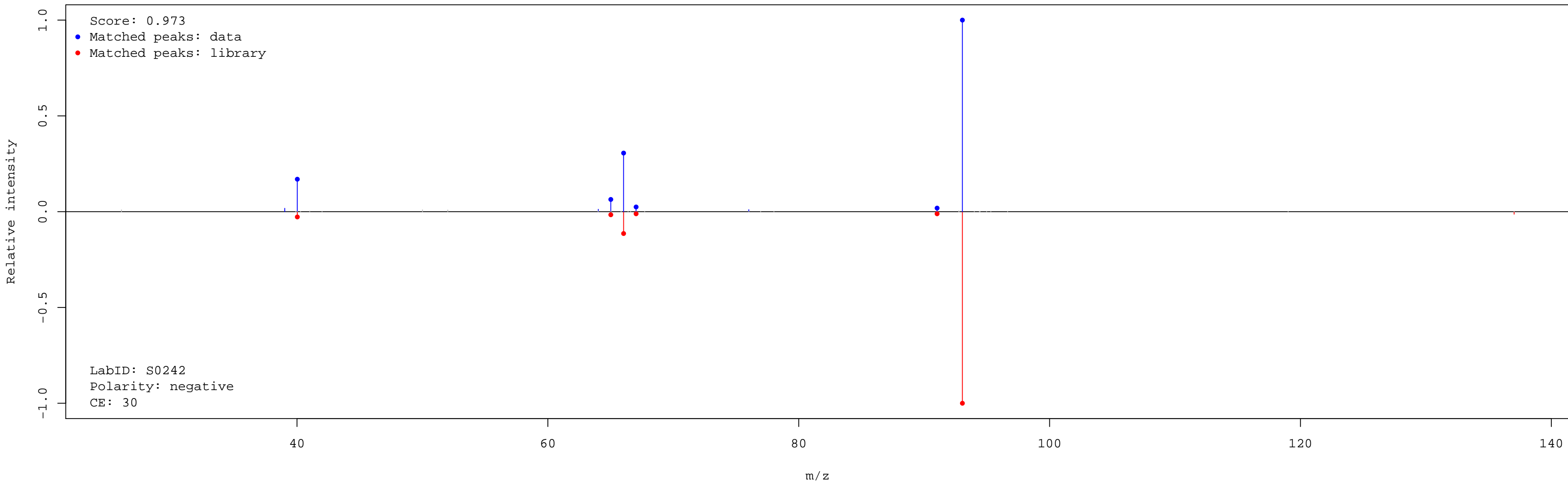

Supplement: Supplementary file 1 [file DataSheet1.ZIP › Supplementary table 1-10 and material 1-3/Material 3-Metlib-MSMS/NEG-Metlib-MSMS/Metlib-MSMS/M137T318_forward/0.973,Urocanic acid,(M-H)-.pdf]

# Salicylic acid

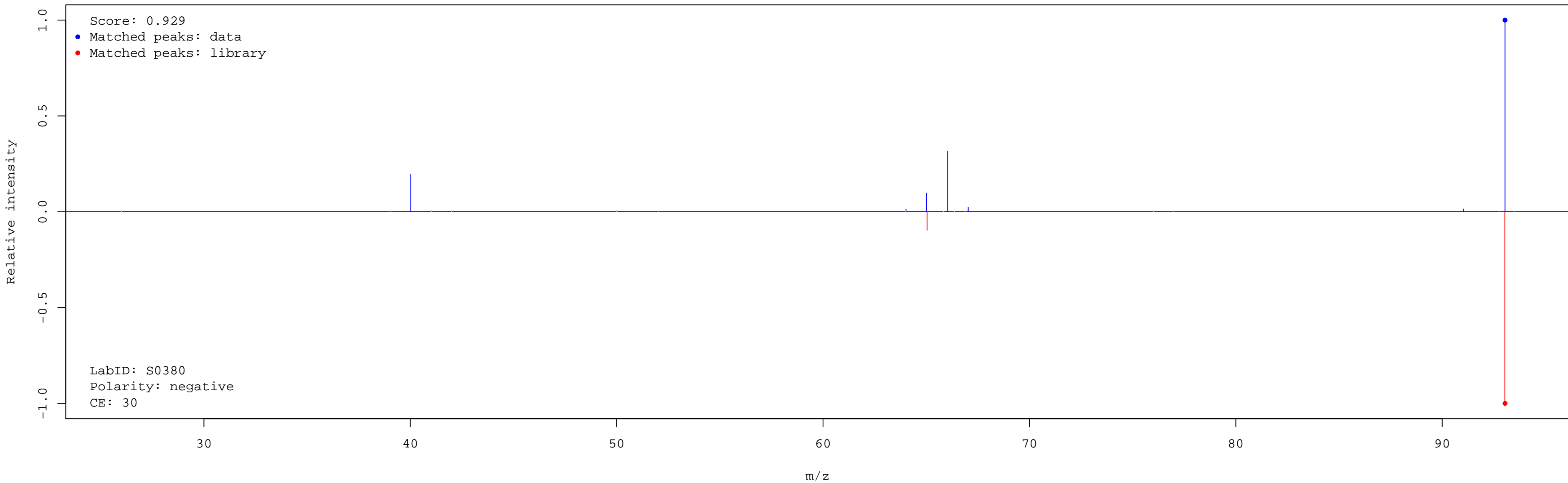

Supplement: Supplementary file 1 [file DataSheet1.ZIP › Supplementary table 1-10 and material 1-3/Material 3-Metlib-MSMS/NEG-Metlib-MSMS/Metlib-MSMS/M137T347_2_forward/0.929,Salicylic acid,(M-H)-.pdf]

# 4-Hydroxybenzoate

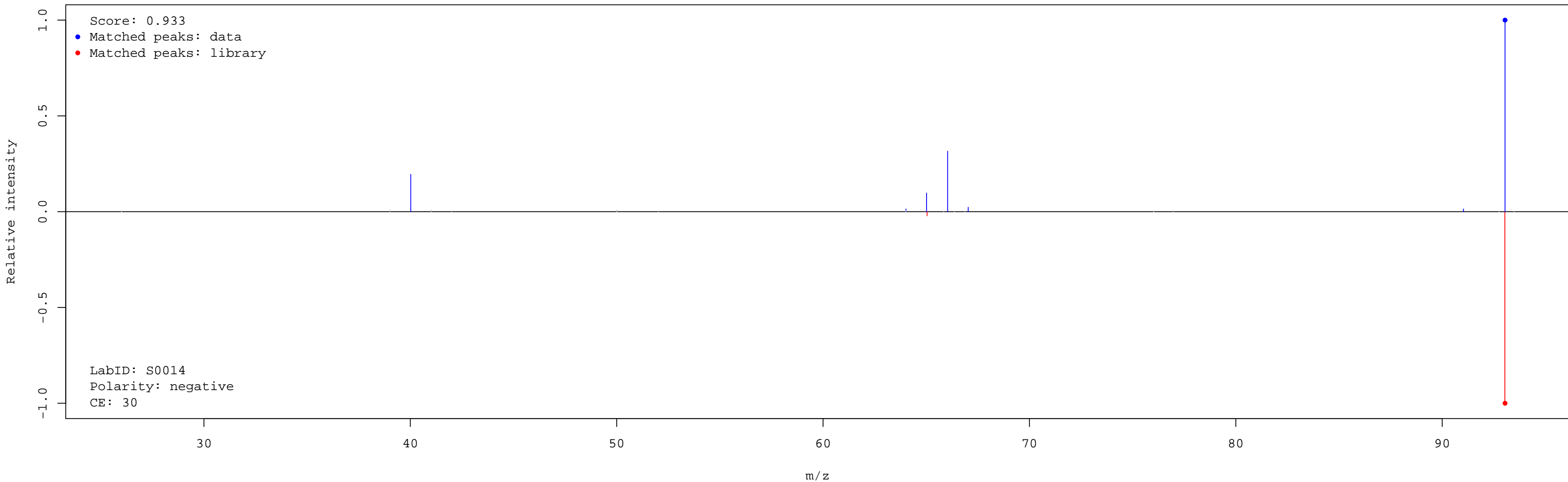

Supplement: Supplementary file 1 [file DataSheet1.ZIP › Supplementary table 1-10 and material 1-3/Material 3-Metlib-MSMS/NEG-Metlib-MSMS/Metlib-MSMS/M137T347_2_forward/0.933,4-Hydroxybenzoate,(M-H)-.pdf]

# Urocanic acid

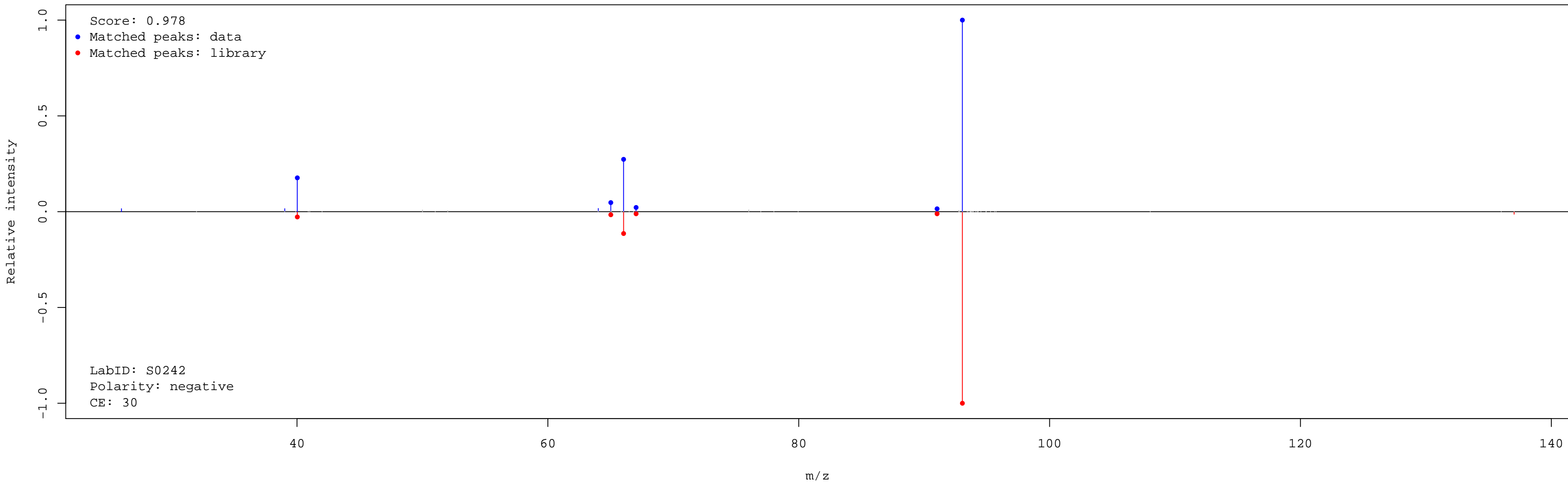

Supplement: Supplementary file 1 [file DataSheet1.ZIP › Supplementary table 1-10 and material 1-3/Material 3-Metlib-MSMS/NEG-Metlib-MSMS/Metlib-MSMS/M137T389_forward/0.978,Urocanic acid,(M-H)-.pdf]

# 3-Hydroxyisovaleric acid

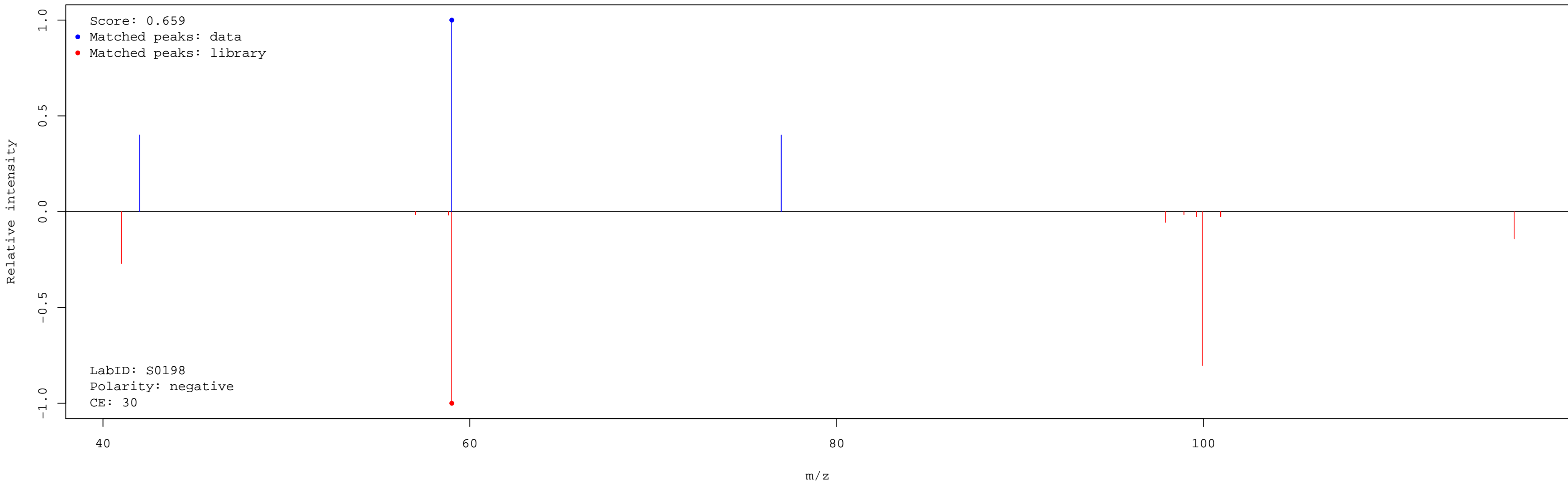

Supplement: Supplementary file 1 [file DataSheet1.ZIP › Supplementary table 1-10 and material 1-3/Material 3-Metlib-MSMS/NEG-Metlib-MSMS/Metlib-MSMS/M139T113_forward/0.659,3-Hydroxyisovaleric acid,(M+Na-2H)-.pdf]

# Acetyl phosphate

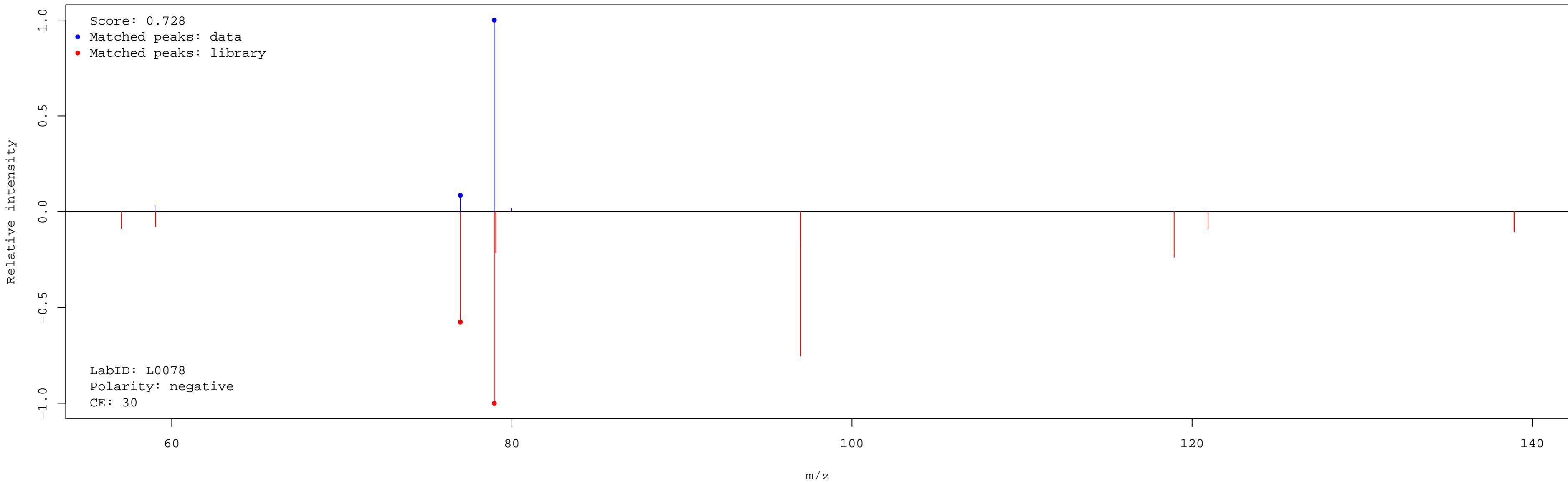

Supplement: Supplementary file 1 [file DataSheet1.ZIP › Supplementary table 1-10 and material 1-3/Material 3-Metlib-MSMS/NEG-Metlib-MSMS/Metlib-MSMS/M139T441_forward/0.728,Acetyl phosphate,(M-H)-.pdf]
